# Supplementary material for: A little frog leaps a long way: compounded colonizations of the Indian Subcontinent discovered in the tiny Oriental frog genus Microhyla (Amphibia: Microhylidae)
Source: PeerJ. 2020 Jul 3;8:e9411. doi: 10.7717/peerj.9411 (PMC7337035; doi:10.7717/peerj.9411)
Supplement: Supplemental Information 1 — Pink shading corresponds to Microhyla distribution; red circles denote localities of samples for which sequences were available via GenBank; green circles denote localities of samples for which sequences were generated in this study. For locality information see Table S1. Base Map created using simplemappr.net. [file peerj-08-9411-s001.pdf]

Supplementary Figure S1. Geographic sampling in the present study.

Pink shading corresponds to *Microhyla* distribution; red circles denote localities of samples for which sequences were available via GenBank; green circles denote localities of samples for which sequences were generated in this study. For locality information see Supplementary Table S1. Base Map created using simplemappr.net.

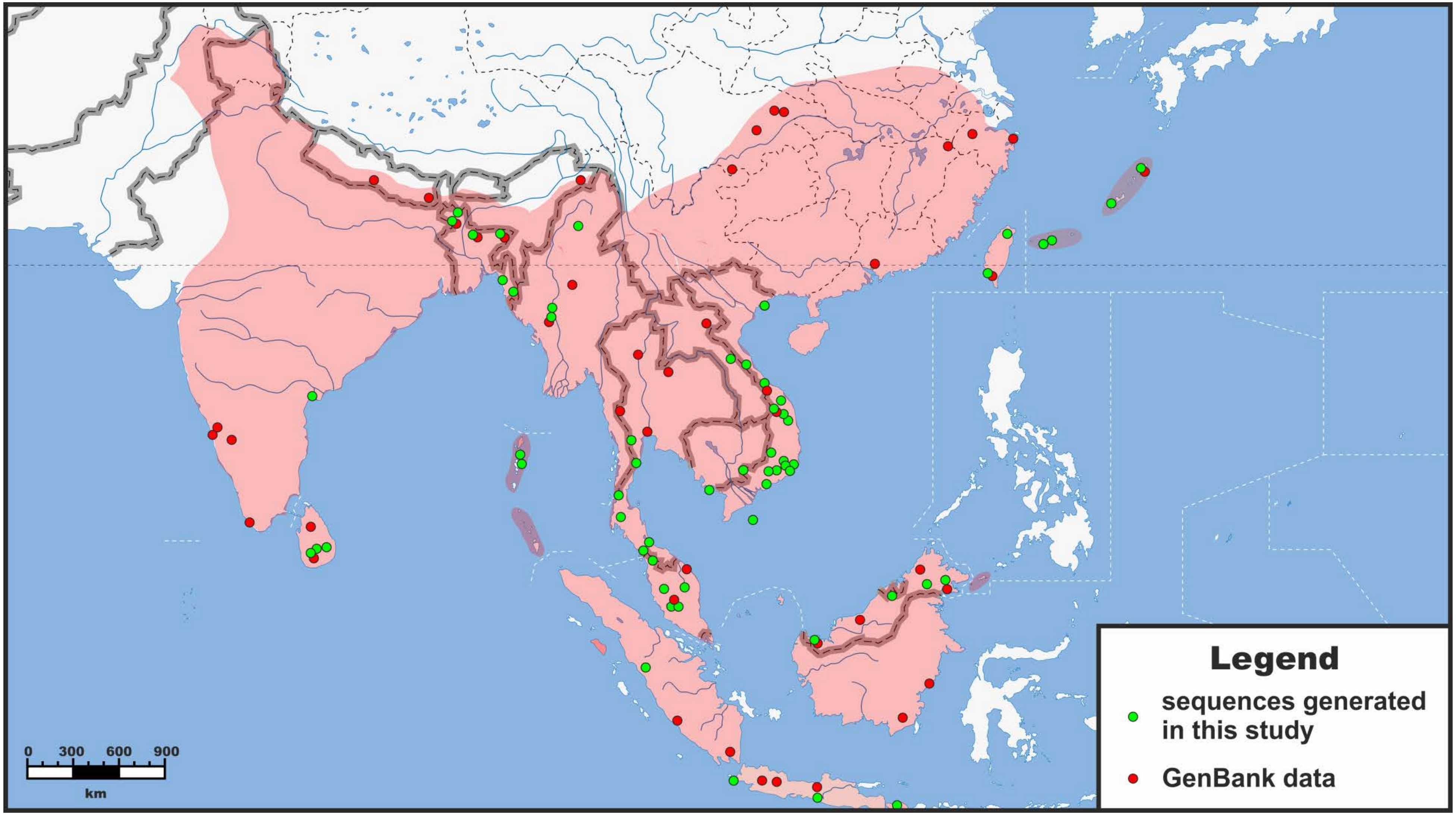
Legend
sequences generated in this study
GenBank data
0 300 600 900
km
