# Supplementary material for: A little frog leaps a long way: compounded colonizations of the Indian Subcontinent discovered in the tiny Oriental frog genus Microhyla (Amphibia: Microhylidae)
Source: PeerJ. 2020 Jul 3;8:e9411. doi: 10.7717/peerj.9411 (PMC7337035; doi:10.7717/peerj.9411)
Supplement: Supplemental Information 2 — BI genealogy of Microhyla and Glyphoglossus reconstructed from 2478 bp of mtDNA fragment. Values at nodes correspond to BI PP/ML BS, respectively; black and white circles correspond to well-supported (BI PP ≥ 0.95; ML BS ≥ 90) and moderately supported (0.95 > BI PP ≥ 0.90; 90 > ML BS ≥ 75) nodes, respectively; no circles indicate unsupported nodes. Color marking of species groups in Microhyla species complex corresponds to Figs. 4 and 5, but not to Fig. 2. Photos by Nikolay A. Poyarkov, Indraneil Das, Vladislav A. Gorin, Parinya Pawangkhanant, Luan Thanh Nguyen, and Evgeniya N. Solovyeva. [file peerj-08-9411-s002.pdf]

Supplementary Figure S2. Updated mtDNA-genealogy of the *Microhyla – Glyphoglossus* assemblage (collapsed tree).

BI genealogy of *Microhyla* and *Glyphoglossus* reconstructed from 2478 bp of mtDNA fragment. Values at nodes correspond to BI PP/ML BS, respectively; black and white circles correspond to well-supported (BI PP ≥ 0.95; ML BS ≥ 90) and moderately supported (0.95 > BI PP ≥ 0.90; 90 > ML BS ≥ 75) nodes, respectively; no circles indicate unsupported nodes. Color marking of species groups in *Microhyla* species complex corresponds to Figs. 4–5, but not to Fig. 2. Photos by Nikolay A. Poyarkov, Indraneil Das, Vladislav A. Gorin, Parinya Pawangkhanant, Luan Thanh Nguyen, and Evgeniya N. Solovyeva.

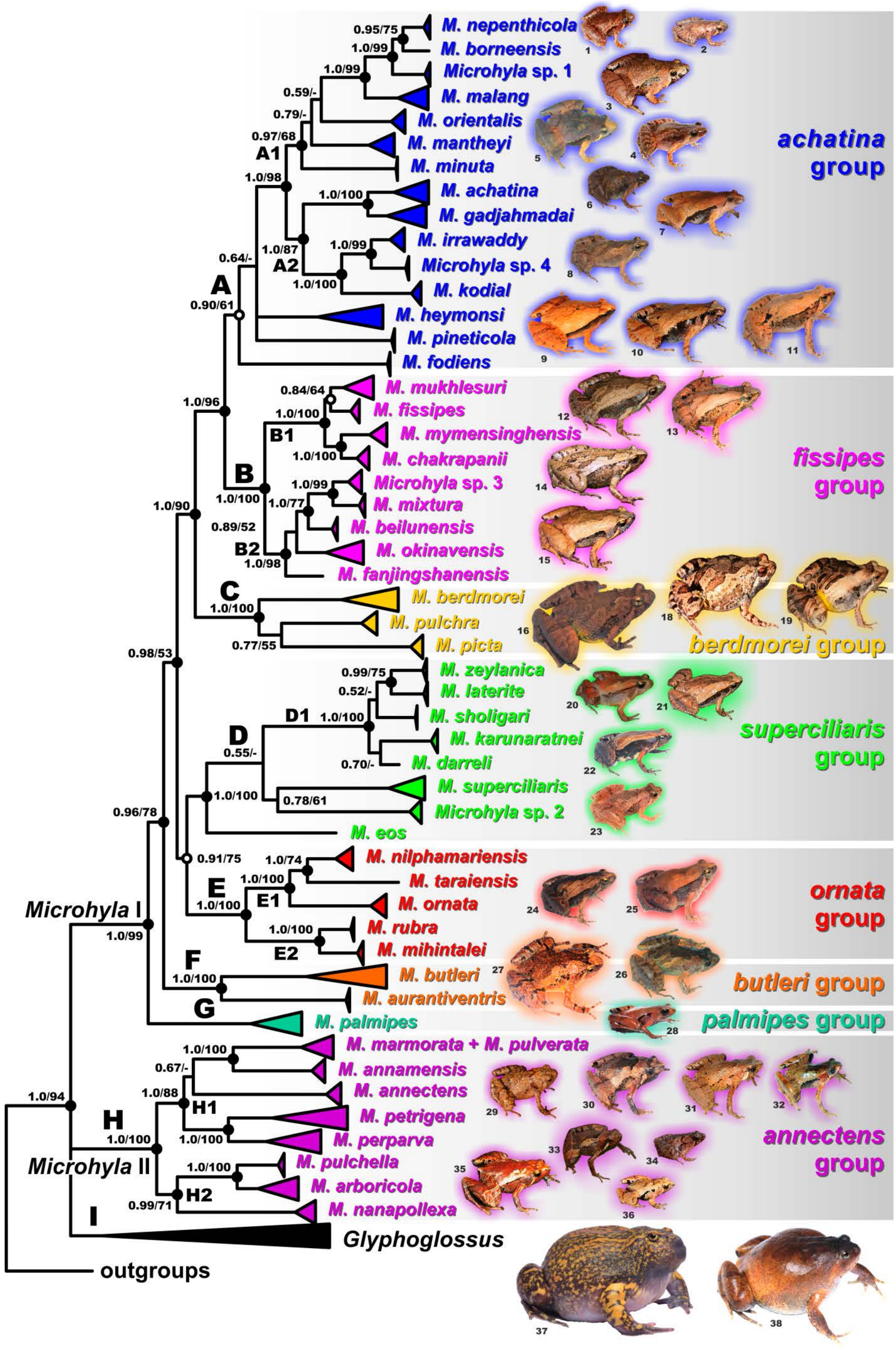

M. nepenthicola
M. borneensis
Microhyla sp. 1
M. malang
M. orientalis
M. mantheyi
M. minuta
M. achatina
M. gadjahmadai
M. irrawaddy
Microhyla sp. 4
M. kodial
M. heymonsi
M. pineticola
M. fodiens
achatina group
M. mukhlesuri
M. fissipes
M. mymensinghensis
M. chakrapanii
Microhyla sp. 3
M. mixtura
M. beilunensis
M. okinavensis
M. fanjingshanensis
fissipes group
M. berdmorei
M. pulchra
M. picta
berdmorei group
M. zeylanica
M. laterite
M. sholigari
M. karunaratnei
M. darreli
M. superciliaris
Microhyla sp. 2
M. eos
superciliaris group
M. nilphamariensis
M. taraiensis
M. ornata
M. rubra
M. mihintalei
ornata group
M. butleri
M. aurantiventris
butleri group
M. palmipes
palmipes group
M. marmorata + M. pulverata
M. annamensis
M. annectens
M. petrigena
M. perparva
M. pulchella
M. arboricola
M. nanapollexa
annectens group
Glyphoglossus
outgroups
Microhyla I
Microhyla II
A
A1
A2
B
B1
B2
C
D
D1
E
E1
E2
F
G
H
H1
H2
I
0.95/75
1.0/99
1.0/99
0.59/-
0.79/-
0.97/68
1.0/98
1.0/100
1.0/87
1.0/99
1.0/100
0.64/-
0.90/61
1.0/96
0.84/64
1.0/100
1.0/100
1.0/99
1.0/100
1.0/77
0.89/52
1.0/98
1.0/90
1.0/100
0.77/55
0.98/53
0.99/75
0.52/-
1.0/100
0.55/-
0.70/-
1.0/100
0.78/61
0.96/78
0.91/75
1.0/74
1.0/100
1.0/100
1.0/100
1.0/100
1.0/99
1.0/100
1.0/100
0.67/-
1.0/88
1.0/94
1.0/100
1.0/100
1.0/100
0.99/71
1
2
3
4
5
6
7
8
9
10
11
12
13
14
15
16
18
19
20
21
22
23
24
25
26
27
28
29
30
31
32
33
34
35
36
37
38
