# Supplementary material for: A little frog leaps a long way: compounded colonizations of the Indian Subcontinent discovered in the tiny Oriental frog genus Microhyla (Amphibia: Microhylidae)
Source: PeerJ. 2020 Jul 3;8:e9411. doi: 10.7717/peerj.9411 (PMC7337035; doi:10.7717/peerj.9411)
Supplement: Supplemental Information 3 — Circle sizes are proportional to the number of haplotypes, circle numbers correspond to sample numbers summarized in Supplementary Table S1, circle colors depict the recognized species groups, small black circles represent hypothetical median vectors, vertical bars on branches represent the number of mutational steps. [file peerj-08-9411-s003.pdf]

Supplementary Figure S3. Nuclear allele median-joining network showing relationships among phased nuclear BDNF gene haplotypes representing 49 *Microhyla* and *Glyphoglossus* species.

Circle sizes are proportional to the number of haplotypes, circle numbers correspond to sample numbers summarized in Supplementary Table S1, circle colors depict the recognized species groups, small black circles represent hypothetical median vectors, vertical bars on branches represent the number of mutational steps.

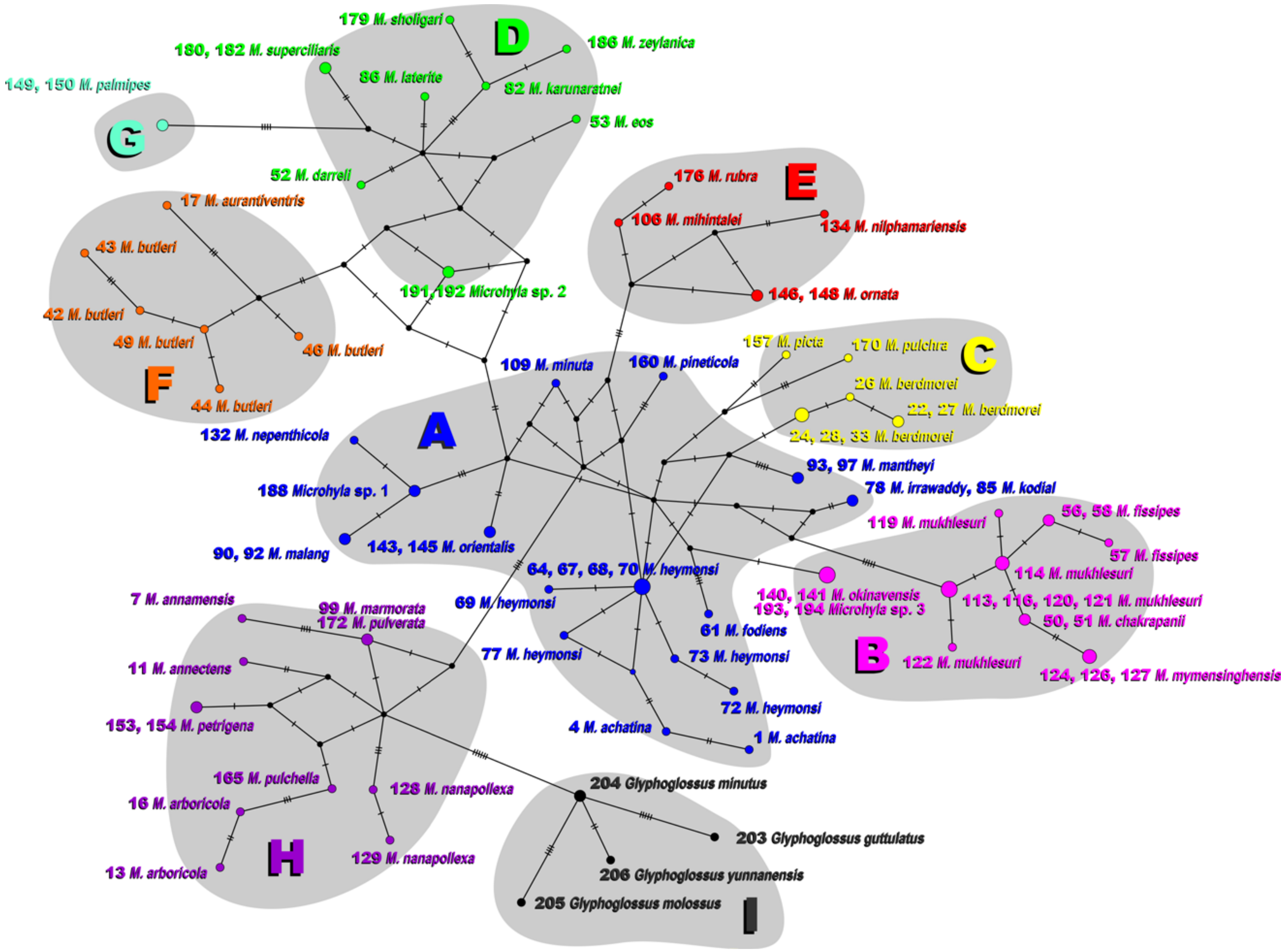

179 M. sholigari
D
186 M. zeylanica
180, 182 M. superciliaris
86 M. laterite
82 M. karunaratnei
149, 150 M. palmipes
G
53 M. eos
52 M. darreli
176 M. rubra
E
17 M. aurantiventris
106 M. mihintalei
134 M. nilphamariensis
43 M. butleri
191,192 Microhyla sp. 2
146, 148 M. ornata
42 M. butleri
49 M. butleri
46 M. butleri
157 M. picta
170 M. pulchra
C
109 M. minuta
160 M. pineticola
F
26 M. berdmorei
44 M. butleri
22, 27 M. berdmorei
132 M. nepenthicola
A
24, 28, 33 M. berdmorei
93, 97 M. mantheyi
188 Microhyla sp. 1
78 M. irrawaddy, 85 M. kodial
56, 58 M. fissipes
119 M. mukhlesuri
90, 92 M. malang
143, 145 M. orientalis
57 M. fissipes
64, 67, 68, 70 M. heymonsi
114 M. mukhlesuri
7 M. annamensis
140, 141 M. okinavensis
193, 194 Microhyla sp. 3
113, 116, 120, 121 M. mukhlesuri
99 M. marmorata
172 M. pulverata
69 M. heymonsi
50, 51 M. chakrapanii
61 M. fodiens
11 M. annectens
77 M. heymonsi
73 M. heymonsi
B
122 M. mukhlesuri
124, 126, 127 M. mymensinghensis
72 M. heymonsi
153, 154 M. petrigena
4 M. achatina
1 M. achatina
165 M. pulchella
128 M. nanapollexa
204 Glyphoglossus minutus
16 M. arboricola
203 Glyphoglossus guttulatus
H
206 Glyphoglossus yunnanensis
13 M. arboricola
129 M. nanapollexa
205 Glyphoglossus molossus
I
