# Supplementary material for: A little frog leaps a long way: compounded colonizations of the Indian Subcontinent discovered in the tiny Oriental frog genus Microhyla (Amphibia: Microhylidae)
Source: PeerJ. 2020 Jul 3;8:e9411. doi: 10.7717/peerj.9411 (PMC7337035; doi:10.7717/peerj.9411)
Supplement: Supplemental Information 4 — Node values correspond to node numbers, for estimated divergence times (in Ma) see Table S11. Red icons correspond to calibration points used in molecular dating analysis, for details see Table S4. Blue bars correspond to 95%-confidence intervals. [file peerj-08-9411-s004.pdf]

Supplementary Figure S4. Bayesian chronogram resulted from *BEAST analysis of the 3207 bp-long concatenated mtDNA + nuclear DNA dataset.

Node values correspond to node numbers, for estimated divergence times (in Ma) see Table S11. Red icons correspond to calibration points used in molecular dating analysis, for details see Table S4. Blue bars correspond to 95%-confidence intervals.

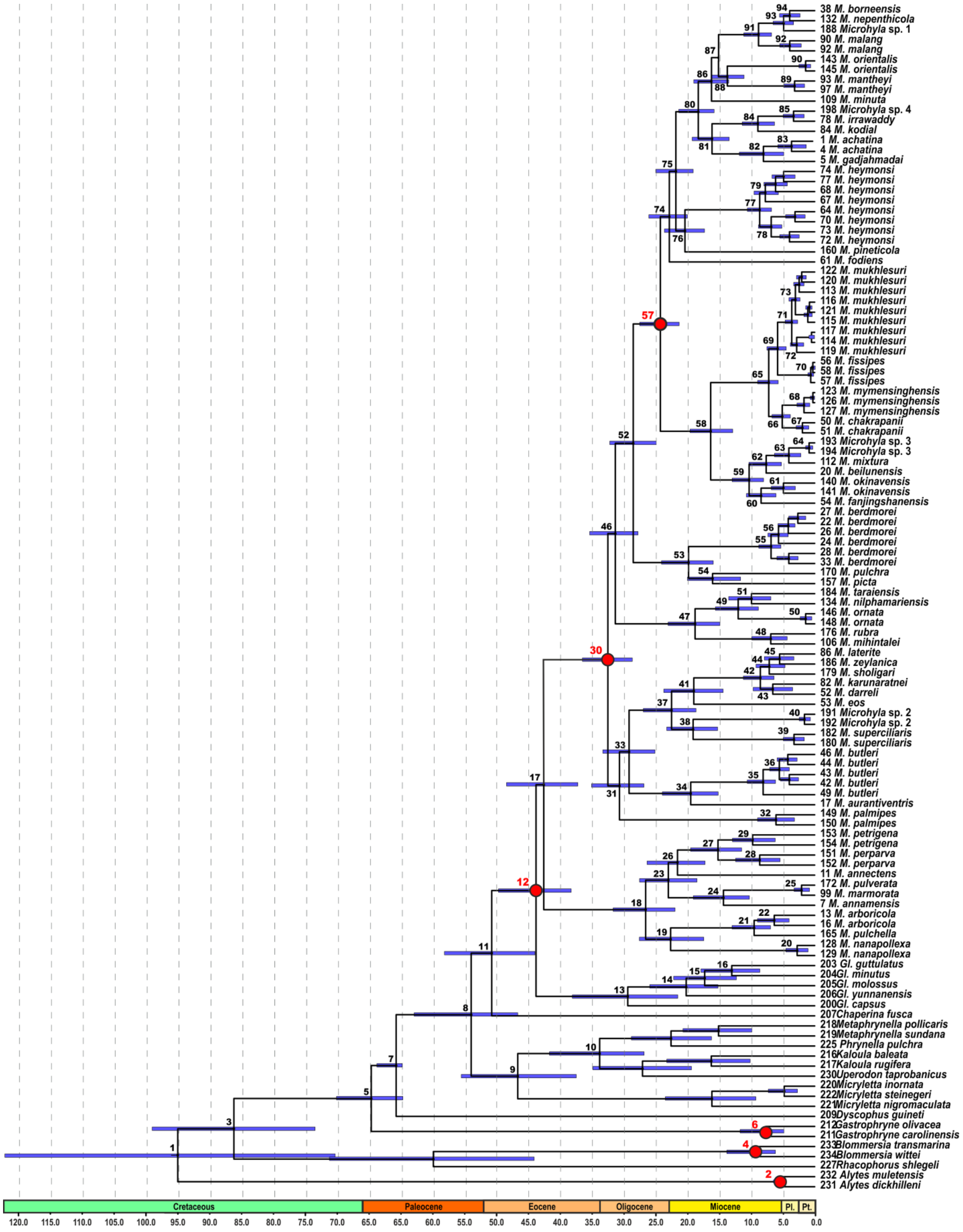
38 M. borneensis
132 M. nepenthicola
188 Microhyla sp. 1
90 M. malang
92 M. malang
143 M. orientalis
145 M. orientalis
93 M. mantheyi
97 M. mantheyi
109 M. minuta
198 Microhyla sp. 4
78 M. irrawaddy
84 M. kodial
1 M. achatina
4 M. achatina
5 M. gadjahmadai
74 M. heymonsi
77 M. heymonsi
68 M. heymonsi
67 M. heymonsi
64 M. heymonsi
70 M. heymonsi
73 M. heymonsi
72 M. heymonsi
160 M. pineticola
61 M. fodiens
122 M. mukhlesuri
120 M. mukhlesuri
113 M. mukhlesuri
116 M. mukhlesuri
121 M. mukhlesuri
115 M. mukhlesuri
117 M. mukhlesuri
114 M. mukhlesuri
119 M. mukhlesuri
56 M. fissipes
58 M. fissipes
57 M. fissipes
123 M. mymensinghensis
126 M. mymensinghensis
127 M. mymensinghensis
50 M. chakrapanii
51 M. chakrapanii
193 Microhyla sp. 3
194 Microhyla sp. 3
112 M. mixtura
20 M. beilunensis
140 M. okinavensis
141 M. okinavensis
54 M. fanjingshanensis
27 M. berdmorei
22 M. berdmorei
26 M. berdmorei
24 M. berdmorei
28 M. berdmorei
33 M. berdmorei
170 M. pulchra
157 M. picta
184 M. taraiensis
134 M. nilphamariensis
146 M. ornata
148 M. ornata
176 M. rubra
106 M. mihintalei
86 M. laterite
186 M. zeylanica
179 M. sholigari
82 M. karunaratnei
52 M. darreli
53 M. eos
191 Microhyla sp. 2
192 Microhyla sp. 2
182 M. superciliaris
180 M. superciliaris
46 M. butleri
44 M. butleri
43 M. butleri
42 M. butleri
49 M. butleri
17 M. aurantiventris
149 M. palmipes
150 M. palmipes
153 M. petrigena
154 M. petrigena
151 M. perparva
152 M. perparva
11 M. annectens
172 M. pulverata
99 M. marmorata
7 M. annamensis
13 M. arboricola
16 M. arboricola
165 M. pulchella
128 M. nanapollexa
129 M. nanapollexa
203 Gl. guttulatus
204Gl. minutus
205Gl. molossus
206Gl. yunnanensis
200Gl. capsus
207Chaperina fusca
218Metaphrynella pollicaris
219Metaphrynella sundana
225 Phrynella pulchra
216Kaloula baleata
217Kaloula rugifera
230Uperodon taprobanicus
220Micryletta inornata
222Micryletta steinegeri
221Micryletta nigromaculata
209Dyscophus guineti
212Gastrophryne olivacea
211Gastrophryne carolinensis
233Blommersia transmarina
234Blommersia wittei
227Rhacophorus shlegeli
232 Alytes muletensis
231 Alytes dickhilleni
Cretaceous
Paleocene
Eocene
Oligocene
Miocene
Pl.
Pt.
120.0 115.0 110.0 105.0 100.0 95.0 90.0 85.0 80.0 75.0 70.0 65.0 60.0 55.0 50.0 45.0 40.0 35.0 30.0 25.0 20.0 15.0 10.0 5.0 0.0
