# Supplementary material for: A little frog leaps a long way: compounded colonizations of the Indian Subcontinent discovered in the tiny Oriental frog genus Microhyla (Amphibia: Microhylidae)
Source: PeerJ. 2020 Jul 3;8:e9411. doi: 10.7717/peerj.9411 (PMC7337035; doi:10.7717/peerj.9411)
Supplement: Supplemental Information 5 — Asterisk (*) denotes sequences that were included in the alignment for timetree calibration. No exact locality information is available for specimens obtained via pet trade and published in earlier works. For references see Supplementary Information file 2. [file peerj-08-9411-s005.docx]

**Supplementary Table S1. Museum voucher information, geographic localities, and GenBank accession numbers of specimens and sequences used in this study.**

Asterisk (*) denotes sequences that were included in the alignment for timetree calibration. No exact locality information is available for specimens obtained via pet trade and published in earlier works. For references see Supplementary Information file 2.

| **No.** | **Species** | **Locality** | **Museum / Sample ID** | **12S rRNA** | **16S rRNA** | **BDNF** | **Reference** |
| --- | --- | --- | --- | --- | --- | --- | --- |
|  | **Ingroup** |  |  |  |  |  |  |
| **1** | *Microhyla achatina** | Indonesia, Java, Ungaran | MZB Amp 16402 | AB634598 | AB634656 | KM509299 | *Matsui et al., 2011* |
| **2** | *Microhyla achatina* | Indonesia, Java, Gede Pangrango | MDK 24 | AB634599 | AB634657 | – | *Matsui et al., 2011* |
| **3** | *Microhyla achatina* | Indonesia, Java, Ungaran | – | KM509162 | | – | *Peloso et al., 2016* |
| **4** | *Microhyla achatina** | Indonesia, Java, Ujung Kulong | ZMMU A5070 | MN534670 | MN534462, MN534563 | MN534402 | *this work* |
| **5** | *Microhyla achatina* | Indonesia, Java, Ujung Kulong | ZMMU A5054-1 | MN534671 | MN534463, MN534564 | – | *this work* |
| **6** | *Microhyla achatina* | Indonesia, Java, Ujung Kulong | ZMMU A5054-2 | MN534672 | MN534464, MN534565 | – | *this work* |
| **7** | *Microhyla annamensis** | Vietnam, Lam Dong, Bidoup - Nui Ba NP | ZMMU A5075-06 | MN534748 | MN534533, MN534639 | MN534443 | *this work* |
| **8** | *Microhyla annamensis* | Vietnam, Dak Lak, Chu Yang Sin NP | ZMMU A4899-99 | MN534749 | MN534534, MN534640 | – | *this work* |
| **9** | *Microhyla annectens* | Malaysia, Pahang, Cameron | KUHE 52438 | AB634601 | AB634659 | – | *Matsui et al., 2011* |
| **10** | *Microhyla annectens* | Malaysia, Selangor, Genting | KUHE 53373 | AB634600 | AB634658 | – | *Matsui et al., 2011* |
| **11** | *Microhyla annectens** | Malaysia, Selangor, Genting | ZMMU A6042-1 | MN534746 | MN534531, MN534637 | MN534442 | *this work* |
| **12** | *Microhyla annectens* | Malaysia, Selangor, Genting | ZMMU A6042-2 | MN534747 | MN534532, MN534638 | – | *this work* |
| **13** | *Microhyla arboricola** | Vietnam, Dak Lak, Lak, Chu Yang Sin NP | ZMMU A4845-60 | MN534759 | MN534543, MN534650 | MN534446 | *this work* |
| **14** | *Microhyla arboricola* | Vietnam, Dak Lak, Lak, Chu Yang Sin NP | ZMMU A5051 | MN534758 | MN534542, MN534649 | – | *this work* |
| **15** | *Microhyla arboricola* | Vietnam, Khanh Hoa, Hon Ba NR, Hon Ba mt. | ZMMU A5052-65 | MN534760 | MN534544, MN534651 | – | *this work* |
| **16** | *Microhyla arboricola** | Vietnam, Khanh Hoa, Hon Ba NR, Hon Ba mt. | ZMMU A5052-67 | MN534761 | MN534545, MN534652 | MN534447 | *this work* |
| **17** | *Microhyla aurantiventris** | Vietnam, Gia Lai, K'Bang, Tram Lap | ITBCZ 4360 | MN534727 | MH286427 | MN534431 | *Nguyen et al., 2019; this work* |
| **18** | *Microhyla aurantiventris* | Vietnam, Gia Lai, K'Bang, Tram Lap | ITBCZ 4361 | MN534728 | MH286426 | – | *Nguyen et al., 2019; this work* |
| **19** | *Microhyla beilunensis* | China, Zhejiang, Ningbo, Beilun, Chaiqiao | CIB BL003 | MH234522 | MH234536 | – | *Zhang et al., 2018* |
| **20** | *Microhyla beilunensis** | China, Sichuan | CIB 20070248 | AB634611 | AB634669 | – | *Matsui et al., 2011* |
| **21** | *Microhyla berdmorei* | Malaysia, Selangor, Gombak | KUHE 52034 | AB598314 | AB598338 | – | *Matsui, 2011* |
| **22** | *Microhyla berdmorei** | Indonesia, Sumatra, Bengkulu | MZB Amp 16413 | AB634602 | AB634660 | KC180094 | *Matsui et al., 2011* |
| **23** | *Microhyla berdmorei* | Indonesia, Kalimantan, Paramasan | MZB Amp 15270 | AB634603 | AB634661 | – | *Matsui et al., 2011* |
| **24** | *Microhyla berdmorei** | Malaysia, Terengganu, Besut | KUHE 52373 | AB634604 | AB634662 | – | *Matsui et al., 2011* |
| **25** | *Microhyla berdmorei* | Thailand, Phrae, Mae Yom | KUHE 21992 | AB634609 | AB634667 | – | *Matsui et al., 2011* |
| **26** | *Microhyla berdmorei** | Malaysia, Borneo, Sabah | RMBR 2153 | MN534706 | MN534498, MN534599 | KC180094 | *this work* |
| **27** | *Microhyla berdmorei** | Malaysia, Taman Negara NP | ZMMU NAP-06744 | MN534707 | MN534499, MN534600 | KC180094 | *this work* |
| **28** | *Microhyla berdmorei** | Bangladesh, Bandarban | IABHU-3862 | MN534709 | MN534501, MN534602 | KC180094 | *this work* |
| **29** | *Microhyla berdmorei* | Bangladesh, Sylhet | DFBGBAU Msp G-7 | MN534708 | MN534500, MN534601 | – | *this work* |
| **30** | *Microhyla berdmorei* | Thailand, Suratthani, Khao Sok NP | ZMMU NAP-04133 | MN534711 | MN534503, MN534604 | – | *this work* |
| **31** | *Microhyla berdmorei* | Vietnam, Tay Ninh, Lo Go, Xa Mat NP | ZMMU NAP-03595 | MN534710 | MN534502, MN534603 | – | *this work* |
| **32** | *Microhyla berdmorei* | Vietnam, Lam Dong, Bidoup - Nui Ba NP, Giang Ly | ZMMU A5073-21 | MN534714 | MN534505, MN534607 | – | *this work* |
| **33** | *Microhyla berdmorei** | Vietnam, Kon Tum, Kon Plong | ZPMSU 06364 | MN534712 | MN534504, MN534605 | KC180094 | *this work* |
| **34** | *Microhyla berdmorei* | Laos, Khammouan, Nakai-Nam Theun | ZISP FN-00237 | MN534713 | MN534606 | – | *this work* |
| **35** | *Microhyla bermodrei* | Bangladesh, Sylhet, Golapganj | DFBGBAU 418 | – | AB530540 | – | *Hasan et al., 2012* |
| **36** | *Microhyla bermodrei* | Bangladesh, Sylhet, Golapganj | DFBGBAU 419 | – | AB530542 | – | *Hasan et al., 2012* |
| **37** | *Microhyla bermodrei* | Malaysia, Gombak FSC | IABHU 21019 | – | AB530638 | – | *Hasan et al., 2014* |
| **38** | *Microhyla borneensis** | Malaysia, Sarawak, Kidi (Bidi) | UNIMAS FN 1874ZAC600 | – | MN534550, MN534657 | MN534394 | *this work* |
| **39** | *Microhyla butleri* | Vietnam, Thua Thien-Hue, A Luoi | KUHE 40591 | AB634606 | AB634664 | – | *Matsui et al., 2011* |
| **40** | *Microhyla butleri* | China, Taiwan, Tainan | KUHE 44203 | AB634607 | AB634665 | – | *Matsui et al., 2011* |
| **41** | *Microhyla butleri* | Malaysia, Kuala Lumpur | – | KT285802 | | – | *Yong et al., 2016* |
| **42** | *Microhyla butleri** | Myanmar, Kachin, Indawgyi, In Gyin Taung mt. | ZMMU A6036 | MK208937 | | MN534435 | *Poyarkov et al., 2019; this work* |
| **43** | *Microhyla butleri** | Malaysia, Tasik Pedu Lake, Kedah | ZMMU NAP-06827 | MN534734 | MN534521, MN534625 | MN534434 | *this work* |
| **44** | *Microhyla butleri** | Vietnam, Hai Phong, Cat Ba NP | ZMMU A6037 | MN534729 | MN534516, MN534620 | MN534432 | *this work* |
| **45** | *Microhyla butleri* | Vietnam, Hai Phong, Cat Ba NP | ZMMU A6038 | MN534731 | MN534518, MN534622 | – | *this work* |
| **46** | *Microhyla butleri** | Vietnam, Ba Ria, Vung Tau, Binh Chau, Phuoc Buu NP | ZMMU A4922-64 | MN534732 | MN534519, MN534623 | MN534433 | *this work* |
| **47** | *Microhyla butleri* | Vietnam, Lam Dong, Bao Loc, Loc Bao forestry | ZMMU NAP-02815 | MN534733 | MN534520, MN534624 | – | *this work* |
| **48** | *Microhyla butleri* | Vietnam, Quang Binh, Ke Go | ZMMU A5549-96 | MN534730 | MN534517, MN534621 | – | *this work* |
| **49** | *Microhyla butleri** | China, Taiwan, Kaohsiung, Yanchao, Zhongliao-shan mt. | ZMMU A5335-40 | MN534735 | MN534626 | MN534436 | *this work* |
| **50** | *Microhyla chakrapanii ** | India, Andaman Islands, Havelock | ZISP 13874 | MN534698 | MN534490, MN534591 | MN534422 | *this work* |
| **51** | *Microhyla chakrapanii ** | India, Andaman Islands, Rangat | ZISP 13875 | MN534697 | MN534489, MN534590 | MN534421 | *this work* |
| **52** | *Microhyla darreli** | India, Kerala, Thiruvanan Thapuram, Karamana | ZSI/WGRC/V/A/962 | – | MH807390 | MH807429 | *Garg et al., 2019* |
| **53** | *Microhyla eos** | India, Arunachal Pradesh, Changlang, Namdapha NP | ZSIC 14312 | – | MN160599 | MN167548 | *Biju et al., 2019* |
| **54** | *Microhyla fanjingshanensis** | China, Guizhou | – | MF538787 | | – | *Zhao et al., 2018* |
| **55** | *Microhyla fissipes* | China, Sichuan | KUHE 27705 | LC465675 | |  | *Tominaga et al., 2019* |
| **56** | *Microhyla fissipes** | China, Anhui, Huang-shan mt. | KUHE 32943 | AB201174 | AB201185 | – | *Matsui et al., 2005* |
| **57** | *Microhyla fissipes** | China, Taiwan, Kaohsiung, Zhongliao-shan mt. | ZMMU A5333 | MN534695 | MN534487, MN534588 | MN534419 | *this work* |
| **58** | *Microhyla fissipes** | China, Zheijang, Hangzhou, Da Tong Lu, Qi-shan NP | ZMMU NAP-6603 | MN534696 | MN534488, MN534589 | MN534420 | *this work* |
| **59** | *Microhyla fodiens* | Myanmar, Magway, Pakoku | CAS 215851 | KM509166 | | – | *Peloso et al., 2016* |
| **60** | *Microhyla fodiens* | Myanmar, Magway, Kan Pauk | ZMMU A5960 | MK208926 | | – | *this work* |
| **61** | *Microhyla fodiens** | Myanmar, Magway, Kan Pauk | ZMMU A5961 | MK208927 | | MN534401 | *this work* |
| **62** | *Microhyla gadjahmadai** | Indonesia, Sumatra, Lampung | MZB Amp 15291 | AB634622 | AB634680 | – | *Matsui et al., 2011* |
| **63** | *Microhyla gadjahmadai* | Indonesia, Sumatra, Bengkulu | MZB Amp 16328 | AB634623 | AB634681 | – | *Matsui et al., 2011* |
| **64** | *Microhyla heymonsi** | Thailand, Ranong | KUHE 23856 | AB598312 | AB598336 | EF396020 | *Matsui, 2011* |
| **65** | *Microhyla heymonsi* | Thailand, Kanchanaburi | KUHE UN (K1845) | AB201179 | AB201190 | – | *Matsui et al., 2005* |
| **66** | *Microhyla heymonsi* | China | – | NC006406 | | – | *Zhang et al., 2005* |
| **67** | *Microhyla heymonsi** | Myanmar, Kachin, Indawgyi, In Gyin Taung mt. | ZMMU NAP-08277 | MK208932 | | MN534404 | *this work* |
| **68** | *Microhyla heymonsi** | China, Taiwan, Pingtong, Yongchin, Qi Kong | ZMMU A4975 | MN534679 | MN534471, MN534572 | MN534407 | *this work* |
| **69** | *Microhyla heymonsi* | China, Taiwan, Kaohsiung, Yanchao, Zhongliao-shan mt. | ZMMU A5334-45 | MN534682 | MN534474, MN534575 | – | *this work* |
| **70** | *Microhyla heymonsi** | Vietnam, Kien Giang, Phu Quoc island, Phu Quoc NP | ZMMU NAP-03780 | MN534678 | MN534470, MN534571 | MN534406 | *this work* |
| **71** | *Microhyla heymonsi* | Malaysia, Taman Negara NP | ZMMU NAP-06740 | MN534684 | MN534476, MN534577 | – | *this work* |
| **72** | *Microhyla heymonsi** | Malaysia, Taman Negara NP | ZMMU NAP-06741 | MN534685 | MN534477, MN534578 | MN534409 | *this work* |
| **73** | *Microhyla heymonsi** | Indonesia, Sumatra, West Sumatra, Bukittinggi | ZMMU NAP-06887 | MN534686 | MN534478, MN534579 | MN534410 | *this work* |
| **74** | *Microhyla heymonsi** | Thailand, Prachuap Khiri Khan | ZMMU A6045 | MN534677 | MN534469, MN534570 | MN534405 | *this work* |
| **75** | *Microhyla heymonsi* | Vietnam, Hai Phong, Cat Ba NP | ZMMU A6044 | MN534681 | MN534473, MN534574 | – | *this work* |
| **76** | *Microhyla heymonsi* | Laos, Khammouan, Nakai-Nam Theun | ZISP FN-00101 | MN534680 | MN534472, MN534573 | – | *this work* |
| **77** | *Microhyla heymonsi** | Vietnam, Kon Tum, Dak Glei, Ngoc Linh mt. | ZPMSU 04424 | MN534683 | MN534475, MN534576 | MN534408 | *this work* |
| **78** | *Microhyla irrawaddy** | Myanmar, Magway, Pakkoku | ZMMU A5966 | MK208928 | | MN534403 | *this work* |
| **79** | *Microhyla irrawaddy* | Myanmar, Magway, Pakkoku | ZMMU A5967 | MK208929 | | – | *this work* |
| **80** | *Microhyla irrawaddy* | Myanmar, Magway, Kan Pauk | ZMMU A5975 | MK208930 | | – | *this work* |
| **81** | *Microhyla irrawaddy* | Myanmar, Magway, Kan Pauk | ZMMU A5976 | MK208931 | | – | *this work* |
| **82** | *Microhyla karunaratnei** | Sri Lanka, Sinharaja FR | released | MN534738 | MN534524, MN534629 | MN534438 | *this work* |
| **83** | *Microhyla karunaratnei* | Sri Lanka, Sinharaja FR | released | MN534739 | MN534525, MN534630 | – | *this work* |
| **84** | *Microhyla kodial* | India, Karnataka, Mangaluru | – | – | MF919453 | – | *Vineeth et al., 2018* |
| **85** | *Microhyla kodial** | India, Karnataka, Mangaluru | – | – | MF919454 | MH807431 | *Vineeth et al., 2018* |
| **86** | *Microhyla laterite** | India, Karnataka, Udupi, Manipal | BNHS 5965 | KT600670 | KT600663 | MH807432 | *Seshadri et al., 2016* |
| **87** | *Microhyla laterite* | India, Karnataka, Udupi, Manipal | BNHS 5967 | KT600671 | KT600664 | – | *Seshadri et al., 2016* |
| **88** | *Microhyla malang* | Malaysia, Sarawak, Serapi | KUHE 53018 | AB598295 | AB598319 | – | *Matsui et al., 2011* |
| **89** | *Microhyla malang* | Malaysia, Sabah, Tawau | BORNEENSIS 9211 | AB598301 | AB598325 | – | *Matsui et al., 2011* |
| **90** | *Microhyla malang** | Indonesia, Kalimantan, Balikpapan | MZB Amp 16364 | AB634619 | AB634677 | – | *Matsui et al., 2011* |
| **91** | *Microhyla malang* | Malaysia, Sarawak, Kubah NP | released | MN534661 | MN534453, MN534554 | – | *this work* |
| **92** | *Microhyla malang** | Malaysia, Sarawak, Kubah NP | ZMMU A6043 | MN534662 | MN534454, MN534555 | MN534396 | *this work* |
| **93** | *Microhyla mantheyi** | Malaysia, Pahang, Temerloh | KUHE 52556 | AB598310 | AB598334 | KM509300 | *Matsui, 2011* |
| **94** | *Microhyla mantheyi* | Malaysia, Selangor, Gombak | KUHE 15726 | AB598309 | AB598333 | – | *Matsui, 2011* |
| **95** | *Microhyla mantheyi* | Malaysia, Selangor, Gombak | KUHE 15726 | KM509163 | | – | *Peloso et al., 2016* |
| **96** | *Microhyla mantheyi* | Malaysia, Taman Negara NP | ZMMU NAP-6745 | MN534665 | MN534457, MN534558 | – | *this work* |
| **97** | *Microhyla mantheyi** | Malaysia, Taman Negara NP | ZMMU NAP-6746 | MN534666 | MN534458, MN534559 | MN534398 | *this work* |
| **98** | *Microhyla marmorata* | Laos, Houapan, Xamneua | KUHE 32455 | AB634610 | AB634668 | AB611951 | *Matsui et al., 2011* |
| **99** | *Microhyla marmorata** | Vietnam, Kon Tum, Kon Plong | ZPMSU 04854 | MN534750 | MN534535, MN534641 | MN534445 | *this work* |
| **100** | *Microhyla marmorata* | Vietnam, Kon Tum, Kon Plong, Hieu | ZPMSU 00644 | MN534751 | MN534536, MN534642 | – | *this work* |
| **101** | *Microhyla marmorata* | Vietnam, Quang Nam | ZMMU NAP-04195 | MN534752 | MN534537, MN534643 | – | *this work* |
| **102** | *Microhyla marmorata* | Vietnam, Kon Tum, Kon Plong, Mang Canh | ZPMSU 08824 | MN534753 | MN534538, MN534644 | – | *this work* |
| **103** | *Microhyla marmorata* | Vietnam, Quang Tri | ZMMU A5072 | MN534754 | MN534645 | – | *this work* |
| **104** | *Microhyla mihintalei* | Sri Lanka, Rathambaldama | released | MN534724 | MN534513, MN534617 | – | *this work* |
| **105** | *Microhyla mihintalei* | Sri Lanka, Rathambaldama | released | MN534725 | MN534514, MN534618 | – | *this work* |
| **106** | *Microhyla mihintalei** | Sri Lanka, Rathambaldama | released | MN534726 | MN534515, MN534619 | MN534430 | *this work* |
| **107** | *Microhyla minuta* | Vietnam, Dong Nai, Cat Tien NP | ZMMU A6025 | MN534669 | MN534461, MN534562 | – | *this work* |
| **108** | *Microhyla minuta* | Vietnam, Dong Nai, Cat Tien NP | ZMMU A5048-91 | MN534667 | MN534459, MN534560 | – | *this work* |
| **109** | *Microhyla minuta** | Vietnam, Dong Nai, Cat Tien NP | ZMMU A5048-96 | MN534668 | MN534460, MN534561 | MN534400 | *this work* |
| **110** | *Microhyla mixtura* | China, Sichuan | CIB 2013051806 | LC465668 | |  | *Tominaga et al., 2019* |
| **111** | *Microhyla mixtura* | China, Sichuan | CIB 2013051807 | LC465669 | |  | *Tominaga et al., 2019* |
| **112** | *Microhyla mixtura** | China, Sichuan, Wanyuan, Hua’e-shan mt. | CIB 20170526001 | MH234529 | MH234540 | – | *Zhang et al., 2018* |
| **113** | *Microhyla mukhlesuri** | Thailand, Bangkok | KUHE 22064 | AB634608 | AB634666 | MH807433 | *Matsui et al., 2011* |
| **114** | *Microhyla mukhlesuri** | Vietnam, Ba Ria-Vung Tau, Con Dao NP, Con Son | ZMMU A4633 | MN534688 | MN534480, MN534581 | MN534412 | *this work* |
| **115** | *Microhyla mukhlesuri** | Vietnam, Dak Lak, Yok Don NP | ZMMU A4686-15 | MN534690 | MN534482, MN534583 | MN534414 | *this work* |
| **116** | *Microhyla mukhlesuri** | Thailand, Suratthani, Khao Sok NP | ZMMU NAP-04108 | MN534691 | MN534483, MN534584 | MN534415 | *this work* |
| **117** | *Microhyla mukhlesuri** | Thailand, Satun, Thale Ban NP | ZMMU NAP-04121 | MN534687 | MN534479, MN534580 | MN534411 | *this work* |
| **118** | *Microhyla mukhlesuri* | Laos, Khammouan, Nakai-Nam Theun | ZISP FN-00236 | MN534693 | MN534485, MN534586 | – | *this work* |
| **119** | *Microhyla mukhlesuri** | Vietnam, Quang Binh | ZMMU A5550 | MN534689 | MN534481, MN534582 | MN534413 | *this work* |
| **120** | *Microhyla mukhlesuri** | Bangladesh, Chittagong | IABHU-3959 | MN534692 | MN534484, MN534585 | MN534416 | *this work* |
| **121** | *Microhyla mukhlesuri** | Malaysia, Tasik Pedu Lake, Kedah | ZMMU NAP-6829 | MN534694 | MN534486, MN534587 | MN534417 | *this work* |
| **122** | *Microhyla mukhlesuri** | Myanmar, Kachin, Indawgyi, In Gyin Taung mt. | ZMMU NAP-8311 | MK208934 | | MN534418 | *this work* |
| **123** | *Microhyla mukhlesuri* | Myanmar, Magway, Pakkoku | ZMMU A6041 | MK208933 | | – | *this work* |
| **124** | *Microhyla mymensinghensis** | Bangladesh, Mymensingh, Char Nilokhia | IABHU F5012 BdMsp 77 | – | AB530534 | MH807434 | *Hasan et al., 2012* |
| **125** | *Microhyla mymensinghensis* | Bangladesh, Mymensingh, Char Nilokhia | IABHU F5012 BdMsp 78 | – | AB530535 | – | *Hasan et al., 2012* |
| **126** | *Microhyla mymensinghensis** | Bangladesh, Mymensingh, BAUC campus | DFBGBAU Msp 306 | – | AB530536 | – | *Hasan et al., 2012* |
| **127** | *Microhyla mymensinghensis** | Bangladesh, Mymensingh | IABHU-4129 | MN534699 | MN534491, MN534592 | MN534423 | *this work* |
| **128** | *Microhyla nanapollexa** | Vietnam, Quang Nam, Tra My, Ngoc Linh mt. | AMNH 163686 / AMCC 106460 | KM509164 | | KM509301 | *Peloso et al., 2016* |
| **129** | *Microhyla nanapollexa** | Vietnam, Kon Tum, Kon Plong | ZMMU A5635 | MN534757 | MN534541, MN534648 | MN534444 | *this work* |
| **130** | *Microhyla nepenthicola* | Malaysia, Borneo, Sarawak, Serapi | KUHE 53165 | AB598305 | AB598329 | – | *Matsui, 2011* |
| **131** | *Microhyla nepenthicola* | Malaysia, Borneo, Sarawak, Serapi | KUHE 53938 | AB634605 | AB634663 | – | *Matsui et al., 2011* |
| **132** | *Microhyla nepenthicola** | Malaysia, Borneo, Sarawak, Kubah NP | ZMMU A6028-1 | MN534658 | MN534450, MN534551 | MN534393 | *this work* |
| **133** | *Microhyla nepenthicola* | Malaysia, Borneo, Sarawak, Kubah NP | ZMMU A6028-2 | MN534659 | MN534451, MN534552 | – | *this work* |
| **134** | *Microhyla nilphamariensis** | Bangladesh, Dinajpur, Parbatipur | DB-Hi-FROG 12005 | AB201176 | AB201187 | MH807435 | *Matsui et al., 2005* |
| **135** | *Microhyla nilphamariensis* | Bangladesh, Dinajpur, Parbatipur | IABHU 22135 | – | AB530537 | – | *Hasan et al., 2012* |
| **136** | *Microhyla nilphamariensis* | Bangladesh, Dinajpur, Parbatipur | IABHU 22136 | – | AB530538 | – | *Hasan et al., 2012* |
| **137** | *Microhyla nilphamariensis* | Nepal, Narayani, Jhuwani, Chitwan | JRK201522 | – | KY655947 | – | *Khatiwada et al., 2017* |
| **138** | *Microhyla nilphamariensis* | Bangladesh, Nilphamari | IABHU-4212 | MN534721 | MN534614 | – | *this work* |
| **139** | *Microhyla nilphamariensis* | Bangladesh, Dinajpur | IABHU 22137 | MN534720 | MN534613 | – | *this work* |
| **140** | *Microhyla okinavensis** | Japan, Amami island | KUHE 12840 | AB201173 | AB201184 | AB611959 | *Matsui et al., 2005* |
| **141** | *Microhyla okinavensis** | Japan, Okinawa island, Yomitan son, Kina | ZMMU A6027-1 | MN534704 | MN534496, MN534597 | MN534426 | *this work* |
| **142** | *Microhyla okinavensis* | Japan, Okinawa island, Yomitan son, Kina | ZMMU A6027-2 | MN534705 | MN534497, MN534598 | – | *this work* |
| **143** | *Microhyla orientalis** | Indonesia, Bali, Batu Karu | MZB Amp 16259 | AB634621 | AB634679 | – | *Matsui et al., 2011* |
| **144** | *Microhyla orientalis* | Indonesia, Java, Yogyakarta | ZMMU A5067-1 | MN534663 | MN534455, MN534556 | – | *this work* |
| **145** | *Microhyla orientalis ** | Indonesia, Java, Yogyakarta | ZMMU A5067-2 | MN534664 | MN534456, MN534557 | MN534397 | *this work* |
| **146** | *Microhyla ornata** | India, Karnataka | ZSIK-A9119 | AB201177 | AB201188 | MH807436 | *Matsui et al., 2005* |
| **147** | *Microhyla ornata* | Sri Lanka, Rathambaldama | released | MN534722 | MN534511, MN534615 | – | *this work* |
| **148** | *Microhyla ornata** | Sri Lanka, Rathambaldama | released | MN534723 | MN534512, MN534616 | MN534428 | *this work* |
| **149** | *Microhyla palmipes** | Indonesia, Bali, Bedegul | MZB Amp 16255 | AB634612 | AB634670 | MN539668 | *Matsui et al., 2011* |
| **150** | *Microhyla palmipes** | Indonesia, Sumatra, Bengkulu | MZB Amp 16323 | AB634613 | AB634671 | MN539669 | *Matsui et al., 2011* |
| **151** | *Microhyla perparva** | Indonesia, Kalimantan, Balikpapan | KUHE UN | AB634614 | AB634672 | – | *Matsui et al., 2011* |
| **152** | *Microhyla perparva** | Malaysia, Sarawak, Mulu | KUHE 53675 | AB634615 | AB634673 | – | *Matsui et al., 2011* |
| **153** | *Microhyla petrigena** | Malaysia, Sabah, Maliau Basin | BORN 22412 | AB634616 | AB634674 | KM509302 | *Matsui et al., 2011* |
| **154** | *Microhyla petrigena** | Malaysia, Sarawak, Bukit Kana | KUHE 53743 | AB634617 | AB634675 | – | *Matsui et al., 2011* |
| **155** | *Microhyla petrigena* | Malaysia, Sarawak, Bukit Kana | KUHE 53743 | KM509165 | | – | *Peloso et al., 2016* |
| **156** | *Microhyla picta* | Vietnam, Ba Ria-Vung Tau, Binh Chau, Phuoc Buu NR | ZMMU A4918-43 | MN534718 | MN534509, MN534611 | – | *this work* |
| **157** | *Microhyla picta** | Vietnam, Ba Ria-Vung Tau, Binh Chau, Phuoc Buu NR | ZMMU A4918-45 | MN534719 | MN534510, MN534612 | MN534427 | *this work* |
| **158** | *Microhyla pineticola* | Vietnam, Lam Dong, Bidoup - Nui Ba NP, Bidoup Mt. | ZMMU A5043 | MN534676 | MN534468, MN534569 | – | *this work* |
| **159** | *Microhyla pineticola* | Vietnam, Lam Dong, Bidoup - Nui Ba NP, Giang Ly | ZMMU A4331-16 | MN534674 | MN534466, MN534567 | – | *this work* |
| **160** | *Microhyla pineticola** | Vietnam, Lam Dong, Bidoup - Nui Ba NP, Giang Ly | ZMMU A5080-50 | MN534673 | MN534465, MN534566 | MN534399 | *this work* |
| **161** | *Microhyla pineticola* | Vietnam, Dak Lak, Lak, Chu Yang Sin NP | ZMMU A6029 | MN534675 | MN534467, MN534568 | – | *this work* |
| **162** | *Microhyla pulchella* | Vietnam, Lam Dong, Bidoup - Nui Ba NP, Giang Ly | ZMMU A5068-24 | MN534762 | MN534546, MN534653 | – | *this work* |
| **163** | *Microhyla pulchella* | Vietnam, Lam Dong, Bidoup - Nui Ba NP, Hon Giao | ZMMU A5079-84 | MN534764 | MN534548, MN534655 | – | *this work* |
| **164** | *Microhyla pulchella* | Vietnam, Dak Lak, Lak, Chu Yang Sin NP | ZMMU A6040 | MN534763 | MN534547, MN534654 | – | *this work* |
| **165** | *Microhyla pulchella** | Vietnam, Lam Dong, Bidoup - Nui Ba NP, Ca Hoi | ZMMU A5045 | MN534765 | MN534549, MN534656 | MN534448 | *this work* |
| **166** | *Microhyla pulchra* | Thailand, Kanchaburi, Pilok | KUHE 22113 | AB634618 | AB634676 | – | *Matsui et al., 2011* |
| **167** | *Microhyla pulchra* | Thailand, Loei, Phu Luan | KUHE 35119 | AB201180 | AB201191 | – | *Matsui et al., 2005* |
| **168** | *Microhyla pulchra* | China, Guangdong, Dongguan, Yingping mt. | – | NC 024547 | | – | *Wu et al., 2016* |
| **169** | *Microhyla pulchra* | Vietnam, Dak Lak, Yok Don NP | ZMMU A4682-10 | MN534717 | MN534506, MN534610 | – | *this work* |
| **170** | *Microhyla pulchra** | Laos, Khammouan, Nakai-Nam Theun | ZISP FN-00154 | MN534716 | MN534507, MN534609 | EF396021 | *this work* |
| **171** | *Microhyla pulchra* | Vietnam, Quang Binh, Ke Go | ZMMU A5548-79 | MN534715 | MN534508, MN534608 | – | *this work* |
| **172** | *Microhyla pulverata** | Vietnam, Kon Chu Rang | ZMMU A6026-1 | MN534755 | MN534539, MN534646 | MN534445 | *this work* |
| **173** | *Microhyla pulverata* | Vietnam, Kon Chu Rang | ZMMU A6026-2 | MN534756 | MN534540, MN534647 | – | *this work* |
| **174** | *Microhyla rubra* | India, Karnataka | released | AB201181 | AB201192 | MH807437 | *Matsui et al., 2005* |
| **175** | *Microhyla rubra* | India, Andhra Pradesh, Bapatla | ZMMU A5006-18 | MK208935 | | – | *Poyarkov et al., 2019; this work* |
| **176** | *Microhyla rubra** | India, Andhra Pradesh, Bapatla | ZMMU A5006-19 | MK208936 | | MN534429 | *Poyarkov et al., 2019; this work* |
| **177** | *Microhyla sholigari* | India, Karnataka, Udupi District, Manipal | ATREE MISH 1 | KT600667 | KT600674 | – | *Seshadri et al., 2016* |
| **178** | *Microhyla sholigari* | India, Karnataka, Udupi District, Manipal | ATREE MISH 2 | KT600668 | KT600675 | – | *Seshadri et al., 2016* |
| **179** | *Microhyla sholigari** | India, Karnataka, Udupi District, Manipal | ATREE MISH 3 | KT600669 | KT600676 | MH807438 | *Seshadri et al., 2016* |
| **180** | *Microhyla superciliaris** | Malaysia, Pahang, Temerloh | KUHE 52558 | AB634624 | AB634682 | – | *Matsui et al., 2011* |
| **181** | *Microhyla superciliaris* | Malaysia, Negeri Sembilan, Kenaboi | KUHE 53371 | AB634625 | AB634683 | – | *Matsui et al., 2011* |
| **182** | *Microhyla superciliaris** | Thailand, Songkhla | ZMMU A6024-1 | MN534744 | MN534530, MN534635 | MN534441 | *this work* |
| **183** | *Microhyla superciliaris* | Thailand, Songkhla | ZMMU A6024-2 | MN534745 | MN534636 | – | *this work* |
| **184** | *Microhyla taraiensis** | Nepal, Mechi, Jamun Khadi, Jhapa | – | MF496241 | | – | *Khatiwada et al., 2018* |
| **185** | *Microhyla zeylanica* | Sri Lanka, Central Province, Nuwara Eliya | released | MN534736 | MN534522, MN534627 | – | *this work* |
| **186** | *Microhyla zeylanica** | Sri Lanka, Central Province, Nuwara Eliya | released | MN534737 | MN534523, MN534628 | MN534437 | *this work* |
| **187** | *Microhyla* sp. 1 | Malaysia, Borneo, Sabah, Crocker | BORN 8480 | AB634620 | AB634678 | – | *Matsui et al., 2011* |
| **188** | *Microhyla* sp. 1* | Malaysia, Borneo, Sabah, Danum Valley | RMBR 2171 | MN534660 | MN534452, MN534553 | MN534395 | *this work* |
| **189** | *Microhyla* sp. 2 | Thailand, Suratthani, Khao Sok NP | ZMMU A6032 | MN534740 | MN534526, MN534631 | – | *this work* |
| **190** | *Microhyla* sp. 2 | Thailand, Suratthani, Khao Sok NP | ZMMU A6033 | MN534741 | MN534527, MN534632 | – | *this work* |
| **191** | *Microhyla* sp. 2* | Thailand, Suratthani, Khao Sok NP | ZMMU A6034 | MN534742 | MN534528, MN534633 | MN534439 | *this work* |
| **192** | *Microhyla* sp. 2* | Thailand, Phetchaburi, Kaeng Krachan | ZMMU A6035 | MN534743 | MN534529, MN534634 | MN534440 | *this work* |
| **193** | *Microhyla* sp. 3* | Japan, Okinawa, Ishigaki Isl. | released | MN534700 | MN534492, MN534593 | MN534424 | *this work* |
| **194** | *Microhyla* sp. 3* | Japan, Okinawa, Iriomote Isl. | released | MN534702 | MN534494, MN534595 | MN534425 | *this work* |
| **195** | *Microhyla* sp. 3 | Japan, Okinawa, Iriomote Isl. | ZMMU NAP-06340 | MN534703 | MN534495, MN534596 | – | *this work* |
| **196** | *Microhyla* sp. 3 | Japan, Okinawa, Ishigaki Isl. | ZMMU NAP-06341 | MN534701 | MN534493, MN534594 | – | *this work* |
| **197** | *Microhyla* sp. 3 | Japan, Okinawa, Ishigaki Isl. | IABHU5263 | – | AB303950 | – | *Igawa et al., 2008* |
| **198** | *Microhyla* sp. 4* | Myanmar, Sagaing | USNM 523975 | – | MG935884 | – | *Mulcahy et al., 2018* |
| **199** | *Microhyla* sp. 4 | Myanmar, Sagaing | USNM 537450 | – | MG935885 | – | *Mulcahy et al., 2018* |
| **200** | *Glyphoglossus capsus** | Malaysia, Sarawak, Padawan, Gunung Penrissen mt. | UNIMAS MYS:9389 | – | KJ488544 | – | *Das et al., 2014* |
| **201** | *Glyphoglossus capsus* | Malaysia, Sarawak, Padawan, Gunung Penrissen mt. | UNIMAS MYS:P0610 | – | KJ488545 | – | *Das et al., 2014* |
| **202** | *Glyphoglossus guttulatus* | Vietnam, Gia Lai, Ankhe | FMNH 252957 | – | KC822483 | – | *Blackburn et al., 2013* |
| **203** | *Glyphoglossus guttulatus** | Thailand, Kanchanaburi, Pilok | KUHE 35163 | AB634627 | AB634685 | AB611864 | *Matsui et al., 2011* |
| **204** | *Glyphoglossus minutus** | Malaysia, Pahang, Temerloh | KUHE 52463 | AB598316 | AB598340 | – | *Matsui, 2011* |
| **205** | *Glyphoglossus molossus** | Thailand, Tak, Barrntak | KUHE 35182 | AB201182 | AB201193 | EF396009 | *Matsui et al., 2005* |
| **206** | *Glyphoglossus yunnanensis** | China, pet trade | KUHE 44148 | AB634626 | AB634684 | KM509234 | *Matsui et al., 2011* |
|  | **Outgroups** |  |  |  |  |  |  |
| **207** | *Chaperina fusca** | Malaysia, Sabah, Crocker | BORN 8478 | AB598318 | AB598342 | AB611868 | *Matsui, 2011* |
| **208** | *Ctenophryne geayi* | Brasil, Pará, Rio Xingu, Fazenda Caracol | MPEG 25397 | KM509124 | | AB611884 | *Peloso et al., 2016* |
| **209** | *Dyscophus guineti* | Pet trade | KUHE 33150 | AB634648 | AB634706 | KM509260 | *Matsui et al., 2011* |
| **210** | *Dyscophus insularis* | Pet trade | KUHE 35001 | AB634649 | AB634707 | – | *Matsui et al., 2011* |
| **211** | *Gastrophryne carolinensis** | USA, Florida, Camel Lake | CAS 214349 | KM509133 | | KM509266 | *Peloso et al., 2016* |
| **212** | *Gastrophryne olivacea** | USA, Texas, Dimmit | KUHE 33224 | AB634650 | AB634708 | AB611899 | *Matsui et al., 2011* |
| **213** | *Gastrophrynoides immaculatus* | Malaysia, Negeri Sembilan | UKM HC 279 | AB634647 | AB634705 | AB611904 | *Matsui et al., 2011* |
| **214** | *Kalophrynus pleurostigma* | Indonesia, Sumatra, Lampung | MZB Amp 15295 | AB634642 | AB634700 | AB611920 | *Matsui et al., 2011* |
| **215** | *Kalophrynus yongi* | Malaysia, Pahang, Cameron | KUHE 15531 | AB634646 | AB634704 | – | *Matsui et al., 2011* |
| **216** | *Kaloula baleata** | Indonesia, Sumba | KUHE 32313 | AB634629 | AB634687 | KM509289 | *Matsui et al., 2011* |
| **217** | *Kaloula rugifera** | China | – | KP682314 | | – | *Deng et al., 2015* |
| **218** | *Metaphrynella pollicaris** | Malaysia, Pahang, Fraser’s Hill | KUZ 21655 | AB634634 | AB634692 | AB611930 | *Matsui et al., 2011* |
| **219** | *Metaphrynella sundana** | Malaysia, Borneo, Sabah, Crocker | BORN 8191 | AB634635 | AB634693 | AB611938 | *Matsui et al., 2011* |
| **220** | *Micryletta inornata** | Thailand, Phrae, Mae Yom | KUHE 20497 | AB598317 | AB598341 | KM509304 | *Matsui, 2011* |
| **221** | *Micryletta nigromaculata** | Vietnam, Hai Phong, Cat Ba NP | ZMMU A5934 |  | MH756150 | MN534449 | *Poyarkov et al., 2018a; this work* |
| **222** | *Micryletta steinegeri** | China, Taiwan, Yunlin | KUHE 35937 | AB634638 | AB634696 | – | *Matsui et al., 2011* |
| **223** | *Oreophryne monticola* | Indonesia, Bali, Batu Karu | MZB Amp 16265 | AB634651 | AB634709 | KM509307 | *Matsui et al., 2011* |
| **224** | *Otophryne robusta* | Guyana, District 8, Wokomung Mt. | ROM 42963 | KM509171 | | KM509309 | *Peloso et al., 2016* |
| **225** | *Phrynella pulchra** | Malaysia, Trengganu, Hulu Trengganu | UKMHC 820 | AB634636 | AB634694 | AB611972 | *Matsui et al., 2011* |
| **226** | *Phrynomantis bifasciatus* | Pet trade | KUHE 33277 | AB634652 | AB634710 | KM509312 | *Matsui et al., 2011* |
| **227** | *Rhacophorus schlegelii** | Japan, Hiroshima | – | AB202078 | AB202078 | – | *Sano et al., 2005* |
| **228** | *Scaphiophryne gottlebei* | Pet trade | KUHE 34977 | AB634653 | AB634711 | KM509335 | *Matsui et al., 2011* |
| **229** | *Synapturanus salseri* | Brazil, Amazonas, Manaus | MZUSP | KM509207 | | KM509348 | *Peloso et al., 2016* |
| **230** | *Uperodon taprobanicus** | Sri Lanka | KUHE 37252 | AB634633 | AB634691 | AB611925 | *Matsui et al., 2011* |
| **231** | *Alytes dickhilleni** | Spain, Sierra Nevada, Parejo | – | AY333672 | AY333710 | EF407511 | *Fromhage et al., 2004* |
| **232** | *Alytes muletensis** | Spain, Mallorca | ZFMK 44683 | AY333671 | AY333709 | EF407510 | *Fromhage et al., 2004* |
| **233** | *Blommersia transmarina** | Comoro Islands | ZSM 652 2000 | AY341585 | AY341639 | EF396017 | *Fromhage et al., 2004* |
| **234** | *Blommersia wittei** | Madagascar, Montagne dAmbre | 2002 876 | AY341586 | AY848105 | EF396018 | *Fromhage et al., 2004* |
