# Supplementary material for: A little frog leaps a long way: compounded colonizations of the Indian Subcontinent discovered in the tiny Oriental frog genus Microhyla (Amphibia: Microhylidae)
Source: PeerJ. 2020 Jul 3;8:e9411. doi: 10.7717/peerj.9411 (PMC7337035; doi:10.7717/peerj.9411)
Supplement: Supplemental Information 6 — “F,” “L”–forward primer, “R,” “H”–reverse primer. For references see Supplementary Information file 2. [file peerj-08-9411-s006.docx]

**Supplementary Table S2. Primers used in this study.**

“F,” “L”–forward primer, “R,” “H”–reverse primer. For references see Supplementary Information file 2.

| **Gene** | **Primer name** | **Primer sequence (5'—3')** | **Source** |
| --- | --- | --- | --- |
| 12S rRNA | 1F-12Stail | ACGCTAAAATGWACCCTAAAAAGT | *this work* |
| 12S rRNA | 600R-12Stail | TAGAGGAGCCTGTTCTATAATCGATTC | *this work* |
| 12S rRNA—16S rRNA | 500F-12Stail | CCACTTGAACCCACGACAGCTAGRAMACAA | *this work* |
| 12S rRNA—16S rRNA | 12SA-L | AAACTGGGATTAGATACCCCACTAT | *Palumbi et al., 1991* |
| 12S rRNA—16S rRNA | 1200R-12Stail | AGTAAAGGCGATYAAAAAATRTTTCAAAG | *this work* |
| 12S rRNA—16S rRNA | R-1169 | GTGGCTGCTTTTAGGCCCACT | *Nguyen et al., 2019* |
| 16S rRNA | L-2188 | AAAGTGGGCCTAAAAGCAGCCA | *Matsui et al., 2006* |
| 16S rRNA | 16SL-1 | CTGACCGTGCAAAGGTAGCGTAATCACT | *Hedges, 1994* |
| 16S rRNA | 16SH-1 | CTCCGGTCTGAACTCAGATCACGTAGG | *Hedges, 1994* |
| BDNF | BDNFAmpF1 | ACCATCCTTTTCCTTACTATGG | *Van der Meijden et al., 2007* |
| BDNF | BDNFAmpR1 | CTATCTTCCCCTTTTAATGGTC | *Van der Meijden et al., 2007* |
