# Supplementary material for: A little frog leaps a long way: compounded colonizations of the Indian Subcontinent discovered in the tiny Oriental frog genus Microhyla (Amphibia: Microhylidae)
Source: PeerJ. 2020 Jul 3;8:e9411. doi: 10.7717/peerj.9411 (PMC7337035; doi:10.7717/peerj.9411)
Supplement: Supplemental Information 7 — The optimal partitioning scheme and model fit was estimated as suggested by the Akaike information criterion (AIC). [file peerj-08-9411-s007.docx]

**Supplementary Table S3. The optimal evolutionary models for gene and codon partitions as estimated in PartitionFinder v1.0.1.**

The optimal partitioning scheme and model fit was estimated as suggested by the Akaike information criterion (AIC).

| **Gene** | **Substitution Model** |
| --- | --- |
| 12S rRNA | GTR+I+G |
| tRNA-Val | JC+G |
| 16S rRNA | GTR+I+G |
| BDNF codon position 1 | K2P+I |
| BDNF codon position 2 | K2P+G |
| BDNF codon position 3 | HKY+I+G |
