# Supplementary material for: A little frog leaps a long way: compounded colonizations of the Indian Subcontinent discovered in the tiny Oriental frog genus Microhyla (Amphibia: Microhylidae)
Source: PeerJ. 2020 Jul 3;8:e9411. doi: 10.7717/peerj.9411 (PMC7337035; doi:10.7717/peerj.9411)
Supplement: Supplemental Information 8 — Node – tree node used for calibration, for node names see Fig. S3; divergence time given in millions years (Ma). For references see Supplementary Information file 2. [file peerj-08-9411-s008.docx]

**Supplementary Table S4. Calibration points for divergence time estimation.**

Node – tree node used for calibration, for node names see Fig. S3; divergence time given in millions years (Ma). For references see Supplementary Information file 2.

| **Node** | **Cladogenetic event** | **Estimated date (Ma)** | **Standart deviation (Ma)** | **Prior distribution** | **Reference** |
| --- | --- | --- | --- | --- | --- |
| **17** | *Microhyla* – *Glyphoglossus* assemblage basal split | 44.54 | 7.01 | normal | *Kurabayashi et al., 2011* |
| **30** | Divergence between MRCA of *M.* *butleri* species group and other *Microhyla* | 18.98 | 3.85 | normal | *Kurabayashi et al., 2011* |
| **57** | Split between MRCA of *M. achatina* and *M. fissipes* species groups | 13.89 | 3.03 | normal | *Kurabayashi et al., 2011* |
| **6** | Split between *Gastrophryne carolinensis* and *G. olivacea* | 1.7 | 0.4 | lognormal | *Holman, 2003* |
| **2** | Split between *Alytes muletensis* and *A. dickhilleni* | [20 - 5] | - | uniform | *Fromhage et al., 2004* |
| **4** | Split between *Blommersia transmarina* and *B. wittei* | [15 - 1] | - | uniform | *Vences et al., 2003* |
