# Supplementary material for: A little frog leaps a long way: compounded colonizations of the Indian Subcontinent discovered in the tiny Oriental frog genus Microhyla (Amphibia: Microhylidae)
Source: PeerJ. 2020 Jul 3;8:e9411. doi: 10.7717/peerj.9411 (PMC7337035; doi:10.7717/peerj.9411)
Supplement: Supplemental Information 9 — Geographic regions: (A) Mainland East Asia; (B) Eastern Indochina; (C) Western Indochina; (D) Indian Subcontinent; (E) Malayan Peninsula; (F) Sumatra - Java - Bali; (G) Borneo and Philippines; (H) Sri Lanka; (I) East Asian Islands; see Fig. 2. No. corresponds to specimen number in Table S1. [file peerj-08-9411-s009.docx]

**Supplementary Table S5. Matrix of modern species distribution within the *Microhyla – Glyphoglossus* assemblage.**

Geographic regions: (A) Mainland East Asia; (B) Eastern Indochina; (C) Western Indochina; (D) Indian Subcontinent; (E) Malayan Peninsula; (F) Sumatra - Java - Bali; (G) Borneo and Philippines; (H) Sri Lanka; (I) East Asian Islands; see Fig. 2. No. corresponds to specimen number in Table S1.

| **No.** | **Species** | **Range** |
| --- | --- | --- |
| 1 | *M. achatina* | F |
| 4 | *M. achatina* | F |
| 7 | *M. annamensis* | B |
| 11 | *M. annectens* | E |
| 13 | *M. arboricola* | B |
| 16 | *M. arboricola* | B |
| 17 | *M. aurantiventris* | B |
| 20 | *M. beilunensis* | A |
| 22 | *M. berdmorei* | F |
| 24 | *M. berdmorei* | E |
| 26 | *M. berdmorei* | G |
| 27 | *M. berdmorei* | E |
| 28 | *M. berdmorei* | D |
| 33 | *M. berdmorei* | B |
| 38 | *M. borneensis* | G |
| 42 | *M. butleri* | C |
| 43 | *M. butleri* | E |
| 44 | *M. butleri* | A |
| 46 | *M. butleri* | B |
| 49 | *M. butleri* | I |
| 50 | *M. chakrapanii* | C |
| 51 | *M. chakrapanii* | C |
| 52 | *M. darreli* | D |
| 53 | *M. eos* | D |
| 54 | *M. fanjingshanensis* | A |
| 56 | *M. fissipes* | A |
| 57 | *M. fissipes* | I |
| 58 | *M. fissipes* | A |
| 61 | *M. fodiens* | C |
| 62 | *M. gadjahmadai* | F |
| 64 | *M. heymonsi* | E |
| 67 | *M. heymonsi* | C |
| 68 | *M. heymonsi* | I |
| 70 | *M. heymonsi* | C |
| 72 | *M. heymonsi* | E |
| 73 | *M. heymonsi* | F |
| 74 | *M. heymonsi* | C |
| 77 | *M. heymonsi* | B |
| 78 | *M. irrawaddy* | C |
| 82 | *M. karunaratnei* | H |
| 85 | *M. kodial* | D |
| 86 | *M. laterite* | D |
| 90 | *M. malang* | G |
| 92 | *M. malang* | G |
| 93 | *M. mantheyi* | E |
| 97 | *M. mantheyi* | E |
| 99 | *M. marmorata* | B |
| 106 | *M. mihintalei* | H |
| 109 | *M. minuta* | B |
| 112 | *M. mixtura* | A |
| 113 | *M. mukhlesuri* | C |
| 114 | *M. mukhlesuri* | B |
| 115 | *M. mukhlesuri* | B |
| 116 | *M. mukhlesuri* | E |
| 117 | *M. mukhlesuri* | E |
| 119 | *M. mukhlesuri* | B |
| 120 | *M. mukhlesuri* | D |
| 121 | *M. mukhlesuri* | E |
| 122 | *M. mukhlesuri* | C |
| 124 | *M. mymensinghensis* | D |
| 126 | *M. mymensinghensis* | D |
| 127 | *M. mymensinghensis* | D |
| 128 | *M. nanapollexa* | B |
| 129 | *M. nanapollexa* | B |
| 132 | *M. nepenthicola* | G |
| 134 | *M. nilphamariensis* | D |
| 140 | *M. okinavensis* | I |
| 141 | *M. okinavensis* | I |
| 143 | *M. orientalis* | F |
| 145 | *M. orientalis* | F |
| 146 | *M. ornata* | D |
| 148 | *M. ornata* | H |
| 149 | *M. palmipes* | F |
| 150 | *M. palmipes* | F |
| 151 | *M. perparva* | G |
| 152 | *M. perparva* | G |
| 153 | *M. petrigena* | G |
| 154 | *M. petrigena* | G |
| 157 | *M. picta* | B |
| 160 | *M. pineticola* | B |
| 165 | *M. pulchella* | B |
| 170 | *M. pulchra* | B |
| 172 | *M. pulverata* | B |
| 176 | *M. rubra* | D |
| 179 | *M. sholigari* | D |
| 180 | *M. superciliaris* | E |
| 182 | *M. superciliaris* | E |
| 184 | *M. taraiensis* | D |
| 186 | *M. zeylanica* | H |
| 188 | *Microhyla* sp. 1 | G |
| 191 | *Microhyla* sp. 2 | E |
| 192 | *Microhyla* sp. 2 | C |
| 193 | *Microhyla* sp. 3 | I |
| 194 | *Microhyla* sp. 3 | I |
| 198 | *Microhyla* sp. 4 | C |
| 200 | *Glyphoglossus capsus* | G |
| 203 | *Glyphoglossus guttulatus* | B |
| 204 | *Glyphoglossus minutus* | E |
| 205 | *Glyphoglossus molossus* | C |
| 206 | *Glyphoglossus yunnanensis* | A |
