# Supplementary material for: A little frog leaps a long way: compounded colonizations of the Indian Subcontinent discovered in the tiny Oriental frog genus Microhyla (Amphibia: Microhylidae)
Source: PeerJ. 2020 Jul 3;8:e9411. doi: 10.7717/peerj.9411 (PMC7337035; doi:10.7717/peerj.9411)
Supplement: Supplemental Information 10 — Four time periods correspond to: (1) 100–57 MYA marks the complete isolation of the ISC from Eurasia; (2) 57–50 MYA marks the first assumed land connections between India and the modern-day Sumatra; (3) during 50–35 MYA the ISC likely continued the counter-clockwise moving northwards forming land bridges with the modern-day Indo-Burma; and (4) the period of 35–0.0 MYA corresponds to the firm collision and formation of a stable land connection between the ISC and Eurasia. Letters encode: (A) Mainland East Asia; (B) Eastern Indochina; (C) Western Indochina; (D) Indian Subcontinent; (E) Malayan Peninsula; (F) Sumatra - Java - Bali; (G) Borneo and Philippines; (H) Sri Lanka; (I) East Asian Islands; see Fig. 2. [file peerj-08-9411-s010.docx]

**Supplementary Table S6. Step-matrix showing dispersal constraints between biogeographic areas.**

Four time periods correspond to: (1) 100–57 MYA marks the complete isolation of the ISC from Eurasia; (2) 57–50 MYA marks the first assumed land connections between India and the modern-day Sumatra; (3) during 50–35 MYA the ISC likely continued the counter-clockwise moving northwards forming land bridges with the modern-day Indo-Burma; and (4) the period of 35–0.0 MYA corresponds to the firm collision and formation of a stable land connection between the ISC and Eurasia. Letters encode: (A) Mainland East Asia; (B) Eastern Indochina; (C) Western Indochina; (D) Indian Subcontinent; (E) Malayan Peninsula; (F) Sumatra - Java - Bali; (G) Borneo and Philippines; (H) Sri Lanka; (I) East Asian Islands; see Fig. 2.

| **(1) 100–57 Ma** | | |  |  |  |  |  |  |  |
| --- | --- | --- | --- | --- | --- | --- | --- | --- | --- |
|  | **A** | **B** | **C** | **D** | **E** | **F** | **G** | **H** | **I** |
| **A** |  | 1 | 1 | 0 | 0 | 0 | 0 | 0 | 1 |
| **B** | 1 |  | 1 | 0 | 1 | 0 | 1 | 0 | 0 |
| **C** | 1 | 1 |  | 0 | 1 | 0 | 1 | 0 | 0 |
| **D** | 0 | 0 | 0 |  | 0 | 0 | 0 | 1 | 0 |
| **E** | 0 | 1 | 1 | 0 |  | 1 | 1 | 0 | 0 |
| **F** | 0 | 0 | 0 | 0 | 1 |  | 1 | 0 | 0 |
| **G** | 0 | 1 | 1 | 0 | 1 | 1 |  | 0 | 0 |
| **H** | 0 | 0 | 0 | 1 | 0 | 0 | 0 |  | 0 |
| **I** | 1 | 0 | 0 | 0 | 0 | 0 | 0 | 0 |  |
| **(2) 57–50 Ma** | | |  |  |  |  |  |  |  |
|  | **A** | **B** | **C** | **D** | **E** | **F** | **G** | **H** | **I** |
| **A** |  | 1 | 1 | 0 | 0 | 0 | 0 | 0 | 1 |
| **B** | 1 |  | 1 | 0 | 1 | 0 | 1 | 0 | 0 |
| **C** | 1 | 1 |  | 0 | 1 | 0 | 1 | 0 | 0 |
| **D** | 0 | 0 | 0 |  | 1 | 1 | 0 | 1 | 0 |
| **E** | 0 | 1 | 1 | 1 |  | 1 | 1 | 0 | 0 |
| **F** | 0 | 0 | 0 | 1 | 1 |  | 1 | 0 | 0 |
| **G** | 0 | 1 | 1 | 0 | 1 | 1 |  | 0 | 0 |
| **H** | 0 | 0 | 0 | 1 | 0 | 0 | 0 |  | 0 |
| **I** | 1 | 0 | 0 | 0 | 0 | 0 | 0 | 0 |  |
| **(3) 50–35 Ma** | | |  |  |  |  |  |  |  |
|  | **A** | **B** | **C** | **D** | **E** | **F** | **G** | **H** | **I** |
| **A** |  | 1 | 1 | 0 | 0 | 0 | 0 | 0 | 1 |
| **B** | 1 |  | 1 | 0 | 1 | 0 | 1 | 0 | 0 |
| **C** | 1 | 1 |  | 1 | 1 | 0 | 1 | 0 | 0 |
| **D** | 0 | 0 | 1 |  | 1 | 0 | 0 | 1 | 0 |
| **E** | 0 | 1 | 1 | 1 |  | 1 | 1 | 0 | 0 |
| **F** | 0 | 0 | 0 | 0 | 1 |  | 1 | 0 | 0 |
| **G** | 0 | 1 | 1 | 0 | 1 | 1 |  | 0 | 0 |
| **H** | 0 | 0 | 0 | 1 | 0 | 0 | 0 |  | 0 |
| **I** | 1 | 0 | 0 | 0 | 0 | 0 | 0 | 0 |  |
|  | | |  |  |  |  |  |  |  |
|  | | |  |  |  |  |  |  |  |
|  | | |  |  |  |  |  |  |  |
| **(4) 35–0.0 Ma** | | |  |  |  |  |  |  |  |
|  | **A** | **B** | **C** | **D** | **E** | **F** | **G** | **H** | **I** |
| **A** |  | 1 | 1 | 0 | 0 | 0 | 0 | 0 | 1 |
| **B** | 1 |  | 1 | 0 | 1 | 0 | 1 | 0 | 0 |
| **C** | 1 | 1 |  | 1 | 1 | 0 | 1 | 0 | 0 |
| **D** | 0 | 0 | 1 |  | 0 | 0 | 0 | 1 | 0 |
| **E** | 0 | 1 | 1 | 0 |  | 1 | 1 | 0 | 0 |
| **F** | 0 | 0 | 0 | 0 | 1 |  | 1 | 0 | 0 |
| **G** | 0 | 1 | 1 | 0 | 1 | 1 |  | 0 | 0 |
| **H** | 0 | 0 | 0 | 1 | 0 | 0 | 0 |  | 0 |
| **I** | 1 | 0 | 0 | 0 | 0 | 0 | 0 | 0 |  |
