# Supplementary material for: A little frog leaps a long way: compounded colonizations of the Indian Subcontinent discovered in the tiny Oriental frog genus Microhyla (Amphibia: Microhylidae)
Source: PeerJ. 2020 Jul 3;8:e9411. doi: 10.7717/peerj.9411 (PMC7337035; doi:10.7717/peerj.9411)
Supplement: Supplemental Information 11 — For each species minimal, maximal and average (when available) body size data is given for both sexes. For references see File S2. Question mark denotes “no data”. Voucher IDs for specimens measured for this study are as follows: Microhyla fodiens: CAS 215851, ZMMU A5960–A5961; Microhyla irrawaddy: ZMMU A5966–A5967; ZMMU A5975–A5976; Microhyla nanapollexa: ZMMU A5635; Microhyla sp. 2: ZMMU A6032–A6035, KIZ-031270–031273; Microhyla sp. 3: ZMMU NAP-6340–6341. [file peerj-08-9411-s011.docx]

**Supplementary Table S7. Body size data for the *Microhyla – Glyphoglossus* assemblage members.**

For each species minimal, maximal and average (when available) body size data is given for both sexes. For references see Supplementary Information file 2. Question mark denotes “no data”. Voucher IDs for specimens measured for this study are given below.

| **№** | **Species** | ♂ | | | ♀ | | | **Source** |
| --- | --- | --- | --- | --- | --- | --- | --- | --- |
|  |  | **min** | **max** | **mean** | **min** | **max** | **mean** |  |
| **1** | *Microhyla achatina* | 19.7 | 23.0 |  | 24.0 | 26.1 |  | *Manthey & Grossmann, 1997; Manthey & Denzer, 2014* |
| **2** | *Microhyla annamensis* | 15.2 | 19.8 |  | 18.2 | 22.6 |  | *Bain & Nguyen, 2004; Poyarkov et al., 2014* |
| **3** | *Microhyla annectens* | 14.4 | 15.6 |  | 18.2 | 18.4 |  | *Inger & Frogner, 1979; Bain & Nguyen, 2004; Poyarkov et al., 2014* |
| **4** | *Microhyla arboricola* | 13.2 | 15.0 |  | 15.9 | 17.0 |  | *Poyarkov et al., 2014* |
| **5** | *Microhyla aurantiventris* | 25.2 | 27.0 |  |  | 30.5 |  | *Nguyen et al., 2019* |
| **6** | *Microhyla beilunensis* | 19.1 | 23.7 | 21.9 | 26.4 | 28.3 | 27.6 | *Zhang et al., 2019* |
| **7** | *Microhyla berdmorei* | 23.8 | 32.5 |  | 26.2 | 45.6 |  | *Bain & Nguyen, 2004; Poyarkov et al., 2014* |
| **8** | *Microhyla borneensis* | 13.8 | |  | 17.3 | |  | *Das & Haas, 2010; Matsui, 2011* |
| **9** | *Microhyla butleri* | 20.0 | 25.0 |  | 21.0 | 26.0 |  | *Bain & Nguyen, 2004; Poyarkov et al., 2014* |
| **10** | *Microhyla chakrapanii* |  |  | 22.0 |  |  |  | *Pillai, 1977* |
| **11** | *Microhyla darevskii* | 27.0 | 32.6 |  | ? | | - | *Poyarkov et al., 2014* |
| **12** | *Microhyla darreli* |  | 15.1 |  |  |  |  | *Garg et al., 2019* |
| **13** | *Microhyla eos* |  | 21.5 |  | 26.9 | 27.8 |  | *Biju et al., 2019* |
| **14** | *Microhyla fanjingshanensis* |  | 22.7 | 21.4 |  | 23.0 | 22.7 | *Li et al., 2019* |
| **15** | *Microhyla fissipes* | 18.0 | 27.5 |  | 20.0 | 28.0 |  | *Kuramoto & Joshy, 2007; Poyarkov et al., 2014* |
| **16** | *Microhyla fodiens* | 20.1 | 29.1 |  | 20.0 | 30.0 |  | *Poyarkov et al., 2019*; this study |
| **17** | *Microhyla fusca* | 23.0 | | 23.0 | ? | | - | *Andersson, 1942; Bain & Nguyen, 2004* |
| **18** | *Microhyla gadjahmadai* | 18.2 | 21.3 | 20.7 | 20.3 | 25.5 | 20.9 | *Atmaja et al., 2018* |
| **19** | *Microhyla heymonsi* | 16.5 | 22.0 |  | 18.0 | 26.5 |  | *Bain & Nguyen, 2004; Fei et al., 2009; Matsui et al., 2013* |
| **20** | *Microhyla irrawaddy* | 12.3 | 17.1 |  | 16.7 | 20.9 |  | *Poyarkov et al., 2019*; this study |
| **21** | *Microhyla karunaratnei* | 15.8 | 19.1 |  | 19.6 | 21.0 |  | *Fernando & Siriwardhane, 1996; Dutta and Manamendra-Arachchi, 1997; Dutta and Ray, 2000* |
| **22** | *Microhyla kodial* | 16.9 | 17.4 |  | 18.0 | 20.4 |  | *Vineeth et al., 2018* |
| **23** | *Microhyla laterite* | 15.3 | 16.6 |  | 18.4 | |  | *Seshadri et al., 2016* |
| **24** | *Microhyla malang* | 18.7 | 22.2 |  | 19.0 | 23.4 |  | *Das & Haas, 2010; Matsui, 2011* |
| **25** | *Microhyla mantheyi* | 15.0 | 29.2 |  | 14.8 | 24.1 |  | *Das et al., 2007; Poyarkov et al., 2014* |
| **26** | *Microhyla marmorata* | 18.8 | 21.5 |  | 21.1 | 23.2 |  | *Bain & Nguyen, 2004; Poyarkov et al., 2014* |
| **27** | *Microhyla mihintalei* | 21.7 | 27.3 |  | 24.4 | |  | *Wijayathilaka et al., 2016* |
| **28** | *Microhyla minuta* | 14.7 | 15.9 |  | 15.7 | 17.2 |  | *Poyarkov et al., 2014* |
| **29** | *Microhyla mixtura* | 20.5 | 23.7 |  | 23.8 | 26.6 |  | *Bain & Nguyen, 2004; Poyarkov et al., 2014; Li et al., 2019* |
| **30** | *Microhyla mukhlesuri* | 16.5 | 21.0 |  | 17.3 | 18.4 |  | *Hasan et al., 2014* |
| **31** | *Microhyla mymensinghensis* | 14.2 | 17.6 |  | 15.2 | 21.3 |  | *Hasan et al., 2014* |
| **32** | *Microhyla nanapollexa* | 13.5 | |  | 16.6 | |  | *Bain & Nguyen, 2004*; this study |
| **33** | *Microhyla nepenthicola* | 10.6 | 12.8 |  | 17.9 | 18.8 |  | *Das & Haas, 2010* |
| **34** | *Microhyla nilphamariensis* | 17.4 | |  | 18.3 | |  | *Hasan et al., 2015* |
| **35** | *Microhyla okinavensis* | 22.5 | 27.2 |  | 26.5 | 28.6 |  | *Kuramoto & Joshy, 2007; Poyarkov et al., 2014* |
| **36** | *Microhyla orientalis* | 15.8 | 17.4 |  | 18.3 | 19.2 |  | *Matsui et al., 2013* |
| **37** | *Microhyla ornata* | 13.4 | 24.9 |  | 24.9 | 26.2 |  | *Dutta and Manamendra-Arachchi, 1997; Kuramoto & Joshy, 2007; Poyarkov et al., 2014* |
| **38** | *Microhyla palmipes* | 16.0 | |  | 21.8 | |  | *Parker, 1928; Bain & Nguyen, 2004; Poyarkov et al., 2014; Manthey and Denzer, 2014* |
| **39** | *Microhyla perparva* | 10.5 | 11.9 |  | 12.4 | 14.5 |  | *Bain & Nguyen, 2004* |
| **40** | *Microhyla petrigena* | 13.9 | 16.2 |  | 15.1 | 17.8 |  | *Bain & Nguyen, 2004* |
| **41** | *Microhyla picta* | 25.2 | 30.1 |  | 27.2 | 33.4 |  | *Bain & Nguyen, 2004; Schenkel, 1901* |
| **42** | *Microhyla pineticola* | 17.2 | 19.5 |  | 18.0 | 23.0 |  | *Poyarkov et al., 2014* |
| **43** | *Microhyla pulchella* | 14.7 | 21.6 |  | 18.1 | 25.8 |  | *Poyarkov et al., 2014* |
| **44** | *Microhyla pulchra* | 23.0 | 32.0 |  | 28.0 | 36.5 |  | *Bain & Nguyen, 2004; Fei et al., 2009; Poyarkov et al., 2014* |
| **45** | *Microhyla pulverata* | 17.5 |  |  | 18.8 | 20.2 |  | *Bain & Nguyen, 2004; Poyarkov et al., 2014* |
| **46** | *Microhyla rubra* | 20.0 | 27.5 |  | 20.5 | 29.5 |  | *Dutta &Manamendra-Arachchi, 1997* |
| **47** | *Microhyla sholigari* | 15.9 | 16.2 |  | 15.9 | 19.2 |  | *Dutta & Ray, 2000* |
| **48** | *Microhyla superciliaris* | 12.7 | |  | 12.0 | |  | *Parker, 1928; Dutta and Ray, 2000; Bain & Nguyen, 2004* |
| **49** | *Microhyla taraiensis* | 19.9 | 20.3 |  | 22.1 | 24.9 |  | *Khatiwada et al., 2017* |
| **50** | *Microhyla zeylanica* | 14.4 | 18.3 |  | 15.8 | 20.0 |  | *Parker & Osman-Hill, 1949; Dutta and Manamendra-Arachchi, 1997; Dutta and Ray, 2000* |
| **51** | *Microhyla* sp. 1 | 12.0 | 13.3 |  | 11.8 | |  | *Inger, 1989* |
| **52** | *Microhyla* sp. 2 | 10.1 | 13.7 |  | 15.8 | 17.6 | 16.3 | this study |
| **53** | *Microhyla* sp. 3 | 24.1 | 28.2 |  | 28.6 | 30.8 |  | *Kuramoto & Joshy, 2007*; this study |
| **54** | *Glyphoglossus capsus* | 34.2 | 36.0 |  | - | 36.0 |  | *Das et al., 2014* |
| **55** | *Glyphoglossus guttulatus* | 34.0 | 47.0 |  | 38.0 | 54.0 |  | *Neang & Holden, 2008; Nguyen et al., 2009* |
| **56** | *Glyphoglossus minutus* | 25.5 | 30.9 |  | - | 32.7 |  | *Das et al., 2004* |
| **57** | *Glyphoglossus molossus* | 41.0 | 94.9 |  | 50.6 | 81.9 |  | *Neang & Holden, 2008; Nguyen et al., 2009* |
| **58** | *Glyphoglossus yunnanensis* | 30.0 | 35.5 |  | 40.0 | 48.8 |  | *Yang, 1991* |
| **59** | *Kaloula baleata* | - | 60.0 |  | - | 65.0 |  | *Pauwels et al., 1999* |

**Specimens examined for this study:**

*Microhyla fodiens*: CAS 215851, ZMMU A5960–A5961

*Microhyla irrawaddy*: ZMMU A5966–A5967; ZMMU A5975–A5976

*Microhyla nanapollexa*: ZMMU A5635

*Microhyla* sp. 2: ZMMU A6032–A6035, KIZ-031270–031273

*Microhyla* sp. 3: ZMMU NAP-6340–6341
