# Supplementary material for: A little frog leaps a long way: compounded colonizations of the Indian Subcontinent discovered in the tiny Oriental frog genus Microhyla (Amphibia: Microhylidae)
Source: PeerJ. 2020 Jul 3;8:e9411. doi: 10.7717/peerj.9411 (PMC7337035; doi:10.7717/peerj.9411)
Supplement: Supplemental Information 12 — Total length (in b.p.), number of conservative (Cons.), variable (Var.) and parsimony-informative (Pars.-Inf.) sites are given (data presented only for the ingroup). [file peerj-08-9411-s012.docx]

**Supplementary Table S8. Characteristics of analyzed mtDNA and nuDNA sequences.**

Total length (in b.p.), number of conservative (Cons.), variable (Var.) and parsimony-informative (Pars.-Inf.) sites are given (data presented only for the ingroup).

| **Genetic marker** | | **Sites (in b.p.)** | | | |
| --- | --- | --- | --- | --- | --- |
|  |  | **Cons.** | **Var.** | **Pars.-Inf.** | **Total** |
| **1** | BDNF alignment | 618.0 | 96.0 | 67.0 | 717.0 |
| **2** | mtDNA alignment | 1204.0 | 1248.0 | 1077.0 | 2478.0 |
