# Supplementary material for: A little frog leaps a long way: compounded colonizations of the Indian Subcontinent discovered in the tiny Oriental frog genus Microhyla (Amphibia: Microhylidae)
Source: PeerJ. 2020 Jul 3;8:e9411. doi: 10.7717/peerj.9411 (PMC7337035; doi:10.7717/peerj.9411)
Supplement: Supplemental Information 13 — Uncorrected average interspecific (below diagonal) and intraspecific (on the diagonal) genetic p-distances for 16S rRNA mtDNA gene fragment (in percentage) are given for species of the Microhyla – Glyphoglossus assemblage (1–57). [file peerj-08-9411-s013.docx]

**Supplementary Table S9. Genetic divergence of the *Microhyla – Glyphoglossus* assemblage (continues on next page).**

Uncorrected average interspecific (below diagonal) and intraspecific (on the diagonal) genetic p-distances for 16S rRNA mtDNA gene fragment (in percentage) are given for species of the *Microhyla – Glyphoglossus* assemblage (1–57).

|  | **Species** | **1** | **2** | **3** | **4** | **5** | **6** | **7** | **8** | **9** | **10** | **11** | **12** | **13** | **14** | **15** | **16** | **17** | **18** | **19** | **20** | **21** | **22** | **23** | **24** | **25** | **26** | **27** | **28** | **29** |
| --- | --- | --- | --- | --- | --- | --- | --- | --- | --- | --- | --- | --- | --- | --- | --- | --- | --- | --- | --- | --- | --- | --- | --- | --- | --- | --- | --- | --- | --- | --- |
| **1** | *M. nepenthicola* | **0.1** |  |  |  |  |  |  |  |  |  |  |  |  |  |  |  |  |  |  |  |  |  |  |  |  |  |  |  |  |
| **2** | *M. borneensis* | 4.6 | **NA** |  |  |  |  |  |  |  |  |  |  |  |  |  |  |  |  |  |  |  |  |  |  |  |  |  |  |  |
| **3** | *Microhyla* sp. 1 | 3.1 | 7.1 | **0.3** |  |  |  |  |  |  |  |  |  |  |  |  |  |  |  |  |  |  |  |  |  |  |  |  |  |  |
| **4** | *M. malang* | 2.7 | 7.5 | 3.5 | **2.4** |  |  |  |  |  |  |  |  |  |  |  |  |  |  |  |  |  |  |  |  |  |  |  |  |  |
| **5** | *M. orientalis* | 5.4 | 10.8 | 6.6 | 6.0 | **1.0** |  |  |  |  |  |  |  |  |  |  |  |  |  |  |  |  |  |  |  |  |  |  |  |  |
| **6** | *M. mantheyi* | 6.1 | 10.1 | 6.0 | 7.1 | 6.1 | **0.7** |  |  |  |  |  |  |  |  |  |  |  |  |  |  |  |  |  |  |  |  |  |  |  |
| **7** | *M. minuta* | 6.1 | 10.1 | 7.2 | 6.4 | 7.2 | 7.7 | **0.0** |  |  |  |  |  |  |  |  |  |  |  |  |  |  |  |  |  |  |  |  |  |  |
| **8** | *M. achatina* | 7.6 | 12.1 | 8.4 | 8.6 | 6.9 | 7.4 | 9.1 | **2.1** |  |  |  |  |  |  |  |  |  |  |  |  |  |  |  |  |  |  |  |  |  |
| **9** | *M. gadjahmadai* | 7.5 | 11.8 | 7.7 | 8.4 | 7.5 | 6.8 | 7.9 | 6.4 | **3.3** |  |  |  |  |  |  |  |  |  |  |  |  |  |  |  |  |  |  |  |  |
| **10** | *M. kodial* | 7.7 | 12.9 | 8.1 | 8.7 | 8.4 | 7.3 | 9.5 | 8.6 | 9.0 | **0.3** |  |  |  |  |  |  |  |  |  |  |  |  |  |  |  |  |  |  |  |
| **11** | *Microhyla* sp. 4 | 9.3 | 14.7 | 9.5 | 9.7 | 9.1 | 8.7 | 9.4 | 8.1 | 9.0 | 6.5 | **0.0** |  |  |  |  |  |  |  |  |  |  |  |  |  |  |  |  |  |  |
| **12** | *M. irrawaddy* | 8.8 | 15.2 | 8.5 | 9.2 | 9.0 | 8.7 | 9.9 | 8.1 | 8.5 | 6.0 | 2.3 | **0.0** |  |  |  |  |  |  |  |  |  |  |  |  |  |  |  |  |  |
| **13** | *M. pineticola* | 8.5 | 14.0 | 9.4 | 8.4 | 8.5 | 9.5 | 9.0 | 10.1 | 10.4 | 10.1 | 10.8 | 10.0 | **0.1** |  |  |  |  |  |  |  |  |  |  |  |  |  |  |  |  |
| **14** | *M. heymonsi* | 8.1 | 13.6 | 7.7 | 7.9 | 8.6 | 8.8 | 8.9 | 9.4 | 10.4 | 8.1 | 9.9 | 9.0 | 8.4 | **3.9** |  |  |  |  |  |  |  |  |  |  |  |  |  |  |  |
| **15** | *M. fodiens* | 13.7 | 18.9 | 13.3 | 13.7 | 13.9 | 12.7 | 13.5 | 12.4 | 13.8 | 13.6 | 13.5 | 13.5 | 12.0 | 11.6 | **0.0** |  |  |  |  |  |  |  |  |  |  |  |  |  |  |
| **16** | *M. mukhlesuri* | 9.9 | 15.6 | 10.3 | 9.5 | 9.1 | 8.6 | 10.2 | 9.2 | 9.7 | 7.9 | 9.5 | 9.1 | 9.5 | 8.4 | 12.6 | **1.8** |  |  |  |  |  |  |  |  |  |  |  |  |  |
| **17** | *M. fissipes* | 9.1 | 15.2 | 9.3 | 9.2 | 8.5 | 8.0 | 9.6 | 9.0 | 9.1 | 7.4 | 9.6 | 8.4 | 9.4 | 8.0 | 12.5 | 2.4 | **0.3** |  |  |  |  |  |  |  |  |  |  |  |  |
| **18** | *M. chakrapanii* | 10.2 | 15.7 | 11.0 | 9.9 | 10.4 | 9.1 | 10.2 | 10.4 | 9.1 | 9.8 | 10.4 | 10.2 | 11.4 | 9.7 | 13.7 | 5.5 | 4.4 | **0.0** |  |  |  |  |  |  |  |  |  |  |  |
| **19** | *M. mymensinghensis* | 9.4 | 14.4 | 9.8 | 9.4 | 9.8 | 8.8 | 9.9 | 8.9 | 10.0 | 7.9 | 9.5 | 8.8 | 9.7 | 8.0 | 12.8 | 4.4 | 3.9 | 3.6 | **0.4** |  |  |  |  |  |  |  |  |  |  |
| **20** | *Microhyla* sp. 3 | 9.4 | 15.3 | 9.9 | 9.1 | 8.7 | 8.9 | 9.1 | 9.8 | 9.4 | 9.1 | 10.0 | 8.7 | 9.3 | 9.1 | 11.8 | 6.4 | 5.2 | 7.0 | 6.7 | **0.6** |  |  |  |  |  |  |  |  |  |
| **21** | *M. okinavensis* | 10.1 | 14.6 | 10.2 | 10.1 | 8.9 | 9.4 | 10.2 | 8.9 | 9.1 | 10.1 | 10.7 | 9.9 | 10.0 | 9.7 | 11.7 | 7.0 | 6.2 | 8.0 | 7.7 | 4.8 | **2.5** |  |  |  |  |  |  |  |  |
| **22** | *M. beilunensis* | 8.2 | 14.3 | 9.1 | 8.1 | 7.5 | 7.4 | 8.5 | 8.2 | 8.1 | 7.6 | 8.8 | 7.5 | 8.0 | 7.9 | 11.0 | 4.9 | 3.9 | 5.7 | 5.5 | 3.0 | 3.7 | **0.3** |  |  |  |  |  |  |  |
| **23** | *M. mixtura* | 9.3 | 14.7 | 10.7 | 9.4 | 9.6 | 8.8 | 9.6 | 9.7 | 9.5 | 8.5 | 9.6 | 8.8 | 9.1 | 9.0 | 11.3 | 6.5 | 5.4 | 7.1 | 6.0 | 2.8 | 5.5 | 3.7 | **0.7** |  |  |  |  |  |  |
| **24** | *M. fanjingshanensis* | 8.2 | 13.5 | 8.4 | 8.2 | 8.0 | 6.9 | 7.5 | 8.2 | 8.7 | 7.6 | 9.1 | 8.9 | 7.8 | 7.3 | 11.3 | 5.9 | 5.0 | 6.7 | 5.3 | 5.1 | 4.7 | 2.7 | 4.7 | **0.5** |  |  |  |  |  |
| **25** | *M. berdmorei* | 11.9 | 14.9 | 11.3 | 12.2 | 11.5 | 11.0 | 12.6 | 9.7 | 11.3 | 13.4 | 13.2 | 12.9 | 13.7 | 12.2 | 10.8 | 12.0 | 11.8 | 13.7 | 12.7 | 12.4 | 11.5 | 11.6 | 12.3 | 11.9 | **2.1** |  |  |  |  |
| **26** | *M. pulchra* | 13.6 | 16.9 | 12.4 | 12.3 | 12.7 | 12.5 | 13.7 | 10.3 | 11.9 | 13.4 | 13.5 | 13.1 | 13.3 | 11.5 | 12.3 | 11.1 | 11.2 | 10.7 | 11.4 | 10.8 | 10.3 | 9.8 | 12.0 | 10.8 | 9.4 | **0.7** |  |  |  |
| **27** | *M. picta* | 14.4 | 18.9 | 13.5 | 14.2 | 13.9 | 13.9 | 15.1 | 11.9 | 13.7 | 13.6 | 15.1 | 15.1 | 14.3 | 13.7 | 12.8 | 12.7 | 13.3 | 14.1 | 13.8 | 13.9 | 13.0 | 12.9 | 14.0 | 13.1 | 9.3 | 6.9 | **0.0** |  |  |
| **28** | *M. nilphamariensis* | 11.2 | 17.6 | 11.3 | 11.3 | 11.8 | 11.1 | 11.2 | 11.3 | 11.0 | 11.8 | 11.7 | 11.4 | 11.5 | 11.8 | 12.2 | 10.6 | 10.4 | 10.4 | 10.2 | 10.0 | 11.4 | 9.8 | 10.6 | 11.2 | 10.9 | 10.5 | 10.3 | **0.2** |  |
| **29** | *M. taraiensis* | 12.9 | 18.0 | 12.6 | 13.6 | 14.6 | 12.1 | 14.0 | 14.5 | 13.2 | 12.6 | 14.7 | 13.7 | 15.3 | 13.5 | 13.7 | 12.3 | 11.4 | 11.7 | 11.9 | 12.7 | 13.9 | 12.3 | 12.8 | 13.1 | 12.6 | 12.5 | 13.3 | 6.3 | **NA** |

**Supplementary Table S9. Genetic divergence of the *Microhyla – Glyphoglossus* assemblage (continued).**

|  | **Species** | **1** | **2** | **3** | **4** | **5** | **6** | **7** | **8** | **9** | **10** | **11** | **12** | **13** | **14** | **15** | **16** | **17** | **18** | **19** | **20** | **21** | **22** | **23** | **24** | **25** | **26** | **27** | **28** | **29** |
| --- | --- | --- | --- | --- | --- | --- | --- | --- | --- | --- | --- | --- | --- | --- | --- | --- | --- | --- | --- | --- | --- | --- | --- | --- | --- | --- | --- | --- | --- | --- |
| **30** | *M. ornata* | 13.5 | 18.7 | 13.8 | 13.0 | 13.8 | 13.5 | 13.2 | 12.7 | 13.1 | 14.1 | 14.4 | 13.9 | 12.7 | 12.1 | 12.4 | 11.5 | 11.3 | 10.8 | 10.9 | 11.9 | 11.5 | 11.5 | 12.2 | 12.2 | 11.8 | 11.2 | 11.7 | 6.1 | 8.4 |
| **31** | *M. rubra* | 12.4 | 16.6 | 13.3 | 12.6 | 13.3 | 12.3 | 12.9 | 12.0 | 12.3 | 13.3 | 13.7 | 13.5 | 13.7 | 12.6 | 13.7 | 11.2 | 11.2 | 12.3 | 12.3 | 12.9 | 13.7 | 11.8 | 12.4 | 11.8 | 11.4 | 11.6 | 10.8 | 7.4 | 8.6 |
| **32** | *M. mihintalei* | 12.0 | 16.1 | 12.4 | 12.2 | 12.6 | 11.2 | 12.5 | 12.2 | 11.6 | 12.9 | 13.0 | 13.5 | 13.6 | 12.1 | 13.3 | 11.6 | 11.2 | 11.8 | 12.3 | 12.9 | 13.5 | 11.6 | 12.3 | 11.8 | 11.9 | 11.3 | 10.9 | 7.5 | 9.2 |
| **33** | *M. aurantiventris* | 12.5 | 17.5 | 12.3 | 12.1 | 13.2 | 12.1 | 13.0 | 13.5 | 12.8 | 12.6 | 14.0 | 14.0 | 12.7 | 12.3 | 12.2 | 12.8 | 12.4 | 12.5 | 12.0 | 11.6 | 12.6 | 10.8 | 11.7 | 12.0 | 12.9 | 11.6 | 12.1 | 9.2 | 11.5 |
| **34** | *M. butleri* | 12.9 | 18.7 | 13.2 | 12.3 | 14.1 | 13.2 | 13.9 | 13.3 | 14.0 | 13.2 | 14.5 | 13.8 | 14.9 | 13.6 | 14.4 | 12.8 | 12.5 | 13.5 | 13.3 | 14.1 | 14.4 | 11.7 | 13.0 | 12.9 | 13.2 | 12.9 | 11.7 | 11.6 | 13.0 |
| **35** | *M. laterite* | 14.5 | 18.4 | 13.8 | 14.4 | 14.6 | 13.7 | 15.5 | 14.4 | 14.7 | 15.1 | 15.2 | 15.7 | 16.0 | 13.7 | 13.5 | 13.6 | 13.2 | 14.5 | 14.0 | 14.3 | 14.7 | 13.8 | 14.0 | 14.7 | 14.1 | 15.0 | 16.7 | 12.9 | 15.0 |
| **36** | *M. sholigari* | 14.2 | 17.5 | 13.6 | 14.3 | 14.6 | 13.4 | 14.5 | 14.4 | 14.8 | 14.6 | 15.2 | 15.5 | 15.3 | 13.5 | 13.5 | 13.2 | 13.1 | 14.2 | 13.7 | 14.6 | 14.8 | 13.3 | 14.3 | 13.5 | 12.2 | 14.1 | 14.4 | 11.4 | 12.4 |
| **37** | *M. darreli* | 13.5 | 18.4 | 13.3 | 13.6 | 14.4 | 13.8 | 14.0 | 14.3 | 15.4 | 14.8 | 15.7 | 15.2 | 14.2 | 12.8 | 13.2 | 13.5 | 13.1 | 14.5 | 13.7 | 14.0 | 14.7 | 12.8 | 13.9 | 13.7 | 13.4 | 14.5 | 14.1 | 10.7 | 12.7 |
| **38** | *M. zeylanica* | 14.5 | 20.3 | 13.1 | 14.1 | 15.1 | 14.1 | 14.7 | 14.2 | 14.3 | 15.4 | 15.5 | 15.2 | 16.3 | 14.4 | 15.2 | 13.1 | 12.7 | 14.5 | 14.5 | 14.1 | 15.3 | 14.1 | 13.5 | 15.3 | 14.4 | 16.1 | 17.7 | 13.9 | 15.0 |
| **39** | *M. karunaratnei* | 13.8 | 19.4 | 13.2 | 13.4 | 14.8 | 14.4 | 14.1 | 14.1 | 15.2 | 14.0 | 15.1 | 15.4 | 14.4 | 12.3 | 13.1 | 13.5 | 13.3 | 14.6 | 14.1 | 14.2 | 14.6 | 12.9 | 14.0 | 13.6 | 14.7 | 14.7 | 15.0 | 12.3 | 13.8 |
| **40** | *M. eos* | 12.2 | 14.7 | 11.5 | 11.8 | 12.9 | 11.1 | 12.7 | 12.9 | 12.8 | 11.8 | 12.4 | 12.4 | 12.5 | 10.6 | 12.4 | 10.8 | 10.9 | 11.7 | 11.2 | 12.0 | 13.1 | 10.8 | 11.8 | 11.9 | 11.9 | 13.1 | 14.9 | 10.5 | 11.4 |
| **41** | *Microhyla* sp. 2 | 12.3 | 15.8 | 11.7 | 12.2 | 13.7 | 12.6 | 13.5 | 11.8 | 13.9 | 13.7 | 14.6 | 14.3 | 13.7 | 11.6 | 12.8 | 10.9 | 10.7 | 12.3 | 11.6 | 13.3 | 11.7 | 10.9 | 13.4 | 11.3 | 12.5 | 12.6 | 12.9 | 10.7 | 12.2 |
| **42** | *M. superciliaris* | 13.6 | 19.2 | 12.6 | 13.3 | 14.0 | 12.1 | 14.3 | 13.8 | 14.0 | 12.3 | 13.8 | 13.5 | 14.5 | 11.2 | 13.6 | 11.2 | 10.9 | 12.1 | 11.5 | 13.5 | 13.6 | 11.5 | 12.8 | 11.3 | 12.9 | 13.5 | 14.0 | 10.9 | 10.9 |
| **43** | *M. palmipes* | 13.8 | 18.7 | 14.2 | 13.6 | 14.8 | 14.1 | 14.0 | 14.5 | 13.6 | 14.1 | 14.1 | 13.1 | 14.3 | 12.5 | 13.3 | 10.9 | 10.5 | 11.2 | 12.1 | 13.2 | 12.9 | 11.0 | 13.7 | 11.9 | 14.7 | 13.0 | 14.0 | 10.9 | 12.3 |
| **44** | *M. petrigena* | 13.7 | 17.1 | 14.3 | 13.9 | 14.6 | 13.5 | 13.5 | 14.0 | 13.9 | 12.4 | 14.2 | 13.8 | 15.5 | 13.2 | 14.5 | 12.7 | 12.6 | 13.2 | 13.1 | 14.5 | 14.3 | 12.2 | 13.6 | 12.4 | 12.6 | 13.8 | 14.5 | 12.5 | 13.3 |
| **45** | *M. perparva* | 13.5 | 16.1 | 13.7 | 13.6 | 15.0 | 14.4 | 13.7 | 14.9 | 14.1 | 13.2 | 14.6 | 14.1 | 15.4 | 13.2 | 14.1 | 12.2 | 11.7 | 12.6 | 12.1 | 14.3 | 13.7 | 12.3 | 13.8 | 12.5 | 14.2 | 14.0 | 14.9 | 13.2 | 13.7 |
| **46** | *M. annectens* | 13.2 | 16.2 | 13.6 | 13.7 | 14.8 | 13.6 | 14.0 | 14.0 | 14.6 | 13.0 | 14.0 | 13.2 | 15.1 | 13.0 | 13.5 | 13.3 | 12.7 | 14.1 | 13.6 | 15.1 | 15.2 | 13.0 | 14.3 | 13.1 | 11.3 | 13.8 | 14.4 | 13.0 | 14.4 |
| **47** | *M. annamensis* | 13.5 | 16.8 | 14.8 | 14.3 | 14.2 | 13.6 | 14.0 | 14.0 | 14.3 | 12.2 | 13.8 | 13.6 | 14.4 | 12.8 | 14.3 | 12.7 | 12.6 | 13.7 | 13.0 | 13.6 | 13.6 | 11.7 | 12.5 | 12.4 | 13.8 | 14.1 | 14.5 | 12.5 | 14.7 |
| **48** | *M. marmorata* | 11.4 | 15.1 | 12.4 | 11.5 | 13.3 | 12.7 | 11.7 | 12.9 | 12.8 | 13.2 | 13.5 | 12.9 | 13.8 | 12.5 | 13.5 | 12.3 | 11.9 | 12.7 | 12.2 | 13.0 | 13.4 | 11.8 | 12.4 | 12.3 | 12.9 | 13.4 | 14.7 | 11.6 | 12.9 |
| **49** | *M. pulverata* | 11.2 | 14.7 | 12.1 | 11.2 | 13.0 | 12.6 | 11.4 | 12.9 | 12.6 | 12.9 | 13.2 | 12.7 | 13.5 | 12.3 | 13.5 | 12.3 | 11.9 | 12.7 | 12.2 | 12.9 | 13.5 | 11.8 | 12.3 | 12.3 | 12.8 | 13.3 | 14.6 | 11.4 | 12.7 |
| **50** | *M. nanapollexa* | 14.0 | 17.7 | 13.6 | 13.3 | 13.2 | 13.5 | 13.8 | 13.3 | 13.0 | 12.7 | 13.3 | 13.3 | 14.8 | 14.9 | 11.8 | 13.9 | 14.2 | 14.2 | 15.0 | 15.1 | 14.4 | 12.9 | 14.7 | 13.8 | 12.2 | 13.3 | 14.6 | 13.3 | 13.7 |
| **51** | *M. arboricola* | 12.5 | 16.5 | 12.5 | 12.2 | 13.7 | 12.6 | 12.8 | 12.8 | 12.6 | 12.6 | 13.3 | 12.8 | 13.9 | 12.9 | 14.5 | 12.0 | 12.1 | 12.2 | 11.8 | 13.7 | 13.0 | 11.2 | 13.0 | 12.1 | 12.2 | 11.6 | 12.6 | 10.9 | 13.0 |
| **52** | *M. pulchella* | 11.5 | 15.2 | 12.2 | 11.6 | 13.1 | 11.5 | 12.1 | 11.9 | 11.7 | 12.1 | 11.5 | 11.3 | 13.5 | 12.0 | 12.8 | 11.2 | 11.5 | 11.8 | 11.2 | 12.7 | 11.9 | 10.2 | 11.3 | 10.8 | 11.4 | 11.1 | 12.8 | 10.0 | 12.3 |
| **53** | *Glyphoglossus yunnanensis* | 12.2 | 17.1 | 11.6 | 12.2 | 11.7 | 11.2 | 12.7 | 11.1 | 11.2 | 11.2 | 12.7 | 12.2 | 13.5 | 11.9 | 12.2 | 10.4 | 10.4 | 12.0 | 10.3 | 11.8 | 11.4 | 11.1 | 11.2 | 10.9 | 10.5 | 10.9 | 11.8 | 10.0 | 10.7 |
| **54** | *Glyphoglossus guttulatus* | 15.8 | 22.7 | 15.5 | 15.4 | 14.9 | 15.3 | 16.0 | 15.3 | 15.6 | 15.8 | 15.0 | 15.2 | 16.0 | 15.6 | 14.8 | 14.4 | 13.8 | 14.2 | 14.5 | 13.6 | 13.5 | 12.3 | 13.9 | 13.2 | 15.6 | 13.2 | 16.3 | 14.5 | 15.4 |
| **55** | *Glyphoglossus minutus* | 13.8 | 19.1 | 13.6 | 13.0 | 12.2 | 12.4 | 12.2 | 12.9 | 13.8 | 12.8 | 13.0 | 13.0 | 14.0 | 13.4 | 12.2 | 11.7 | 11.7 | 12.8 | 12.5 | 12.3 | 11.6 | 10.8 | 12.0 | 10.8 | 12.2 | 11.7 | 12.9 | 11.4 | 11.5 |
| **56** | *Glyphoglossus molossus* | 14.5 | 19.4 | 14.3 | 14.0 | 13.4 | 13.0 | 14.7 | 13.3 | 14.1 | 12.9 | 14.2 | 14.0 | 14.4 | 13.6 | 12.9 | 12.4 | 11.9 | 13.2 | 12.1 | 13.0 | 12.4 | 11.8 | 12.4 | 12.1 | 13.0 | 12.5 | 13.3 | 11.7 | 13.2 |
| **57** | *Glyphoglossus capsus* | 15.3 | 19.5 | 16.2 | 16.6 | 15.3 | 15.2 | 15.8 | 16.4 | 15.9 | 15.8 | 16.6 | 17.3 | 18.1 | 16.7 | 16.1 | 16.0 | 16.2 | 17.2 | 17.0 | 16.9 | 16.0 | 15.7 | 16.9 | 15.7 | 16.1 | 15.9 | 17.0 | 14.9 | 16.2 |

**Supplementary Table S9. Genetic divergence of the *Microhyla – Glyphoglossus* assemblage (continued).**

|  | **Species** | **30** | **31** | **32** | **33** | **34** | **35** | **36** | **37** | **38** | **39** | **40** | **41** | **42** | **43** | **44** | **45** | **46** | **47** | **48** | **49** | **50** | **51** | **52** | **53** | **54** | **55** | **56** | **57** |
| --- | --- | --- | --- | --- | --- | --- | --- | --- | --- | --- | --- | --- | --- | --- | --- | --- | --- | --- | --- | --- | --- | --- | --- | --- | --- | --- | --- | --- | --- |
| **30** | *M. ornata* | **0.3** |  |  |  |  |  |  |  |  |  |  |  |  |  |  |  |  |  |  |  |  |  |  |  |  |  |  |  |
| **31** | *M. rubra* | 9.5 | **0.5** |  |  |  |  |  |  |  |  |  |  |  |  |  |  |  |  |  |  |  |  |  |  |  |  |  |  |
| **32** | *M. mihintalei* | 9.7 | 3.4 | **0.2** |  |  |  |  |  |  |  |  |  |  |  |  |  |  |  |  |  |  |  |  |  |  |  |  |  |
| **33** | *M. aurantiventris* | 10.9 | 12.7 | 11.8 | **0.0** |  |  |  |  |  |  |  |  |  |  |  |  |  |  |  |  |  |  |  |  |  |  |  |  |
| **34** | *M. butleri* | 13.3 | 11.2 | 12.0 | 8.4 | **2.2** |  |  |  |  |  |  |  |  |  |  |  |  |  |  |  |  |  |  |  |  |  |  |  |
| **35** | *M. laterite* | 13.8 | 14.0 | 14.0 | 9.9 | 13.5 | **0.0** |  |  |  |  |  |  |  |  |  |  |  |  |  |  |  |  |  |  |  |  |  |  |
| **36** | *M. sholigari* | 12.4 | 12.2 | 12.8 | 11.2 | 12.8 | 5.6 | **0.0** |  |  |  |  |  |  |  |  |  |  |  |  |  |  |  |  |  |  |  |  |  |
| **37** | *M. darreli* | 11.2 | 11.4 | 11.8 | 10.2 | 12.5 | 5.1 | 4.1 | **NA** |  |  |  |  |  |  |  |  |  |  |  |  |  |  |  |  |  |  |  |  |
| **38** | *M. zeylanica* | 14.0 | 14.5 | 14.6 | 12.0 | 14.3 | 4.8 | 6.9 | 6.3 | **0.0** |  |  |  |  |  |  |  |  |  |  |  |  |  |  |  |  |  |  |  |
| **39** | *M. karunaratnei* | 12.1 | 13.3 | 13.2 | 9.5 | 11.9 | 5.5 | 6.0 | 3.4 | 6.7 | **0.3** |  |  |  |  |  |  |  |  |  |  |  |  |  |  |  |  |  |  |
| **40** | *M. eos* | 11.3 | 10.9 | 11.0 | 10.2 | 11.4 | 8.6 | 9.4 | 8.6 | 9.6 | 9.0 | **NA** |  |  |  |  |  |  |  |  |  |  |  |  |  |  |  |  |  |
| **41** | *Microhyla* sp. 2 | 10.3 | 11.1 | 11.0 | 9.2 | 10.3 | 9.9 | 9.9 | 9.3 | 11.0 | 9.1 | 8.9 | **0.6** |  |  |  |  |  |  |  |  |  |  |  |  |  |  |  |  |
| **42** | *M. superciliaris* | 11.4 | 10.6 | 9.9 | 10.1 | 9.8 | 11.1 | 11.1 | 10.3 | 12.2 | 9.6 | 7.9 | 7.1 | **1.6** |  |  |  |  |  |  |  |  |  |  |  |  |  |  |  |
| **43** | *M. palmipes* | 10.9 | 12.1 | 12.4 | 12.1 | 12.7 | 14.7 | 14.1 | 14.0 | 15.4 | 13.7 | 12.4 | 11.4 | 11.0 | **3.6** |  |  |  |  |  |  |  |  |  |  |  |  |  |  |
| **44** | *M. petrigena* | 13.3 | 12.7 | 12.4 | 12.7 | 13.3 | 15.5 | 14.3 | 14.2 | 15.9 | 14.2 | 13.1 | 12.3 | 11.5 | 11.9 | **3.7** |  |  |  |  |  |  |  |  |  |  |  |  |  |
| **45** | *M. perparva* | 13.3 | 14.1 | 13.8 | 11.8 | 13.2 | 14.6 | 14.7 | 14.0 | 16.4 | 13.7 | 12.6 | 11.8 | 12.3 | 11.7 | 7.1 | **5.1** |  |  |  |  |  |  |  |  |  |  |  |  |
| **46** | *M. annectens* | 13.6 | 13.2 | 14.2 | 11.8 | 12.2 | 15.2 | 13.7 | 14.2 | 15.7 | 14.1 | 11.0 | 12.3 | 13.0 | 12.9 | 8.1 | 8.0 | **0.4** |  |  |  |  |  |  |  |  |  |  |  |
| **47** | *M. annamensis* | 13.2 | 13.1 | 13.7 | 12.3 | 13.1 | 14.3 | 14.8 | 13.8 | 15.6 | 12.9 | 12.7 | 12.1 | 12.6 | 14.3 | 9.3 | 8.8 | 9.0 | **1.8** |  |  |  |  |  |  |  |  |  |  |
| **48** | *M. marmorata* | 12.0 | 12.6 | 13.7 | 12.6 | 11.8 | 13.7 | 13.9 | 13.4 | 13.9 | 12.8 | 11.1 | 10.3 | 12.3 | 12.4 | 8.5 | 7.9 | 6.3 | 6.5 | **0.7** |  |  |  |  |  |  |  |  |  |
| **49** | *M. pulverata* | 12.0 | 12.4 | 13.5 | 12.5 | 11.5 | 13.5 | 13.7 | 13.2 | 13.7 | 12.6 | 10.9 | 10.2 | 12.2 | 12.4 | 8.4 | 7.6 | 6.2 | 6.3 | 0.4 | **0.0** |  |  |  |  |  |  |  |  |
| **50** | *M. nanapollexa* | 14.6 | 13.5 | 13.3 | 12.7 | 13.5 | 14.7 | 13.7 | 15.2 | 14.7 | 14.1 | 12.7 | 13.7 | 13.7 | 14.1 | 9.5 | 10.7 | 10.5 | 11.4 | 11.2 | 10.9 | **0.8** |  |  |  |  |  |  |  |
| **51** | *M. arboricola* | 12.0 | 12.4 | 12.1 | 11.4 | 11.7 | 14.8 | 13.9 | 13.6 | 15.2 | 14.1 | 11.4 | 11.3 | 12.3 | 13.1 | 8.6 | 8.4 | 8.0 | 10.3 | 9.1 | 8.8 | 9.6 | **2.6** |  |  |  |  |  |  |
| **52** | *M. pulchella* | 11.6 | 11.8 | 12.1 | 11.1 | 11.4 | 14.3 | 13.3 | 13.1 | 15.1 | 13.7 | 10.5 | 11.5 | 11.5 | 12.6 | 8.4 | 8.5 | 6.9 | 9.8 | 8.9 | 8.8 | 9.1 | 3.0 | **0.3** |  |  |  |  |  |
| **53** | *Glyphoglossus yunnanensis* | 11.3 | 11.5 | 11.8 | 12.2 | 12.4 | 13.7 | 13.0 | 13.5 | 14.2 | 13.6 | 11.7 | 12.3 | 12.5 | 12.7 | 11.1 | 11.1 | 10.3 | 10.6 | 9.4 | 9.2 | 12.0 | 9.7 | 9.3 | **NA** |  |  |  |  |
| **54** | *Glyphoglossus guttulatus* | 15.4 | 16.4 | 15.2 | 13.7 | 15.2 | 15.5 | 14.6 | 14.7 | 15.8 | 14.1 | 14.9 | 14.6 | 14.9 | 15.9 | 12.3 | 13.4 | 15.8 | 13.1 | 13.5 | 13.4 | 12.7 | 12.2 | 12.4 | 11.1 | **1.7** |  |  |  |
| **55** | *Glyphoglossus minutus* | 12.2 | 12.8 | 12.1 | 12.5 | 13.4 | 14.8 | 12.2 | 12.8 | 14.8 | 12.4 | 13.0 | 12.2 | 12.5 | 13.5 | 10.4 | 12.8 | 12.6 | 11.5 | 10.7 | 10.5 | 10.7 | 10.7 | 10.1 | 6.4 | 7.0 | **NA** |  |  |
| **56** | *Glyphoglossus molossus* | 12.4 | 15.2 | 15.1 | 11.5 | 12.5 | 14.2 | 13.5 | 13.2 | 15.2 | 13.1 | 11.2 | 13.4 | 13.1 | 13.6 | 13.5 | 11.8 | 11.4 | 12.7 | 11.5 | 11.4 | 14.2 | 11.5 | 10.5 | 6.9 | 10.6 | 7.7 | **NA** |  |
| **57** | *Glyphoglossus capsus* | 15.1 | 14.9 | 14.6 | 15.9 | 16.9 | 15.7 | 16.5 | 15.9 | 17.5 | 16.1 | 13.5 | 16.0 | 15.9 | 17.0 | 14.9 | 15.6 | 15.7 | 14.0 | 14.5 | 14.4 | 15.2 | 14.2 | 13.3 | 11.9 | 15.0 | 12.9 | 13.5 | **0.6** |
