# Supplementary material for: A little frog leaps a long way: compounded colonizations of the Indian Subcontinent discovered in the tiny Oriental frog genus Microhyla (Amphibia: Microhylidae)
Source: PeerJ. 2020 Jul 3;8:e9411. doi: 10.7717/peerj.9411 (PMC7337035; doi:10.7717/peerj.9411)
Supplement: Supplemental Information 14 — Number of species-level groups recovered by bGMYC and ABGD analyses presented for each of the morphospecies within Microhyla sensu lato (1–52). [file peerj-08-9411-s014.docx]

**Supplementary Table S10. Results of species delimitation analyses of *Microhyla*.**

Number of species-level groups recovered by bGMYC and ABGD analyses presented for each of the morphospecies within *Microhyla* sensu lato (1–52).

| **No.** | **Species** | **Number of samples** | **Number of groups** | |
| --- | --- | --- | --- | --- |
|  |  |  | **bGMYC** | **ABGD** |
| 1 | *Microhyla achatina* | 6 | 2 | 2 |
| 2 | *Microhyla annamensis* | 2 | 1 | 1 |
| 3 | *Microhyla annectens* | 4 | 1 | 1 |
| 4 | *Microhyla arboricola* | 4 | 2 | 2 |
| 5 | *Microhyla aurantiventris* | 2 | 1 | 1 |
| 6 | *Microhyla beilunensis* | 2 | 2 | 0 |
| 7 | *Microhyla bermodrei* | 17 | 4 | 3 |
| 8 | *Microhyla borneensis* | 1 | 1 | 0 |
| 9 | *Microhyla butleri* | 11 | 4 | 2 |
| 10 | *Microhyla chakrapanii* | 2 | 2 | 1 |
| 11 | *Microhyla darreli* | 1 | 1 | 0 |
| 12 | *Microhyla eos* | 1 | 1 | 1 |
| 13 | *Microhyla fanjingshanensis* | 1 | 1 | 1 |
| 14 | *Microhyla fissipes* | 4 | 1 | 1 |
| 15 | *Microhyla fodiens* | 3 | 1 | 1 |
| 16 | *Microhyla gadjahmadai* | 2 | 2 | 2 |
| 17 | *Microhyla heymonsi* | 14 | 8 | 7 |
| 18 | *Microhyla irrawaddy* | 4 | 1 | 1 |
| 19 | *Microhyla karunaratnei* | 2 | 1 | 1 |
| 20 | *Microhyla kodial* | 2 | 1 | 1 |
| 21 | *Microhyla laterite* | 2 | 1 | 1 |
| 22 | *Microhyla malang* | 5 | 3 | 3 |
| 23 | *Microhyla mantheyi* | 5 | 2 | 1 |
| 24 | *Microhyla marmorata* | 6 | 1 | 1 |
| 25 | *Microhyla mihintalei* | 3 | 1 | 1 |
| 26 | *Microhyla minuta* | 3 | 1 | 1 |
| 27 | *Microhyla mixtura* | 3 | 2 | 1 |
| 28 | *Microhyla mukhlesuri* | 11 | 3 | 0 |
| 29 | *Microhyla mymensinghensis* | 4 | 1 | 1 |
| 30 | *Microhyla nanapollexa* | 2 | 1 | 1 |
| 31 | *Microhyla nepenthicola* | 4 | 1 | 1 |
| 32 | *Microhyla nilphamariensis* | 6 | 1 | 1 |
| 33 | *Microhyla okinavensis* | 3 | 2 | 2 |
| 34 | *Microhyla orientalis* | 3 | 2 | 1 |
| 35 | *Microhyla ornata* | 3 | 1 | 1 |
| 36 | *Microhyla palmipes* | 2 | 2 | 2 |
| 37 | *Microhyla perparva* | 2 | 2 | 2 |
| 38 | *Microhyla petrigena* | 3 | 2 | 2 |
| 39 | *Microhyla picta* | 2 | 1 | 1 |
| 40 | *Microhyla pineticola* | 4 | 1 | 1 |
| 41 | *Microhyla pulchella* | 4 | 1 | 1 |
| 42 | *Microhyla pulchra* | 6 | 1 | 1 |
| 43 | *Microhyla pulverata* | 2 | 0 | 0 |
| 44 | *Microhyla rubra* | 3 | 1 | 1 |
| 45 | *Microhyla sholigari* | 3 | 1 | 1 |
| 46 | *Microhyla superciliaris* | 4 | 2 | 1 |
| 47 | *Microhyla taraiensis* | 1 | 1 | 1 |
| 48 | *Microhyla zeylanica* | 2 | 1 | 1 |
| 49 | *Microhyla* sp. 1 | 2 | 1 | 1 |
| 50 | *Microhyla* sp. 2 | 4 | 1 | 1 |
| 51 | *Microhyla* sp. 3 | 5 | 1 | 0 |
| 52 | *Microhyla* sp. 4 | 2 | 1 | 0 |
|  | **Sum Total:** | **199** | **81** | **63** |
