# Supplementary material for: A little frog leaps a long way: compounded colonizations of the Indian Subcontinent discovered in the tiny Oriental frog genus Microhyla (Amphibia: Microhylidae)
Source: PeerJ. 2020 Jul 3;8:e9411. doi: 10.7717/peerj.9411 (PMC7337035; doi:10.7717/peerj.9411)
Supplement: Supplemental Information 15 — Node No. – estimated tree node, for node names see Fig. S3; divergence time given in millions years (Ma). [file peerj-08-9411-s015.docx]

**Supplementary Table S11. Results of divergence time estimates.**

Node No. – estimated tree node, for node names see Fig. S3; divergence time given in millions years (Ma).

| **Node No.** | **Estimated age (Ma)** | |
| --- | --- | --- |
|  | **mean** | **95% CI range** |
| **1** | 100.21 | [81.17 – 128.29] |
| **2** | 5.42 | [5 – 6.58] |
| **3** | 91.38 | [80.31 – 103.55] |
| **4** | 9.44 | [5.91 – 12.84] |
| **5** | 69.77 | [65.47 – 74.94] |
| **6** | 7.67 | [4.81 – 11.08] |
| **7** | 65.85 | [65 – 68.62] |
| **8** | 54.04 | [46.71 – 61.49] |
| **9** | 46.72 | [38.56 – 54.97] |
| **10** | 33.84 | [27.31 – 40.82] |
| **11** | 50.81 | [44.12 – 57.04] |
| **12** | 43.84 | [38.74 – 49.11] |
| **13** | 29.41 | [22.38 – 37.43] |
| **14** | 20.22 | [15.42 – 25.16] |
| **15** | 17.31 | [13.01 – 21.96] |
| **16** | 13.05 | [9.26 – 17.67] |
| **17** | 42.62 | [37.79 – 47.96] |
| **18** | 26.59 | [22.27 – 31.09] |
| **19** | 22.67 | [18.38 – 27.56] |
| **20** | 2.76 | [1.35 – 4.51] |
| **21** | 9.53 | [6.95 – 12.44] |
| **22** | 6.36 | [4.35 – 8.86] |
| **23** | 23.03 | [19.01 – 27.21] |
| **24** | 14.35 | [10.39 – 18.39] |
| **25** | 2.07 | [1.09 – 3.28] |
| **26** | 21.58 | [17.42 – 25.69] |
| **27** | 15.21 | [11.88 – 19.16] |
| **28** | 8.65 | [5.68 – 11.78] |
| **29** | 9.77 | [6.78 – 13.13] |
| **30** | 32.54 | [29.24 – 36.36= |
| **31** | 30.71 | [27.03 – 34.51] |
| **32** | 6.09 | [3.72 – 8.99] |
| **33** | 29.2 | [25.69 – 33.13 |
| **34** | 19.5 | [15.7 – 23.67 |
| **35** | 8.1 | [6.11 – 10.14 |
| **36** | 5.56 | [4.16 – 6.98] |
| **37** | 22.52 | [18.96 – 26.47] |
| **38** | 19.13 | [15.61 – 22.87] |
| **39** | 3.25 | [1.92 – 4.92] |
| **40** | 1.61 | [0.9 – 2.493] |
| **41** | 19.02 | [15 – 23.47] |
| **42** | 8.55 | [6.46 – 10.84] |
| **43** | 6.6 | [3.67 – 9.3] |
| **44** | 7.15 | [5.19 – 9.32] |
| **45** | 5.52 | [3.52 – 7.73] |
| **46** | 31.37 | [28.02 – 34.92] |
| **47** | 16.82 | [15.17 – 22.75] |
| **48** | 6.91 | [4.55 – 9.6] |
| **49** | 12.03 | [9.04 – 15.17] |
| **50** | 1.42 | [0.72 – 2.37] |
| **51** | 9.94 | [7.14 – 13.16] |
| **52** | 28.55 | [25.21 – 31.84 |
| **53** | 19.85 | [16.31 – 23.69] |
| **54** | 16.07 | [12.31 – 19.89] |
| **55** | 6.86 | [5.35 – 8.5] |
| **56** | 5.69 | [4.28 – 7.17] |
| **57** | 24.28 | [21.65 – 27.28] |
| **58** | 16.36 | [13.48 – 19.57] |
| **59** | 10.31 | [8.14 – 12.62] |
| **60** | 8.43 | [6.35 – 10.57] |
| **61** | 4.91 | [3.38 – 6.8] |
| **62** | 7.63 | [5.51 – 10.15] |
| **63** | 4.07 | [2.32 – 6.08] |
| **64** | 0.87 | [0.44 – 1.45] |
| **65** | 7.23 | [5.79 – 8.73] |
| **66** | 5.1 | [3.87 – 6.49] |
| **67** | 1.93 | [1.134 – 2.94] |
| **68** | 1.68 | [0.83 – 2.66] |
| **69** | 5.33 | [4.61 – 7.37] |
| **70** | 0.62 | [0.25 – 1.07] |
| **71** | 3.81 | [2.76 – 4.55] |
| **72** | 2.83 | [1.9 – 3.81] |
| **73** | 3.03 | [2.31 – 39] |
| **74** | 22.87 | [20.25 – 25.75] |
| **75** | 21.84 | [19.27 – 24.61] |
| **76** | 20.41 | [17.74 – 23.46] |
| **77** | 8.65 | [7.11 – 10.56] |
| **78** | 6.84 | [5.23 – 8.56] |
| **79** | 7.77 | [6.13 – 9.6] |
| **80** | 18.31 | [15.85 – 20.88] |
| **81** | 16.15 | [13.58 – 18.85] |
| **82** | 8.07 | [5.9 – 10.5] |
| **83** | 3.64 | [2.3 – 5.29] |
| **84** | 8.94 | [5.86 – 12.19] |
| **85** | 3.32 | [1.52 – 5.57] |
| **86** | 16.26 | [13.8 – 18.82] |
| **87** | 15.1 | [12.86 – 17.6] |
| **88** | 13.3 | [10.74 – 15.81] |
| **89** | 3.16 | [1.79 – 4.77] |
| **90** | 1.43 | [0.68 – 2.36] |
| **91** | 8.83 | [6.81 – 10.76] |
| **92** | 3.92 | [2.53 – 5.59] |
| **93** | 4.88 | [3.48 – 6.4] |
| **94** | 3.93 | [2.54 – 5.48] |
