# Supplementary material for: A little frog leaps a long way: compounded colonizations of the Indian Subcontinent discovered in the tiny Oriental frog genus Microhyla (Amphibia: Microhylidae)
Source: PeerJ. 2020 Jul 3;8:e9411. doi: 10.7717/peerj.9411 (PMC7337035; doi:10.7717/peerj.9411)
Supplement: Supplemental Information 16 [file peerj-08-9411-s016.pdf]

<u>**Supplementary Information File 1.**</u>

Biogeographic area definition for South, Southeast and East Asia.

In the present paper follow the number of recent major works on biodiversity and biogeography of South, Southeast Asia and East Asia (*Myers et al., 2000*; *Averyanov et al., 2003*; *De Bruyn et al., 2014*) and on biogeographic analysis of Southeast Asian herpetofauna (*Bain & Hurley, 2011*). In total, within the modern range of the genus *Microhyla* we defined nine regions for the biogeographic analyses:

A) **Mainland East Asia** – territories of central, eastern and southern mainland China (including the island of Hainan) and northern Vietnam to the north of the Red River. The Red River forms an important biogeographic barrier for both flora and fauna, separating predominantly tropical areas to the south from predominantly subtropical areas to the north of its valley (*Delacour et al., 1928*; *MacKinnon, 1997*; *Geissman et al., 2000*; *Orlov et al., 2001*; *Averyanov et al., 2003*). The island of Hainan has no endemic Microhyla lineages and its fauna is fundamentally similar to southern China and north-western Vietnam (*Zhao & Adler, 1993*).

B) **Eastern Indochina** – territories of the Annamite (Truong Son) mountains and surrounding lowlands in Vietnam, Laos and easternmost Cambodia, southwards of the Red River valley but to the east of Mekong River. The Annamite Mountain Range is known for its endemism (*Stattersfield et al., 1998*; *Baltzer et al., 2001*; *Sterling et al., 2006*). The Mekong River is the longest basin in Southeast Asia and forms an important biogeographic barrier to fauna and separates Eastern and Western Indochina (*Fooden, 1996*; *Meijaard & Groves, 2006*; *Giessler et al., 2015*).

C) **Western Indochina –** lowland areas of western and central Cambodia, western Laos, and southern Vietnam westwards from the Mekong River Valley, Thailand as far south as the Isthmus of Kra, Myanmar as far westwards as the Arakan (Rakhine) Moutains (formally interpreted as the country border between Myanmar, Bangladesh and India), including the Andaman and Nicobar Island Arc (a continuation of the Arakan Mountain Range). Arakan Mountains represent an important barrier between the Assam and Irrawaddy basins (*Akhtar et al., 2009*). Herpetofauna of the Andaman and Nicobar islands is closer to herpetofauna of western Indochina than to the mainland India (*Das, 1994*). The Isthmus of Kra is widely known as a biogeographic border separating the Sundaland from the mainland Southeast Asia (*Pauwels et al., 2003*; *De Bruyn et al., 2005*).

D) **Indian subcontinent –** territories of mainland India including Assam, as well as lowland areas of Pakistan, Nepal, Bhutan and Bangladesh. These territories are known for hosting a diverse biota with several local biodiversity hotspots (*Mani, 1995*; *Myers et al., 2000*).

E) **Malayan Peninsula** – represents the mainland part of the Sundaland encompassing southern peninsular Thailand to the south of the Isthmus of Kra and the mainland part of Malaysia. These territories host unique and diverse herpetofauna, combining both autochthonous and Sundanese elements (*Grismer, 2008*; *Grismer et al., 2017*, *2018*).

F) **Sumatra – Java – Bali** – encompasses an island arc in the western edge of the Sundaland, with adjacent smaller offshore islands. These territories were numerous times connected with Borneo and the Malayan Peninsula during sea-level transgressions and re-isolated during inundation periods (*Voris et al., 2000*; *Woodruff et al., 2010*; *De Bruyn et al., 2014*).

G) **Borneo and Philippines** – this area includes the island of Borneo (Kalimantan) which is known as one of the major centers of biodiversity in Southeast Asia (*De Bruyn et al., 2014*). *Microhyla* are known to occur on small Sulu Islands belonging to the Philippines (*Diesmos et al., 2002*), which are adjacent to Borneo, so we include the Sulu Archipelago to this biogeographic region.

H) **Sri Lanka** – the island of Sri Lanka is known as a major biodiversity hotspot with biogeographic affinities to the Western Ghats of southern India (see *Myers et al., 2000*; *Agarwal et al., 2017*, and references therein).

I) **East Asian islands** – this regions joins the island of Taiwan and the Ryukyu Islands of Japan, the latter include several archipelagos (Okinawa, Amami, Yaeyama). Biogeography of this region was discussed in a number of works (see *Ota, 1998*, and references therein).

For references cited here see Supplementary Information File 2.
