# Supplementary material for: A little frog leaps a long way: compounded colonizations of the Indian Subcontinent discovered in the tiny Oriental frog genus Microhyla (Amphibia: Microhylidae)
Source: PeerJ. 2020 Jul 3;8:e9411. doi: 10.7717/peerj.9411 (PMC7337035; doi:10.7717/peerj.9411)
Supplement: Supplemental Information 17 [file peerj-08-9411-s017.pdf]

Supplementary Information File 2.

Additional references in Supplementary tables and Supplementary Information File 1.

Agarwal I, Biswas S, Bauer AM, Greenbaum E, Jackman TR, de Silva A, Batuwita S. 2017. Cryptic species, taxonomic inflation, or a bit of both? New species phenomenon in Sri Lanka as suggested by a phylogeny of dwarf geckos (Reptilia, Squamata, Gekkonidae, *Cnemaspis*). *Systematics and Biodiversity* **15**:1–13.

Akhtar SM, Chakrabarti S, Singh RK, Moulik SR, Bhattacharya J, Singh H. 2009. Structural style and deformation history of Assam & Assam Arakan Basin, India: from Integrated Seismic Study. In: *Adapted from oral presentation at AAPG Annual Convention*, Denver, Colorado; 10:2009.

Andersson LG. 1942. A small collection of frogs from Annam collected in the years 1938–1939 by Bertil Björkegren. *Arkiv för Zoologi* **34(6)**: 1–11.

Atmaja VY, Hamidy A, Arisuryanti T, Matsui M, Smith EN. 2018. A new species of *Microhyla* (Anura: Microhylidae) from Sumatra, Indonesia. *Treubia* **45**: 25–46.

Averyanov L, Cribb P, Phan KL, Nguyen TH. 2003. *Slipper orchids of Vietnam with an introduction to the flora of Vietnam*. Portland: Timber Press, 83 pp.

Bain RH, Hurley MM. 2011. A biogeographic synthesis of the amphibians and reptiles of Indochina. *Bulletin of the American Museum of Natural History* **2011(360)**:1–38.

Bain RH, Nguyen QT. 2004. Three new species of narrow-mouth frogs (Genus: *Microhyla*) from Indochina, with comments on *Microhyla annamensis* and *Microhyla palmipes***.** *Copeia* **2004(3)**:507–524.

Baltzer M, Dao NT, Shore RG. 2001. *Towards a vision of biodiversity conservation in the forests for the lower Mekong ecoregion complex*. Vol. 1: Main Report. Hanoi: WWF Indochina, 109 pp.

Biju SD, Garg S, Kamei RG, Maheswaran G. 2019. A new *Microhyla* species (Anura: Microhylidae) from riparian evergreen forest in the eastern Himalayan state of Arunachal Pradesh, India. *Zootaxa* **4674**:100–116.

Blackburn DC, Siler CD, Diesmos AC, McGuire JA, Cannatella DC, Brown RM. 2013. An adaptive radiation of frogs in a Southeast Asian island archipelago. *Evolution* **67(9):** 2631–2646.

Das I, Haas A. 2010. New species of *Microhyla* from Sarawak: Old World's smallest frogs crawl out of miniature pitcher plants on Borneo (Amphibia: Anura: Microhylidae). *Zootaxa* **2571**:37–52.

Das I, Min PY, Hsu WW, Hertwig ST, Haas A. 2014. Red hot chili pepper. A new *Calluella* Stoliczka, 1872 (Lissamphibia: Anura: Microhylidae) from Sarawak, East Malaysia (Borneo). *Zootaxa* **3785(4)**: 550–560.

Das I, Yaakob N, Liat LB. 2004. A new species of *Calluella* Stoliczka, 1872 (Anura: Microhylidae) from Taman Negara, Pahang State, Peninsular Malaysia. *Raffles Bulletin of Zoology* **52**: 257–260.

Das I, Yaakob NS, Sukumaran J. 2007. A new species of *Microhyla* (Anura: Microhylidae) from the Malay Peninsula. *Hamadryad* **31**: 304–314.

Das I. 1994. A check-list of the amphibians and reptiles of Andaman and Nicobar Islands. *Journal of the Andaman Science Association* **10(1)**:44-49.

De Bruyn M, Nugroho E, Hossain MM, Wilson JC, Mather PB. 2005. Phylogeographic evidence for the existence of an ancient biogeographic barrier: the Isthmus of Kra Seaway. *Heredity* **94**(3):370–378.

De Bruyn M, Stelbrink B, Morley RJ, Hall R, Carvalho GR, Cannon CH, Van den Bergh G, Meijaard E, Metcalfe I, Boitani L, Maiorano L, Shoup R, von Rintelen T. 2014. Borneo and Indochina are major evolutionary hotspots for Southeast Asian biodiversity. *Systematic Biology* **63**:879-901.

Delacour J, Jabouille P, Lowe WP. 1928. On the birds collected during the third expedition to French Indo-china. [Part I]. *Ibis*, Series 12 **4**:23–51.

Deng X, Wang S, Liang X, Jiang J, Wang B, Deng L. 2016. The complete mitochondrial genome of *Kaloula* rugifera (Amphibia, Anura, Microhylidae). *Mitochondrial DNA Part A* **27(5)**: 3391–3392.

Diesmos AC, Brown RM, Alcala AC, Sison RV, Afuang LE, Gee GV. 2002. Philippine amphibians and reptiles: an overview of species diversity, biogeography, and conservation. *Philippine biodiversity conservation priorities: a second iteration of the National Biodiversity Strategy and Action Plan*. 26–44 pp.

Dutta SK, Manamendra-Arachchi K. 1996. The amphibian fauna of Sri Lanka. Wildlife Heritage Trust of Sri Lanka.

Dutta SK, Ray P. 2000. *Microhyla sholigari*, a new species of microhylid frog (Anura: Microhylidae) from Karnataka, India. *Hamadryad* **25**: 38–44.

Fernando P, Siriwardhane M. 1996. *Microhyla karunaratnei* (Anura: Microhylidae), a new species of frog endemic to Sri Lanka. *Journal of South Asian Natural History* **2**: 135–142.

Fooden J. 1996. Zoogeography of Vietnamese primates. *International Journal of Primatology* **17**:845–899.

Fromhage L, Vences M, Veith M. 2004. Testing alternative vicariance scenarios in Western Mediterranean discoglossid frogs. *Molecular Phylogenetics and Evolution* **31(1)**:308–322.

Garg S, Suyesh R, Das A, Jiang J, Wijayathilaka N, Amarasinghe AT, Alhadi F, Vineeth KK, Aravind NA, Senevirathne G, Meegaskumbura M, Biju SD. 2019. Systematic revision of *Microhyla* (Microhylidae) frogs of South Asia: a molecular, morphological, and acoustic assessment. *Vertebrate Zoology* **69(1)**:1–71.

Geissler P, Hartmann T, Ihlow F, Rödder D, Poyarkov NA, Nguyen TQ, Ziegler T, Böhme W. 2015. The Lower Mekong: an insurmountable barrier to amphibians in southern Indochina?. *Biological Journal of the Linnean Society* **114**(4):905–914.

Geissmann T, Nguyen XD, Lormée N, Momberg F. 2000. *Vietnam primate conservation status review 2000*. Part 1. Hanoi: Fauna and Flora International, 130 pp.

Grismer LL, Wood Jr PL, Aowphol A, Cota M, Grismer MS, Murdoch ML, Aguilar C, Grismer JL. 2017. Out of Borneo, again and again: biogeography of the Stream Toad genus *Ansonia* Stoliczka (Anura: Bufonidae) and the discovery of the first limestone cave-dwelling species. *Biological Journal of the Linnean Society* **120**(2):371–395.

Grismer LL, Wood Jr PL, Quah ES, Anuar S, Ngadi EB, Izam NA, Ahmad N. 2018. Systematics, ecomorphology, cryptic speciation and biogeography of the lizard genus *Tytthoscincus* Linkem, Diesmos & Brown (Squamata: Scincidae) from the sky-island archipelago of Peninsular Malaysia. *Zoological Journal of the Linnean Society* **183**(3):635–671.

Grismer LL. 2008. A revised and updated checklist of the lizards of Peninsular Malaysia. *Zootaxa* **1860**(1):28–34.

Hasan M, Islam MM, Khan MM, Alam MS, Kurabayashi A, Igawa T, Kuramoto M, Sumida M. 2012. Cryptic anuran biodiversity in Bangladesh revealed by mitochondrial 16S rRNA gene sequences. *Zoological science* **29(3)**:162–172.

Hasan M, Razzaque MA, Sarker AK, Kuramoto M, Sumida M. 2015. Genetic variation, advertisement call, and morphometry of *Microhyla nilphamariensis* from Bangladesh. *Philippine Journal of Systematic Biology* **9:** 63–80.

Hasan MK, Islam MM, Kuramoto M, Kurabayashi A, Sumida M. 2014. Description of two new species of *Microhyla* (Anura: Microhylidae) from Bangladesh. *Zootaxa* **3755**:401–408.

Igawa T, Kurabayashi A, Usuki C, Fujii T, Sumida M. 2008. Complete mitochondrial genomes of three neobatrachian anurans: a case study of divergence time estimation using different data and calibration settings. *Gene* **407(1-2):** 116–129.

Inger RF, Frogner KJ. 1979. New species of narrow-mouth frogs (genus *Microhyla*) from Borneo. *Sarawak Museum Journal* **27**: 311–322.

Inger RF. 1989. Four new species of frogs from Borneo. *Malayan Nature Journal* **42:** 229–243.

Khatiwada JR, Shu GC, Wang SH, Thapa A, Wang B, Jiang J. 2017. A new species of the genus *Microhyla* (Anura: Microhylidae) from Eastern Nepal. *Zootaxa* **4254(2)**:221–239.

Khatiwada JR, Wang S, Shu G, Xie F, Jiang J. 2018. The mitochondrial genome of the *Microhyla taraiensis* (Anura: Microhylidae) and related phylogenetic analyses. *Conservation Genetics Resources* **10(3)**: 441–444.

Kuramoto M, Joshy SH. 2007. New frogs from the Western Ghats. *Current Science* **93(6):** 752–754.

Li S, Zhang M, Xu N, Lv J, Jiang J, Liu J, Wei G, Wang B. 2019. A new species of the genus *Microhyla* (Amphibia: Anura: Microhylidae) from Guizhou Province, China. *Zootaxa* **4624(4)**: 551–575.

MacKinnon J, MacKinnon K. 1986. *Review of the protected areas system in the Indo-Malayan realm*. Gland, Switzerland: IUCN, 284 pp.

Mani MS. 1995. *Biogeography in India.* Surya Publications, Dehra Dun, Uttaranchal, India, 1995.

Manthey U, Denzer W. 2014. Südostasiatische Anuren im Fokus: Spezies der Gattung *Microhyla* Tschudi, 1838 (Anura: Microhylidae: Microhylinae**).** *Sauria* **36(4)**: 31–48.

Manthey U, Grossmann W. 1997. Amphibien and Reptilien Südostasiens, Natur und Tier-Verlag, Münster.

Matsui M, Hamidy A, Belabut DM, Ahmad N, Panha S, Sudin A, Khonsue W, Oh HS, Yong HS, Jiang JP, Nishikawa K. 2011. Systematic relationships of Oriental tiny frogs of the family Microhylidae (Amphibia, Anura) as revealed by mtDNA genealogy. *Molecular phylogenetics and evolution* **61(1)**:167–176.

Matsui M, Hamidy A, Eto K. 2013. Description of a new species of *Microhyla* from Bali, Indonesia (Amphibia, Anura). *Zootaxa* **3670(4)**:579–590.

Matsui M, Ito H, Shimada T, Ota H, Saidapur SK, Khonsue W, Tanaka-Ueno T, Wu GF. 2005. Taxonomic relationships within the pan-oriental narrowmouth toad *Microhyla ornata* as revealed by mtDNA analysis (Amphibia, Anura, Microhylidae). *Zoological Science* **22(4)**:489–495.

Matsui M, Shimada T, Liu WZ, Maryati M, Khonsue W, Orlov N. 2006. Phylogenetic relationships of Oriental torrent frogs in the genus *Amolops* and its allies (Amphibia, Anura, Ranidae). *Molecular Phylogenetics and Evolution* **38(3)**:659–666.

Matsui M. 2011. Taxonomic revision of one of the Old World's smallest frogs, with description of a new Bornean *Microhyla* (Amphibia, Microhylidae). *Zootaxa* **2814(1)**:33–49.

Meijaard E, Groves CP. 2006. The geography of mammals and rivers in Mainland Southeast Asia. In **Lehman SM, Fleagle JG** (editors), *Primate biogeography: progress and prospects*, New York: Springer pp. 305–329.

Mulcahy DG, Lee JL, Miller AH, Chand M, Thura MK, Zug GR. 2018. Filling the BINs of life: Report of an amphibian and reptile survey of the Tanintharyi (Tenasserim) Region of Myanmar, with DNA barcode data. *ZooKeys* **757**:85–152.

Myers N, Mittermeier RA, Mittermeier CG, Da Fonseca GA, Kent J. 2000. Biodiversity hotspots for conservation priorities. *Nature* **403**(6772):853.

Neang T, Holden J. 2008. A field guide to the amphibians of Cambodia. Fauna & Flora International.

Nguyen LT, Poyarkov NA, Nguyen TT, Nguyen TA, Tran VH, Gorin VA, Murphy RW, Nguyen SN. 2019. A new species of the genus *Microhyla* Tschudi, 1838 (Amphibia: Anura: Microhylidae) from Tay Nguyen Plateau, Central Vietnam. *Zootaxa* **4543**:549–580.

Ngyuen SV, Ho CT, Ngyuen TQ. 2009. Herpetofauna of Vietnam. Edition Chimaira.

Orlov NL, Lathrop A, Murphy R, Ho TC. 2001. New records of frogs of the family Rhacophoridae (Anura: Amphibia) in the north part of the Hoang Lien Mountains (Mount Fan Si Pan, Sa Pa District, Lao Cai Province), Vietnam. *Russian Journal of Herpetology* **8**:17–44.

Ota H. 1998. Geographic patterns of endemism and speciation in amphibians and reptiles of the Ryukyu Archipelago, Japan, with special reference to their paleogeographic implications. *Researches on Population Ecology* **40**:189–204.

Palumbi, S. 1991. Simple fool's guide to PCR. Available at: *https://palumbilab.stanford.edu/SimpleFoolsMaster.pdf* (accessed January 15 2020).

Parker HW, Osman-Hill WC. 1949 "1948". Frogs of the genus *Microhyla* from Ceylon. *Annals and Magazine of Natural History* **12(1)**: 759–764.

Parker HW. 1928. The brevicipitid frogs of the genus *Microhyla*. *Annals and Magazine of Natural History*, Series 10 **2**:473–499.

Pauwels OS, David P, Chimsunchart C, Thirakhupt K. 2003. Reptiles of Phetchaburi Province, Western Thailand: a list of species, with natural history notes, and a discussion on the biogeography at the Isthmus of Kra. *Tropical Natural History* **3**(1):23–53.

Pauwels OSG, Ohler A, Dubois A., Nabhitabhata J, Laohawat OA, Chimsunchart C. 1999. *Kaloula baleata* (Müller, 1836) (Anura: Microhylidae), an addition to the batrachofauna of Thailand. *Natural History Bulletin of the Siam Society* **47(2)**: 261–264.

Peloso PL, Frost DR, Richards SJ, Rodrigues MT, Donnellan S, Matsui M, Raxworthy CJ, Biju SD, Lemmon EM, Lemmon AR, Wheeler WC. 2016. The impact of anchored phylogenomics and taxon sampling on phylogenetic inference in narrow-mouthed frogs (Anura, Microhylidae). *Cladistics* **32(2)**:113–140.

Pillai RS. 1977. On two frogs of the family Microhylidae from Andamans including a new species. *Proceedings of the Indian Academy of Sciences*. Section B **86**: 135–138.

Poyarkov NA, Gorin VA, Zaw T, Kretova VD, Gogoleva SI, Pawangkhanant P, Che J. 2019. On the road to Mandalay: contribution to the *Microhyla* Tschudi, 1838 (Amphibia: Anura: Microhylidae) fauna of Myanmar with description of two new species. *Zoological Research* **40(4)**:244–276 DOI 10.24272/j.issn.2095-8137.2019.044.

Poyarkov NA, Nguyen TV, Duong TV, Gorin VA, Yang JH. 2018. A new limestone-dwelling species of *Micryletta* (Amphibia: Anura: Microhylidae) from northern Vietnam. *PeerJ* **6**:e5771 DOI 10.7717/peerj.5771

Poyarkov NA, Vassilieva AB, Orlov NL, Galoyan EA, Tran DTA, Le DTT, Kretova VD, Geissler P. 2014. Taxonomy and distribution of narrow-mouth frogs of the genus *Microhyla* Tschudi, 1838 (Anura: Microhylidae) from Vietnam with descriptions of five new species. *Russian Journal of Herpetology* **21**:89–148.

Sano N, Kurabayashi A, Fujii T, Yonekawa H, Sumida M. 2005. Complete nucleotide sequence of the mitochondrial genome of Schlegel’s tree frog *Rhacophorus schlegelii* (family Rhacophoridae): duplicated control regions and gene rearrangements. *Genes & genetic systems* **80(3)**: 213–224.

Seshadri KS, Singal R, Priti H, Ravikanth G, Vidisha MK, Saurabh S, Pratik M, Gururaja KV. 2016. *Microhyla laterite* sp. nov., a new species of *Microhyla* Tschudi, 1838 (Amphibia: Anura: Microhylidae) from a laterite rock formation in South West India. *PloS one* **11(3)**:e0149727.

Stattersfield AJ, Crosby MJ, Long AJ, Wege DC. 1998. *Endemic bird areas of the world: priorities for biodiversity conservation*. Cambridge, UK: BirdLife International, 846 pp.

Sterling EJ, Hurley MM, Le DM. 2006. *Vietnam: a natural history*. New Haven, CT: Yale University Press, 448 pp.

Tominaga A, Matsui M, Shimoji N, Khonsue W, Wu CS, Toda M, Eto K, Nishikawa K, Ota H. 2019. Relict distribution of *Microhyla* (Amphibia: Microhylidae) in the Ryukyu Archipelago: High diversity in East Asia maintained by insularization. *Zoologica Scripta* **48(4)**:440–453.

Van der Meijden A, Vences M, Hoegg S, Boistel R, Channing A, Meyer A. 2007. Nuclear gene phylogeny of narrow-mouthed toads (Family: Microhylidae) and a discussion of competing hypotheses concerning their biogeographical origins. *Molecular Phylogenetics and Evolution* **44**:1017–1030.

Vences M, Vieites DR, Glaw F, Brinkmann H, Kosuch J, Veith M, Meyer A. 2003. Multiple overseas dispersal in amphibians. *Proceedings of the Royal Society of London* Series B: Biological Sciences. **270(1532)**:2435–2442.

Vineeth KK, Radhakrishna UK, Godwin RD, Anwesha S, Rajashekhar KP, Aravind NA. 2018. A new species of *Microhyla* Tschudi, 1838 (Anura: Microhylidae) from West Coast of India: an integrative taxonomic approach. *Zootaxa* **4420**:151–179.

Voris HK. 2000. Maps of Pleistocene sea levels in Southeast Asia: shorelines, river systems and time durations. *Journal of Biogeography* **27**:1153–1167.

Wijayathilaka N, Garg S, Senevirathne G, Karunarathna N, Biju SD, Meegaskumbura M. 2016. A new species of *Microhyla* (Anura: Microhylidae) from Sri Lanka: an integrative taxonomic approach. *Zootaxa* **4066(3)**: 331–342.

Woodruff DS. 2010. Biogeography and conservation in Southeast Asia: how 2.7 million years of repeated environmental fluctuations affect today's patterns and the future of the remaining refugial-phase biodiversity. *Biodiversity and Conservation* **19**(4):919**–**941.

Wu X, Li Y, Zhang H, Yan L, Wu XB. 2016. The complete mitochondrial genome of *Microhyla pulchra* (Amphidia, Anura, Microhylidae). *Mitochondrial DNA Part A* **27(1)**: 40–41.

Yong HS, Song SL, Lim PE, Eamsobhana P, Tan J. 2016. Complete mitochondrial genome and phylogeny of *Microhyla butleri* (Amphibia: Anura: Microhylidae). *Biochemical Systematics and Ecology* **66**:243–253.

Zhang M, Fei L, Ye C, Wang Y, Wang B, Jiang J. 2018. A New Species of Genus *Microhyla* (Amphibia: Anura: Microhylidae) from Zhejiang Province, China. *Asian Herpetological Research* **9(3)**: 135–148D.

Zhang P, Zhou H, Chen YQ, Liu YF, Qu LH. 2005. Mitogenomic perspectives on the origin and phylogeny of living amphibians. *Systematic Biology* **54(3)**: 391–400.

Zhao EM, Adler K. 1993. *Herpetology of China*. Contributions to herpetology number 10. St. Louis: Society for the Study of Amphibians and Reptiles, 522 pp.

Zhao Y, Meng H, Su L. 2018. The complete mitochondrial genome of the mixtured pygmy frog *Microhyla mixtura* (Anura, Microhylidae). *Conservation Genetics Resources* **10(3)**: 427–430.
