# Supplementary material for: A little frog leaps a long way: compounded colonizations of the Indian Subcontinent discovered in the tiny Oriental frog genus Microhyla (Amphibia: Microhylidae)
Source: PeerJ. 2020 Jul 3;8:e9411. doi: 10.7717/peerj.9411 (PMC7337035; doi:10.7717/peerj.9411)
Supplement: Supplemental Information 18 — See Supplemental Table S1 for sequence details. [file peerj-08-9411-s018.pdf]

Supplemental File 3. Raw data file - aligned newly generated mtDNA sequences (12S rRNA – 16S rRNA fragment) (see Supplemental Table S1 for sequence details).

>M. nepenthicola MN534658, MN534450, MN534551

TTATTATCATCTACCTCTCAACTTACACATGCAAGTATCAGCACACCTGTGAGAACGCCC

TTAATTCTT-TATAGAACAAGGAGCTGGTATCAGGCACAG---ATT------TCTAGCCC

ATAACACCTAGCTTTGCCACACCCTCAAGGGTATTCAGCAGTGATTAACATTGTTTATAA

GCGCCAGCTTGACTCAGTTAGAGAACCC--AGAGCCGGCCAATACGGTGCCAGCCGCCGC

GGCTACACCGCTAGGCTCAAGTTGATATT-ATTCGGCGTTAAGCGTGTTTAAAGTACTT-

-CAT-GATTAGAATTAAACTTAAACCAAGTTGTGACACACTTGTTTTTAAGAAAAACATA

CACGAAAGTTATTCTAACCTAGCCACTTGAACCCACGACAGCTAGGACACAAACTGGGAT

TAGGTACCCCACTATGCCTAGCCGTAAAACATTTATTTACACCT-TAAACCGCCCGGGAA

TTACGAGCCCAAGCTTAAAACCCAAAGGACTTGACGGTGTCCCACCCACCTAGAGGAGCC

TGTTCTATAA-TCGATTCTCCCC-GATATACCTCACCACTTTTAGCCTC--TCAGCCTGT

ATACCTCCGTCGTAAGCTTACCATATGAACGTT--TTTAGTGAGCTAAAAGAT-CATCAC

ATAAATACGTCAGGTCAAGGTGCAGCCAACAAAGTGGTAAGTAATGGGCTACAATTTCTA

CAGC--TAGAACACA-CGAAAGACTACATGCAA-CCCAGTCATGAAGGCGGATTTAGAAG

TAAAAAGAAAATAGAGAGTTCTTTTTAATTAGGCGCTGGGACGCGTACACACCGCCCGTC

ACCCTCTTCAAACGCTCCT-ATTTAG-TTCATAACAAT-TTTATGCACTACAGAAGAGGT

AAGTCGTAACACGGTAAGTGTACTGGAAAGTGCGCTTGGATAAA-CAAAATGTAGCTTAA

CTAAA-GCATCTCGCTTACACCGAGAATATATCAGTGCAACCCAGATCATTTTGA--GCC

CAAAACTTAGCTTAT--CCCCCCCT-AATATTAACC--TAACTGTTTAACAAA---TCAA

TAAAACATTTTATAC-ATTTAGTAAAGGCGATAAAAAAATGTCTG-AAAGCTATAAAA-T

TAGTACCGCAAGGGAACAGTGAAATATTAATGAAACAA-CTTCAAGCACATAAAAGCAGA

GCTGTAACCTCGTACCTTTTGCATCATGGTCTAACTAGTCTAATCAAGCAAAACGAACTT

TAAGTTTGACCCCCCGAAACTAGGTGAGCTACTTAAAAACAGCCTTCT-GAGCAAACCCG

TCTCTGTTGCAAAAGAGTGGGAAGATTTT-TAAGTAGAGGTGACAGACCTACCGAACCTA

GAGATAGCTGGTTATTCAGGAAAAGGATTTAAGTCCTACCTTAGGTTTTCT-ATACA-AC

A-CAATATTTCTTA-AACCTTAAGAGCTATTCAAATAAGGTACAGCCTATTTGAAACAGG

ATACAACCTAACC---

---------AAAAAAGCGTTAAAGCTTCATTATTTTTATTCTAAAAA-TTTCACAA-CC

TCTTTCTAACCCAT-AAATT-ATACTGAATGATCTCATAACCATATGAAAGCCTATATGT

TAGAACTAGTAACAAGAAGACAACCCTTCTCCA-AAATGTAAGCATAAGCCAAAATGAAC

AATTCATTGGCACTTAAAGTTAATAAATCACCAGTAGCAACTCA----ACAAGAAAACTC

TAC------------------TAACTGC-AACGTCAACCTTACACTAGTACATTTCTGGA

AAGATTAAAAGAATGGGAAGGAACTCGGCAAACTA-TTAACCCCGCCTGTTTACCAAAAA

CATCGCCTCTTGCCACCC----ATAAGAGGTCCAGCCTGCCCAGTGACCAA--GTTAAAC

GGCCGCGGTACCCTAACCGTGCAAAGGTAGCGCAATCACTTGTTCTTTAAATGAGGACTA

GTATGAACGGCATCACGAGGGTTATGCTGTCTCCCCACTCTATTCAGTGAAACTGATCTC

CCCGTGAAGAAGCGGGGATAAAAATATAAGACGAGAAGACCCCATGGAGCTTTAAACTCA

GTATCAACTGCTTTACA-AATTAACCTAACATTTAT-GCAA-CTCTGAT-TACTAGTTTT

CGGTTGGGGTGACCACGGAGTATAAAACAACCTCCACGACGAAAGGAAATAACAACCTAA

ATCAAGAGTTACAACTCTAAATATCAAC--------AAATTGACTAAA-TTGATCCAATA

AATT--GATCAACGAACCAAGTTACCCTGGGGATAACAGCGCAATCCATTTCAAGAGCTC

ATATCGACAAATGGGTTTACG-ACC-TCGATGTTGGATCAGGGTACCCCAGTG-GCGCAG

CCGCTA-CTAA-CGGTTC

>M. nepenthicola MN534659, MN534451, MN534552

TTATTATCATCTACCTCTCAACTTACACATGCAAGTATCAGCACACCTGTGAGAACGCCC

TTAATTCTT-TATAGAACAAGGAGCTGGTATCAGGCACAG---ATT------TCTAGCCC

ATAACACCTAGCTTTGCCACACCCTCAAGGGTATTCAGCAGTGATTAACATTGTTTATAA

GCGCCAGCTTGACTCAGTTAGAGAACCC--AGAGCCGGCCAATACGGTGCCAGCCGCCGC

GGCTACACCGCTAGGCTCAAGTTGATATT-ATTCGGCGTTAAGCGTGTTTAAAGTACTT-

-CAT-GATTAGAATTAAACTTAAACCAAGTTGTGACACACTTGTTTTTAAGAAAAACATA

CACGAAAGTTATTCTAACCTAGCCACTTGAACCCACGACAGCTAGGACACAAACTGGGAT

TAGGTACCCCACTATGCCTAGCCGTAAAACATTTATTTACACCT-TAAACCGCCCGGGAA

TTACGAGCCCAAGCTTAAAACCCAAAGGACTTGACGGTGTCCCACCCACCTAGAGGAGCC

TGTTCTATAA-TCGATTCTCCCC-GATATACCTCACCACTTTTAGCCTC--TCAGCCTGT

ATACCTCCGTCGTAAGCTTACCATATGAACGTT--TTTAGTGAGCTAAAAGAT-CATCAC

ATAAATACGTCAGGTCAAGGTGCAGCCAACAAAGTGGTAAGTAATGGGCTACAATTTCTA

CAGC--TAGAACACA-CGAAAGACTACATGCAA-CCCAGTCATGAAGGCGGATTTAGAAG

TAAAAAGAAAATAGAGAGTTCTTTTTAATTAGGCGCTGGGACGCGTACACACCGCCCGTC

ACCCTCTTCAAACGCTCCT-ATTTAG-TTCATAACAAT-TTTATGCACTACAGAAGAGGT

AAGTCGTAACACGGTAAGTGTACTGGAAAGTGCGCTTGGATAAA-CAAAATGTAGCTTAA

CTAAA-GCATCTCGCTTACACCGAGAATATATCAGTGCAACCCAGATCATTTTGA--GCC

CAAAACTTAGCTTAT--CCCCCCCT-AATATTAACC--TAACTGTTTAACAAA---TCAA

TAAAACATTTTATAC-ATTTAGTAAAGGCGATAAAAAAATGTCTG-AAAGCTATAAAA-T

TAGTACCGCAAGGGAACAGTGAAATATTAATGAAACAA-CTTCAAGCACATAAAAGCAGA

GCTGTAACCTCGTACCTTTTGCATCATGGTCTAACTAGTCTAATCAAGCAAAACGAACTT

TAAGTTTGACCCCCCGAAACTAGGTGAGCTACTTAAAAACAGCCTTCT-GAGCAAACCCG

TCTCTGTTGCAAAAGAGTGGGAAGATTTT-TAAGTAGAGGTGACAGACCTACCGAACCTA

GAGATAGCTGGTTATTCAGGAAAAGGATTTAAGTCCTACCTTAGGTTTTCT-ATACA-AC

A-CAATATTTCTTA-AACCTTAAGAGCTATTCAAATAAGGTACAGCCTATTTGAAACAGG

ATACAACCTAACCC--

---------AAAAAAGCGTTAAAGCTTCATTATTTTTATTCTAAAAA-TTTCACAA-CC

TCTTTCTAACCCAT-AAATT-ATACTGAATGATCTCATAACCATATGAGAGCCTATATGT

TAGAACTAGTAACAAGAAGACAACCCTTCTCCA-AAATGTAAGCATAAGCCAAAATGAAC

AATTCATTGGCACTTAAAGTTAATAAATCACCAGTAGCAACTCA----ACAAGAAAACTC

TAC------------------TAACTGC-AACGTCAACCTTACACTAGTACATTTCTGGA

AAGATTAAAAGAATGGGAAGGAACTCGGCAAACTA-TTAACCCCGCCTGTTTACCAAAAA

CATCGCCTCTTGCCACCC----ATAAGAGGTCCAGCCTGCCCAGTGACCAA--GTTAAAC

GGCCGCGGTACCCTAACCGTGCAAAGGTAGCGCAATCACTTGTTCTTTAAATGAGGACTA

GTATGAACGGCATCACGAGGGTTATGCTGTCTCCCCACTCTATTCAGTGAAACTGATCTC

CCCGTGAAGAAGCGGGGATAAAAATATAAGACGAGAAGACCCCATGGAGCTTTAAACTCA

GTATCAACTGCTTTACA-AATTAACCTAACATTTAT-GCAA-CTCTGAT-TACTAGTTTT

CGGTTGGGGTGACCACGGAGTATAAAACAACCTCCACGACGAAAGGAAGTAACAACCTAA

ATCAAGAGTTACAACTCTAAATATCAAC--------AAATTGACTAAA-TTGATCCAATA

AATT--GATCAACGAACCAAGTTACCCTGGGGATAACAGCGCAATCCATTTCAAGAGCTC

ATATCGACAAATGGGTTTACG-ACC-TCGATGTTGGATCAGGGTACCCCAGTG-GCGCAG

CCGCTA-CTAA-CGGTTC

>M. borneensis MN534550, MN534657

----------------------------ATGTCAGTGAAACCCAGATCATTTTGA--GCC

CAAAACTTAGCTTAA--CCCATTCTTAATATTAACC--CAACTCTTTAACAAA---TTAA

TAAAACATTTTACAC-ATTTAGTAAAGGCGATAAAAAAATGTCTAAAAAGCTATAAAA-T

TAGTACCGCAAGGGAACAGTGAAATACTTATGAAACAA-CTTCAAGCACATAAAAGCAGA

GCTATAACCTCGTACCTTTTGCATCATGGTCTAACTAGTCTAATCAAGCAAAACGAACTT

TAAGTTTGACCCCCCGAAACTAGGTGAGCTACTTAAAAACAGCCTTTT-GAGCAAACCCG

TCTCTGTTGCAAAAGAGTGGGAAGATTTT-TAAGTAGAGGTGACAGACCTACCGAACCTA

GAGATAGCTGGTTATTCAGGAAAAGGATTTAAGTCCTACCTTAGGTTCTTT-GTACA-AT

A-CAATACCCCTTA-AACCTTAAGAGCTATTCAAATAAGGTACAGCCTATTTGAAACAGG

ATACAACCTAACCCATAGGGTAACTACCCCAATTA-TTAACCAAGTAGGCCTAAAAGCAG

CCACCTTTC-AAAAAGCGTTAAAGCTTCATTA--TTTTCCCCTAAAAA-TTTCACAA-CC

TCTTTCTAACCCAT-AAATT-ATACTGAATGATCTCATAACCATATGAGAGCCTATATGT

TAGAACTAGTAACAAGAAGACAACCCTTCTCCA-AAATGTAAGCATAAGCCAAAATGAAC

AACTCATTGGCACTTAAAGTTAATAAACCACTAGTAGCAACTCA----ACAAGAAAATTC

TAC------------------TAACTGC-AACGTCAACCTTACACCAGTACATTTCTGGA

AAGATTAAAAGAATGGGAAGGAACTCGGCAAACCA-TTAACCCCGCCTGTTTACCAAAAA

CATCGCCTCTTGCCACCC----ATAAGAGGTCCAGCCTGCCCAGTGACCAA--GTTAAAC

GGCCGCGGTACCCTAACCGTGCAAAGGTAGCGCAATCACTTGTTCTTTAAATAAGGACTA

GTATGAACGGCATCACGAGGGTTATGCTGTCTCCCCACTCTATTCAGTGAAACTGATCTC

CCCGTGAAGAACCGGGGATAAGGATATAAGACGAGAAGACCCCATGGAGCTTTAAACTCA

GTATCAACTGCTTTATA-AATTAACCTAACATTTAT-GCAA-CTCTGAC-TACTAGTTTT

CGGTTGGGGTGACCCCGGAGTTTAAAACAACCTCCCCGACCAAAGGAATTAAC-------

>Microhyla sp. 1 MN534660, MN534452, MN534553

TTATTATCATCTACCTCTCAACTTACACATGCAAGTTTCAGCACACCTGTGAGAACGCCC

TTAATTCTT-TATAGAACAAGGAGCTGGTATCAGGCACAG---ACC------TCTAGCCC

ATAACACCTAGCTTTGCCACGCCCTCAAGGGTATTCAGCAGTGATTAACATTGTCTATAA

GCGCCAGCTTGACTCAGTTAGAGAGCCC--AGAGCCGGCTAATACGGTGCCAGCCGCCGC

GGCTACACCGCTAGGCTCAAGTTGATATT-ATTCGGCGTTAAGCGTGTTTAAAGTACTC-

-CAT-GATTAGAATTAAACTTAAACCAAGTTGTGACACACTTGTTTTTAAGAAAATCATA

CACGAAAGTTATTCTAACCCAACCACTTGAACCCACGACAGCTAAGACACAAACTGGGAT

TAGGTACCCCACTATGCCTAGCCGTAAAATATTTATTTACACCT-TAAACCGCCCGGGAA

TTACGAGCCCAAGCTTAAAACCCAAAGGACTTGACGGTGTCCCACCCACCTAGAGGAGCC

TGTTCTATAA-TCGATTCTCCCCCGATATACCTCACCACTTTTAGCCTC--TCAGCCTGT

ATACCTCCGTCGTAAGCTTACCATATGAACGTT--TTTAGTGAGCTAAAAGAT-CACCAC

ATAAATACGTCAGGTCAAGGTGCAGCCAACGAAGTGGCAAGTAATGGGCTACAATTTCTA

TGTC--TAGAACAAA-CGAAAGACTACATGCAA-CTCAGTCATGAAGGCGGATTTAGAAG

TAAAAAGAAAATAGAGAGTTCTTTTTAATTAGGCGCTGGGACGCGTACACACCGCCCGTC

ACCCTCTTCAAACGCTCCA-ATTTAG-TTCATAACAAC-CTTATGCACTACAGAAGAGGT

AAGTCGTAACACGGTAAGCGTACTGGAAAGTGCGCTTGGATAAA-CAAAATGTAGCTTAA

CCAAA-GCATCTCGCTTACACCGAGAACATGTCAGTGAAACCCAGATCATTTTGA--GCC

CAAAACTTAGCTTAA---CCCTTCTTAATATCAACC--TAACTATACAACAAA---TTAA

TAAAACATTTTACAC-ATTTAGTAAAGGCGATAAAAAAATGTCTA-AAAGCTATAAAA-C

TAGTACCGCAAGGGAACAGTGAAATACTAATGAAATAA-TTTCAAGCACATAAAAGCAGA

GCTGTAACCTCGTACCTTTTGCATCATGGTCTAACTAGTCCAATCAAGCAAAACGAACTT

TAAGTTTGACCCCCCGAAACTAGGTGAGCTACTTAAAAACAGCCTTTT-GAGCCAACCCG

TCTCTGTTGCAAAAGAGTGGGAAGATTTT-TAAGTAGAGGTGACAAACCTACCGAACCTA

GAGATAGCTGGTTATTCAGGAGAAGGATTTAAGTCCTACCTTAGGTTCTTT-GTACA-AT

A-CAATATTTCTTA-AACCTTAAGAGCTATTCAAATAAGGTACAGCCTATT-GAAACAGG

ATACAACCTAACC---

--TTCACAA-CC

TTTTTTCAACCCAT-AAACC-GTACTGAATGATCTCATAACTATATGAGAGCCTATATGT

TAGAACTAGTAACAAGAAGACAATTCTTCTCCA-AAATGTAAGCATAAGCCAAAACGAAC

AATTCATTGGCACTTAAAGTTAATAAACCATCAGTAGCAACTCA----TCAAGAAAAACC

TAC------------------TATCTGA-AACGTCAACCTTACACTAGTACATTACTGGA

AAGATTAAAAGAATGGGAAGGAACTCGGCAAACCA-TTAACCCCGCCTGTTTACCAAAAA

CATCGCCTCTTGCCACCC----ATAAGAGGTCCAGCCTGCCCAGTGACCAA--GTTAAAC

GGCCGCGGTACCCTAACCGTGCAAAGGTAGCGCAATCACTTGTTCTTTAAATAAGGACTA

GTATGAATGGCATCACGAGGGTTATGCTGTCTCCCCACTTTATTCAGTGAAACTGATCTC

CCCGTGAAGAAGCGGGAATAAAAATATAAGACGAGAAGACCCCATGGAGCTTTAAACTCA

GTATCAACTGCTCCATA-AATCAACTTAACATTTAT-GCAA-CTCTGAC-TACTAGTTTT

CGGTTGGGGTGACCACGGAGTATAAAACAACCTCCACGACGAAAGGAAATAATAACCTAA

ATCAAGAGCTACAACTCTAAATATCAAC--------AAATTGACTAAA-TTGATCCAATA

AATT--GATCAACGAACCAAGTTACCCTGGGGATAACAGCGCAATCCATTTCAAGAGCTC

ATATCGACAAATGGGTTTACG-ACC-TCGATGTTGGATCAGGGTATCCCAGTG-GCGCAG

CCGCTA-CTAA-CGGTTC

>M. malang MN534661, MN534453, MN534554

TTATTATCACCTACCTCTCAACTTACACATGCAAGTATCAGCACACCTGTGAGAACGCCC

TTAATTCTT-TATAGAACAAGGAGCTGGTATCAGGCACAG---ACCC-----CCTAGCCC

ATGACACCTAGCTTTGCCACACCCTCAAGGGTATTCAGCAGTGATTAACATTGTTCATAA

GCGCCAGCTTGACTCAGTTAGAGAGCCC--AGAGCCGGCTAATACGGTGCCAGCCGCCGC

GGCTACACCGCTAGGCTCAAGTTGATATT-ATTCGGCGTTAAGCGTGTTTAAAGTACTA-

TCAC-GATTAGAATTGAACTTAAACCAAGTTGTGACACACTTGTTCTTAAGAAAAACATA

AACGAAAGTTATTCTAACATAACCACTTGAACCCACGACAGCTAAGAAACAAACTGGGAT

TAGGTACCCCACTATGCTTAGCCGTAAAACATTTATTTACACCT-TAAACCGCCCGGGAA

TTACGAGCCCAAGCTTAAAACCCAAAGGACTTGACGGTGTCCCACCCACCTAGAGGAGCC

TGTTCTATAA-TCGATTCTCCCC-GATACACCTCTCCACTTCTAGCCTC--TCAGCCTGT

ATACCTCCGTCGTAAGCTTACCATATGAACGTT-TTCTAGTGAGCTAAAAGAT-TACCAC

ATAAATACGTCAGGTCAAGGTGCAGCCCACGAAGTGGCAAGTAATGGGCTACAATTTCTA

TAAC--TAGAACACA-CGAAAGACTACATGCAA-CCCAGTCATGAAGGCGGATTTAGAAG

TAAAAAGAAAATAGAGAGTTCTTTTTAATTAGGCGCTGGGACGCGTACACACCGCCCGTC

ACCCTCTTCAAACGCTCAA-AATTAG-TTCATAACAAC-CCTATGCACTACAGAAGAGGT

AAGTCGTAACACGGTAAGCGTACTGGAAAGTGCGCTTGGATAAA-CAAAATGTAGCTTAA

CTAAA-GCATCTCGCTTACACCGAGAACATGTCAGTGAAACCCGGATCATTTTGA--GCC

AAAAACTTAGCTTAACCCCTCTTCTTAATGTACACA--CAACCATTTAACAAA---TTAA

TAAAACATTTTACAC-ATTTAGTAAAGGCGATAAAAAAATGTCTA-AGAGCTATAAAA-C

TAGTACCGCAAGGGAATAGTGAAATATTAATGAAAAA--CTCCAAGCACACAAAAGCAGA

GCTTTAACCTCGTACCTTTTGCATCATGGTCTAACTAGTCTAATCAAGCAAAACGAATTT

TAAGTTTGACCCCCCGAAACTAGGTGAGCTACTTAAAAACAGCCTTAT-GGGCCAACCCG

TCTCTGTTGCAAAAGAGTGGGAAGATTTTTTAAGTAGAGGTGACAAACCTACCGAACCTA

GAGATAGCTGGTTATTCAGGAAAAGGATTTAAGTCCTACCTTAGGTTATTC-GTATA-TT

A-TAA---

---AAAGCAG

CCACCTTCCAAAAAAGCGTTAAAGCTTTATTA---TTCTATCCCAAAA-TTCCACTA-CC

ATTTATTAACCCTT-AAACC-GTACTGAATGATCTCATAACCTTATGAGAGCCTATATGT

TAGAACTAGTAACAAGAAGACAGCCCTTCTCCA-AAATGTAAACATGAACCAAAACGAAC

CACTCATTGGCACTTAAAGTTAATAAATCAATAGTAGAAACCTA----ACAAGAAAAGTC

TAC------------------TAAATAC-AACGTCAACCTTACACTAGTACATTACTGGA

AAGAATAAAAGAATGGGAAGGAACTCGGCAAACCA-CTAACCCCGCCTGTTTACCAAAAA

CATCGCCTCTTGACACCC----ATAAGAGGTCCAGCCTGCCCAGTGACTAA--GTTAAAC

GGCCGCGGTACCCTAACCGTGCAAAGGTAGCGCAATCACTTGTTCTTTAAATGAGGACTA

GTATGAACGGCATCACGAGGGTTATGCTGTCTCCCCACTCTATTCAGTGAAACTGATCTC

CCCGTGAAGAAGCGGGGATAAAAATATAAGACGAGAAGACCCCATGGAGCTTTAAACTCA

GTATCAACTGCTTTATA-AATCAACCTAATATCTAT-GCAA-CCCTGAT-TACTAGTTTT

CGGTTGGGGTGACCACGGAGCATAAAACAACCTCCACGACGAAAGGAAGTAATAACCTAA

ATTAAGAGTTACAACTCTAAATATCAAC--------AAATTGACTAAA-TTGATCCAATA

AATT--GATCAACGAACCAAGTTACCCTGGGGATAACAGCGCAATCCATTTCAAGAGCTC

ATATCGACAAATGGGTTTACG-ACC-TCGATGTTGGATCAGGGTATCCCAGTG-GCGCAG

CCGCTA-CTAA-CGGTTC

>M. malang MN534662, MN534454, MN534555

TTATTATCACCTACCTCTCAACTTACACATGCAAGTATCAGCACACCTGTGAGAACGCCC

TTAATTCTT-TATAGAACAAGGAGCTGGTATCAGGCACAG---ACCC-----CCTAGCCC

ATGACACCTAGCTTTGCCACACCCTCAAGGGTATTCAGCAGTGATTAACATTGTTCATAA

GCGCCAGCTTGACTCAGTTAGAGAGCCC--AGAGCCGGCTAATACGGTGCCAGCCGCCGC

GGCTACACCGCTAGGCTCAAGTTGATATT-ATTCGGCGTTAAGCGTGTTTAAAGTACTA-

TCAC-GATTAGAATTGAACTTAAACCAAGTTGTGACACACTTGTTCTTAAGAAAAACATA

AACGAAAGTTATTCTAACATAACCACTTGAACCCACGACAGCTAAGAAACAAACTGGGAT

TAGGTACTCCCCTATGCTTAACCGTAAAACATTTATTTACACCT-TAAACCGCCCGGGAA

TTACGAGCCCAAGCTTAAAACCCAAAGGACTTGACGGTGTCCCACCCACCTAGAGGAGCC

TGTTCTATAA-TCGATTCTCCCC-GATACACCTCTCCACTTCTAGCCTC--TCAGCCTGT

ATACCTCCGTCGTAAGCTTACCATATGAACGTT-TTCTAGTGAGCTAAAAGAT-TACCAC

ATAAATACGTCAGGTCAAGGTGCAGCCCACGAAGTGGCAAGTAATGGGCTACAATTTCTA

TAAC--TAGAACACA-CGAAAGACTACATGCAA-CCCAGTCATGAAGGCGGATTTAGAAG

TAAAAAGAAAATAGAGAGTTCTTTTTAATTAGGCGCTGGGACGCGTACACACCGCCCGTC

ACCCTCTTCAAACGCTCAA-AATTAG-TTCATAACAAC-CCTATGCACTACAGAAGAGGT

AAGTCGTAACACGGTAAGCGTACTGGAAAGTGCGCTTGGATAAA-CAAAATGTAGCTTAA

CTAAA-GCATCTCGCTTACACCGAGAACATGTCAGTGAAACCCGGATCATTTTGA--GCC

AAAAACTTAGCTTAACCCCTCTTCTTAATGTACACA--CAACCATTTAACAAA---TTAA

TAAAACATTTTACAC-ATTTAGTAAAGGCGATAAAAAAATGTCTA-AGAGCTATAAAA-C

TAGTACCGCAAGGGAATAGTGAAATATTAATGAAAAA--CTCCAAGCACACAAAAGCAGA

GCTTTAACCTCGTACCTTTTGCATCATGGTCTAACTAGTCTAATCAAGCAAAACGAATTT

TAAGTTTGACCCCCCGAAACTAGGTGAGCTACTTAAAAACAGCCTTAT-GGGCCAACCCG

TCTCTGTTGCAAAAGAGTGGGAAGATTTTTTAAGTAGAGGTGACAAACCTACCGAACCTA

GAGATAGCTGGTTATTCAGGAAAAGGATTTAAGTCCTACCTTAGGTTATTC-GTATA-TT

A-TAA---

---AAAGCAG

CCACCTTCC-AAAAAGCGTTAAAGCTTTATTA---TTCTATCTCAAAA-TTCCACTA-CC

ATTTATTAACCCTT-AAACC-GTACTGAATGATCTCATAACCTTATGAGAGCCTATATGT

TAGAACTAGTAACAAGAAGACAGCCCTTCTCCA-AAATGTAAACATGAACCAAAACGAAC

CACTCATTGGCACTTAAAGTTAATAAATCAATAGTAGAAACCTA----ACAAGAAAAGTC

TAC------------------TAAATAC-AACGTCAACCTTACACTAGTACATTACTGGA

AAGAATAAAAGAATGGGAAGGAACTCGGCAAACCA-CTAACCCCGCCTGTTTACCAAAAA

CATCGCCTCTTGACACCC----ATAAGAGGTCCAGCCTGCCCAGTGACTAA--GTTAAAC

GGCCGCGGTACCCTAACCGTGCAAAGGTAGCGCAATCACTTGTTCTTTAAATGAGGACTA

GTATGAACGGCATCACGAGGGTTATGCTGTCTCCCCACTCTATTCAGTGAAACTGATCTC

CCCGTGAAGAAGCGGGGATAAAAATATAAGACGAGAAGACCCCATGGAGCTTTAAACTCA

GTATCAACTGCTTTATA-AATCAACCTAATATCTAT-GCAA-CCCTGAT-TACTAGTTTT

CGGTTGGGGTGACCACGGAGCATAAAATAACCTCCACGACGAAAGGAAGTAATAACCTAA

ATTAAGAGTTACAACTCTAAATATCAAC--------AAATTGACTAAA-TTGATCCAATA

AATT--GATCAACGAACCAAGTTACCCTGGGGATAACAGCGCAATCCATTTCAAGAGCTC

ATATCGACAAATGGGTTTACG-ACC-TCGATGTTGGATCAGGGTATCCCAGTG-GCGCAG

CCGCTA-CTAA-CGGTTC

>M. orientalis MN534663, MN534455, MN534556

TTATTATCATCTACTTCTCAACTTACACATGCAAGTATCAGCACACCCGTGAGAACGCCC

TTTCCTCTT-CATAGAATAAGGAGCTGGTATCAGGCACAG---ACC------TCTAGCCC

ATGACACCTAGTTTTGCCACACCCTCAAGGGTATTCAGCAGTGATTAACATTGTTTATAA

GCGCCAGCTTGACTCAGTTAGAGAACCC--AGGGCCGGCAAATACGGTGCCAGCCGCCGC

GGCTACACCGCTAGGCTCAAGTTGATATT-ATTCGGCGTTAAGCGTGTTTAAAGTGTCT-

ACAA-GATTAGAATTGAACTTAAACCAAGTTGTGACACACTTGTTTTTAAGAAAAACGCT

TACGAAAGTTATTCTAACATAACCACTTGAACCCACGACAGCTAGGACACAAACTGGGAT

TAGGTACCCCACTATGCCTAGCCGTAAAATATTTATTTACACCT-TTAACCGCCCGGGAA

TTACGAGCCCAAGCTTAAAACCCAAAGGACTTGACGGTGTCCCACCCACCTAGAGGAGCC

TGTTCTATAA-TCGATTCCCCCCGATATACCTCACCACTTTTAGCCTC--TCAGCCTGT

ATACCTCCGTCGTAAGCTTACCATATGAATGCA-TTTTAGTGAGCTAAAAGAT-TCTTAC

ATAAACACGTCAGGTCAAGGTGCAGCCCACGAAGTGGAAAGCAATGGGCTACAATTTCTA

TAAC--TAGAACATA-CGAAAGACTACATGCAA-CCTAGTCATGAAGGCGGATTTAGCAG

TAAAAAGAAACTAGAGAGTTCTTTTTAATTAGGCACTGGGACGCGTACACACCGCCCGTC

ACCCTCTTCAAATGCCCCA-ATTCAG-TTTATAACAAC-TCTATGCACTACAGAAGAGGT

AAGTCGTAACACGGTAAGCGTACTGGAAAGTGCGCTTGGATAAA-CAAAATGTAACTTAA

TTAAA-GTACCTTGTTTACACCAAGATTATGTCTGTGAAGCCCAGATCATTTTGA--GCT

AAAAACTTAGCTTTAACTTCTTTAT-AATACTCAAC--ATACCTCTTAATAAA---CCAA

TAAAACATTTTCTAT-GTTTAGTAAAGGCGATCAAAAAATACCTA-AAAGCTATAAAA-T

TAGTACCGCAAGGGAACAGTGAAATATTAATGAAAAA--CCTCAAGCACACAAAAGCAGA

GTTATAATCTTGTACCTTTTGCATCATGGTCTAACTAGTCTAATCAAGCAAAAAGAACTT

TAAGTCTGACCCCCCGAAACTAGGTGAGCTACTTAGAAACAGCCTCAT-GGGCCAACCCG

TCTCTGTTGCAAAAGAGTGGGAAGATTTT-TAAGTAGAGGTGATAAACCTACCGAACCTA

GAGATAGCTGGTTATTCAGGAAAAGGATTTAAGTCCTACCTTAGGTTTTTC-GTATT-AT

A-TAATACCCCCCT-AACCTTAAGAGCTATTCAAATAAGGTACAGCCTATTTGAAATAGG

ATACAACCTCAC--

---------------------------TATTA---TTTCTTCTACAA--TTCCACCA-CC

ATATTTTAACCCCT-AAATA-GTACTGAATGATTCCATAACTTTATGGAAGCCTATATGT

TAGAACTAGTAACAAGAAGGCAGCCCTTCTCCA-AAATGTAAGCATGAGCCAAAATGAAC

TATTCATTGGCACTTAAAGCTAATAAACCACTAGTAGCAACTCA---ATCAAGAAAAACC

TAC------------------TAATAAT-ATCGTCAACCTTACACTAGCACATTTCTGGA

AAGATTAAAAGACTGGGAAGGAACTCGGCAAACTACTTAACCCCGCCTGTTTACCAAAAA

CATCGCCTCTTGTTTTTATATTATAAGAGGTCCAGCCTGCCCAGTGACAAA--GTTAAAC

GGCCGCGGTACCCTAACCGTGCAAAGGTAGCACAATCACTTGTTCTTTAAATGAGGACTA

GTATGAACGGCATCACGAGGGTTATGCTGTCTCCCCATTCTATTCAGTGAAACTGATCTC

TCCGTGAAGAAGCGGAGATAAAAATATAAGACGAGAAGACCCCATGGAGCTTTAAACTCA

AAATCAATTGCTTTAAACAATTAACCTAATATTTAT-GCAA-CCCTGAT-TTCTAGTTTT

CGGTTGGGGTGACCACGGAGTACAAACAAACCTCCATGATGAAAGGAACTAATAACCTAA

ATCAAGAGCTACAGCTCTAAATATCAAC--------AAACTGACTAAA-TTGATCCAATT

ACTT--GATCAACGAACCAAGTTACCCTGGGGATAACAGCGCAATCCATTTCAAGAGCTC
ATATCGACAAATGGGTTTACG-ACC-TCGATGTTGGATCAGGGTATCCCAGTG-GCGCAG
CCGCTA-CTAA-CGGTTC
>M. orientalis MN534664, MN534456, MN534557
TTATTATCATCTACTTCTCAACTTACACATGCAAGTATCAGCACACCCGTGAGAACGCCC
TTTCCTCTT-CATAGAATAAGGAGCTGGTATCAGGCACAG---ACC------TCTAGCCC
ATGACACCTAGTTTTGCCACACCCTCAAGGGTATTCAGCAGTGATTAACATTGTTTATAA
GCGCCAGCTTGACTCAGTTAGAGAACCC--AGGGCCGGCAAATACGGTGCCAGCCGCCGC
GGCTACACCGCTAGGCTCAAGTTGATATT-ATTCGGCGTTAAGCGTGTTTAAAGTGCCT-
ACAA-GATTAGAATTGAACTTAAACCAAGTTGTGACACACTTGTTTTTAAGAAAAACGCT
TACGAAAGTTATTCTAACATAACCACTTGAACCCACGACAGCTAGGACACAAACTGGGAT
TAGGTACCCCACTATGCCTAGCCGTAAAATATTTATTTACACCT-TTAACCGCCCGGGAA
TTACGAGCCCAAGCTTAAAACCCAAAGGACTTGACGGTGTCCCACCCACCTAGAGGAGCC
TGTTCTATAA-TCGATTCCCCCCGATATACCTCACCACTTTTAGCCTC--TCAGCCTGT
ATACCTCCGTCGTAAGCTTACCATATGAATGCA-TTTTAGTGAGCTAAAAGAT-TCTTAC
ATAAACACGTCAGGTCAAGGTGCAGCCCACGAAGTGGAAAGCAATGGGCTACAATTTCTA
TAAC--TAGAACATA-CGAAAGACTACATGCAA-CCTAGTCATGAAGGCGGATTTAGCAG
TAAAAAGAAACTAGAGAGTTCTTTTTAATTAGGCACTGGGACGCGTACACACCGCCCGTC
ACCCTCTTCAAATGCCCCA-ATTCAG-TTTATAACAAC-TCTATGCACTACAGAAGAGGT
AAGTCGTAACACGGTAAGCGTACTGGAAAGTGCGCTTGGATAAA-CAAAATGTAACTTAA
TTAAA-GTACCTTGTTTACACCAAGATTATGTCTGTGAAGCCCAGATCATTTTGA--GCT
AAAAACTTAGCTTTAACTTCTTTAT-AATACTCAAC--ATACCTCTTAATAAA---CCAA
TAAAACATTTTCTAT-GTTTAGTAAAGGCGATCAAAAAATACCTA-AAAGCTATAAAA-T
TAGTACCGCAAGGGAACAGTGAAATATTAATGAAAAA--CCTCAAGCACACAAAAGCAGA
GTTATAATCTTGTACCTTTTGCATCATGGTCTAACTAGTCTAATCAAGCAAAAAGAACTT
TAAGTCTGACCCCCCGAAACTAGGTGAGCTACTTAGAAACAGCCTCAT-GGGCCAACCCG
TCTCTGTTGCAAAAGAGTGGGAAGATTTT-TAAGTAGAGGTGATAAACCTACCGAACCTA
GAGATAGCTGGTTATTCAGGAAAAGGATTTAAGTCCTACCTTAGGTTTTTC-GTATC-AT
A-TAATACCCCCCT-AACCTTAAGAGCTATTCAAATAAGGTACAGCCTATTTGAAATAGG
ATACAACCTCAC--

---------------------------TATTA---TTTCTTCTAAAAA-TTCCACCA-CC

ATATTTTAACCCCT-AAATA-GTACTGAATGATTCCATAACTTTATGGAAGCCTATATGT

TAGAACTAGTAACAAGAAGGCAGCCCTTCTCCA-AAATGTAAGCATGAGCCAAAATGAAC

TATTCATTGGCACTTAAAGCTAATAAACCACTAGTAGCAACTCA---ATCAAGAAAAACC

TAC------------------TAATAAC-ATCGTCAACCTTACACTAGCACATTTCTGGA

AAGATTAAAAGACTGGGAAGGAACTCGGCAAACTACTTAACCCCGCCTGTTTACCAAAAA

CATCGCCTCTTGTTTTTATATTATAAGAGGTCCAGCCTGCCCAGTGACAAA--GTTAAAC

GGCCGCGGTACCCTAACCGTGCAAAGGTAGCACAATCACTTGTTCTTTAAATGAGGACTA

GTATGAACGGCATCACGAGGGTTATGCTGTCTCCCCACTCTATTCAGTGAAACTGATCTC

TCCGTGAAGAAGCGGAGATAAAAATATAAGACGAGAAGACCCCATGGAGCTTTAAACTCA

AAATCAATTGCTTTAAACAATTAACCTAATATTTAT-GCAA-CCCTGAT-TTCTAGTTTT

CGGTTGGGGTGACCACGGAGTACAAACAAACCTCCATGATGAAAGGAACTAATAACCTAA

ATCAAGAGCTACAGCTCTAAATATCAAC--------AAACTGACTAAA-TTGATCCAATT

ACTT--GATCAACGAACCAAGTTACCCTGGGGATAACAGCGCAATCCATTTCAAGAGCTC

ATATCGACAAATGGGTTTACG-ACC-TCGATGTTGGATCAGGGTATCCCAGTG-GCGCAG

CCGCTA-CTAA-CGGTTC

>M. mantheyi MN534665, MN534457, MN534558

TTATTATCATCTATTTCTCAACTTACACATGCAAGTATCAGCACACCCGTGAGAACGCCC

TTCCCCCTT-CATAGGACAAGGAGCCGGTATCAGGCACAG---TCT------TCTAGCCC

ATGACACCTAGCTTTGCCACACCCTCAAGGGTATTCAGCAGTGATTAACATTGTTCATAA

GCGCCAGCTTGATTCAGTCAGAGAACCC--AGAGCCGGCTAATACGGTGCCAGCCGCCGC

GGCTACACCGCTGGGCTCAAGTTGATACTATTTCGGCGTTAAGCGTGTTTAAAGTGCTC-

ACTA-AATTAGAATTGAACTTAAACCAAGTTGTGACATGCTTGTTTTTAAGAAAAACAAA

CACGAAAGTTATTCTAAACTAACCACTTGAACCCACGACAGCTAAGACACAAACTGGGAT

TAGGTACCCCACTATGCTTAGCCGTAAAATATTTACTTACACCC-CAAACCGCCCGGGAA

TTACGAGCCCAAGCTTAAAACCCAAAGGACTTGACGGTGTCCCACCCACCTAGAGGAGCC

TGTTCTATAA-TCGATTCCCCCC-GATATACCTCACCACTTTTAGCCTT--TCAGCCTGT

ATACCTCCGTCGTAAGCTTACCATATGAACGCT-TTTTAGTGAGCTAAAAGAT-TTCTAC

ATAAATACGTCAGGTCAAGGTGCAGCCCACGAAGTGGCAAGTAATGGGCTACAATTTCTA

CAAC--TAGAACACA-CGAAAGACTACATGCAA-CCTAGTCATGAAGGCGGATTTAGAAG

TAAAAAGAAAATAGAGAGTTCTTTTTAATTAGGCACTGGGACGCGTACACACCGCCCGTC

ACCCTCTTCAAACGCTCCA-ACTTAG-TTCATAACAAC-TCTATGCACAACAGAAGAGGT

AAGTCGTAACACGGTAAGGGTATGGGAAAGTGCGCTTGGAAA---CAAAAGGTAGCTTAA

TTAAA-GCATCTCGCTTACACCGAGAACATGTCCGTGAAACTCAGATCATTTTGA--GCC

AAAAACTTAG-TTAAACTTCGTTCT-AATGTTCCCCC-CCACCACCACATCAA---CCAA

TAAAACATTTTCTAC-ATTTAGTAAAGGCGATCAAAAAATGTCTA-AAAGCTATAAAA-T

TAGTACCGCAAGGGAATAGTGAAATATTAATGAAAA---CCTAAAGCACATAAAAGCAGA

GCTATAACCTCGTACCTTTTGCATCATGGTCTAACTAGTCTAATCAAGCAAAACGAACTT

TAAGTCTGACCCCCCGAAACTAGGTGAGCTACTTAAAAACAGCCTTAC-GGGCTAACCCG

TCTCTGTTGCAAAAGAGTGGGAAGATTTT-TAAGTAGAGGTGACAGACCTATCGAACCTA

GAGATAGCTGGTTATTCAGGAAAAGGATTTAAGTCCTACCTTAGGTTTTTT-ATACC-CC

C-AAATATCTACCA-AACCTTAAGAGCTATTCAAATAAGGTACAGCCTATTTGAAACAGG

ATACAACCTCACCAA---

-CACCTTCC-AAAAAGCGTTAAAGCTTCATTA---TTATGCATAAAAA-TTCCACCA-CC

TCTTTTTAACCCCT-AAACC-GTACTGAATGATCCCATAGCCCTATGGGAGCCTATATGT

TAGAACTAGTAACAAGAAGATAGTTCTTCTCCA-AAATGTAAGCATGGGCCAAAATGAAC

CACTCATTGGCACTTAAAGTTAATAAACCCTTAGTAGTAACCCA----GCAAGAAAAATC

TAC------------------TAATTAT-AACGTCAACCTTACACAAGCACATTTCTGGA

AAGATTAAAAAAATGGGAAGGAACTCGGCAAACTA-TTAACCCCGCCTGTTTACCAAAAA

CATCGCCTCTTGTTACCC----ATAAGAGGTCCAGCCTGCCCAGTGACAAA--GTTAAAC

GGCCGCGGTACCCTAACCGTGCAAAGGTAGCGCAATCACTTGTTCTTTAAATGAGGACTA

GTATGAATGGCATCACGAGGGTTATGCTGTCTCCCCAATTTACTCAGTGAAACTGATCTC

CCCGTGAAGAAGCGGGGATAAGAATATAAGACGAGAAGACCCCATGGAGCTTTAAACTCA

GTATCAACTGCACCACA-AAATAACCTAATATTTAT-GCAA-CCCTGTC-TACTAGTTTT

CGGTTGGGGTGACCACGGAGTATAAACAAACCTCCACGATGAAAGGAACTAACAACCTAA

ACCAAGAGCTACAGCTCTAAATATCAAC--------AAATTGACTAAA-TTGATCCAATC

ACTT--GATCAACGAACCAAGTTACCCTGGGGATAACAGCGCAATCCATTTCAAGAGCTC

ATATCGACAAATGGGTTTACG-ACC-TCGATGTTGGATCAGGGTATCCAAGTG-GCGCAG

CCGCTA-CTAA-TGGTTC

>M. mantheyi MN534666, MN534458, MN534559

TTATTATCATCTATTTCTCAACTTACACATGCAAGTATCAGCACACCCGTGAGAACGCCC

TTCCCCCTT-CATAGGACAAGGAGCCGGTATCAGGCACAG---TCT------TCTAGCCC

ATGACACCTAGCTTTGCCACACCCTCAAGGGTATTCAGCAGTGATTAACATTGTTCATAA

GCGCCAGCTTGATTCAGTCAGAGAACCC--AGAGCCGGCTAATACGGTGCCAGCCGCCGC

GGCTACACCGCTAGGCTCAAGTTGATACTATTTCGGCGTTAAGCGTGTTTAAAGTGCTC-

ACTA-AATTAGAATTGAACTTAAACCAAGTTGTGACATGCTTGTTTTTAAGAAAAACAAA

CACGAAAGTTATTCTAAACTAACCACTTGAACCCACGACAGCTAAGACACAAACTGGGAT

TAGGTACCCCACTATGCTTAGCCGTAAAATATTTACTTACACCC-TAAACCGCCCGGGAA

TTACGAGCCCAAGCTTAAAACCCAAAGGACTTGACGGTGTCCCACCCACCTAGAGGAGCC

TGTTCTATAA-TCGATTCCCCCC-GATATACCTCACCACTTTTAGCCTT--TCAGCCTGT

ATACCTCCGTCGTAAGCTTACCATATGAACGCT-TTTTAGTGAGCTAAAAGAT-TTCTAC

ATAAATACGTCAGGTCAAGGTGCAGCCCACGAAGTGGCAAGTAATGGGCTACAATTTCTA

CAAC--TAGAACACA-CGAAAGACTACATGCAA-CCTAGTCATGAAGGCGGATTTAGAAG

TAAAAAGAAAATAGAGAGTTCTTTTTAATTAGGCACTGGGACGCGTACACACCGCCCGTC

ACCCTCTTCAAACGCTCCA-ACTTAG-TTCATAACAAC-TCTATGCACAACAGAAGAGGT

AAGTCGTAACACGGTAAGGGTATGGGAAAGTGCGCTTGGAAA---CAAAAGGTAGCTTAA

TTAAA-GCATCTCGCTTACACCGAGAACATGTCCGTGAAACTCAGATCATTTTGA--GCC

AAAAATTTAG-TTAAACTTCGTTCT-AATGTTCCCCC-CCACCACCACATCAA---CCAA

TAAAACATTTTCTAC-ATTTAGTAAAGGCGATCAAAAAATGTCTA-AAAGCTATAAAA-T

TAGTACCGCAAGGGAATAGTGAAATATTAATGAAAA---CCTAAAGCACATAAAAGCAGA

GCTATAACCTCGTACCTTTTGCATCATGGTCTAACTAGTCTAATCAAGCAAAACGAACTT

TAAGTCTGACCCCCCGAAACTAGGTGAGCTACTTAAAAACAGCCTTAC-GGGCTAACCCG

TCTCTGTTGCAAAAGAGTGGGAAGATTTT-TAAGTAGAGGTGACAGACCTATCGAACCTA

GAGATAGCTGGTTATTCAGGAAAAGGATTTAAGTCCTACCTTAGGTTTTTT-ATACC-CC

C-AAATATTTACCA-AACCTTAAGAGCTATTCAAATAAGGTACAGCCTATTTGAAACAGG

ATACAACCTCACCAAT--

------TCC-AAAAAGCGTTAAAGCTTCATTA---TTACGCATAAAAA-TTCCACCA-CC

TCTTTTTAACCCCT-AAACC-GTACTGAATGATCCCATAGCCCTATGGGAGCCTATATGT

TAGAACTAGTAACAAGAAGATAGTTCTTCTCCA-AAATGTAAGCATGGGCCAAAATGAAC

CACTCATTGGCACTTAAAGTTAATAAACCCTTAGTAGTAACCCA----GCAAGAAAATC

TAC------------------TAATTAT-AACGTCAATCTTACACAAGCACATTTCTGGA

AAGATTAAAAAAATGGGAAGGAACTCGGCAAACTA-TTAACCCCGCCTGTTTACCAAAAA

CATCGCCTCTTGTTACCC----ATAAGAGGTCCAGCCTGCCCAGTGACAAA--GTTAAAC

GGCCGCGGTACCCTAACCGTGCAAAGGTAGCGCAATCACTTGTTCTTTAAATGAGGACTA

GTATGAATGGCATCACGAGGGTTATGCTGTCTCCCCAATTTACTCAGTGAAACTGATCTC

CCCGTGAAGAAGCGGGGATAAGAATATAAGACGAGAAGACCCCATGGAGCTTTAAACTCA

GTATCAATTGCACCACA-AAATAACCTAATATTTAT-GCAA-CCCTGTC-TACTAGTTTT

CGGTTGGGGTGACCACGGAGTATAAACAAACCTCCACGATGAAAGGAACTAACAACCTAA

ACCAAGAGCTACAGCTCTAAATATCAAC--------AAATTGACTAAA-TTGATCCAATC

ACTT--GATCAACGAACCAAGTTACCCTGGGGATAACAGCGCAATCCATTTCAAGAGCTC

ATATCGACAAATGGGTTTACG-ACC-TCGATGTTGGATCAGGGTATCCAAGTG-GCGCAG

CCGCTA-CTAA-CGGTTC

>M. minuta MN534667, MN534459, MN534560

TTACTATCATCTTTATCTCAACTTACACATGCAAGTATCCGCATCCCCGTGAGAACGCCC

TTTCCCCTT-AATAGGATAAGGAGCTGGTATCAGGCACAG---GCCC-----CCTAGCCC

ATGACACCTAGCTTTGCCACACCCTCAAGGGTATTCAGCAGTGATTAACATTGTTTATAA

GCGCCAGCTTGACTCAGTTAGAGAATTA--AGAGCCGGCCAATACGGTGCCAGCCGCCGC

GGCTACACCGCTAGGCTCAAGTTGATATT-ACTCGGCGTTAAGCGTGTTTAAAGTGCTA-

CAAATAATTAGAATTAAACTTAAACCAAGTTGTGACACGCTTGTTTCTAAGAAAAACATA

AACGAAAGTTATTCTAATTTAACCACTTGAACCCACGACAGCTAGGACACAAACTGGGAT

TAGGTACCCCACTATGCCTAGCCGTAAAATATTTACTTACACCT-TTAACCGCCCGGGAA

TTACGAGCCCAAGCTTAAAACCCAAAGGACTTGACGGTGTCCCACCCACCTAGAGGAGCC

TGTTCTATAA-TCGATTCTCCCC-GATACACCTCACCACTTCTAGCCTC--TCAGCCTGT

ATACCTCCGTCGTAAGCTTACCATATGAACGAT-TTTTAGTGAGCTAAAAGAT-TTACGC

ATAAACACGTCAGGTCAAGGTGCAGCCAACGAAATGGTAAGCAATGGGCTACAATTTCTA

TAAT--TAGAACACA-CGAAAGACTACATGCAA-CTCAGTCATGAAGGCGGATTTAGCAG

TAAAAAGAAAATAGAGAGTTCTTTTTAATTAGGCACTGGGACGCGTACACACCGCCCGTC

ACCCTCTTCAAACGCTCCA-GCTCAG-TTTATAACACC-ACTATGCACCATAGAAGAGGT

AAGTCGTAACATGGTAAGCGTACTGGAAAGTGCGCTTGGATAAA-CAAAATGTAGCTTAA

TTAAA-GCATCTCGCTTACACCGAAAATATGTCTGTGAAACTCAGATCATTTTGA--GCC

AAAAACTTAGCTTAA--CTCCTTCTCAATGCACACC--TTACTTTATAATAAT---TTAA

TAAAACATTTTCCAC-ATTTAGTAAAGGCGATCAAAAAATGTCTA-AAAGCTATAAAA-T

TAGTACCGCAAGGGAACAGTGAAATAAAAATGAAAAA--TCTAAAGCACATAAAAGCAGA

GTCACATCCTCGTACCTTTTGCATCATGGTTTAACTAGTCTAATCAAGCAAAACGAATTT

TAAGTTTGACCCCCCGAAACTAGGTGAGCTACTTAAAAACAGCCTTAT-GGGCAAACCCG

TCTCTGTTGCAAAAGAGTGGGAAGATTCT-TAAGTAGAGGTGACAGACCTATCGAACCTA

GAGATAGCTGGTTATTCAGGAAAAGGATTTAAGTCCTACCTTAGGTTTTTT-TATAC-TC

A-TAAATACCCATA-AACCTTAAGAGCTATTCAAATAAGGTACAGCCTATTTGAAACAGG

ATACAACCTA--CTAAAAGCAG

CCACCTTCC-AAAAAGCGTTAAAGCTTAATTA---TTTTTATTAAAAA-TTCCACCA-TT

TTTCTACAACCCTT-AATTT-ATACTGAATGATCTCATAAGCTTATGAGAGCCTATATGT

TAAAACTAGTAACAAGAAGA-AGACCTTCTCCA-AAATGTAAGCATGAGCCAAAATGAAC

AATTCATTGGCATTTAAAGTTA-TAAACCTCCAGTAGTAACACT----ACAAGAAAAACC

TAC------------------TTACTTT-GACGTCAATCTTACACTAGCACATTTCTGGA

AAGATTAAAAGAATGGGAAGGAACTCGGCAAACTA-CTAACCCCGCCTGTTTACCAAAAA

CATCGCCTCTTGCTTCTC----ATAAGAGGTCCAGCCTGCCCAGTGACAAA--GTTAAAC

GGCCGCGGTACCCTAACCGTGCAAAGGTAGCGCAATCACTTGTTCTTTAAATGAGGACTA

GTATGAACGGCATCACGAGGGTTATGCTGTCTCCCCACTCTATTCAGTGAAACTGATTTC

CCCGTGAAGAAGCGGGGATATAAATATAAGACGAGAAGACCCCATGGAGCTTTAAACTTA

GTATCAACTGCTTTTTA-AAATAACCTAACATTTAT-GCAA-CCATGAC-TACTAGTTTT

CGGTTGGGGTGACCACGGAGCAAAAATAAACCTCCACGATGAACGGAACTAAATACCTAA

ATCAAGAGTTACAACTCTAAATATCAAT--------AAATTGACTAT--TTGATCCAATT

ACTT--GATCAACGAACCAAGTTACCCTGGGGATAACAGCGCAATCCATTTCAAGAGCTC

ATATCGACAAATGGGTTTACGTACACTCGATGTTGGATCAGGGTATCCCAGTGTGCGCAG

CCGCTATCTATACGGTTC

>M. minuta MN534668, MN534460, MN534561

TTACTATCATCTTTATCTCAACTTACACATGCAAGTATCCGCATCCCCGTGAGAACGCCC

TTTCCCCTT-AATAGGATAAGGAGCTGGTATCAGGCACAG---GCCC-----CCTAGCCC

ATGACACCTAGCTTTGCCACACCCTCAAGGGTATTCAGCAGTGATTAACATTGTTTATAA

GCGCCAGCTTGACTCAGTTAGAGAATTA--AGAGCCGGCCAATACGGTGCCAGCCGCCGC

GGCTACACCGCTAGGCTCAAGTTGATATT-ACTCGGCGTTAAGCGTGTTTAAAGTGCTA-

CAAATAATTAGAATTAAACTTAAACCAAGTTGTGACACGCTTGTTTCTAAGAAAAACATA

AACGAAAGTTATTCTAATTTAACCACTTGAACCCACGACAGCTAGGACACAAACTGGGAT

TAGGTACCCCACTATGCCTAGCCGTAAAATATTTACTTACACCT-TTAACCGCCTGGGAA

TTACGAGCCCAAGCTTAAAACCCAAAGGACTTGACGGTGTCCCACCCACCTAGAGGAGCC

TGTTCTATAA-TCGATTCTCCCC-GATACACCTCACCACTTCTAGCCTC--TCAGCCTGT

ATACCTCCGTCGTAAGCTTACCATATGAACGAT-TTTTAGTGAGCTAAAAGAT-TTACGC

ATAAACACGTCAGGTCAAGGTGCAGCCAACGAAATGGTAAGCAATGGGCTACAATTTCTA

TAAT--TAGAACACA-CGAAAGACTACATGCAA-CTCAGTCATGAAGGCGGATTTAGCAG

TAAAAAGAAAATAGAGAGTTCTTTTTAATTAGGCACTGGGACGCGTACACACCGCCCGTC

ACCCTCTTCAAACGCTCCA-GCTCAG-TTTATAACACC-ACTATGCACCATAGAAGAGGT

AAGTCGTAACATGGTAAGCGTACTGGAAAGTGCGCTTGGATAAA-CAAAATGTAGCTTAA

TTAAA-GCATCTCGCTTACACCGAAAATATGTCTGTGAAACTCAGATCATTTTGA--GCC

AAAAACTTAGCTTAA--CTCCTTCTCAATGCACACC--TTACTTTATAATAAT---TTAA

TAAAACATTTTCCAC-ATTTAGTAAAGGCGATCAAAAAATGTCTA-AAAGCTATAAAA-T

TAGTACCGCAAGGGAACAGTGAAATAAAAATGAAAAA--TCTAAAGCACATAAAAGCAGA

GTCACATCCTCGTACCTTTTGCATCATGGTTTAACTAGTCTAATCAAGCAAAACGAATTT

TAAGTTTGACCCCCCGAAACTAGGTGAGCTACTTAAAAACAGCCTTAT-GGGCAAACCCG

TCTCTGTTGCAAAAGAGTGGGAAGATTCT-TAAGTAGAGGTGACAGACCTATCGAACCTA

GAGATAGCTGGTTATTCAGGAAAAGGATTTAAGTCCTACCTTAGGTTTTTT-TATAC-TC

A-TAAATACCCATA-AACCTTAAGAGCTATTCAAATAAGGTACAGCCTATTTGAAACAGG

ATACAACCTAA---------------------------------------CTAAAAGCAG

CCACCTTCC-AAAAAGCGTTAAAGCTTAATTA---TTTTTATTAAAAA-TTCCACCA-TT

TTTCTACAACCCTT-AATTT-ATACTGAATGATCTCATAAGCTTATGAGAGCCTATATGT

TAAAACTAGTAACAAGAAGA-AGACCTTCTCCA-AAATGTAAGCATGAGCCAAAATGAAC

AATTCATTGGCATTTAAAGTTA-TAAACCTCCAGTAGTAACACT----ACAAGAAAAACC

TAC-----------------TTACTTT-GACGTCAATCTTACACTAGCACATTTCTGGA

AAGATTAAAAGAATGGGAAGGAACTCGGCAAACTA-CTAACCCCGCCTGTTTACCAAAAA

CATCGCCTCTTGCTTCTC----ATAAGAGGTCCAGCCTGCCCAGTGACAAA--GTTAAAC

GGCCGCGGTACCCTAACCGTGCAAAGGTAGCGCAATCACTTGTTCTTTAAATGAGGACTA

GTATGAACGGCATCACGAGGGTTATGCTGTCTCCCCACTCTATTCAGTGAAACTGATTTC

CCCGTGAAGAAGCGGGGATATAAATATAAGACGAGAAGACCCCATGGAGCTTTAAACTTA

GTATCAACTGCTTTTTA-AAATAACCTAACATTTAT-GCAA-CCATGAC-TACTAGTTTT

CGGTTGGGGTGACCACGGAGCAAAAATAAACCTCCACGATGAACGGAACTAAATACCTAA

ATCAAGAGTTACAACTCTAAATATCAAT--------AAATTGACTAT--TTGATCCAATT

ACTT--GATCAACGAACCAAGTTACCCTGGGGATAACAGCGCAATCCATTTCAAGAGCTC

ATATCGACAAATGGGTTTACGTACACTCGATGTTGGATCAGGGTATCCCAGTGTGCGCAG

CCGCTATCTATACGGTTC

>M. minuta MN534669, MN534461, MN534562

TTACTATCATCTTTATCTCAACTTACACATGCAAGTATCCGCATCCCCGTGAGAACGCCC

TTTCCCCTT-AATAGGATAAGGAGCTGGTATCAGGCACAG---GCCC-----CCTAGCCC

ATGACACCTAGCTTTGCCACACCCTCAAGGGTATTCAGCAGTGATTAACATTGTTTATAA

GCGCCAGCTTGACTCAGTTAGAGAATTA--AGAGCCGGCCAATACGGTGCCAGCCGCCGC

GGCTACACCGCTAGGCTCAAGTTGATATT-ACTCGGCGTTAAGCGTGTTTAAAGTGCTA-

CAAATAATTAGAATTAAACTTAAACCAAGTTGTGACACGCTTGTTTCTAAGAAAAACATA

AACGAAAGTTATTCTAATTTAACCACTTGAACCCACGACAGCTAGGACACAAACTGGGAT

TAGGTACCCCACTATGCCTAGCCGTAAAATATTTACTTACACCT-TTAACCGCCTGGGAA

TTACGAGCCCAAGCTTAAAACCCAAAGGACTTGACGGTGTCCCACCCACCTAGAGGAGCC

TGTTCTATAA-TCGATTCTCCCC-GATACACCTCACCACTTCTAGCCTC--TCAGCCTGT

ATACCTCCGTCGTAAGCTTACCATATGAACGAT-TTTTAGTGAGCTAAAAGAT-TTACGC

ATAAACACGTCAGGTCAAGGTGCAGCCAACGAAATGGTAAGCAATGGGCTACAATTTCTA

TAAT--TAGAACACA-CGAAAGACTACATGCAA-CTCAGTCATGAAGGCGGATTTAGCAG

TAAAAAGAAAATAGAGAGTTCTTTTTAATTAGGCACTGGGACGCGTACACACCGCCCGTC

ACCCTCTTCAAACGCTCCA-GCTCAG-TTTATAACACC-ACTATGCACCATAGAAGAGGT

AAGTCGTAACATGGTAAGCGTACTGGAAAGTGCGCTTGGATAAA-CAAAATGTAGCTTAA

TTAAA-GCATCTCGCTTACACCGAAAATATGTCTGTGAAACTCAGATCATTTTGA--GCC

AAAAACTTAGCTTAA--CTCCTTCTCAATGCACACC--TTACTTTATAATAAT---TTAA

TAAAACATTTTCCAC-ATTTAGTAAAGGCGATCAAAAAATGTCTA-AAAGCTATAAAA-T

TAGTACCGCAAGGGAACAGTGAAATAAAAATGAAAAA--TCTAAAGCACATAAAAGCAGA

GTCACATCCTCGTACCTTTTGCATCATGGTTTAACTAGTCTAATCAAGCAAAACGAATTT

TAAGTTTGACCCCCCGAAACTAGGTGAGCTACTTAAAAACAGCCTTAT-GGGCAAACCCG

TCTCTGTTGCAAAAGAGTGGGAAGATTCT-TAAGTAGAGGTGACAGACCTATCGAACCTA

GAGATAGCTGGTTATTCAGGAAAAGGATTTAAGTCCTACCTTAGGTTTTTT-TATAC-TC

A-TAAATACCCATA-AACCTTAAGAGCTATTCAAATAAGGTACAGCCTATTTGAAACAGG

ATACAACCTA--CTAAAAGCAG

CCACCTTCC-AAAAAGCGTTAAAGCTTAATTA---TTTTTATTAAAAA-TTCCACCA-TT

TTTCTACAACCCTT-AATTT-ATACTGAATGATCTCATAAGCTTATGAGAGCCTATATGT

TAAAACTAGTAACAAGAAGA-AGACCTTCTCCA-AAATGTAAGCATGAGCCAAAATGAAC

AATTCATTGGCATTTAAAGTTA-TAAACCTCCAGTAGTAACACT----ACAAGAAAAACC

TAC------------------TTACTTT-GACGTCAATCTTACACTAGCACATTTCTGGA

AAGATTAAAAGAATGGGAAGGAACTCGGCAAACTA-CTAACCCCGCCTGTTTACCAAAAA

CATCGCCTCTTGCTTCTC----ATAAGAGGTCCAGCCTGCCCAGTGACAAA--GTTAAAC

GGCCGCGGTACCCTAACCGTGCAAAGGTAGCGCAATCACTTGTTCTTTAAATGAGGACTA

GTATGAACGGCATCACGAGGGTTATGCTGTCTCCCCACTCTATTCAGTGAAACTGATTTC

CCCGTGAAGAAGCGGGGATATAAATATAAGACGAGAAGACCCCATGGAGCTTTAAACTTA

GTATCAACTGCTTTTTA-AAATAACCTAACATTTAT-GCAA-CCATGAC-TACTAGTTTT

CGGTTGGGGTGACCACGGAGCAAAAATAAACCTCCACGATGAACGGAACTAAATACCTAA

ATCAAGAGTTACAACTCTAAATATCAAT--------AAATTGACTAT--TTGATCCAATT

ACTT--GATCAACGAACCAAGTTACCCTGGGGATAACAGCGCAATCCATTTCAAGAGCTC

ATATCGACAAATGGGTTTACGTACACTCGATGTTGGATCAGGGTATCCCAGTGTGCGCAG

CCGCTATCTATACGGTTC

>M. achatina MN534670, MN534462, MN534563

TTATCATCATCTTTTCCTTGACTTACACATGCAAGTATCAGCACACCCGTGAGAACGCCC

TTTCACCTT-TATAGACTAAGGAGCCGGTATCAGGCACAGG--AATCAAC--CCTAGCCC

ATAACACCTAGCTTTGCCACACCCTCAAGGGTATTCAGCAGTGATTAACATTGTTTATAA

GCGCCAGCTTGATTCAGTCAGAGGACCT--AGAGCCGGCTAATACGGTGCCAGCCGCCGC

GGCTACACCGCTAGGCTCAAGTTGATGTT-ACTCGGCGTTAAGCGTGTTTAAAGTGCCC-

ATCA-GATTAGAGTTAAACTTTAACCAAGCCGTGACACGCTTGTTCCTAAGAAAACCACA

AACGAAAGTTATTCTAACCAAATCACTTGAACCCACGACAGCTAGGGCACAAACTGGGAT

TAGGTACCCCACTATGCCTAGCCGTAAAATATTTACTTACACCT-CTAACCGCCTGGGAA

TTACGAGCCCAAGCTTAAAACCCAAAGGACTTGACGGTGTCCCACCCACCTAGAGGAGCC

TGTTCTATAA-TCGATTCCCCCC-GATACACCTAACCACTCCTAGCCTT--TCAGCCTGT

ATACCTCCGTCGTAAGCTTACCATATGAACGCT-TTTTAGTGAGCTAAAAGAT--TCATC

ATAAATACGTCAGGTCAAGGTGCAGCCAACGAAGTGGCAAGTAATGGGCTACAATTTCTA

CAAC--TAGAACACAACGAAAGACTACATGCAA-CCAAGTCATGAAGGCGGATTTAGAAG

TAAAAAGAAAATAGAGAGTTCTTTTTAATTAGGCACTGGGACGCGTACACACCGCCCGTC

ACCCTCTTCAAATGCTTTT-CCACAG-TATTTAACAAC-TGCCCGCACCACAGAAGAGGT

AAGTCGTAACACGGTAAGCGTACTGGAAAGTGCGCTTGGATAAA-CAAAATGTAGCTTAA

CTAAA-GCATCCCGCTTACACCGAGACTATATCCGTGAAACTCAGATCATTTTGA--GCC

AAAAACTTAGCTTTA--CTCCACCT-AATGCCCCTA--CAAACTTTTAAACA---TAAAA

TAAAACATTTTCTAC-TTTAAGTAAAGGCGATTAAAAAATGTCTT-AAAGCTATAAAA-T

TAGTACCGCAAGGGAATAGTGAAATATTAATGAAAA---CCTCAAGCACTTTAAAGCAGA

GCTAAATTCTCGTACCTTTTGCATCATGGTCTAACTAGTCTAATCAAGCAAAACGAACTT

TAAGTCTGACCCCCCGAAACTAAGCGAGCTACTTAAGAACAGCCAAAT-GGGCGAACCCG

TCTCTGTTGCAAAAGAGTGGGAAGATTTTTTAAGTAGAGGTGATAAACCTACCGAGCTTA

GAGATAGCTGGTTATTCAGGAAAAGGATTTAAGTCCTACCTTAGGTTTCTA-GTATA--C

ACAAATACCCCTCA-AACCTTAAGAGCTATTCAAATAAGGTACAGCCTATTTGAAACAGG

ATACAACCT--AGCAG

CCACCTTT-AAAAAAGCGTTAAAGCTTTATTT--TCCTTTTGTATAAA-TTCCACTA-CC

TCACTTCAACCCAT-AAGCC-ATACTGAATGATTTCATAACTATATGAAAGCCTATATGT

TAGAACTAGTAACAAGAAGA-AGCCCTTCTCCA-AAATGTAAGTATAAGCCAAAATGAAC

TACTCACTGGCAATTAAAGTCAATAAACTATTAGTAGTAACTCA----ACAAGAAAAACC

TAC------------------TAATCAC-AACGTTAACCTTACACCAGCACATTTCTGGA

AAGATTAAAAGGACGGGAAGGAACTCGGCAAACTA-TTAACCCCGCCTGTTTACCAAAAA

CATCGCCTCTTGCTTCCC----ATAAGAGGTCCAGCCTGCCCAGTGACAAA--GTTAAAC

GGCCGCGGTACCCTAACCGTGCAAAGGTAGCGCAATCACTTGTTCTTTAAATGAGGACTA

GTATGAACGGCATCACGAGGGTTATGCTGTCTCCCCGACCCATTCAGTGAAACTGATCTC

CCCGTGAAGAAGCGGGGATAAAAATATAAGACGAGAAGACCCCATGGAGCTTTAAACTCA

GAATCAACTGCTACACA-AAATAACCTAATATTTAT-GCAA-CTCTGAT-TTCTAGTTTT

CGGTTGGGGTGACCACGGAGTAAAAATTAACCTCCACGATGAAAGGAAATAATAACCTAA

ACCAAGAGCTACAGCTCTAAGTATTAAT--------AAATTAACCAAA-CTGATCCAATT

ACTT--GATCAACGAACCAAGTTACCCTGGGGATAACAGCGCAATCCATTTCAAGAGCTC

ATATCGACAAATGGGTTTACG-ACC-TCGATGTTGGATCAGGATATCCCAGTG-GCGCAG

CCGCTA-CTAA-CGGTTC

>M. achatina MN534671, MN534463, MN534564

TTATCATCATCTTTTCCTCGACTTACACATGCAAGTATCAGCACACCCGTGAGAACGCCC

TTTCACCTT-TATAGACTAAGGAGCCGGTATCAGGCACAGG--AATCAAC--CCTAGCCC

ATAACACCTAGCTTTGCCACACCCTCAAGGGTATTCAGCAGTGATTAACATTGTTTATAA

GCGCCAGCTTGATTCAGTCAGAGGACCC--AGAGCCGGCTAATACGGTGCCAGCCGCCGC

GGCTACACCGCTAGGCTCAAGTTGATGTT-ACTCGGCGTTAAGCGTGTTTAAAGTGCCC-

ATCA-GATTAGAGTTAAACTTTAACCAAGCCGTGACACGCTTGTTCCTAAGAAAGCCATA

AACGAAAGTTATTCTAACCAAATCACTTGAATCCACGACAGCTAGGGTACAAACTGGGAT

TAGGTACCCCACTATGCCTAGCCGTAAAATATTTACTTACACCT-TTAACCGCCTGGGAA

TTACGAGCCCAAGCTTAAAACCCAAAGGACTTGACGGTGTCCCACCCACCTAGAGGAGCC

TGTTCTATAA-TCGATTCCCCCC-GATACACCTAACCACTCCTAGCCTC--TCAGCCTGT

ATACCTCCGTCGTAAGCTTACCATATGAACGCT-TTTTAGTGAGCTAAAAGAT---CCTC

ATAAATACGTCAGGTCAAGGTGCAGCCAACGAAGTGGCAAGTAATGGGCTACAATTTCTA

CAAC--TAGAACACAACGAAAGACTACATGCAA-CCAAGTCATGAAGGCGGATTTAGAAG

TAAAAAGAAAATAGAGAGTTCTTTTTAATTAGGCACTGGGACGCGTACACACCGCCCGTC

ACCCTCTTCAAATGCTTTC-CTACAG-TACTTAACAAC-TGCCCGCACCACAGAAGAGGT

AAGTCGTAACACGGTAAGCGTACTGGAAAGTGCGCTTGGATAAA-CAAAATGTAGCTTAA

CTAAA-GCATCCCGCTTACACCGAGACTATATCCGTGAAACTCAGATCATTTTGA--GCC

AAAAACTTAGCTTTA--CTCCACCT-AATGCCCCCA--CAAACTTTTAAACA---TAAGA

TAAAACATTTTCTAC-TTTAAGTAAAGGCGATTAAAAAATGTCTT-AAAGCTATAAAA-T

TAGTACCGCAAGGGAATAGTGAAATATTAATGAAAA---CCTCAAGCACTTTAAAGCAGA

GCTAAATTCTCGTACCTTTTGCATCATGGTCTAACTAGTCTAATCAAGCAAAACGAACTT

TAAGTCTGACCCCCCGAAACTAAGCGAGCTACTTAAGAACAGCCAAAT-GGGCCAACCCG

TCTCTGTTGCAAAAGAGTGGGAAGATTTT-TAAGTAGGGGTGATAAACCTACCGAGCTTA

GAGATAGCTGGTTATTCAGGAAAAGGATTTAAGTCCTACCTTAGGTTTCTC-GTATA--T

ACAAATACCCCTCA-AACCTTAAGAGCTATTCAAATAAGGTACAGCCTATTTGAAACAGG

ATACAACCT--AGCAG

CCACCTTT-AAAAAAGCGTTAAAGCTTTATTT--TCCTTTTGTATAAA-TTCCACTA-CC

TTACTACAACCCAT-AAACC-ATACTGAATGATTTCATAACTGTATGAAAGCCTATATGT

TAGAACTAGTAACAAGAAGA-AGCCCTTCTCCA-AAATGTAAGTATAAGCCAAAATGAAC

TACTCACTGGCAATTAAAGTCAATAAACTATTAGTAGTAACTCA----ACAAGAAAAACC

TAC------------------TAATCAC-AACGTTAACCTTACACCAGCACATTTCTGGA

AAGATTAAAAGGACGGGAAGGAACTCGGCAAACTA-CTAACCCCGCCTGTTTACCAAAAA

CATCGCCTCTTGCTTCCC----ATAAGAGGTCCAGCCTGCCCAGTGACAAA--GTTAAAC

GGCCGCGGTACCCTAACCGTGCAAAGGTAGCGCAATCACTTGTTCTTTAAATGAGGACTA

GTATGAACGGCATCACGAGGGTTATGCTGTCTCCCCGCCCCATTCAGTGAAACTGATCTC

CCCGTGAAGAAGCGGGGATAAAAATATAAGACGAGAAGACCCCATGGAGCTTTAAACTCA

AAATCAACTGCTACACA-AAATAACCTAATATTCAT-GCAA-CTCTGAT-TTCTAGTTTT

CGGTTGGGGTGACCACGGAGTAAAAATTAACCTCCACGATGAAAGGAAATAATAACCTAA

ACCAAGAGCTACAGCTCTAAGTATTAAT--------AAATTAACCAAA-CTGATCCAATT

ACTT--GATCAACGAACCAAGTTACCCTGGGGATAACAGCGCAATCCATTTCAAGAGCTC

ATATCGACAAATGGGTTTACG-ACC-TCGATGTTGGATCAGGATATCCCAGTG-GCGCAG

CCGCTA-CTAA-CGGTTC

>M. achatina MN534672, MN534464, MN534565

TTATCATCATCTTTTCCTCGACTTACACATGCAAGTATCAGCACACCCGTGAGAACGCCC

TTTCACCTT-TATAGACTAAGGAGCCGGTATCAGGCACAGG--AATCAAC--CCTAGCCC

ATAACACCTAGCTTTGCCACACCCTCAAGGGTATTCAGCAGTGATTAACATTGTTTATAA

GCGCCAGCTTGATTCAGTCAGAGGACCC--AGAGCCGGCTAATACGGTGCCAGCCGCCGC

GGCTACACCGCTAGGCTCAAGTTGATGTT-ACTCGGCGTTAAGCGTGTTTAAAGTGCCC-

ATCA-GATTAGAGTTAAACTTTAACCAAGCCGTGACACGCTTGTTCCTAAGAAAGCCATA

AACGAAAGTTATTCTAACCAAATCACTTGAATCCACGACAGCTAGGGTACAAACTGGGAT

TAGGTACCCCACTATGCCTAGCCGTAAAATATTTACTTACACCT-TTAACCGCCTGGGAA

TTACGAGCCCAAGCTTAAAACCCAAAGGACTTGACGGTGTCCCACCCACCTAGAGGAGCC

TGTTCTATAA-TCGATTCCCCCC-GATACACCTAACCACTCCTAGCCTC--TCAGCCTGT

ATACCTCCGTCGTAAGCTTACCATATGAACGCT-TTTTAGTGAGCTAAAAGAT--TCCTC

ATAAATACGTCAGGTCAAGGTGCAGCCAACGAAGTGGCAAGTAATGGGCTACAATTTCTA

CAAC--TAGAACACAACGAAAGACTACATGCAA-CCAAGTCATGAAGGCGGATTTAGAAG

TAAAAAGAAAATAGAGAGTTCTTTTTAATTAGGCACTGGGACGCGTACACACCGCCCGTC

ACCCTCTTCAAATGCTTTC-CTACAG-TACTTAACAAC-TGCCCGCACCACAGAAGAGGT

AAGTCGTAACACGGTAAGCGTACTGGAAAGTGCGCTTGGATAAA-CAAAATGTAGCTTAA

CTAAA-GCATCCCGCTTACACCGAGACTATATCCGTGAAACTCAGATCATTTTGA--GCC

AAAAACTTAGCTTTA--CTCCACCT-AATGCCCCCA--CAAACTTTTAAACA---TAAGA

TAAAACATTTTCTAC-TTTAAGTAAAGGCGATTAAAAAATGTCTT-AAAGCTATAAAA-T

TAGTACCGCAAGGGAATAGTGAAATATTAATGAAAA---CCTCAAGCACTTTAAAGCAGA

GCTAAATTCTCGTACCTTTTGCATCATGGTCTAACTAGTCTAATCAAGCAAAACGAACTT

TAAGTCTGACCCCCCGAAACTAAGCGAGCTACTTAAGAACAGCCAAAT-GGGCCAACCCG

TCTCTGTTGCAAAAGAGTGGGAAGATTTTTTAAGTAGGGGTGATAAACCTACCGAGCTTA

GAGATAGCTGGTTATTCAGGAAAAGGATTTAAGTCCTACCTTAGGTTTCTC-GTATA--T

ACAAATACCCCTCA-AACCTTAAGAGCTATTCAAATAAGGTACAGCCTATTTGAAACAGG

ATACAACCT--CCTAGCAG

CCACCTTT-AAAAAAGCGTTAAAGCTTTATTT--TCCTTTTGTATAAA-TTCCACTA-CC

TTACTACAACCCAT-AAACC-ATACTGAATGATTTCATAACTGTATGAAAGCCTATATGT

TAGAACTAGTAACAAGAAGA-AGCCCTTCTCCA-AAATGTAAGTATAAGCCAAAATGAAC

TACTCACTGGCAATTAAAGTCAATAAACTATTAGTAGTAACTCA----ACAAGAAAAACC

TAC-----------------TAATCAC-AACGTTAACCTTACACCAGCACATTTCTGGA

AAGATTAAAAGGACGGGAAGGAACTCGGCAAACTA-CTAACCCCGCCTGTTTACCAAAAA

CATCGCCTCTTGCTTCCC----ATAAGAGGTCCAGCCTGCCCAGTGACAAA--GTTAAAC

GGCCGCGGTACCCTAACCGTGCAAAGGTAGCGCAATCACTTGTTCTTTAAATGAGGACTA

GTATGAACGGCATCACGAGGGTTATGCTGTCTCCCCGCCCCATTCAGTGAAACTGATCTC

CCCGTGAAGAAGCGGGGATAAAAATATAAGACGAGAAGACCCCATGGAGCTTTAAACTCA

AAATCAACTGCTACACA-AAATAACCTAATATTCAT-GCAA-CTCTGAT-TTCTAGTTTT

CGGTTGGGGTGACCACGGAGTAAAAATTAACCTCCACGATGAAAGGAAATAATAACCTAA

ACCAAGAGCTACAGCTCTAAGTATTAAT--------AAATTAACCAAA-CTGATCCAATT

ACTT--GATCAACGAACCAAGTTACCCTGGGGATAACAGCGCAATCCATTTCAAGAGCTC

ATATCGACAAATGGGTTTACG-ACC-TCGATGTTGGATCAGGATATCCCAGTG-GCGCAG

CCGCTA-CTAA-CGGTTC

>M. pineticola MN534673, MN534465, MN534566

TTATTATCAACTCCCTCTCGACTTACACATGCAAGTATCAGCACACCCGTGAGAACGCCC

TTCACCCTT-AATAGGATAAGGAGCTGGTATCAGGCTCAG---TTT------TCTAGCCC

ACGACACCTAGCTTTGCCACACCCTCAAGGGCATTCAGCAGTGATTAACATTGTTTATAA

GCGCCAGCTTGATTCAGTTAGAGAATTT--AGAGCCGGCTAATATGGTGCCAGCCGCCGC

GGCTACACCACTGGGCTCAAGTTGATTTT-ATTCGGCGTTAAGCGTGTTTAAAGTGCAC-

ACAA-GATTAGAATTGAACTTAAACCAAACAGTGATACGTTTGTTTTTAAGAAAGACAAA

AACGAAAGTTATTCTAACAAAACCACTTGAACCCACGACAGCTGGGGCACAAACTGGGAT

TAGGTACCCCACTATGCCCAGCCGTAAAACATCTATTCACACCT-TAAACCGCCCGGGAA

TTACGAGCTCAAGCTTAAAACCCAAAGGACTTGACGGTGTCCCACCCACCTAGAGGAGCC

TGTTCTATAA-TCGATTATCCAC-GATTCACCTCACCACTTCTAGCTTT--TCAGCCTGT

ATACCTCCGTCGTAAGCTCACCGTATGAACGGT-TTTTAGTGAGCTAAAAGAT-TTTTAC

GTAAATACGTCAGGTCAAGGTGCAGCCAACGAAGTGGCAAGCAATGGGCTACAATTTCTA

CACT--TAGAACATAACGAAAGACCGCATGAAA-TACGGTCATGAAGGTGGATTTAGTAG

TAAAAAGAAAATAGAGAGTTCTTTTTAACCAGGCACTGGGACGCGTACACACCGCCCGTC

ACCCTCTTCAAACGCCTTA-ATTCAG-TACATAACAAG-TACCCGCATCACAGAAGAGGT

AAGTCGTAACACGGTAAGCGTACTGGAAAGTGCGCTTGGATAAA-CAAAATGTAGCTTAA

TTAAA-GCATCTCGCTTACACCGAGAATATGTCTGTTAAACTCGGATCATCTTGA--GCT

AAAAACTTAGCTTAA--TCTCTTCTA-ATGTACAACC--CTTAAAACCCGCAA--CTAAG

TAAAACATTTTCTAC-ATTTAGTAAAGGCGATCAAAAAATGTCTT-AAAGCTATACAA-T

TAGTACCGCAAGGGAAAAGTGAAATAGCAATGAAAAA--CCTCAAGCACATAAAAGCAGA

GATTCAATCTTGTACCTTTTGCATCATGGTTTAACTAGTCTAATCAAGCAAAATGATTTT

TAAGTCTGACCCCCCGAAACTAGGTGAGCTACTTAAAAACAGCCTTAT-GGGCGAACCCG

TCTCTGTTGCAAAAGAGTGGGAAGATTTT-TAAGTAGGGGTGATAAGCCTATCGAACCTA

GAGATAGCTGGTTATTCAGGAAAAGGATTTAAGTCCTACCTTAGGTTTTTC-GTATA-TT

T-AAATACACCTCT-AACCTTAAGAGTCATTCAAATAAGGTACAGCCTATT-GAAACAGG

ATACAACCTCATCT-----------------------------AGTGGGCCCTAAAGCAG

CCACCTTTT-AAAAAGCGTTAAAGCTTTATTTAA-TTTTCTATAAAAA-TTCCACTA-AT

TATCTGTAACCCTC-AACCT-GTACTGAATGATTTCATAATTTTATGAAAACCTATATGT

TAGAACTAGTAACAAGAAGGAAGCCCTTCTCCAAAAATGTAAGCATGAACCAAAATGAAC

CACTCATTGGTATTTAAAGTTAATAAACCTCAAGTAGCAACTTA----ACAAGAAAAAAC
TAT------------------TTATTAT-AGCGTCAATCTTACACCAGCACATTTCCGGA
AAGATTAAAAGAATGGGAAGGAACTCGGCAAACAA-CTGACCTCGCCTGTTTACCAAAAA
CATCGCCTCCTGCATTTT----ATAGGAGGTCCAGCCTGCCCAGTGACAAA--GTTAAAC
GGCCGCGGTACCCTAACCGTGCAAAGGTAGCGCAATCATTTGTTCTTTAAATGAGGACTA
GTATGAATGGCATCACGAAGGTTATGCTGTCTCCCCACTCTATTCAGTGAAACTGATCTC
CCCGTGAAGAAGCGGGGATAAAATTATAAGACGAGAAGACCCCATGGAGCTTTAAACTCA
GTAACAACTGTTATTTT---TAAGCCTGATATTTAT-GCAG-CCATGAT-TACTAGTTTT
CGGTTGGGGTGACCACGGAGTAAAACCTAACCTCCACGATGAAAGGAAGTACAAACCTAA
ATCAAGAGCGACAGCTCCAAATATCAAC--------AAATTGACTAAA-TTGACCCAATT
ACTT--GATCAACGAACTAAGTTACCCTGGGGATAACAGCGCAATCCATTTCAAGAGCTC
ATATCGACAAATGGGTTTACG-ACC-TCGATGTTGGATCAGGGTATCCCAGTG-GCGCAG
TCACTA-CTAA-CGGTTC

>M. pineticola MN534674, MN534466, MN534567

TTATTATCAACTCCCTCTCGACTTACACATGCAAGTATCAGCACACCCGTGAGAACGCCC
TTCACCCTT-AATAGGATAAGGAGCTGGTATCAGGCTCAG---TTT------TCTAGCCC
ACGACACCTAGCTTTGCCACACCCTCAAGGGCATTCAGCAGTGATTAACATTGTTTATAA
GCGCCAGCTTGATTCAGTTAGAGAATTT--AGAGCCGGCTAATATGGTGCCAGCCGCCGC
GGCTACACCACTGGGCTCAAGTTGATTTT-ATTCGGCGTTAAGCGTGTTTAAAGTGCAC-
ACAA-GATTAGAATTGAACTTAAACCAAACAGTGATACGTTTGTTTTTAAGAAAGACAAA
AACGAAAGTTATTCTAACAAAACCACTTGAACCCACGACAGCTGGGGCACAAACTGGGAT
TAGGTACCCCACTATGCCCAGCCGTAAAACATCTATTCACACCT-TAAACCGCCCGGGAA
TTACGAGCTCAAGCTTAAAACCCAAAGGACTTGACGGTGTCCCACCCACCTAGAGGAGCC
TGTTCTATAA-TCGATTATCCAC-GATTCACCTCACCACTTCTAGCTTT--TCAGCCTGT
ATACCTCCGTCGTAAGCTCACCGTATGAACGGT-TTTTAGTGAGCTAAAAGAT-TTTTAC
GTAAATACGTCAGGTCAAGGTGCAGCCAACGAAGTGGCAAGCAATGGGCTACAATTTCTA
CACT--TAGAACATAACGAAAGACCGCATGAAA-TACGGTCATGAAGGTGGATTTAGTAG
TAAAAAGAAAATAGAGAGTTCTTTTTAACCAGGCACTGGGACGCGTACACACCGCCCGTC
ACCCTCTTCAAACGCCTTA-ATTCAG-TACATAACAAG-TACCCGCATCACAGAAGAGGT
AAGTCGTAACACGGTAAGCGTACTGGAAAGTGCGCTTGGATAAA-CAAAATGTAGCTTAA

TTAAA-GCATCTCGCTTACACCGAGAATATGTCTGTTAAACTCGGATCATCTTGA--GCT

AAAAACTTAGCTTAA--TCTCTTCTA-ATGTACAACC--CTTAAAACCCGCAA--CTAAG

TAAAACATTTTCTAC-ATTTAGTAAAGGCGATCAAAAAATGTCTT-AAAGCTATACAA-T

TAGTACCGCAAGGGAAAAGTGAAATAGCAATGAAAAA--CCTCAAGCACATAAAAGCAGA

GATTCAATCTTGTACCTTTTGCATCATGGTTTAACTAGTCTAATCAAGCAAAATGATTTT

TAAGTCTGACCCCCCGAAACTAGGTGAGCTACTTAAAAACAGCCTTAT-GGGCGAACCCG

TCTCTGTTGCAAAAGAGTGGGAAGATTTT-TAAGTAGGGGTGATAAGCCTATCGAACCTA

GAGATAGCTGGTTATTCAGGAAAAGGATTTAAGTCCTACCTTAGGTTTTTC-GTATA-TT

T-AAATACACCTCT-AACCTTAAGAGTCATTCAAATAAGGTACAGCCTATT-GAAACAGG

ATACAACCTCATCT-----------------------------AGTGGGCCCTAAAGCAG

CCACCTTTT-AAAAAGCGTTAAAGCTTTATTTAA-TTTTCTATAAAAA-TTCCACTA-AT

TATCTGTAACCCTC-AACCT-GTACTGAATGATTTCATAATTTTATGAAAACCTATATGT

TAGAACTAGTAACAAGAAGGAAGCCCTTCTCCA-AAATGTAAGCATGAACCAAAATGAAC

CACTCATTGGTATTTAAAGTTAATAAACCTCAAGTAGCAACTTA----ACAAGAAAAAAC

TAT------------------TTATTAT-AGCGTCAATCTTACACCAGCACATTTCCGGA

AAGATTAAAAGAATGGGAAGGAACTCGGCAAACAA-CTGACCTCGCCTGTTTACCAAAAA

CATCGCCTCCTGCATTTT----ATAGGAGGTCCAGCCTGCCCAGTGACAAA--GTTAAAC

GGCCGCGGTACCCTAACCGTGCAAAGGTAGCGCAATCATTTGTTCTTTAAATGAGGACTA

GTATGAATGGCATCACGAAGGTTATGCTGTCTCCCCACTCTATTCAGTGAAACTGATCTC

CCCGTGAAGAAGCGGGGATAAAATTATAAGACGAGAAGACCCCATGGAGCTTTAAACTCA

GTAACAACTGTTATTTT---TAAGCCTGATATTCAT-GCAG-CCATGAT-TACTAGTTTT

CGGTTGGGGTGACCACGGAGTAAAACCTAACCTCCACGATGAAAGGAAGTACAAACCTAA

ATCAAGAGCGACAGCTCCAAATATCAAC--------AAATTGACTAAA-TTGACCCAATT

ACTT--GATCAACGAACTAAGTTACCCTGGGGATAACAGCGCAATCCATTTCAAGAGCTC

ATATCGACAAATGGGTTTACG-ACC-TCGATGTTGGATCAGGGTATCCCAGTG-GCGCAG

TCACTA-CTAA-CGGTTC

>M. pineticola MN534675, MN534467, MN534568

TTATTATCAACTCCCTCTCGACTTACACATGCAAGTATCAGCACACCCGTGAGAACGCCC

TTCACCCTT-AATAGGATAAGGAGCTGGTATCAGGCTCAGG---TTT------TCTAGCCC

ACGACACCTAGCTTTGCCACACCCTCAAGGGCATTCAGCAGTGATTAACATTGTTTATAA

GCGCCAGCTTGATTCAGTTAGAGAATTT--AGAGCCGGCTAATATGGTGCCAGCCGCCGC

GGCTACACCACTGGGCTCAAGTTGATTTT-ATTCGGCGTTAAGCGTGTTTAAAGTGCAC-

ACAA-GATTAGAATTGAACTTAAACCAAACAGTGATACGTTTGTTTTTAAGAAAGACAAA

AACGAAAGTTATTCTAACAAAACCACTTGAACCCACGACAGCTGGGGCACAAACTGGGAT

TAGGTACCCCACTATGCCCAGCCGTAAAACATCTATTCACACCT-TAAACCGCCCGGGAA

TTACGAGCTCAAGCTTAAAACCCAAAGGACTTGACGGTGTCCCACCCACCTAGAGGAGCC

TGTTCTATAA-TCGATTATCCAC-GATTCACCTCACCACTTCTAGCTTT--TCAGCCTGT

ATACCTCCGTCGTAAGCTCACCGTATGAACGGT-TTTTAGTGAGCTAAAAGAT-TTTTAC

GTAAATACGTCAGGTCAAGGTGCAGCCAACGAAGTGGCAAGCAATGGGCTACAATTTCTA

CACT--TAGAACATAACGAAAGACCGCATGAAA-TACGGTCATGAAGGTGGATTTAGTAG

TAAAAAGAAAATAGAGAGTTCTTTTTAACCAGGCACTGGGACGCGTACACACCGCCCGTC

ACCCTCTTCAAACGCCTTA-ATTCAG-TACATAACAAG-TACCCGCATCACAGAAGAGGT

AAGTCGTAACACGGTAAGCGTACTGGAAAGTGCGCTTGGATAAA-CAAAATGTAGCTTAA

TTAAA-GCATCTCGCTTACACCGAGAATATGTCTGTTAAACTCGGATCATCTTGA--GCT

AAAAACTTAGCTTAA--TCTCTTCTA-ATGTACAACC--CTTAAAACCCGCAA--CTAAG

TAAAACATTTTCTAC-ATTTAGTAAAGGCGATCAAAAAATGTCTT-AAAGCTATACAA-T

TAGTACCGCAAGGGAAAAGTGAAATAGCAATGAAAAA--CCTCAAGCACATAAAAGCAGA

GATTCAATCTTGTACCTTTTGCATCATGGTTTAACTAGTCTAATCAAGCAAAATGATTTT

TAAGTCTGACCCCCCGAAACTAGGTGAGCTACTTAAAAACAGCCTTAT-GGGCGAACCCG

TCTCTGTTGCAAAAGAGTGGGAAGATTTT-TAAGTAGGGGTGATAAGCCTATCGAACCTA

GAGATAGCTGGTTATTCAGGAAAAGGATTTAAGTCCTACCTTAGGTTTTTC-GTATA-TT

T-AAATACACCTCT-AACCTTAAGAGTCATTCAAATAAGGTACAGCCTATT-GAAACAGG

ATACAACCTCATCT-----------------------------AGTGGGCCCTAAAGCAG

CCACCTTTT-AAAAAGCGTTAAAGCTTTATTTAA-TTTTCTATAAAAA-TTCCACTA-AT

TATCTGTAACCCTC-AACCT-GTACTGAATGATTTCATAATTTTATGAAAACCTATATGT

TAGAACTAGTAACAAGAAGGAAGCCCTTCTCCA-AAATGTAAGCATGAACCAAAATGAAC

CACTCATTGGTATTTAAAGTTAATAAACCTCAAGTAGCAACTTA----ACAAGAAAAAAC

TAT------------------TTATTAT-AGCGTCAATCTTACACCAGCACATTTCCGGA

AAGATTAAAAGAATGGGAAGGAACTCGGCAAACAA-CTGACCTCGCCTGTTTACCAAAAA

CATCGCCTCCTGCATTTT----ATAGGAGGTCCAGCCTGCCCAGTGACAAA--GTTAAAC

GGCCGCGGTACCCTAACCGTGCAAAGGTAGCGCAATCATTTGTTCTTTAAATGAGGACTA

GTATGAATGGCATCACGAAGGTTATGCTGTCTCCCCACTCTATTCAGTGAAACTGATCTC

CCCGTGAAGAAGCGGGGATAAAATTATAAGACGAGAAGACCCCATGGAGCTTTAAACTCA

GTAACAACTGTTATTTT---TAAGCCTGATATTTAT-GCAG-CCATGAT-TACTAGTTTT

CGGTTGGGGTGACCACGGAGTAAAACCTAACCTCCACGATGAAAGGAAGTACAAACCTAA

ATCAAGAGCGACAGCTCCAAATATCAAC--------AAATTGACTAAA-TTGACCCAATT

ACTT--GATCAACGAACTAAGTTACCCTGGGGATAACAGCGCAATCCATTTCAAGAGCTC

ATATCGACAAATGGGTTTACG-ACC-TCGATGTTGGATCAGGGTATCCCAGTG-GCGCAG

TCACTA-CTAA-CGGTTC

>M. pineticola MN534676, MN534468, MN534569

TTATTATCAACTCCCTCTCGACTTACACATGCAAGTATCAGCACACCCGTGAGAACGCCC

TTCACCCTT-AATAGGATAAGGAGCTGGTATCAGGCTCAG---TTT------TCTAGCCC

ACGACACCTAGCTTTGCCACACCCTCAAGGGCATTCAGCAGTGATTAACATTGTTTATAA

GCGCCAGCTTGATTCAGTTAGAGAATTT--AGAGCCGGCTAATATGGTGCCAGCCGCCGC

GGCTACACCACTGGGCTCAAGTTGATTTT-ATTCGGCGTTAAGCGTGTTTAAAGTGCAC-

ACAA-GATTAGAATTGAACTTAAACCAAACAGTGATACGTTTGTTTTTAAGAAAGACAAA

AACGAAAGTTATTCTAACAAAACCACTTGAACCCACGACAGCTGGGGCACAAACTGGGAT

TAGGTACCCCACTATGCCCAGCCGTAAAACATCTATTCACACCT-TAAACCGCCCGGGAA

TTACGAGCTCAAGCTTAAAACCCAAAGGACTTGACGGTGTCCCACCCACCTAGAGGAGCC

TGTTCTATAA-TCGATTATCCAC-GATTCACCTCACCACTTATAGCTTT--TCAGCCTGT

ATACCTCCGTCGTAAGCTCACCGTATGAACGGT-TTTTAGTGAGCTAAAAGAT-TTTTAC

GTAAATACGTCAGGTCAAGGTGCAGCCAACGAAGTGGCAAGCAATGGGCTACAATTTCTA

CACT--TAGAACATAACGAAAGACCGCATGAAA-TACGGTCATGAAGGTGGATTTAGTAG

TAAAAAGAAAATAGAGAGTTCTTTTTAACCAGGCACTGGGACGCGTACACACCGCCCGTC

ACCCTCTTCAAACGCCTTA-ATTCAG-TACATAACAAG-TACCCGCATCACAGAAGAGGT

AAGTCGTAACACGGTAAGCGTACTGGAAAGTGCGCTTGGATAAA-CAAAATGTAGCTTAA

TTAAA-GCATCTCGCTTACACCGAGAATATGTCTGTTAAACTCGGATCATCTTGA--GCT

AAAAACTTAGCTTAA--TCTCTTCTA-ATGTACAACC--CTTAAAACCCGCAA--CTAAG

TAAAACATTTTCTAC-ATTTAGTAAAGGCGATCAAAAAATGTCTT-AAAGCTATACAA-T

TAGTACCGCAAGGGAAAAGTGAAATAGCAATGAAAAA--CCTCAAGCACATAAAAGCAGA

GATTCAATCTTGTACCTTTTGCATCATGGTTTAACTAGTCTAATCAAGCAAAATGATTTT

TAAGTCTGACCCCCCGAAACTAGGTGAGCTACTTAAAAACAGCCTTAT-GGGCGAACCCG

TCTCTGTTGCAAAAGAGTGGGAAGATTTT-TAAGTAGGGGTGATAAGCCTATCGAACCTA

GAGATAGCTGGTTATTCAGGAAAAGGATTTAAGTCCTACCTTAGGTTTTTC-GTATA-TT

T-AAATACACCTCT-AACCTTAAGAGTCATTCAAATAAGGTACAGCCTATT-GAAACAGG

ATACAACCTCATCT-----------------------------AGTGGGCCCTAAAGCAG

CCACCTTTT-AAAAAGCGTTAAAGCTTTATTTAA-TTTTCTATAAAAA-TTCCACTA-AT

TATCTGTAACCCTC-AACCT-GTACTGAATGATTTCATAATTTTATGAAAACCTATATGT

TAGAACTAGTAACAAGAAGGAAGCCCTTCTCCA-AAATGTAAGCATGAACCAAAATGAAC

CACTCATTGGTATTTAAAGTTAATAAACCTCAAGTAGCAACTTA----ACAAGAAAAAAC

TAT------------------TTATTAT-AGCGTCAATCTTACACCAGCACATTTCCGGA

AAGATTAAAAGAATGGGAAGGAACTCGGCAAACAA-CTGACCTCGCCTGTTTACCAAAAA

CATCGCCTCCTGCATTTT----ATAGGAGGTCCAGCCTGCCCAGTGACAAA--GTTAAAC

GGCCGCGGTACCCTAACCGTGCAAAGGTAGCGCAATCATTTGTTCTTTAAATGAGGACTA

GTATGAATGGCATCACGAAGGTTATGCTGTCTCCCCACTCTATTCAGTGAAACTGATCTC

CCCGTGAAGAAGCGGGGATAAAATTATAAGACGAGAAGACCCCATGGAGCTTTAAACTCA

GTAACAACTGTTATTTT---TAAGCCTGATATTTAT-GCAG-CCATGAT-TACTAGTTTT

CGGTTGGGGTGACCACGGAGTAAAACCTAACCTCCACGATGAAAGGAAGTACAAACCTAA

ATCAAGAGCGACAGCTCCAAATATCAAC--------AAATTGACTAAA-TTGACCCAATT

ACTT--GATCAACGAACTAAGTTACCCTGGGGATAACAGCGCAATCCATTTCAAGAGCTC

ATATCGACAAATGGGTTTACG-ACC-TCGATGTTGGATCAGGGTATCCCAGTG-GCGCAG

TCACTA-CTAA-CGGTTC

>M. heymonsi MN534677, MN534469, MN534570

TTATTATCAACTTTTTCTTGATTTACACATGCAAGTATCAGCACACCCGTGAGAACGCCC

TTTACCCTT-TATAGGACAAGGAGCTGGTATCAGGCTCAG---TCT------TCTAGCCC

ATAACACCTAGCTTTGCCACACCCTCAAGGGTATTCAGCAGTGATTAACATTGTATATAA

GCGCCAGCTTGACCCAGTCAAAGATCCT--AGAGCCGGCTAATACGGTGCCAGCCGCCGC

GGCTACACCGCTAGGCTCAAGTTGATATT-AATCGGCGTTAAGCGTGTTTAAAGTGCAC-

AAGA-GATTAGAATTAAACTCAAACCAAGCTGTGACACGCTTGTTTTTAAGAAAAACAAA

AACGAAAGTTATTCTAACCAAACCACTTGAATCCACGACAGCTAGGACACAAACTGGGAT

TAGGTACCCCACTATGCCTAGCCGTAAAATATTTATTTACACCT-TTAACCGCCTGGGAA

TTACGAGCCCAAGCTTAAAACCCAAAGGACTTGACGGTGCCCCACCCACCTAGAGGAGCC

TGTTCTATAA-TCGATTCTCCCC-GATATACCTCACCACTTCTAGCCTA--TCAGCCTGT

ATACCTCCGTCGTAAGCTTACCATATGAACGCT-TTTTAGTGAGCTAAAAGAT--TTTAC

ATAAATACGTCAGGTCAAGGTGCAGCCCACGAAGTGGAAAGAAATGGGCTACAATTTCTA

AAA---TAGAACACAACGAAAGACTACATGCAA-CTTAGTCATGAAGGCGGATTTAGTAG

TAAAAAGAAAATAGAGTGTTCTTTTTAATTAGGCGCTGGGACGCGTACACACCGCCCGTC

ACCCTCTTCAAACGCCTTA--TCAAG-TTTATAACAAC-ACCACGCATTACAGAAGAGGT

AAGTCGTAACACGGTAAGCGTACTGGAAAGTGCGCTTGGATAAA-CAAAATGTAGCTTAA

TCAAA-GCATCTCGCTTACACCGAGAACATATCTGTGATACCCGGATCATTTTGA--GCT

AAAAACTTAGCTTTA--CTTCTCTCCAATGTTTACCC-GCGCCTC--AAATAA--CAAAA

TAAAACATTTTACAT-ATTAAGTAAAGGCGATTAAAAAATATCTT-AAAGCTATAAAA-C

AAGTACCGCAAGGGAATAGTGAAATAATAATGAAAAA---CTAAAGCACATAAAAGCAGA

GACTCAACCTCGTACCTTTTGCATCATGGTCTAACTAGTCCCTTCGAGCAAAATGAGTTT

TAAGTTTGATCCCCCGAAACTAGGTGAGCTACTTAAAAACAGCCTTCT-GGGCCAACCCG

TCTCTGTTGCAAAAGAGTGGGAAGATTTT-TAAGTAGAGGTGATAAACCTACCGAACCTA

GAGATAGCTGGTTATTCAGGAAAAGGATTTAAGTCCTACCTTATGTTTTTT-GTACA--T

ACAAGTACCCCATC-AACCCTT--------------------------------------

---AGCAG

CCACCTTT-AAGAAAGCGTTAAAGCTTCATTA---TATTTTATAAAAA-TTTCGCTA-AT

TTTCTAAAACCCTT-AACCT-GTACTGAATGGTCTCATGACTTCATGAGAGTCTATATGT

TAGAACTAGTAACAAGAAGA-AGCCCTTCTCCA-AAATGTAAGCATGAGCCAAAATGAAC

TATTCATTGGCATTTAAAGTTAATAATCTTCTAGTAGTAACTGC----TCAAGAAAAATC

TAC------------------TATTCTT-AACGTCAACCTTACACCAGCACATTTCTGGA

AAGATTAAAAGACTAGGAAGGAACTCGGCAAACAA-TTAACCCCGCCTGTTTACCAAAAA

CATCGCCTCCTGATTTTT----ATAGGAGGTCTAGCCTGCCCAGTGACAAA--GTTAAAC

GGCCGCGGTACCCTAACCGTGCAAAGGTAGCGCAATCACTTGTTCTTTAAATGAGGACTA

GTATGAACGGCATCACGAGGGTTATGCTGTCTCCCTACTTTATTCAGTGAAACTGATCTC

CCCGTGAAGAAGCGGGGATAAAAATATAAGACGAGAAGACCCCATGGAGCTTTAAACTCA

GTGTCAACTGCTATGTT--TAAAACCTAATATACAA-GCAATTTCTGAC-TACTAGTTTT

CGGTTGGGGTGACCACGGAGTAAAACCTAACCTCCACGATGAAAGGAAATAACAACCTAA

ATTAAGAGCTACAACTCTAAATATCAAT--------AAATTGACTGA--TTGATCCAATT

AATT--GATCAACGAACCAAGTTACCCTGGGGATAACAGCGCAATCCATTTCAAGAGCTC

CTATCGACAAATGGGTTTACG-ACC-TCGATGTTGGATCAGGGTATCCCAGTG-GCGCAG

CTGCTA-CTAA-TGGTTC

>M. heymonsi MN534678, MN534470, MN534571

TTATTATCAACTTTTTCTTAATTTACACATGCAAGTTTCAGCACACCCGTGAGAACGCCC

TTTACCCTT-AATAGGACAAGGAGCTGGTATCAGGCTCAG---TCT------TCTAGCCC

ATAACACCTAGCTTTGCCACACCCTCAAGGGTATTCAGCAGTGATTAACATTGTTTATAA

GCGCCAGCTTGAACCAGTTAAAGAACAT--AGAGCCGGCCAATACGGTGCCAGCCGCCGC

GGCTACACCGCTAGGCTCAAGTTGATATT-AACCGGCGTTAAGCGTGTTTAAAGTGCAC-

AAAA-GATTAGAATTGAACTTAAACCAAGCTGTGACACGCTTGTTTTTAAGAAAAACATA

AACGAAAGTTATTCTAACCAAACCACTTGAATCCACGACAGCTAGGACACAAACTGGGAT

TAGGTACCCCACTATGCCTAGCCGTAAAATATTTATTTACACCT-TTCACCGCCCGGGAA

TTACGAGCCCAAGCTTAAAACCCAAAGGACTTGACGGTGCCCCACCCACCTAGAGGAGCC

TGTTCTATAA-TCGATTCTCCCC-GATATACCTCACCACTTCTAGCCTA--TCAGCCTGT

ATACCTCCGTCGTAAGCTTACCATATGAACGCT-TTTTAGTGAGCTAAAAGAT--TTTAC

ATAAACACGTCAGGTCAAGGTGCAGCCCACGAAGTGGTAAGTAATGGGCTACAATTTCTA

AAA---TAGAACATAACGAAAGACTATATGCAA-CTTAGTCATGAAGGCGGATTTAGTAG

TAAAAAGAAAATAGAGTGTTCTTTTTAATTAGGCGCTGGGACGCGTACACACCGCCCGTC

ACCCTCTTCAAACGCTTTT--CCAAG-TTTATAACAAT-ATTATGCACTACAGAAGAGGT

AAGTCGTAACACGGTAAGCGTACTGGAAAGTGCGCTTGGATAAA-CAAAATGTAGCTTAA

CTAAA-GCATCTCGCTTACACCGAGAATATATCTGTGATACCCAGATCATTTTGA--GCT

AAAAACTTAGCTTTA--CTTCTCTCTAATGCTGACCC-CAACCTT--AAACAA--TAAAA

TAAAACATTTTACAC-ATTAAGTAAAGGCGATTAAAAAATGTCTT-AAAGCTATAAAA-C

AAGTACCGCAAGGGAACAGTGAAATAATAATGAAAAA---TTAAAGCACATAAAAGCAGA

GCCTTAACCTCGTACCTTTTGCATCATGGTCTAACTAGTCCCCTCGAGCATAATGAATTT

TAAGTTTGACCCCCCGAAACTAGGTGAGCTACTTAAAAACAGCCTTAT-GGGCCAACCCG

TCTCTGTTGCAAAAGAGTGGGAAGATTTTTTTAGTAGAGGTGATAAACCTACCGAACCTA

GAGATAGCTGGTTATTCAGGAAAAGGATTTAAGTCCTACCTTAGGTTTTTT-GTACA-TC

ACAAGTACCCCAA--AACCT--

---AGCAG

CCACCTTC-AAAAAAGCGTTAAAGCTTCATTGG--TTTTTTATAAAAA-TCCCTTTA-AT

TTTTTATAACCCCT-AATCT-GTACTGAATGGTCTCATGACTTCATGAGAGCCTATATGT

TAGAACTAGTAACAAGAAGA-AGCCCTTCTCCA-AAATGTAAGCATGAGCCAAAACGAAC

AATTCATCGGCATTTAAAGTTAATAATCTCCTAGTAGTAACTTT----TCAAGAAAAACC

TAC------------------TATATTT-AACGTCAACCTTACACCAGCTCATTTATGGA

AAGATTAAAAGACTAGGAAGGAACTCGGCAAATAA-TTAACCCCGCCTGTTTACCAAAAA

CATCGCCTCCTGATTTTC----ATAGGAGGTCTAGCCTGCCCAGTGACAAA--GTTAAAC

GGCCGCGGTACCCTAACCGTGCAAAGGTAGCGCAATCACTTGTTCTTTAAATGAGGACTA

GTATGAACGGCATCACGAGGGTTATGCTGTCTCCCTACTTTATTCAGTGAAACTGATCTC

CCCGTGAAGAAGCGGGGATAAAAATATAAGACGAGAAGACCCCATGGAGCTTTAAACTCA

GTATCAACTGCCATTAT--TAAAACCTAATACCCAA-GCAGTATGTGAC-TACTAGTTTT

CGGTTGGGGTGACCACGGAGTAAAACCTAACCTCCATGATGAAAGGAAATGATAACCTAA

ATTAAGAGCAACAACTCTAAATATCAAA--------AAATTGACTAAA-TTGATCCAATT

AATT--GATCAACGAACCAAGTTACCCTGGGGATAACAGCGCAATCCATTTCAAGAGCTC

TTATCGACAAATGGGTTTACG-ACC-TCGATGTTGGATCAGGGTATCCCAGTG-GCGCAG

CCGCTA-CTAA-CGGTTC

>M. heymonsi MN534679, MN534471, MN534572

TTATTATCAACTTTTTCTTGATTTACACATGCAAGTATCAACACACCCGTGAGAACGCCC

TTTACCCTT-TATAGGACAAGGAGCTGGTATCAGGCTCAG---TCT------TCTAGCCC

ATAACACCTAGCTTTGCCACACCCTCAAGGGTATTCAGCAGTGATTAACATTGTTCATAA

GCGCCAGCTTGACTCAGTCAAAGAACCT--AGAGCCGGCCAATACGGTGCCAGCCGCCGC

GGCTACACCGCTAGGCTCAAGTTGATATT-AATCGGCGTTAAGCGTGTTTAAAGTATGT-

AAAA-GATTAGAATTAAACTTAAACCAAGCTGTGACACGCTTGTTCTTAAGAAAAACAAA

AACGAAAGTTATTCTAACTAAACCACTTGAACCCACGATAGCTAGGACACAAACTGGGAT

TAGGTACCCCACTATGCCTAGCCGTAAAATATTTATTTACACCT-TAAACCGCCAGGGAA

TTACGAGCCCAAGCTTAAAACCCAAAGGACTTGACGGTGCCCCACCCACCTAGAGGAGCC

TGTTCTATAA-TCGATTCTCCCC-GATATACCTCACCACTTCTAGCCTA--TCAGCCTGT

ATACCTCCGTCGTAAGCTTACCATATGAACGCT-TTTTAGTGAGCTAAAAGAT--TTTAC

ATAAATACGTCAGGTCAAGGTGCAGCCCACGAAGTGGTAAGTAATGGGCTACAATTTCTA

GAA---TAGAACATAACGAAAGACTATATGCAA-CTTAGTCATGAAGGCGGATTTAGTAG

TAAAAAGAAAATAGAGCGTTCTTTTTAATCAGGCGCTGGGACGCGTACACACCGCCCGTC

ACCCTCTTCAAACGCTTTA--TCAAG-TTTATAACAAC-ATTATGCACTACAGAAGAGGT

AAGTCGTAACACGGTAAGCGTACTGGAAAGTGCGCTTGGATAAA-CAAAATGTAGCTTAA

TCAAA-GCATCTCGCTTACACCGAGAACATATCTGTGATACCCGGATCATTTTGA--GCT

GATAACTTAGCTTTA--CTTCTCTCAAATGTCCACTC-TTACCTA--AAAAAA--CAAAA

TAAAACATTTTACAC-ATTAAGTAAAGGCGATTAAAAAATGTCTT-AAAGCTATAAAA-C

AAGTACCGCAAGGGAACAGTGAAATAATAATGAAAA----CTAAAGCACATAAAAGCAGA

GCCTTAACCTCGTACCTTTTGCATCATGGTCTAACTAGTCTCCTCGAGCAAAATGAGTTT

TAAGTTTGATCCCCCGAAACTAGGTGAGCTACTTAAAAACAGCCTTCT-GGGCCAACCCG

TCTCTGTTGCAAAAGAGTGGGAAGATTTTTTAAGTAGAGGTGATAAACCTACCGAACCTA

GAGATAGCTGGTTATTCAGGAAAAGGATTTAAGTCCTACCTTAGGTTTTTT-GTACA-AT

ACAAGTACCCC---

---------------------------------------TTGTAAAA--TTCCACTA-AT

TTTCTAAAACCCTT-AACCT-GTACTGAATGGTCTTATGACTTCATGAGAGCCTATATGT

TAGAACTAGTAACAAGAAGA-AGCCCTTCTCCA-AAATGTAAGCATGAGCCAAAATGAAC

TATTCATTGGCATTTAAAGTTAATAATTCCCTAGTAGTAACTTT---TTCAAGAAAAACC

TAC------------------TATATTC-AACGTCAACCTTACACCAGCACATTTCTGGA

AAGATTAAAAGACTAGGAAGGAACTCGGCAAACAA-TTAACCCCGCCTGTTTACCAAAAA

CATCGCCTCCTGATTATT----ATAGGAGGTCTAGCCTGCCCAGTGACAAA--GTTAAAC

GGCCGCGGTACCCTAACCGTGCAAAGGTAGCGCAATCACTTGTTCTTTAAATGAGGACTA

GTATGAACGGCATCACGAGGGTTATGCTGTCTCCCTACTTTATTCAGTGAAACTAATCTC

CCCGTGAAGAAGCGGGGATAAAAATATAAGACGAGAAGACCCCATGGAGCTTTAAACTCA

GTATCAACTGCCATAAT--TAAAACTTAATATTCAA-GCAATTTCTGAT-TACTAGTTTT

CGGTTGGGGTGACCACGGAGTAAAACCTAACCTCCATGATGAAAGGAAATAATAACCTAA

ATTAAGAGCTACAGCTCTAAATATCAAT--------AAATTGACTAA--TTGACCCAATT

ATTT--GATCAACGAACCAAGTTACCCTGGGGATAACAGCGCAATCCATTTCAAGAGCTC

TTATCGACAAATGGGTTTACG-ACC-TCGATGTTGGATCAGGGTATCCCAGTG-GCGCAG

C-----------------

>M. heymonsi MN534680, MN534472, MN534573

TTATTATCAACTTTTTCTTGATTTACACATGCAAGTATCAGCACACCCGTGAGAACGCCC

TTCAACCTCATATAGGACAAGGAGCTGGTATCAGGCTCAG--ATCT------TCTAGCCC

ATAACACCTAGCTTTGCCACACCCTCAAGGGTATTCAGCAGTGATTAACATTGTATATAA

GCGCCAGCTTGACCCAGTCAAAGAACCT--AGAGCCGGCTAATACGGTGCCAGCCGCCGC

GGCTACACCGCTAGGCTCAAGTTGATATT-AATCGGCGTTAAGCGTGTTTAAAGTGCAC-

AAAGAGATTAGAATTAAACTCAAACCAAGCTGTGACACGCTTGTTTTTAAGAAAAACAAA

AACGAAAGTTATTCTAACCAAACCACTTGAATCCACGACAGCTAGGACACAAACTGGGAT

TAGGTACCCCACTATGCCTAGCCGTAAAATATTTATTTACACCT-TTAACCGCCCGGGAA

TTACGAGCCCAAGCTTAAAACCCAAAGGACTTGACGGTGCCCCACCCACCTAGAGGAGCC

TGTTCTATAA-TCGATTCTCCCC-GATATACCTCACCACTTCTAGCCTA--TCAGCCTGT

ATACCTCCGTCGTAAGCTTACCATATGAACGCT-TTTTAGTGAGCTAAAAGAT--TTTAC

ATAAATACGTCAGGTCAAGGTGCAGCCCACGAAGTGGAAAGAAATGGGCTACAATTTCTA

AAA---TAGAACACAACGAAAGACTACATGCAA-CTTAGTCATGAAGGCGGATTTAGTAG

TAAAAAGAAAATAGAGCGTTCTTTTTAATTAGGCGCTGGGACGCGTACACACCGCCCGTC

ACCCTCTTCAAACGCTTTA--TCAAG-TTTATAACAAC-ACTACGCATTACAGAAGAGGT

AAGTCGTAACACGGTAAGCGTACTGGAAAGTGCGCTTGGACAAA-CAAAATGTAGCTTAA

TCAAA-GCATCTCGCTTACACCGAGAACATATCTGTGATACCCGGATCATTTTGA--GCT

AAAAACTTAGCTTTA--CTTCTCTCCAATGTTCACCC-GTACCTC--AAACAA--CAAAA

TAAAACATTTTACAT-ATAAAGTAAAGGCGATTAAAAAATATCTT-AAAGCTATAAAA-C

AAGTACCGCAAGGGAATAGTGAAATAATAATGAAAAA---CTAAAGCACATAAAAGCAGA

GACTCAACCTCGTACCTTTTGCATCATGGTCTAACTAGTCCCTTCGAGCAAAATGAGTTT

TAAGTTTGATCCCCCGAAACTAGGTGAGCTACTTAAAAACAGCCTTCT-GGGCCAACCCG

TCTCTGTTGCAAAAGAGTGGGAAGATTTTTTAAGTAGAGGTGATAAACCTACCGAACCTA

GAGATAGCTGGTTATTCAGGAAAAGGATTTAAGTCCTACCTTAGGTTTTTT-GTATA--T

ACAAGTACCACATC-AA---

---AGCAG

CCACCTTT-AAGAAAGCGTTAAAGCTTCATTA---TATCTTATAAAAA-TTCCACTA-AT

TTTCTAAAACCCTT-AACCT-GTACTGAATGGTCTCATGACTTCATGAGAGTCTATATGT

TAGAACTAGTAACAAGAAGA-AGCCCTTCTCCA-AAATGTAAGCATGAGCCAAAATGAAC

TATTCATTGGCATTTAAAGTTAATAATCTTCTAGTAGTAACTAT----TCAAGAAAAATC

TAC------------------TATACTT-AACGTCAACCTTACACCAGCACATTTCTGGA

AAGATTAAAAGACTAGGAAGGAACTCGGCAAACAA-TTAACCCCGCCTGTTTACCAAAAA

CATCGCCTCCTGATTTCT----ATAGGAGGTCTAGCCTGCCCAGTGACAAA--GTTAAAC

GGCCGCGGTACCCTAACCGTGCAAAGGTAGCGCAATCACTTGTTCTTTAAATGAGGACTA

GTATGAACGGCATCACGAGGGTTATGCTGTCTCCCTACTTTATTCAGTGAAACTGATCTC

CCCGTGAAGAAGCGGGAATAAAAATATAAGACGAGAAGACCCCATGGAGCTTTAAACTCA

GTATCAACTGCTATTAT--TAAAACCTAATACACAA-GCAATCTCTGAC-TACTAGTTTT

CGGTTGGGGTGACCACGGAGTAAAACCTAACCTCCACGATGAAAGGAAATAACAACCTAA

ATTAAGAGCTACAACTCTAAATATCAAT--------AAATTGACTGA--TTGATCCAATT

AATT--GATCAACGAACCAAGTTACCCTGGGGATAACAGCGCAATCCATTTCAAGAGCTC

CTATCGACAAATGGGTTTACG-ACC-TCGATGTTGGATCAGGGTATCCCAGTG-GCGCAG

CTGCTA-CTAA-TGGTTC

>M. heymonsi MN534681, MN534473, MN534574

TTATTATCAACTTTTTCTTGATTTACACATGCAAGTATCAGCACACCCGTGAGAACGCCC

TTCACCCTT-CATAGGACAAGGAGCTGGTATCAGGCTCAG---TCT------TCTAGCCC

ATAACACCTAGCTTTACCACACCCTCAAGGGTATTCAGCAGTGATTAACATTGTATATAA

GCGCCAGCTTGACTCAGTTAAAGAACCC--AGAGCCGGCTAATACGGTGCCAGCCGCCGC

GGCTACACCGCTAGGCTCAAGTTGATATT-AATCGGCGTTAAGCGTGTTTAAAGTGCAC-

AAAA--ATTAGAATTAAACTTAAACCGAGCTGTGACACGCTTGTTTTTAAGAAAAACAAA

AACGAAAGTTATTCTAACCAAACCACTTGAACCCACGACAGCTAGGACACAAACTGGGAT

TAGGTACCCCACTATGCCTAGCCGTAAAATATTTATTTACACCT-TTAACCGCCCGGGAA

TTACGAGCCCAAGCTTAAAACCCAAAGGACTTGACGGTGCCCCACCCACCTAGAGGAGCC

TGTTCTATAA-TCGATTCCCCCC-GATATACCTCACCACTTCTAGCCTA--TCAGCCTGT

ATACCTCCGTCGTAAGCTTACCATATGAACGCT-TTTTAGTGAGCTAAAAGAT-TTTTAC

ATAAATACGTCAGGTCAAGGTGCAGCCCACGAAGTGGTAAGTAATGGGCTACAATTTCTA

AAA---TAGAACATA-CGAAAGACTACATGCAA-TTTAGTCATGAAGGCGGATTTAGTAG

TAAAAAGAAAATAGAGTGTTCTTTTTAATTAGGCGCTGGGACGCGTACACACCGCCCGTC

ACCCTCTTCAAACGCTTTA--TCAAG-TTTATAACAAT-ATTATGCATAACAGAAGAGGT

AAGTCGTAACATGGTAAGCGTACTGGAAAGTGCGCTTGGATAAA-CAAAATATAGCTTAA

TCAAA-GCATCTCGCTTACACCGAGAATATATCTGTGATACCCAGATTATTTTGA--GCC

AAAAACTTAGCTTTA--ACTCTCCCCAATATTCACCC-GCACCAA--AAACAA--CAAAA

TAAAACATTTTACAC-ATTAAGTAAAGGCGATTAAAAAATGTCTT-AAAGCTATAAAA-C

AAGTACCGCAAGGGAATAGTGAAATAATAATGAAAAA---CTAAAGCACATAAAAGCAGA

GCCTTAACCTCGTACCTTTTGCATCATGGTCTAACTAGTCCCCTCGAGCAAAATGAGTTT

TAAGTTTGATCCCCCGAAACTAGGTGAGCTACTTAAAAACAGCCTTCT-GGGCCAACCCG

TCTCTGTTGCAAAAGAGTGGGAAGATTTT-TAAGTAGAGGTGATAAACCTACCGAACCTA

GAGATAGCTGGTTATTCAGGAAAAGGATTTAAGTCCTACCTTAGGTTTTTT-GTACA-AT

ATAAGTACCCCCACCAACCCTTA-------------------------------------

---AGCAG

CCACCTTT-AAAAAAGCGTTTAAGCTTCATTG---TATTTTATAAAAATTTCCACTA-AT

TTTCTAAAACCCTTTAACCT-GTACTGAATGGTCTTATGACTTCATAAGAGCCTATATGT

TAGAACTAGTAACAAGAAGA-AGCCCTTCTCCA-AAATGTAAGCATGAACCAAAATGAAC

TATTCATTGGCATTTAAAGTTAATAATCTTCTAGTAGTAACTAC----TCAAGAAAAACC

TAC------------------TATATTT-AACGTCAACCTTACACCAGCACATTCCTGGA

AAGATTAAAAGACTAGGAAGGAACTCGGCAAACAA-ATAACCCCGCCTGTTTACCAAAAA

CATCGCCTCCTGATTTTT----ATAGGAGGTCTAGCCTGCCCAGTGACAAA--GTTAAAC

GGCCGCGGTACCCTAACCGTGCAAAGGTAGCGCAATCACTTGTTCTTTAAATGAGGACTA

GTATGAACGGCATCACGAGGGTTATGCTGTCTCCCTACTTTATTCAGTGAAACTGATCTC

CCCGTGAAGAGGCGGGGATAAAAATATAAGACGAGAAGACCCCATGGAGCTTTAAACTCA

GTGTCAACTGCCATATT--TAAAACCTAATATTCAA-GCAAGTTATGAC-TACTAGTTTT

CGGTTGGGGTGACCACGGAGTAAAACCTAACCTCCACGATGAAAGGAAATAGCAACCTAA

ATTAAGAGCTACAGCTCTAAATATCAAT--------AAATTGACTTA--TTGACCCAATT

AATT--GATCAACGAACCAAGTTACCCTGGGGATAACAGCGCAATCCATTTCAAGAGCTC

CTATCGACAAATGGGTTTACG-ACC-TCGATGTTGGATCAGGGTATCCCAGTG-GCGCAG

CCGCTA-CTAA-TGGTTC

>M. heymonsi MN534682, MN534474, MN534575

TTATAATCAACTTCTTCTTGATTTACACATGCAAGTATCAACACACCCGTGAGAACGCCC

TTTACCCTT-TATAGGACAAGGAGCTGGTATCAGGCTCAG---TCT------TCTAGCCC

```
ATAACACCTAGCTTTGCCACACCCTCAAGGGTATTCAGCAGTGATTAACATTGTTCATAA
GCGCCAGCTTGACTCAGTCAAAGAACCT--AGAGCCGGCCAATACGGTGCCAGCCGCCGC
GGCTACACCGCTAGGCTCAAGTTGATATT-AATCGGCGTTAAGCGTGTTTAAAGTATGT-
AAAA-GATTAGAATTAAACTTAAACCAAGCTGTGACACGCTTGTTCTTAAGAAAAACAAA
AACGAAAGTTATTCTAACTAAACCACTTGAACCCACGATAGCTAGGACACAAACTGGGAT
TAGGTACCCCACTATGCCTAGCCGTAAAATATTTATTTACACCT-TAAACCGCCAGGGAA
TTACGAGCCCAAGCTTAAAACCCAAAGGACTTGACGGTGCCCCACCCACCTAGAGGAGCC
TGTTCTATAA-TCGATTCTCCCC-GATATACCTCACCACTTCTAGCCTA--TCAGCCTGT
ATACCTCCGTCGTAAGCTTACCATATGAACGCT-TTTTAGTGAGCTAAAAGAT--TTTAC
ATAAATACGTCAGGTCAAGGTGCAGCCCACGAAGTGGTAAGTAATGGGCTACAATTTCTA
GAA---TAGAACATAACGAAAGACTATATGCAA-CTTAGTCATGAAGGCGGATTTAGTAG
TAAAAAGAAAATAGAGCGTTCTTTTTAATCAGGCGCTGGGACGCGTACACACCGCCCGTC
ACCCTCTTCAAACGCTTTA--TCAAG-TTTATAACAAC-ATTATGCACTACAGAAGAGGT
AAGTCGTAACACGGTAAGCGTACTGGAAAGTGCGCTTGGATAAA-CAAAATGTAGCTTAA
TCAAA-GCATCTCGCTTACACCGAGAACATATCTGTGATACCCGGATCATTTTGA--GCT
GATAACTTAGCTTTA--CTTCTCTCAAATGTCCACTC-TTACCTA--AGAAAA--CAAAA
TAAAACATTTTACAC-ATTAAGTAAAGGCGATTAAAAAATGTCTT-AAAGCTATAAAA-C
AAGTACCGCAAGGGAACAGTGAAATAATAATGAAAA----CTAAAGCACATAAAAGCAGA
GCCTTAACCTCGTACCTTTTGCATCATGGTCTAACTAGTCTCCTCGAGCAAAATGAGTTT
TAAGTTTGATCCCCCGAAACTAGGTGAGCTACTTAAAAACAGCCTTCT-GGGCCAACCCG
TCTCTGTTGCAAAAGAGTGGGAAGATTTTTTAAGTAGAGGTGATAAACCTACCGAACCTA
GAGATAGCTGGTTATTCAGGAAAAGGATT-------------------------------
------------------------------------------------------------
-------------------------------------------------------AGCAG
CCACCTTT-AAAAAAGCGTTAAAGCTTCATTA---TATTTTGTAAAAA-TTCCACTA-AT
TTTCTAAAACCCTT-AACCT-GTACTGAATGGTCTCATGACTTCATGAGAGCCTATATGT
TAGAACTAGTAACAAGAAGA-AGCCCTTCTCCA-AAATGTAAGCATGAGCCAAAATGAAC
TATTCATTGGCATTTAAAGTTAATAATTCCCTAGTAGTAACTTT---TTCAAGAAAAACC
TAC------------------TATATTC-AACGTCAACCTTACACCAGCACATTTCTGGA
AAGATTAAAAGACTAGGAAGGAACTCGGCAAACAA-TTAACCCCGCCTGTTTACCAAAAA
```

CATCGCCTCCTGATTATT----ATAGGAGGTCTAGCCTGCCCAGTGACAAA--GTTAAAC

GGCCGCGGTACCCTAACCGTGCAAAGGTAGCGCAATCACTTGTTCTTTAAATGAGGACTA

GTATGAACGGCATCACGAGGGTTATGCTGTCTCCCTACTTTATTCAGTGAAACTAATCTC

CCCGTGAAGAAGCGGGGATAAAAATATAAGACGAGAAGACCCCATGGAGCTTTAAACTCA

GTATCAACTGCCATAAT--TAAAACTTAATATTCAA-GCAATTTCTGAT-AACTAGTTTT

CGGTTGGGGTGACCACGGAGTAAAACCTAACCTCCATGATGAAAGGAAATAATAACCTAA

ATTAAGAGCTACAGCTCTAAATATCAAT--------AAATTGACTAA--TTGACCCAATT

ATTT--GATCAACGAACCAAGTTACCCTGGGGATAACAGCGCAATCCATTTCAAGAGCTC

TTATCGACAAATGGGTTTACG-ACC-TCGATGTTGGATCAGGGTATCCCAGTG-GCGCAG

CCGCTA-CTAA-CGGTTC

>M. heymonsi MN534683, MN534475, MN534576

TTATTATCAACTTTTTCTTGATTTACACATGCAAGTATCAGCACACCCGTGAGAACGCCC

TTTACCCTT-TATAGGATAAGGAGCTGGTATCAGGCTCAG---TCT------TCTAGCCC

ATAACACCTAGCTTTGCCACACCCTCAAGGGTATTCAGCAGTGATTAACATTGTATATAA

GCGCCAGCTTGACTCAGTCAAAGAACCT--AGAGCCGGCTAATACGGTGCCAGCCGCCGC

GGCTACACCGCTAGGCTCAAGTTGACATT-AATCGGCGTTAAGCGTGTTTAAAGTGCAC-

TAAAA-ATTAGAATTAAACTTAAACCAAGCTGTGACACGCTTGTTTTTAAGAAAAACAAA

AACGAAAGTTATTCTAACCAAACCACTTGAACCCACGACAGCTAGGGCACAAACTGGGAT

TAGGTACCCCACTATGCCTAGCCGTAAAATATTTATTTACACCT-TAAACCGCCTGGGAA

TTACGAGCCCAAGCTTAAAACCCAAAGGACTTGACGGTGCCCCACCCACCTAGAGGAGCC

TGTTCTATAA-TCGATTCTCCCC-GATATACCTCACCACTTCTAGCCTA--TCAGCCTGT

ATACCTCCGTCGTAAGCTTACCATATGAACGCT-TTTTAGTGAGCTAAAAGAT--TTTAC

ATAAATACGTCAGGTCAAGGTGCAGCCCACGAAGTGGTAAGTAATGGGCTACAATTTCTA

AAA---TAGAACACAACGAAAGACTATATGCAA-CTTAGTCATGAAGGCGGATTTAGTAG

TAAAAAGAAAATAGAGCGTTCTTTTTAATTAGGCACTGGGACGCGTACACACCGCCCGTC

ACCCTCTTCAAACGCTTTA--TCAAG-TTTATAACAAT-TTTATGCATCACAGAAGAGGT

AAGTCGTAACACGGTAAGCGTACTGGAAAGTGTGCTTGGATAAA-CAAAATGTAGCTTAA

TCAAA-GCATCTCGCTTACACCGAGAATATATCTGTGATACCCGGATCATTTTGA--GCT

AAAAACTCAGCTTTA--CTTCTCCCCAATGTTCACCC-GTTCCTT--AAACAA--CAAAA

TAAAACATTTTATAC-ATTAAGTAAAGGCGATTAAAAAATGTCTT-AAAGCTATAAAA-C

AAGTACCGCAAGGGAATAGTGAAATAATAATGAAAA----CTAAAGCACGTAAAAGCAGA
GCTTTAACCTCGTACCTTTTGCATCATGGTCTAACTAGTCCCCTCGAGCAAAATGAATTT
TAAGTTTGATCCCCCGAAACTAGGTGAGCTACTTAAAAACAGCCTTCT-GGGCCAACCCG
TCTCTGTTGCAAAAGAGTGGGAAGATTTTTTAAGTAGAGGTGACAAACCTACCGAACCTA
GAGATAGCTGGTTATTC---

---AGCAG
CCACCTTTT-AAAAAGCGTTAAAGCTTCATTGC---GTAATATAAAAATTTCCACTA-AT
TTTCTAAAACCCCCTAACCT-GTACTGAATGGTCTTATGACTTCATAAGAGCCTATATGT
TAGAACTAGTAACAAGAAGA-AGCCCTTCTCCA-AAATGTAAGCATGAGCCAAAATGAAC
AATTCATTGGCATTTAAAGTTAATAATCTTCTAGTAGTAACTAT----TCAAGAAAAACC
TAC------------------TATATCT-AACGTCAACCTTACACCAGCACATTTCTGGA
AAGATTAAAAGATTAGGAAGGAACTCGGCAAACAA-ATAACCCCGCCTGTTTACCAAAAA
CATCGCCTCCTGATTTTT----ATAGGAGGTCTAGCCTGCCCAGTGACAAA--GTTAAAC
GGCCGCGGTACCCTAACCGTGCAAAGGTAGCGCAATCACTTGTTCTTTAAATGAGGACTA
GTATGAACGGCATCACGAGGGTTATGCTGTCTCCCTACTTTATTCAGTGAAACTGATCTC
CCCGTGAAGAGGCGGGGATAAAAATATAAGACGAGAAGACCCCATGGAGCTTTAAACTCA
GTATCAACTGCCATATT--TAAAACCTAATATTCAA-GCAATTTCTGAC-TACTAGTTTT
TGGTTGGGGTGACCACGGAGTAAAACCTAACCTCCATGATGAAAGGAAATAACAACCTAA
ATCAAGAGCTACAGCTCTAAATATCAAT--------AAATTGACTGA--TTGATCCAATT
AATT--GATCAACGAACCAAGTTACCCTGGGGATAACAGCGCAATCCATTTCAAGAGCTC
CTATCGACAAATGGGTTTACG-ACC-TCGATGTTGGATCAGGGTATCCCAGTG-GCGCAG
CCGCTA-CTAA-CGGTTC
>M. heymonsi MN534684, MN534476, MN534577
TTATTATCAGCTTTTTCTTAACTTACACATGCAAGTATCAGCAAACCCGTGAGAACGCCC
TTTACCCTT-TATAGGACAAGGAGCTGGTATCAGGCTCAG---TCT------TCTAGCCC
ATAACACCTAGCTTTGCCACACCCTCAAGGGTACTCAGCAGTGATTAACATTGTTTATAA
GCGCCAGCTTAAATCAGTCAAAGAATCT--AGAGCCGGCTAATACGGTGCCAGCCGCCGC
GGCTACACCGCTAGGCTCAAGTTGATATT-AATCGGCGTTAAGCGTGTTTAAAGTGCAC-
AAAA-GATTAGAATTAAACTTAAACCAAGCTGTGACACGCTTGTTCTTAAGAAAAACAAA

AACGAAAGTTATTCTAACCAAACCACTTGAACCCACGACAGCTAAGACACAAACTGGGAT

TAGGTACCCCACTATGCCTAGCCGTAAAATATTTATTTACACCT-TTAACCGCCCGGGAA

TTACGAGCCCAAGCTTAAAACCCAAAGGACTTGACGGTGCCCCACCCACCTAGAGGAGCC

TGTTCTATAA-TCGATTCTCCCC-GATATACCTCACCACTTCTAGCCTA--TCAGCCTGT

ATACCTCAGTCGTAAGCTTACCATATGAACGCT-TTTTAGTGAGCTAAAAGAT--TTTAC

ATAAACACGTCAGGTCAAGGTGCAGCCCACGAAGTGGTAAGTAATGGGCTACAATTTCTA

AAA---TAGAACACAACGAAAGACTACATGCAA-CTTAGTCATGAAGGCGGATTTAGAAG

TAAAAAGAAAATAGAGCGTTCTTTTTAATCAGGCGCTGGGACGCGTACACACCGCCCGTC

ACCCTCTTCAAACGCTTCA--TCAAG-TTTATAACAAT-ATTTTGCACTACAGAAGAGGT

AAGTCGTAACATGGTAAGCGTACTGGAAAGTGCGCTTGGATAAA-CAAAATGTAGCTTAA

CCAAA-GCATCTCGCTTACACCGAGAATATATCTGTGACACCCAGATCATTTTGA--GCT

AAAAACTTAGCTTTA--CTTCCCCCCAATGTTTACCCTAATTCTT---AACAA--TAAAA

TAAAACATTTTATAC-ATTAAGTAAAGGCGATTAAAAAATGTCTT-AAAGCTATAAAA-C

AAGTACCGCAAGGGAACAGTGAAATAACAATGAAAAA---TTAAAGCATATAAAAGTAGA

GCTTCAACCTCGTACCTTTTGCATCATGGTCTAACTAGTCCCCTCAAGCAGAATGAATTT

TAAGTTTGACCCCCCGAAACTAAGTGAGCTACTTAAAAACAGCCTTCT-GGGCCAACCCG

TCTCTGTTGCAAAAGAGTGGGAAGATTTT-TAAGTAGAGGTGACAAACCTACCGAACCTA

GAGATAGCTGGTTATTCAGGAAAAGGATTTAAGTCCTACCTTAGGTTTTT--GTACA-AC

ACAAGTAACCCAA--AACCTTAAGAGCTATTCAAATAAGGTACAGCCTATTTGAAACAGG

ATACAACCTCAC---AGCAG

CCACCTTT--AAAAAGCGTTTAAGCTTCATTT---GTTATTATAAAAA--TCCTTAAATT

TTTCTACAACCCTT-AATCT-GTACTGAATGATCTCATGACTTCATGAGAGCCTATATGT

TAGAACTAGTAACAAGAAGAAAGCACTTCTCCA-AAATGTAAGCATGAGCCAAAATGAAC

TATTCATTGGCATTTAAAGTTAATAAATCCCTAGTAGTTAACTA---TTCAAGAAAAACC

TACT-----------------TATATAT-GACGTCAACCTTACACCAGCCCATTTCTGGA

AAGATTAAAAAACTAGGAAGGAACTCGGCAAATAA-CTAACCCCGCCTGTTTACCAAAAA

CATCGCCTCCTGATTTCT----ATAAGAGGTCTAGCCTGCCCAGTGACAAA--GTTAAAC

GGCCGCGGTACCCTAACCGTGCAAAGGTAGCGCAATCACTTGTTCTTTAAATGAGGACTA

GTATGAACGGCATCACGAGGGTTATGCTGTCTCCCTACTTTATTCAGTGAAACTAATCTC

CCCGTGAAGAAGCGGGGATAAAAATATAAGACGAGAAGACCCCATGGAGCTTTAAACTCA

GTATCAACTGCCATTAT--TAAAACCTTATACCCAA-GCAGTCTATGAC-TACTAGTTTT

CGGTTGGGGTGACCACGGAGTAAAATCTAACCTCCATGATGAAAGGAACTAAAAACCTAA

ATTAAGGGCTACAACCCTACATATCATA--------AAATTGACTAA--TTGATCCAATT

AATT--GATCAACGAACCAAGTTACCCTGGGGATAACAGCGCAATCCATTTCAAGAGCTC

TTATCGACAAATGGGTTTACG-ACC-TCGATGTTGGATCAGGGTATCCCAGTG-GCGCAG

CCGCTA-CTAA-------

>M. heymonsi MN534685, MN534477, MN534578

TTATTATCAGCTTTTTCTTAACTTACACATGCAAGTATCAGCAAACCCGTGAGAACGCCC

TTTACCCTT-TATAGGACAAGGAGCTGGTATCAGGCTCAG---TCT------TCTAGCCC

ATAACACCTAGCTTTGCCACACCCTCAAGGGTACTCAGCAGTGATTAACATTGTTTATAA

GCGCCAGCTTAAATCAGTCAAAGAATCT--AGAGCCGGCTAATACGGTGCCAGCCGCCGC

GGCTACACCGCTAGGCTCAAGTTGATATT-AATCGGCGTTAAGCGTGTTTAAAGTGCAC-

AAAA-GATTAGAATTAAACTTAAACCAAGCTGTGACACGCTTGTTCTTAAGAAAAACAAA

AACGAAAGTTATTCTAACCAAACCACTTGAACCCACGACAGCTAAGACACAAACTGGGAT

TAGGTACCCCACTATGCCTAGCCGTAAAATATTTATTTACACCT-TTAACCGCCCGGGAA

TTACGAGCCCAAGCTTAAAACCCAAAGGACTTGACGGTGCCCCACCCACCTAGAGGAGCC

TGTTCTATAA-TCGATTCCCCCC-GATATACCTCACCACTTCTAGCCTA--TCAGCCTGT

ATACCTCCGTCGTAAGCTTACCATATGAACGCT-TTTTAGTGAGCTAAAAGAT--TTTAC

ATAAACACGTCAGGTCAAGGTGCAGCCCACGAAGTGGTAAGTAATGGGCTACAATTTCTA

AAA---TAGAACACAACGAAAGACTACATGCAA-CTTAGTCATGAAGGCGGATTTAGAAG

TAAAAAGAAAATAGAGCGTTCTTTTTAATCAGGCGCTGGGACGCGTACACACCGCCCGTC

ACCCTCTTCAAACGCTTCA--TCAAG-TTTATAACAAT-ATTTTGCACTACAGAAGAGGT

AAGTCGTAACATGGTAAGCGTACTGGAAAGTGCGCTTGGATAAA-CAAAATGTAGCTTAA

CCAAA-GCATCTCGCTTACACCGAGAATATATCTGTGACACCCAGATCATTTTGA--GCT

AAAAACTTAGCTTTA--CTTCCCCCCAATGTTTACCCTAATTCTT---AACAA--TAAAA

TAAAACATTTTATAC-ATTAAGTAAAGGCGATTAAAAAATGTCTT-AAAGCTATAAAA-C

AAGTACCGCAAGGGAACAGTGAAATAACAATGAAAAA---TTAAAGCATATAAAAGTAGA

GCTTCAACCTCGTACCTTTTGCATCATGGTCTAACTAGTCCCCTCAAGCAGAATGAATTT

TAAGTTTGACCCCCCGAAACTAAGTGAGCTACTTAAAAACAGCCTTCT-GGGCCAACCCG

TCTCTGTTGCAAAAGAGTGGGAAGATTTT-TAAGTAGAGGTGATAAACCTACCGAACCTA

GAGATAGCTGGTTATTCAGGAAAAGGATTTAAGTCCTACCTTAGGTTTTT--ATACA-AC
ACAAGTAACCCAA--AACCTTAAGAGCTATTCAAATAAGGTACAGCCTATTTGAAACAGG
ATACAACCTCA--AGCAG
CCACCTTT-AAAAAAGCGTTTAAGCTTCATTT---GTTATTATAAAAA--TCCTTAAATT
TTTCTACAACCCTT-AATCT-GTACTGAATGATCTCATGACTTCATGAGAGCCTATATGT
TAGAACTAGTAACAAGAAGAAAGCACTTCTCCA-AAATGTAAGCATGAGCCAAAATGAAC
TATTCATTGGCATTTAAAGTTAATAAATCCCTAGTAGTTAACTA---TTCAAGAAAAACC
TACT-----------------TATATAT-GACGTCAACCTTACACCAGCCCATTTCTGGA
AAGATTAAAAAACTAGGAAGGAACTCGGCAAATAA-CTAACCCCGCCTGTTTACCAAAAA
CATCGCCTCCTGATTTCT----ATAAGAGGTCTAGCCTGCCCAGTGACAAA--GTTAAAC
GGCCGCGGTACCCTAACCGTGCAAAGGTAGCGCAATCACTTGTTCTTTAAATGAGGACTA
GTATGAACGGCATCACGAGGGTTATGCTGTCTCCCTACTTTATTCAGTGAAACTAATCTC
CCCGTGAAGAAGCGGGGATAAAAATATAAGACGAGAAGACCCCATGGAGCTTTAAACTCA
GTATCAACTGCCATTAT--TAAAACCTTATACCCAA-GCAGTCTATGAC-TACTAGTTTT
CGGTTGGGGTGACCACGGAGTAAAATCTAACCTCCATGATGAAAGGAACTAAAAACCTAA
ATTAAGGGCTACAACCCTACATATCATA--------AAATTGACTAA--TTGATCCAATT
AATT--GATCAACGAACCAAGTTACCCTGGGGATAACAGCGCAATCCATTTCAAGAGCTC
TTATCGACAAATGGGTTTACG-ACC-TCGATGTTGGATCAGGGTATCCCAGTG-GCGCAG
CCGCTA-CTAA-CGGTTC
>M. heymonsi MN534686, MN534478, MN534579
TTACTATCAACTTTTTCTTGACTTACACATGCAAGTATCAGCAAACCCGTGAGAACGCCC
TTTACCCTT-TATAGGACAAGGAGCTGGTATCAGGCTCAG---TCT------TCTAGCCC
ATAACACCTAGCTTTGCCACACCCTCAAGGGTACTCAGCAGTGATTAACATTGTTTATAA
GCGCCAGCTTGAATCAGTTAAAGAACCT--AGAGCCGGCTAATACGGTGCCAGCCGCCGC
GGCTACACCGCTAGGCTCAAGTTGATATT-AATCGGCGTTAAGCGTGTTTAAAGTGAAC-
AAAA-GATTAGAATTGAACTTAAACCAAGCTGTGACACGCTTGTTTTTAAGAAAAACAAA
AACGAAAGTTATTCTAACCAAACCACTTGAATCCACGACAGCTAGGACACAAACTGGGAT
TAGGTACCCCACTATGCCTAGCCGTAAAATATTTACTTACACCT-TTAACCGCCCGGGAA
TTACGAGCCCAAGCTTAAAACCCAAAGGACTTGACGGTGCCCCACCCACCTAGAGGAGCC
TGTTCTATAA-TCGATTCTCCCC-GATATACCTCACCACTTCTAGCCTA--TCAGCCTGT

ATACCTCCGTCGTAAGCTTACCATATGAACGCT-TTTTAGTGAGCCAAAAGAT--TTTAC

ATAAACACGTCAGGTCAAGGTGCAGCCCACGAAGTGGTAAGTAATGGGCTACAATTTCTA

AAA---TAGAACACA-CGAAAGACTATATGCAA-CTTGGTCATGAAGGCGGATTTAGAAG

TAAAAAGAAAATAGAGTGTTCTTTTTAATTAGGCGCTGGGACGCGTACACACCGCCCGTC

ACCCTCTTCAAACGCTTCA--TCAAG-TTTATAACAAT-ATTATGCACTACAGAAGAGGT

AAGTCGTAACACGGTAAGTGTACTGGAAAGTGCACTTGGATAAA-CAAAATGTAGCTTAA

TCAAA-GCATCTCGCTTACACCGAGGATATATCTGTGATACCCGGATCATTTTGA--GCT

AAAAACTTAGCTTTA--CTTCTCTCCAATGTTTACCCTAAT-CTT--AAACAA--TAAAA

TAAAACATTTTACAC-ATTTAGTAAAGGCGATTAAAAAATGTCTT-AAAGCTATAAAA-C

AAGTACCGCAAGGGAACAGTGAAATAATAATGAAAA----CTAAAGCACATAAAAGCAGA

GCCTCAACCTCGTACCTTTTGCATCATGGTCTAACTAGTCCCCTCAAGCAGAATGAACTT

TAAGTTTGACCCCCCGAAACTAAGTGAGCTACTTAAAAACAGCCTTCT-GGGCCAACCCG

TCTCTGTTGCAAAAGAGTGGGAAGATTCT-TAAGTAGAGGTGATAAACCTACCGAACTTA

GAGATAGCTGGTTATTCAGGAAAAGGATTTAAGTCCTACCTTAGGTTTTT--ATACA-AC

ATAAGTACCTCAA--AACCTTAAGAGCTATTCAAATAAGGTACAGCCTATTTGAAACAGG

ATACAACCTCACCA--

--------------------------GAGGTCTAGCCTGCCCAGTGACAAA--GTTAAAC

GGCCGCGGTACCGTAACCGTGCAAAGGTAGCACATTCACTTGTTCTTTAAATGAGGACTG

GTATGAACGGCATCACGAGGGTTATGCTGTCTCCCTACTTTATTCAGTGAAACTAATCTC

CCCGTGAAGAAGCGGGGATAAAAATATAAGACGAGAAGACCCCATGGAGCTTTAAACTCA

GTATCAACTGCCATAAT--TATAGCCTTATATTCAA-GCAGTCTATGAC-TACTAGTTTT

CGGTTGGGGTGACCACGGAGTAAAACCTAACCTCCATGATGAAAGGAACTAAAAACCTAA

ATTAAGGGCTACAACCCTAAATATCATA--------AAATTGACTAA--CTGATCCAATT

AATT--GATCAACGAACCAAGTTACCCTGGGGATAACAGCGCAATCCATTTCAAGAGCTC

TTATCGACAAATGGGTTTACG-ACC-TCGATGTTGGATCAGGGTATCCAAGTG-GCGCAG

CCGCTA-CTAA-TGGTTC

>M. mukhlesuri MN534687, MN534479, MN534580

TTATTATCACCTCCCTCTCGACTTACACATGCAAGTTTCAGCACACCCGTGAGAACGCCC

TTCACCCTT-TTTAGGACAAGGAGCTGGTATCAGGCACAG---ATT------TCTAGCCC

ATGACACCTAGCTTTGCCACACCCTCAAGGGTCTTCAGCAGTGATTAACATTGTTTATAA

GCGCCAGCTTGAATCAGTCAGAGAACCA--AGAGCCGGCCAATACGGTGCCAGCCGCCGC

GGCTACACCGCTAGGCTCAAGTTGATATT-ATTCGGCGTTAAGCGTGTTTAAAGTGCCC-

AAACAGATTAGAATTAAACTTTAACCAAGCCGTGATACGCTTGTTTCTAAGAAAACCACA

ATCGAAAGTTATTCTAACCAAACCACTTGAACCCACGACAGCTAGGGAACAAACTGGGAT

TAGGTACCCCACTATGCCTAGCCGTAAAACATTTACTTACACCC-TTAACCGCCCGGGAA

TTACGAGCCCAAGCTTAAAACCCAAAGGACTTGACGGTGTCCCACCCACCTAGAGGAGCC

TGTTCTATAA-TCGATTCCCCCC-GATCTACCTCACCATTTCTAGCCTT--TCAGCCTGT

ATACCTCCGTCGTAAGCTTACCATATGAACGTT-TCTAAGTGAGCTAAAAGAT-TTCCTC

GTAAATACGTCAGGTCAAGGTGCAGCCCACGAAATGGCAAGCAATGGGCTACAATTTCTA

CTA---TAGAACATAACGAAAGACTACATGCAA-CTTAGTCATGAAGGCGGATTTAGCAG

TAAAAAGAAAGTAGAGAGTTCTTTTTAATTAGGCGCTGGGACGCGTACACACCGCCCGTC

ACCCTCTTCAAACGCTTCA-AATATG-TTTATAACACT-TTTATGCATCACAGAAGAGGT

AAGTCGTAACATGGTAAGTGTACTGGAAAGTGCACTTGGATTAA-CAAAATGTAGCTTAA

CTAAA-GCATCTCGCTTACACCGAGAACATGTCCGTGAAACTCAGATCATTTTGA--GCT

GAAAACCTAGCTTAC--TCCATTCT-TATGAATATAC-CACCTTAA-CACATA--CTAAA

TAAAACATTTTGTAC-ATTAAGTAAAGGCGATTAAAAAATGTCTT-AAAGCTATAGAAAT

TAGTACCGCAAGGGAAAAGTGAAATATTAATGAAAAA--CCTCAAGCACAAAAAAGTAGA

GCTACAACCTCGTACCTTTTGCATCATGGTCTAACTAGTTCAACCAAGCAAAATGAATTT

TAAGTTTGACTTCCCGAAACTAGGTGAGCTACTTAAAAACAGCCTTAC-GGGCCAACCCG

TCTCTGTTGCAAAAGAGTGGGAAGATTTT-TAAGTAGAGGTGACAGACCTACCGAACCTA

GAGATAGCTGGTTATTCAGGAAAAGGATTTAAGTCCTACCTTAAGTTTATT-GTATA-AA

A-TAATACACAACT-AACTT--

------TAC-AAAAAGCGTTAAAGCTTCATTG---TTTTTATCAAAAA-TACCATTA-AT

TAACTGTAACCCTT-CACCG-GTACTGAATGATCTCATAGTGGTATGAGAGCCTATATGT

TAGAACTAGTAACAAGAAGA-AGCCCTTCTCCA-AAATGTAAGCATGAGCCAAAATGAAC

TATTCACTGGCACTTAAAGTTA-TAAACCTATTGTAGCAACTTA----ACAAGAAAAACC

TAC------------------AACTGCA-AACGTCAACCTTACACCAGCACATTTCTGGA

AAGATAAAAAGAATAGGAAGGAACTCGGCAAATAA-CTAACCCCGCCTGTTTACCAAAAA

CATCGCCTCCTGATCACCC---ATAGGAGGTCCAGCCTGCCCAGTGACTAA--GTTAAAC

GGCCGCGGTACCCTAACCGTGCAAAGGTAGCGCAATCACTTGTTCTTTAAATGAGGACTA

GTATGAACGGCATCACGAGGGTTATGCTGTCTCCCTACTCTACTCAGTGAAACTGATCTC

CCCGTGAAGAAGCGGGGATAAAAATATAAGACGAGAAGACCCCATGGAGCTTTAAACTCA

GTACCAACTGCCCAAAT--AATAGCCTATTACCCCT-GCAG-CTATGGT-TACTAGTTTT

CGGTTGGGGTGACCACGGAGTAAAATTTAACCTCCACGATGAAAGGAACTAATATCCTAA

CCTATGAGCTACAGCTCTAAGTATCAAC--------AAATTGACTAA--TTGACCCAATT

ACTT--GATCAACGAACCAAGTTACCCTGGGGATAACAGCGCAATCCATTTCAAGAGCTC

ATATCGACAAATGGGTTTACG-ACC-TCGATGTTGGATCAGGGTATCCCAGTG-GCGCAG

CCGCTA-CTAA-CGGTTC

>M. mukhlesuri MN534688, MN534480, MN534581

TTATTATCACCTCCCTCTCGACTTACACATGCAAGTTTCAGCACACCCGTGAGAACGCCC

TTCACCCTT-TTTAGGACAAGGAGCTGGTATCAGGCACAG---ATT------TCTAGCCC

ATGACACCTAGCTTTGCCACACCCTCAAGGGTCTTCAGCAGTGATTAACATTGTTTATAA

GCGCCAGCTTGAATCAGTCAGAGAACCA--AGAGCCGGCCAATACGGTGCCAGCCGCCGC

GGCTACACCGCTAGGCTCAAGTTGATATT-ATTCGGCGTTAAGCGTGTTTAAAGTGCCC-

AAACAGATTAGAATTAAACTTTAACCAAGCCGTGATACGCTTGTTTCTAAGAAAACCACA

ATCGAAAGTTATTCTAACCAAACCACTTGAACCCACGACAGCTAGGGAACAAACTGGGAT

TAGGTACCCCACTATGCCTAGCCGTAAAACATTTACTTACACCC-TTAACCGCCTGGGAA

TTACGAGCCCAAGCTTAAAACCCAAAGGACTTGACGGTGTCCCACCCACCTAGAGGAGCC

TGTTCTATAA-TCGATTCCCCCC-GATCTACCTCACCATTTCTAGCCTT--TCAGCCTGT

ATACCTCCGTCGTAAGCTTACCATATGAACGTT-TCTAAGTGAGCTAAAAGAT-TTCCTC

ATAAATACGTCAGGTCAAGGTGCAGCCCACGAAATGGCAAGCAATGGGCTACAATTTCTA

CTA---TAGAACATAACGAAAGACTACATGCAA-CTTAGTCATGAAGGCGGATTTAGCAG

TAAAAAGAAAGTAGAGAGTTCTTTTTAATTAGGCGCTGGGACGCGTACACACCGCCCGTC

ACCCTCTTCAAACGCTTCA-AATATG-TTTATAACACT-TTTATGCATCACAGAAGAGGT

AAGTCGTAACATGGTAAGTGTACTGGAAAGTGCACTTGGATTAA-CAAAATGTAGCTTAA

CTAAA-GCATCTCGCTTACACCGAGAACATGTCCGTGAAACTCAGATCATTTTGA--GCT

GAAAACCTAGCTTAC--TCCATTCT-TATGAATATAC-CACCCTAA-CACATA--CTAAA

TAAAACATTTTGTAC-ATTAAGTAAAGGCGATTAAAAAATGTCTT-AAAGCTATAGAAAT

TAGTACCGCAAGGGAAAAGTGAAATATTAATGAAAAA--CCTCAAGCACAAAAAAGTAGA

GCTACAATCTCGTACCTTTTGCATCATGGTCTAACTAGTTCAACCAAGCAAAATGAATTT

TAAGTTTGACTTCCCGAAACTAGGTGAGCTACTTAAAAACAGCCTTAC-GGGCCAACCCG

TCTCTGTTGCAAAAGAGTGGGAAGATTTTTTAAGTAGAGGTGACAGACCTACCGAACCTA

GAGATAGCTGGTTATTCAGGAAAAGGATTTAAGTCCTACCTTAAGTTTATT-GT------

--AG

CCACTTTAC-AAAAAGCGTTAAAGCTTCATTG--TTTTTTATCAAAAA-TACCATTA-AT

TAACTGTAACCCTT-CACCA-GTACTGAATGATCTCATAGTAGTATGAGAGCCTATATGT

TAGAACTAGTAACAAGAAGA-AGCCCTTCTCCA-AAATGTAAGCATGAGCCAAAATGAAC

TATTCACTGGCATTTAAAGTTA-TAAACCTATTGTAGCAACTTA----ACAAGAAAAACC

TAC------------------AACTGCA-AACGTCAACCTTACACCAGCACATTTCTGGA

AAGATAAAAAGAATAGGAAGGAACTCGGCAAATAA-CTAACCCCGCCTGTTTACCAAAAA

CATCGCCTCCTGATCACCC---ATAGGAGGTCCAGCCTGCCCAGTGACTAA--GTTAAAC

GGCCGCGGTACCCTAACCGTGCAAAGGTAGCGCAATCACTTGTTCTTTAAATGAGGACTA

GTATGAACGGCATCACGAGGGTTATGCTGTCTCCCTACTCTACTCAGTGAAACTGATCTC

CCCGTGAAGAAGCGGGGATAAAAATATAAGACGAGAAGACCCCATGGAGCTTTAAACTCA

GTACCAACTGCCCAAAT--AATAGCCTATTACCCCT-GCAG-CTATGGT-TACTAGTTTT

CGGTTGGGGTGACCACGGAGTAAAATTTAACCTCCACGATGAAAGGAACTAATATCCTAA

CCTATGAGCTACAGCTCTAAGTATCAAC--------AAATTGACTAA--TTGACCCAATT

ACTT--GATCAACGAACCAAGTTACCCTGGGGATAACAGCGCAATCCATTTCAAGAGCTC

ATATCGACAAATGGGTTTACG-ACC-TCGATGTTGGATCAGGGTATCCCAGTG-GCGCAG

CCGCTA-CTAA-CGGTTC

>M. mukhlesuri MN534689, MN534481, MN534582

TTATTATCACCTCCCTCTCGACTTACACATGCAAGTTTCAGCACGCCCGTGAGAACGCCC

TTCACCCTT-TTTAGGACAAGGAGCTGGTATCAGGCACAG---ATT------TCTAGCCC

ATAACACCTAGCTTTGCCACGCCCTCAAGGGTCTTCAGCAGTGATTAACATTGTTTATGA

GCGCCAGCTTGAATCAGTCAGAGAACCA--AGAGCCGGCCAATACGGTGCCAGCCGCCGC

GGCTACACCGCTAGGCTCAAGTTGATATT-ATTCGGCGTTAAGCGTGTTTAAAGTGCTC-

AAACAGATTAGAATTAAACTTTAACCAAGCCGTGATACGCTTGTTTCTAAGAAAACCGCA

ATCGAAAGTTATTCTAACCAAACCACTTGAATCCACGACAGCTAGGGAACAAACTGGGAT

TAGGTACCCCACTATGCCTAGCCGTAAAATATTTACTTACACTC-TTAACCGCCCGGGAA

TTACGAGCCCAAGCTTAAAACCCAAAGGACTTGACGGTGTCCCACCCACCTAGAGGAGCC

TGTTCTATAA-TCGATTCCCCC-GATCTACCTCACCATTTCTAGCCTT--TCAGCCTGT

ATACCTCCGTCGTAAGCTTACCATATGAACGTT-TCTCAGTGAGCTAAAAGAT-TTCCTC

GTAAATACGTCAGGTCAAGGTGCAGCCCACGAAATGGCAAGCAATGGGCTACAATTTCTA

CCA---TAGAACATAACGAAAGACTACATGCAA-CTCAGTCATGAAGGCGGATTTAGCAG

TAAAAAGAAAGTAGAGAGTTCTTTTTAATTAGGCGCTGGGACGCGTACACACCGCCCGTC

ACCCTCTTCAAACGCTTTA-TATATG-TTTATAACACT-TTTATGCATCACAGAAGAGGT

AAGTCGTAACATGGTAAGTGTACTGGAAAGTGCACTTGGATTAA-CAAAATGTAGCTTAA

CTAAA-GCATCTCGCTTACACCGAGAACATGTCCGTGAAACTCAGATCATTTTGA--GCT

GAAAACCTAGCTTAA--CTTATTCT-TATGAATATAC-CACCTTAA-CACTTA--CTAAA

TAAAACATTTTGTAC-ATTAAGTAAAGGCGATTAAAAAATGTCTT-AAAGCTATAGAAAC

TAGTACCGCAAGGGAAAAGTGAAATATTAATGAAAAA--CCTCAAGCACAAAAAAGTAGA

GCTACAACCTCGTACCTTTTGCATCATGGTCTAACTAGTTCAACCAAGCAAAATGAATTT

TAAGTTTGACTTCCCGAAACTAGGTGAGCTACTTAAAAACAGCCTTAC-GGGCCAACCCG

TCTCTGTTGCAAAAGAGTGGGAAGATTTT-TAAGTAGAGGTGACAGACCTATCGAACCTA

GAGATAGCTGGTTATTCAGGAAAAGGATTTAAGTCCTACCTTAAGTTTACT-GTACA-TA

A-TAATACACAATT-AACTTTAAGAGCTATTCAAATAAGGTACAGCCTATT-GAAACAGG

ATACAACCTCCATAA---

----------AAAAAGCGTTAAAGCTTCATTG--TTTTTTATCAAAAAATACCACTA-AT

TAACTGTAACCCTT-CACCT-GTACTGAATGATCTCATAGTTATATGAGAGCCTATATGT

TAGAACTAGTAACAAGAAGA-AGCCCTTCTCCA-AAATGTAAGCATGAGCCAAAATGAAC

TATTCACTGGCATTTAAAGTTA-TAAACCTATTGTAGCAACTTA----ACAAGAAAAATC

TAC------------------AAATGCA-AACGTCAACCTTACACCAGCACATTTCTGGA

AAGATAAAAAGAATAGGAAGGAACTCGGCAAATAA-CTAACCCCGCCTGTTTACCAAAAA

CATCGCCTCCTGATCATCC---ATAGGAGGTCCAGCCTGCCCAGTGACTAA--GTTAAAC

GGCCGCGGTACCCTAACCGTGCAAAGGTAGCGCAATCACTTGTTCTTTAAATGAGGACTA

GTATGAACGGCATCACGAGGGTTATGCTGTCTCCCTACTCTACTCAGTGAAACTGATCTC

CCCGTGAAGAAGCGGGGATAAAAATATAAGACGAGAAGACCCCATGGAGCTTTAAACTCA

GTGCCAACTGCCCTAAT--AATAACCTATTACCCTT-GCAG-CCATGGT-TACTAGTTTT

CGGTTGGGGTGACCACGGAGTAAAATTTAACCTCCACGATGAAAGGAACTAATATCCTAA

CCTATGAGCTACAGCTCTAAGTATCAAC--------AAATTGACTAA--TTGACCCAATT

ACTT--GATCAACGAACCAAGTTACCCTGGGGATAACAGCGCAATCCATTTCAAGAGCTC

ATATCGACAAATGGGTTTACG-ACC-TCGATGTTGGATCAGGGTATCCCAGTG-GCGCAG

CCGCTA-CTAA-CGGTTC

>M. mukhlesuri MN534690, MN534482, MN534583

TTATTATCACCTCCCTCTCGACTTACACATGCAAGTTTCAGCACACCCGTGAGAACGCCC

TTCACCCTT-TTTAGGACAAGGAGCTGGTATCAGGCACAG---ACT------TCTAGCCC

ATGACACCTAGCTTTGCCACACCCTCAAGGGTCTTCAGCAGTGATTAACATTGTTTATAA

GCGCCAGCTTGAATCAGTCAGAGAACCA--AGAGCCGGCCAATACGGTGCCAGCCGCCGC

GGCTACACCGCTAGGCTCAAGTTGATATT-ATTCGGCGTTAAGCGTGTTTAAAGTGCCCA

ATATAGATTAGAATTAAACTTTAACCAAGCCGTGATACGCTTGTTTCTAAGAAAACCATA

ATCGAAAGTTATTCTAACCAAACCACTTGAACCCACGACAGCTAGGGAACAAACTGGGAT

TAGGTACCCCACTATGCCTAGCCGTAAAACATTTACTTACACCC-TTAACCGCCCGGGAA

TTACGAGCCCAAGCTTAAAACCCAAAGGACTTGACGGTGTCCCACCCACCTAGAGGAGCC

TGTTCTATAA-TCGATTCCCCCC-GATCTACCTCACCATTTCTAGCCTT--TCAGCCTGT

ATACCTCCGTCGTAAGCTTACCATATGAACGTT-TCTCAGTGAGCTAAAAGAT-TTCCTC

ATAAATACGTCAGGTCAAGGTGCAGCCCACGAAATGGTAAGCAATGGGCTACAATTTCTA

TCA---TAGAACATAACGAAAGACTACATGCAA-CTTAGTCATGAAGGCGGATTTAGCAG

TAAAAAGAAAATAGAGAGTTCTTTTTAATTAGGCGCTGGGACGCGTACACACCGCCCGTC

ACCCTCTTCAAACGCTTTA-AATATG-TTTATAACACT-TTTATGCATCACAGAAGAGGT

AAGTCGTAACATGGTAAGTGTACTGGAAAGTGCACTTGGATTAA-CAAAATGTAGCTTAA

CTAAA-GCATCTCGCTTACACCGAGAACATGTCCGTGAAACTCAGATCATTTTGA--GCT

GAAAACCTAGCTTAC--TTTATTCT-TATGAATATAC-CACCTTAA-CACATA--CTAAA

TAAAACATTTTGTAC-ATTGAGTAAAGGCGATTAAAAAATGTCTA-AAAGCTATAGAAAC

TAGTACCGCAAGGGAAAAGTGAAATATTAATGAAAAA--CCTCAAGCACAAAAAAGTAGA

GCTATAACCTCGTACCTTTTGCATCATGGTCTAACTAGTTCAACCAAGCAAAATGAATTT

TAAGTTTGACTTCCCGAAACTAGGTGAGCTACTTAAAAACAGCCTTAC-GGGCCAACCCG

TCTCTGTTGCAAAAGAGTGGGAAGATTTTTTAAGTAGAGGTGACAGACCTACCGAACCTA

GAGATAGCTGGTTATTCAGGAAAAGGATTTAAGTCCTACCTTAAGTTTACT-GTACA---

--AG

CCATCTTTC-AAAAAGCGTTAAAGCTTCATTG---TTTTTATCAAAAA-TACCATTA-AT

TAACTGTAACCCTT-CACCT-GTACTGAATGATCTCATAATTGTATGAGAGCCTATATGT

TAGAACTAGTAACAAGAAGA-AGCCCTTCTCCA-AAATGTAAGCATAAGCCAAAATGAAC

TATTCATTGGCATTTAAAGTTA-TAAACCCATTGTAGCAACTTA----ACAAGAAAAACC

TAC------------------AACTGCA-AACGTCAACCTTACACCAGCACATTTCTGGA

AAGATAAAAAGAATAGGAAGGAACTCGGCAAATAA-CTAACCCCGCCTGTTTACCAAAAA

CATCGCCTCCTGATCACCC---ATAGGAGGTCCAGCCTGCCCAGTGACTAA--GTTAAAC

GGCCGCGGTACCCTAACCGTGCAAAGGTAGCGCAATCACTTGTTCTTTAAATGAGGACTA

GTATGAACGGCATCACGAGGGTTATGCTGTCTCCCTACTCTACTCAGTGAAACTGATCTC

CCCGTGAAGAAGCGGGGATAAAAATATAAGACGAGAAGACCCCATGGAGCTTTAAACTCA

GTACCAACTGCCCCAAT--AATAACCTATTATCCTT-GCAG-CCATGGT-TACTAGTTTT

CGGTTGGGGTGACCACGGAGTAAAATTTAACCTCCACGATGAAAGGAACTAATATCCTAA

CCTATGAGCTACAGCTCTAAGTATCAAC--------AAATTGACTAA--TTGACCCAATT

ACTT--GATCAACGAACCAAGTTACCCTGGGGATAACAGCGCAATCCATTTCAAGAGCTC

ATATCGACAAATGGGTTTACG-ACC-TCGATGTTGGATCAGGGTATCCCAGTG-GCGCAG

CCGCTA-CTAA-CGGTTC

>M. mukhlesuri MN534691, MN534483, MN534584

TTATTATCACCTCCCTCTCGACTTACACATGCAAGTTTCAGCACACCCGTGAGAACGCCC

TTCACCCTT-TTTAGGACAAGGAGCTGGTATCAGGCACAG---ACT------TCTAGCCC

ATGACACCTAGCTTTGCCACACCCTCAAGGGTCTTCAGCAGTGATTAACATTGTTTATAA

GCGCCAGCTTGAATCAGTCAGAGAACCA--AGAGCCGGCCAATACGGTGCCAGCCGCCGC

GGCTACACCGCTAGGCTCAAGTTGATATT-ATTCGGCGTTAAGCGTGTTTAAAGTGCCCA

ATATAGATTAGAATTAAACTTTAACCAAGCCGTGATACGCTTGTTTCTAAGAAAACCATA
ATCGAAAGTTATTCTAACCAAACCACTTGAACCCACGACAGCTAGGGAACAAACTGGGAT
TAGGTACCCCACTATGCCTAGCCGTAAAACATTTACTTACACCC-TTAACCGCCCGGGAA
TTACGAGCCCAAGCTTAAAACCCAAAGGACTTGACGGTGTCCCACCCACCTAGAGGAGCC
TGTTCTATAA-TCGATTCCCCCC-GATCTACCTCACCATTTCTAGCCTT--TCAGCCTGT
ATACCTCCGTCGTAAGCTTACCATATGAACGTT-TCTCAGTGAGCTAAAAGAT-TTCCTC
ATAAATACGTCAGGTCAAGGTGCAGCCCACGAAATGGTAAGCAATGGGCTACAATTTCTA
TCA---TAGAACATAACGAAAGACTACATGCAA-CTTAGTCATGAAGGCGGATTTAGCAG
TAAAAAGAAAATAGAGAGTTCTTTTTAATTAGGCGCTGGGACGCGTACACACCGCCCGTC
ACCCTCTTCAAACGCTTTA-AATATG-TTTATAACACT-TTTATGCATCACAGAAGAGGT
AAGTCGTAACATGGTAAGTGTACTGGAAAGTGCACTTGGATTAA-CAAAATGTAGCTTAA
CTAAA-GCATCTCGCTTACACCGAGAACATGTCCGTGAAACTCAGATCATTTTGA--GCT
GAAAACCTAGCTTAC--TTTATTCT-TATGAATATAC-CACCTTAA-CACATA--CTAAA
TAAAACATTTTGTAC-ATTGAGTAAAGGCGATTAAAAAATGTCTA-AAAGCTATAGAAAC
TAGTACCGCAAGGGAAAAGTGAAATATTAATGAAAAA--CCTCAAGCACAAAAAAGTAGA
GCTATAACCTCGTACCTTTTGCATCATGGTCTAACTAGTTCAACCAAGCAAAATGAATTT
TAAGTTTGACTTCCCGAAACTAGGTGAGCTACTTAAAAACAGCCTTAC-GGGCCAACCCG
TCTCTGTTGCAAAAGAGTGGGAAGATTTT-TAAGTAGAGGTGACAGACCTACCGAACCTA
GAGATAGCTGGTTATTCAGGAAAAGGATTTAAGTCCTACCTTAAGTTTACT-GTACA-TT
A-TAATACACAA--
---AGCAG
CCATCTTTC-AAAAAGCGTTAAAGCTTCATTG---TTTTTATCAAAAAATACCATTA-AT
TAACTGTAACCCTT-CACCT-GTACTGAATGATCTCATAATTGTATGAGAACCTATATGT
TAGAACTAGTAACAAGAAGA-AGCCCTTCTCCA-AAATGTAAGCATAAGCCAAAATGAAC
CATTCATTGGCATTTAAAGTTA-TAAACCCATTGTAGCAACTTA----ACAAGAAAAACC
TAC------------------AACTGCA-AACGTCAACCTTACACCAGCACATTTCTGGA
AAGATAAAAAGAATAGGAAGGAACTCGGCAAATAA-CTAACCCCGCCTGTTTACCAAAAA
CATCGCCTCCTGATCACCC---ATAGGAGGTCCAGCCTGCCCAGTGACTAA--GTTAAAC
GGCCGCGGTACCCTAACCGTGCAAAGGTAGCGCAATCACTTGTTCTTTAAATGAGGACTA
GTATGAACGGCATCACGAGGGTTATGCTGTCTCCCTACTCTACTCAGTGAAACTGATCTC

CCCGTGAAGAAGCGGGGATAAAAATATAAGACGAGAAGACCCCATGGAGCTTTAAACTCA

GTACCAACTGCCCCAAT--AATAACCTATTATCCTT-GCAG-CCATGGT-TACTAGTTTT

CGGTTGGGGTGACCACGGAGTAAAATTTAACCTCCACGATGAAAGGAACTAATATCCTAA

CCTGTGAGCTACAGCTCTAAGTATCAAC--------AAATTGACTAA--TTGACCCAATT

ACTT--GATCAACGAACCAAGTTACCCTGGGGATAACAGCGCAATCCATTTCAAGAGCTC

ATATCGACAAATGGGTTTACG-ACC-TCGATGTTGGATCAGGGTATCCCAGTG-GCGCAG

CCGCTA-CTAA-CGGTTC

>M. mukhlesuri MN534692, MN534484, MN534585

TTATTATCACCTCCCTCTCGACTTACACATGCAAGTTTCAGCACACCCGTGAGAACGCCC

TTCACCCTT-TTTAGGACAAGGAGCTGGTATCAGGCACAG---ACT------TCTAGCCC

ATGACACCTAGCTTTGCCACGCCCTCAAGGGTATTCAGCAGTGATTAACATTGTTTATGA

GCGCCAGCTTGAATCAGTCAGAGAACCA--AGAGCCGGCCAATACGGTGCCAGCCGCCGC

GGCTACACCGCTAGGCTCAAGTTGATATT-ATTCGGCGTTAAGCGTGTTTAAAGTGCCC-

AAACAGATTAGAGTTAAACTTTAACCAAGCCGTGATACGCTTGTTTCTAAGAAAACCACA

ATCGAAAGTTATTCTAACCAAACCACTTGAACCCACGACAGCTAGGGAACAAACTGGGAT

TAGGTACCCCACTATGCCTAGCCGTAAAACATTTACTTACACTC-TTAACCGCCCGGGAA

TTACGAGCCCAAGCTTAAAACCCAAAGGACTTGACGGTGTCCCACCCACCTAGAGGAGCC

TGTTCTATAA-TCGATTCCCCCC-GATCTACCTCACCATTTCTAGCCTT--TCAGCCTGT

ATACCTCCGTCGTAAGCTTACCATATGAACGTT-TCTCAGTGAGCTAAAAGAT-TTCTTG

TAAAATACGTCAGGTCAAGGTGCAGCCCACGAAATGGTAAGCAATGGGCTACAATTTCTA

CCA---TAGTACATAGCGAAAGACTACATCAAA-CTGAGTCATGAAGGCGGATTTAGCAG

TAAAAGAAAAGTAGAGAGTTCTTTTTAATTAGGCGGTGGGACGCGTACACACCGCCCCTC

ACCCTCTTCAACCGCTTTA-AATATG-TTTATAATACT-TTTATGCATCACAGAAGAGGT

AAGTCGTAACATGGTAAGTGTACTGGAAAGTGCAATTGGATTAA-CAAAATGTAGCTTAA

CTAAA-GCATCTCGCTTACACCGAGAACATGTCCGTGAAACTCAGATCATTTTGA--GCT

GAAAACCTAGCTTAC--TTTATTCT-TATGAATATAC-CACCTTAA-CACACA--CTAAA

TAACGCATTTTGTAC-ACTAAGTAAAGGCGATTAAAAGATGTCTT-AAAGCTATAGAAAC

TAGTACCGCAAGGGAAAAGTGAAATATTAGTGAAAAA--CCTCAAGCACAAAAAAGTAGA

GCTATAGCCTCGTACCTTTTGCATCATGGTCTAACTAGTTCAATCAAGCAAAATGAATTT

TAAGTTTGACTTCCCGAAACTAGGTGAGCTACTTAAAAACAGCCTTAC-GGGCCAACCCG

TCTCTGTTGCAAAAGAGTGGGAAGATTTT-TAAGTAGAGGTGACAGACCTACCGAACCTA
GAGATAGCTGGTTATTCAGGAAAAGGATTTAAGTCCTACCTTAAGTTTACT-GTACA-TA
A-TAATACATAATT-AACTTTAAGAGCTATTCAAATAAGGTACAGCCTATTTGAAACAGG
ATACAACCTCC---CAG
CCACCTTAC-AAAAAGCGTTAAAGCTTCATTG---TTTTTATCAAAAA-TACCATAA-AT
CAACTGTAACCCTT-CACCT-GTACTGAATGATCTCATAATTGTATGAGAGCCTATATGT
TAGAACTAGTAACAAGAAGA-AGCCCTTCTCCA-AAATGTAAGCATGAGCCAAAATGAAC
TATTCATTGGCATTTAAAGTTA-TAAACCTATTGTAGCAACTTA----ACAAGAAAAATC
TAC------------------AACTGTA-TACGTCAACCTTACACCAGCACATCTCTGGA
AAGATAAAAAGAATAGGAAGGAACTCGGCAAATAA-CTAACCCCGCCTGTTTACCAAAAA
CATCGCCTCCTGATTACCC---ATAGGAGGTCCAGCCTGCCCAGTGACTAA--GTTAAAC
GGCCGCGGTACCCTAACCGTGCAAAGGTAGCGCAATCACTTGTTCTTTAAATGAGGACTA
GTATGAACGGCATCACGAGGGTTATGCTGTCTCCCTACTCTACTCAGTGAAACTGATCTC
CCCGTGAAGAAGCGGGGATAAAAATATAAGACGAGAAGACCCCATGGAGCTTTAAACTCA
GTACCAACTGCCCTAGT--AATAACCTATTACCCTT-GCAG-TCATGGT-TACTAGTTTT
CGGTTGGGGTGACCACGGAGTAAAACTTAACCTCCACGATGAAAGGAACTAATATCCTAA
CCTATGAGCTACAGCTCTAAGTATCAAC--------AAATTGACTAA--CTGATCCAATT
ACTT--GATCAACGAACCAAGTTACCCTGGGGATAACAGCGCAATCCATTTCAAGAGCTC
ATATCGACAAATGGGTTTACG-ACC-TCGATGTTGGATCAGGGTATCCCAGTG-GCGCAG
CCGCTA-CTAA-TGGTTC
>M. mukhlesuri MN534693, MN534485, MN534586
TTATTATCACCTCCCTCTCGACTTACACATGCAAGTTTCAGCACACCCGTGAGAACGCCC
TTCACCCTT-TTTAGGACAAGGAGCTGGTATCAGGCACAG---ATT------TCTAGCCC
ATGACACCTAGCTTTGCCACGCCCTCAAGGGTCTTCAGCAGTGATTAACATTGTTTATAA
GCGCCAGCTTGAATCAGTCAGAGAACCA--AGAGCCGGCCAATACGGTGCCAGCCGCCGC
GGCTACACCGCTAGGCTCAAGTTGATATT-ATTCGGCGTTAAGCGTGTTTAAAGTGCCC-
AAACAGATTAGAATTAAACTTTAACCAAGCCGTGATACGCTTGTTTCTAAGAAAACCACA
ATCGAAAGTTATTCTAACCAAACCACTTGAACCCACGACAGCTAGGGAACAAACTGGGAT
TAGGTACCCCACTATGCCTAGCCGTAAAACATTTACTTACACCC-TTAACCGCCCGGGAA
TTACGAGCCCAAGCTTAAAACCCAAAGGACTTGACGGTGTCCCACCCACCTAGAGGAGCC

TGTTCTATAA-TCGATTCCCCCC-GATCTACCTCACCATTTCTAGCCTT--TCAGCCTGT

ATACCTCCGTCGTAAGCTTACCATATGAACGTT-TCTCAGTGAGCTTAAAGAT-TTCTTC

GTAAATACGTCAGGTCAAGGTGCAGCCCACGAAATGGCAAGCAATGGGCTACAATTTCTA

TCA---TAGAACATAACGAAAGACTACATGCAA-CTCAGTCATGAAGGCGGATTTAGCAG

TAAAAAGAAAGTAGAGAGTTCTTTTTAATTAGGCGCTGGGACGCGTACACACCGCCCGTC

ACCCTCTTCAAACGCTTTA-AATATG-TTTATAACACT-TTTATGCATCACAGAAGAGGT

AAGTCGTAACATGGTAAGTGTACTGGAAAGTGCACTTGGATTAA-CAAAATGTAGCTTAA

CTAAA-GCATCTCGCTTACACCGAGAACATGTCCGTGAAACTCAGATCATTTTGA--GCT

GAAAACCTAGCTTAC--TTTATTCT-TATGAACATAC-TACCTTAT-TGTATA--CTAAA

TAAAACATTTTGTAC-ATTAAGTAAAGGCGATTAAAAAATGTCTT-AAAGCTATAGAAAC

CAGTACCGCAAGGGAAAAGTGAAATATAAATGAAAAA--CCTCAAGCACAAAAAAGTAGA

GCTATAACCTCGTACCTTTTGCATCATGGTCTAACTAGTTCAACCAAGCAAAATGAATTT

TAAGTTTGACTTCTCGAAACTAGGTGAGCTACTTAAAAACAGCCTTAC-GGGCCAACCCG

TCTCTGTTGCAAAAGAGTGGGAAGATTTTTTAAGTAGAGGTGACAGACCTACCGAACCTA

GAGATAGCTGGTTATTCAGGAAAAGGATTTAAGTCCTACCTTAAGTTTACT-GTACA---

--------C-AAAAAGCGTTAAAGCTTCATTG---TTTTTATCAAAAA-TACCATTA-AT

CTACTGTAACCCTT-CACCT-GTACTGAATGATCTCATAATTATATGAGAGCCTATATGT

TAGAACTAGTAACAAGAAGA-AGCCCTTCTCCA-AAATGTAAGCATGAGCCAAAATGAAC

TATTCATTGGCATTTAAAGTTA-TAAACCCATTGTAGCAACTTA----ACAAGAAAAACC

TAC------------------AACTGCA-AACGTCAACCTTACACCAGCACATTTCTGGA

AAGATAAAAAGAATAGGAAGGAACTCGGCAAATAA-CTAACCCCGCCTGTTTACCAAAAA

CATCGCCTCCTGACTACCC---ATAGGAGGTCCAGCCTGCCCAGTGACTAA--GTTAAAC

GGCCGCGGTACCCTAACCGTGCAAAGGTAGCGCAATCACTTGTTCTTTAAATGAGGACTA

GTATGAACGGCATCACGAGGGTTATGCTGTCTCCCTACTCTATTCAGTGAAACTGATCTC

CCCGTGAAGAAGCGGGGATAAAGATATAAGACGAGAAGACCCCATGGAGCTTTAAACTCA

GTACCAACTGCCCGCAT--AATAACCTATTACCCTT-GCAG-CCATGGT-TACTAGTTTT

CGGTTGGGGTGACCACGGAGTAAAATTTAACCTCCACGATGAAAGGAACTAATGTCCTAA

CCTATGAGCTACAGCTCTAAGTATCAAC--------AAATTGACTAA--TTGACCCAATT

ACTT--GATCAACGAACCAAGTTACCCTGGGGATAACAGCGCAATCCATTTCAAGAGCTC

ATATCGACAAATGGGTTTACG-ACC-TCGATGTTGGATCAGGGTATCCCAGTG-GCGCAG

CCGCTA-CTAA-CGGTTC

>M. mukhlesuri MN534694, MN534486, MN534587

TTATTATCACCTCCCTCTCGACTTACACATGCAAGTTTCAGCACACCCGTGAGAACGCCC

TTCACCCTT-TTTAGGACAAGGAGCTGGTATCAGGCACAG---ACT------TCTAGCCC

ATGACACCTAGCTTTGCCACACCCTCAAGGGTCTTCAGCAGTGATTAACATTGTTTATAA

GCGCCAGCTTGAATCAGTCAGAGAACCA--AGAGCCGGCCAATACGGTGCCAGCCGCCGC

GGCTACACCGCTAGGCTCAAGTTGATATT-ATTCGGCGTTAAGCGTGTTTAAAGTGCCCA

ATATAGATTAGAATTAAACTTTAACCAAGCCGTGATACGCTTGTTTCTAAGAAAACCATA

ATCGAAAGTTATTCTAACCAAACCACTTGAACCCACGACAGCTAGGGAACAAACTGGGAT

TAGGTACCCCACTATGCCTAGCCGTAAAACATTTACTTACACCC-TTAACCGCCCGGGAA

TTACGAGCCCAAGCTTAAAACCCAAAGGACTTGACGGTGTCCCACCCACCTAGAGGAGCC

TGTTCTATAA-TCGATTCCCCCC-GATCTACCTCACCATTTCTAGCCTT--TCAGCCTGT

ATACCTCCGTCGTAAGCTTACCATATGAACGTT-TCTCAGTGAGCTAAAAGAT-------

--------------------------------------AAGCAATGGGCTACAATTTCTA

CCA---TAGAACATAACGAAAGACTACATGCAA-CTTAGTCATGAAGGCGGATTTAGCAG

TAAAAAGAAAGTAGAGAGTTCTTTTTAATTAGGCGCTGGGACGCGTACACACCGCCCGTC

ACCCTCTTCAAACGCTTTA-AATATG-TTTATAACACT-TTTATGCATCACAGAAGAGGT

AAGTCGTAACATGGTAGGTGTACTGGAAAGTGTACTTGGATTAA-CAAAATGTAGCTTAA

CTAAA-GCATCTCGCTTACACCGAGAACATGTCTGTGAAACTCAGATCATTTAGA--GCT

GAAAACCTAGCTTAC--TTTATTTT-TATGAATATAC-CACCATAA-CACATA--CTAAA

TAAAACATTTTGTAC-ATTGAGTAAAGGCGATTAAAAAATGTCTA-AAAGCTATAGAAAC

TAGTACCGCAAGGGAAAAGTGAAATATTAATGAAAAA--CCTCAAGCACAAAAAAGTAGA

GCTATAACCTCGTACCTTTTGCATCATGGTCTAACTAGTTCAACCAAGCAAAATGAATTT

TAAGTTTGACTTCCCGAAACTAGGTGAGCTACTTAAAAACAGCCTTAC-GGGCCAACCCG

TCTCTGTTGCAAAAGATTTGGAAAATTTT-TAAGTAGAGGTGACAGACCTACCGAACCTA

GAGATAGCTGGTTATTCAGGAAAAGGATTTATTTCCTACATTAAGTTTACT-GTACA-TT

A-TAATACACAACT-AACTTTAAGAGCTATTCAAATAAGGTACAGCCTATTTGAAGCAGG

ATACAACCTCCACA--

---TATGAGAGCCTATATGT
AAGAACTAGTAACAAGAAGA-AGCCCTAATCCA-AAATGTAAGCATAAGCCAAAATGAAC
TATTCATTGGCATTTAAAGTTA-TAAACCCATTGTAGCAACTTA----ACAAGAAAAACC
TAC------------------AACTGCA-AACGTCAACCTTACACCAGCACATTTCTGGA
AAGATAAAAAGAATAGGAAGGAACTCGGCAAATAA-CTAACCCCGCCTGTTTACCAAAAA
CATCGCCTCCTGATCACCC---ATAGGAGGTCCAGCCTGCCCAGTGACTAA--GTTAAAC
GGCCGCGGTACCCTAACCGTGCAAAGGTAGCGCAATCACTTGTTCTTTAAATGAGGACTA
GTATGAACGGCATCACGAGGGTTATGCTGTCTCCCTACTCTACTCAGTGAAACTGATCTC
CCCGTGAAGAAGCGGGGATAAAAATATAAGACGAGAAGACCCCATGGAGCTTTAAACTCA
GTACCAACTGCCCCAAT--AATAACCTATTATCCTT-GCAG-CCATGGT-TACTAGTTTT
CGGTTGGGGTGACCACGGAGTAAAATTTAACCTCCACGATGAAAGGAACTAATATCCTAA
CCTGTGAGCTACAGCTCTAAGTATCAAC--------AAATTGACTAA--TTGACCCAATT
ACTT--GATCAACGAACCAAGTTACCCTGGGGATAACAGCGCAATCCATTTCAAGAGCTC
ATATCGACAAATGGGTTTACG-ACC-TCGATGTTGGATCAGGGTATCCCAGTG-GCGCAG
CCGCTA-CTAA-TGGTTC

>M. fissipes MN534695, MN534487, MN534588

TTATTATCACCTCATTCTCGACTTACACATGCAAGTTTCAGCACACCCGTGAGAACGCCC
TTCACCCTT-TTTAGGACAAGGAGCTGGTATCAGGCACAG---AAC------TCTAGCCC
ATGACACCTAGTTTTGCCACACCCTCAAGGGTCTTCAGCAGTGATTAACATTGTTTATAA
GCGCCAGCTTGAACCAGTTAGAGAACCC--AGAGCCGGCCAATACGGTGCCAGCCGCCGC
GGCTACACCGCTAGGCTCAAGTTGATATTATTTCGGCGTTAAGCGTGTTTAAAGTGCCCG
TAACAGATTAGAATTAAACTTTAACCAAGCCGTGATACGCTTGTTTCTAAGAAAACCACA
ATCGAAAGTTATTCTAACCAAACCACTTGAACCCACGACAGCTAGGGAACAAACTGGGAT
TAGATACCCCACTATGCCTAGCCGTAAAACATTTACTTACACCC-CTTACCGCCCGGGAA
TTACGAGCCCAAGCTTAAAACCCAAAGGACTTGACGGTGTCCCACCCACCTAGAGGAGCC
TGTTCTATAA-TCGATTCCCCCC-GATCTACCTCACCACTTCTAGCTTT-TTCAGCCTGT
ATACCTCCGTCGTAAGCTTACCATATGAACGTT-TCTCAGTGAGCTAAAAGAT-TTCTTC
ATAAATACGTCAGGTCAAGGTGCAGCCCACGAAGTGGCAAGCAATGGGCTACAATTTCTA
TAA---TAGAACATAACGAAAGACTACATGCAA-CTCAGTCATGAAGGCGGATTTAGCAG

TAAAAAGAAAGTAGAGAGTTCTTTTTAATTAGGCGCTGGGACGCGTACACACCGCCCGTC

ACCCTCTTCAAACGCTTTA-AATATG-TTTATAACACT-TTTATGCACCCCAGAAGAGGT

AAGTCGTAACATGGTAAGTGTACTGGAAAGTGCACTTGGATAAA-CAAAATGTAGCTTAA

CTAAA-GCATCTCGCTTACACCGAGAACATGTCCGTGAAACTCAGATCATTTTGA--GCT

GAAAACCTAGCTTAC--CCAATTCC-TATGAATATAC-CACCTTAA-CATAAA--CTAAA

TAAAACATTTTGTAC-ATTTAGTAAAGGCGATTAAAAAATGTCTC-AAAGCTATAGAAAC

TAGTACCGCAAGGGAAAAGTGAAATATTAGTGAAAAA--CCTCAAGCACAAAAAAGTAGA

GCTATAACCTCGTACCTTTTGCATCATGGTCTAACTAGTTCAACCAAGCAAAATGAATTT

TAAGTTTGACTTCCCGAAACTAAGTGAGCTACTTAAAAACAGCCCTCC-GGGCCAACCCG

TCTCTGTTGCAAAAGAGTGGGAAGATTTT-TAAGTAGAGGTGATAGACCTACCGAACCTA

GAGATAGCTGGTTATTCAGGAAAAGGATTTAAGTCCTACCTTAAGTTTACA-GTATA---

--GCAG

CCATCTTAC-AAAAAGCGTTAAAGCTTCATTG--TTTTTTATCAAAAAATACCATTA-AT

TAACTGTAACCCTT-CACCT-GTACTGAATGATCTCATAGTTATATGAGAGCCTATATGT

TAGAACTAGTAACAAGAAGA-AGCCCTTCTCCA-AAATGTAAGCGTAAGCCAAAATGAAC

TATTCATTGGCATTTAAAGTTA-TAAACCCATTGTAGCAACTTA----ACAAGAAAAACC

TAC------------------AACTGAT-AACGTCAACCTTACACAAGCACATTTCTGGA

AAGATAAAAAGAATAGGAAGGAACTCGGCAAACAA-CTAACCCCGCCTGTTTACCAAAAA

CATCGCCTCCTGATCACCC---ATAGGAGGTCCAGCCTGCCCAGTGACTAA--GTTAAAC

GGCCGCGGTACCCTAACCGTGCAAAGGTAGCGCAATCACTTGTTCTTTAAATGAGGACTA

GTATGAACGGCATCACGAGGGTTATGCTGTCTCCCTACTCTACTCAGTGAAACTGATCTC

CCCGTGAAGAAGCGGGGATAAAAATATAAGACGAGAAGACCCCATGGAGCTTTAAACTCA

GTACCAACTGCCCAAAT--AAGAACCTATTATACTT-GCAG-CTATGGT-TACTAGTTTT

CGGTTGGGGTGACCACGGAGTAAAATTAAACCTCCACGATGAAAGGAACTAATAACCTAA

CCTATGAGCTACAGCTCTAAGTATCAAC--------AAATTGACTAA--TTGACCCAATT

ACTT--GATCAACGAACCAAGTTACCCTGGGGATAACAGCGCAATCCATTTCAAGAGCTC

ATATCGACAAATGGGTTTACG-ACC-TCGATGTTGGATCAGGGTATCCCAGTG-GCGCAG

CCGCTA-CTAA-CGGTTC

>M. fissipes MN534696, MN534488, MN534589

TTATTATCACCTCATTCTCGACTTACACATGCAAGTTTCAGCACACCCGTGAGAACGCCC

TTCACCCTT-TTTAGGACAAGGAGCTGGTATCAGGCACAG---AAC------TCTAGCCC

ATGACACCTAGTTTTGCCACACCCTCAAGGGTCTTCAGCAGTGATTAACATTGTTTATAA

GCGCCAGCTTGAACCAGTTAGAGAACCC--AGAGCCGGCCAATACGGTGCCAGCCGCCGC

GGCTACACCGCTAGGCTCAAGTTGATATTATTTCGGCGTTAAGCGTGTTTAAAGTGCCCG

TAACAGATTAGAATTAAACTTTAACCAAGCCGTGATACGCTTGTTTCTAAGAAAACCACA

ATCGAAAGTTATTCTAACCAAACCACTTGAACCCACGACAGCTAGGGAACAAACTGGGAT

TAGGTACCCCACTATGCCTAGCCGTAAAACATTTACTTACACCC-CTTACCGCCCGGGAA

TTACGAGCCCAAGCTTAAAACCCAAAGGACTTGACGGTGTCCCACCCACCTAGAGGAGCC

TGTTCTATAA-TCGATTCCCCCC-GATCTACCTCACCACTTCTAGCTTT-TTCAGCCTGT

ATACCTCAGTCGTAAGCTTACCATATGAACGTT-TCTCAGTGAGATAAAAGAT-TTCTTC

ATAAATACGTCAGGTCAAGGTGCAGCCCACGAAGTGGCAAGCAATGGGCTACAATTTCTA

TAA---TAGAACATAACGAAAGACTACATGCAA-CTCAGTCATGAAGGCGGATTTAGCAG

TAAAAAGAAAGTAGAGAGTTCTTTTTAATTAGGCGCTGGGACGCGTACACACCGCCCGTC

ACCCTCTTCAAACGCTTTA-AATATG-TTTATAACACT-TTTATGCACCCCAGAAGAGGT

AAGTCGTAACATGGTAAGTGTACTGGAAAGTGCACTTGGATAAA-CAAAATGTAGCTTAA

CTAAA-GCATCTCGCTTACACCGAGAACATGTCCGTGAAACTCAGATCATTTTGA--GCT

GAAAACCTAGCTTAC--CCAATTCC-TATGAATACAC-CACCTTAA-CATAAA--CTAAA

TAAAACATTTTGTAC-ATTTAGTAAAGGCGATTAAAAAATGTCTC-AAAGCTATAGAAAC

TAGTACCGCAAGGGAAAAGTGAAATATTAGTGAAAAA--CCTCAAGCACAAAAAAGTAGA

GCTATAACCTCGTACCTTTTGCATCATGGTCTAACTAGTTCAACCAAGCAAAATGAATTT

TAAGTTTGACTTCCCGAAACTAAGTGAGCTACTTAAAAACAGCCCTCC-GGGCCAACCCG

TCTCTGTTGCAAAAGAGTGGGAAGATTTT-TAAGTAGAGGTGATAGACCTACCGAACCTA

GAGATAGCTGGTTATTCAGGAAAAGGATTTAAGTCCTACCTTAAGTTTACA-GTATA---

--GCAG

CCATCTTAC-AAAAAGCGTTAAAGCTTCATCG---TTTTTATCAAAAAATACCATTA-AT

TAACTGTAACCCTT-CACCT-GTACTGAATGATCTCATAGTTATATGAGAGCCTATATGT

TAGAACTAGTAACAAGAAGA-AGCCCCTCTCCA-AAATGTAAGCGTAAGCCAAAATGAAC

TATTCATTGGCATTTAAAGTTA-TAAACCTATTGTAGCAACTTA----ACAAGAAAAACC

TAC------------------AACTGATAAACGTCAACCTTACACAAGCACATTTCTGGA

AAGATAAAAAGAATAGGAAGGAACTCGGCAAACAA-CTAACCCCGCCTGTTTACCAAAAA

CATCGCCTCCTGATCACCC---ATAGGAGGTCCAGCCTGCCCAGTGACTAA--GTTAAAC

GGCCGCGGTACCCTAACCGTGCAAAGGTAGCGCAATCACTTGTTCTTTAAATGAGGACTA

GTATGAACGGCATCACGAGGGTTATGCTGTCTCCCTACTCTACTCAGTGAAACTGATCTC

CCCGTGAAGAAGCGGGGATAAAAATATAAGACGAGAAGACCCCATGGAGCTTTAAACTCA

GTACCAACTGCCCAAAT--AAGAACCTATTATACTT-GCAG-CTATGGT-TACTAGTTTT

CGGTTGGGGTGACCACGGAGTAAAATTAAACCTCCACGATGAAAGGAACTAATAACCTAA

CCTATGAGCTACAGCTCTAAGTATCAAC--------AAATTGACTAA--TTGACCCAATT

ACTT--GATCAACGAACCAAGTTACCCTGGGGATAACAGCGCAATCCATTTCAAGAGCTC

ATATCGACAAATGGGTTTACG-ACC-TCGATGTTGGATCAGGGTATCCCAGTG-GCGCAG

CCGCTA-CTAA-CGGTTC

>M. chakrapanii MN534697, MN534489, MN534590

TTACTATCACCCCCTTCTCGACTTACACATGCAAGTATCAGCACACCCGTGAGAACGCCC

TTCACCCTT-CTTAGGACAAGGAGCCGGTATCAGGCACAG---AAC------CCTAGCCC

ATGACACCTAGCTTTGCCACACCCTCAAGGGTCTTCAGCAGTGATTAACATTGTTTATAA

GCGCCAGCTTGAATCAGTTAGAGAACCA--AGAGCCGGCCAATACGGTGCCAGCCGCCGC

GGCTACACCGCTAGGCTCAAGTTGATAGT-ATTCGGCGTTAAGCGTGTTTAAAGTGCTT-

AAACAGATTAGAATCAAACTTTAACCAAGCCGTGATACGCTTGTTTTTAAGAAAACCACA

ATCGAAAGTTATTCTAACCAAACCACTTGAACCCACGACAGCTAGGAAACAAACTGGGAT

TAGGTACCCCACTATGCCTAGCCGTAAAATATTTATTCACACCT-TTAACCGCCTGGGAA

TTACGAGCCCAAGCTTAAAACCCAAAGGACTTGACGGTGTCCCACCCACCTAGAGGAGCC

TGTTCTATAA-TCGATTCCCCCC-GATCAACCTCACCATTTCTAGCCTC--TCAGCCTGT

ATACCTCCGTCGTAAGCTTACCATATGAACGTT-TCTCAGTGAGCTAAAAGAT-TTCTTC

ATAAATACGTCAGGTCAAGGTGCAGCCCACGAAATGGCAAGCAATGGGCTACAATTTCTA

AAA---TAGAACATAACGAAAGACTACATGCAA-CTCAGTAATGAAGGCGGATTTAGCAG

TAAAAAGAAAATAGAGAGTTCTTTTTAATTAGGCGCTGGGACGCGTACACACCGCCCGTC

ACCCTCTTCAAACGCTTTA-AACATG-TTTATAACACT-TTTATGCACCACAGAAGAGGT

AAGTCGTAACATGGTAAGCGTACTGGAAAGTGCGCTTGGATAAA-CAAAATGTAGCTTAA

CTAAA-GCATCTCGCTTACACCGAGAACATGTCCGTTAAACTCAGATCATTTTGA--GCT

AAAAACCTAGCTTAC-CTTTATTCT-TATGAATACAC-CACCTTAA-CAAAA---CTAAA

TAAAACATTTTGTAC-ATTAAGTAAAGGCGATTAAAAAATGTCTC-AAAGCTATAGAAAC

TAGTACCGCAAGGGAAAAATGAAATATTAATGAAAAA--CCTCAAGCACAAAAAAGTAGA

GCTATAACCTCGTACCTTTTGCATCATGGTCTAACTAGTTCAACCAAGCAAAATGAATTT

TAAGTCTGACTTCCCGAAACTAGGTGAGCTACTTAAAAACAGCCTTCC-GGGCCAACCCG

TCTCTGTTGCAAAAGAGTGGGAAGATTTT-TAAGTAGAGGTGACAGACCTACCGAACCTA

GAGATAGCTGGTTATTCAGGAAAAGGATCTAAGTCCTACCTTAAGTTTATT-GTACA-AG

AACAATACACTATT-AAC--

----------------CGTTAAAGCTTCATTG---TTTTTATCAAAAA-TACCATCA-AT

TATCTGTAACCCTT-CATCT-GTACTGAATGATTTCATAATTATATGAAAGCCTATATGT

TAGAACTAGTAACAAGAAGA-AGCCCTTCTCCA-AAATGTAAGCATAAGCCAAAATGAAC

TATTCATTGGCATTTAAAGTTA-TAAACCTATTGTAGCAACTCA----ACAAGAAAAACC

TAC------------------AACTGCA-AACGTCAACCTTACACCAGCACATTTCTGGA

AAGATAAAAAGAATAGGAAGGAACTCGGCAAATAA-CTAACCCCGCCTGTTTACCAAAAA

CATCGCCTCCTGATCACCC---ATAGGAGGTCCAGCCTGCCCAGTGACTAA--GTTAAAC

GGCCGCGGTACCCTAACCGTGCAAAGGTAGCACAATCACTTGTTCTTTAAATGAGGACTA

GTATGAATGGCATCACGAGGGTTATGCTGTCTCCCTATTCCACTCAGTGAAACTGATCTC

CCCGTGAAGAAGCGGGGATAAAAATATAAGACGAGAAGACCCCATGGAGCTTTAAACTCA

GTACCAACTGCCTAATT--AAGAACCTTTCATCCCT-GCAG-CCATGGT-TACTAGTTTT

CGGTTGGGGTGACCACGGAGTAAAATTAAACCTCCATGATGAAAGGAACTAACAACCTAA

CCTATGAGCTACAGCTCTAAGCATCAAT--------AAATTGACTAA--TTGATCCAATC

AGTT--GATCAACGAACCTAGTTACCCTGGGGATAACAGCGCAATCCATTTCAAGAGCTC

ATATCGACAAATGGGTTTACG-ACC-TCGATGTTGGATCAGGGTATCCCAGTG-GCGCAG

CCGCTA-CTAA-CGGTTC

>M. chakrapanii MN534698, MN534490, MN534591

TTACTATCACCCCCTTCTCAACTTACACATGCAAGTATCAGCACACCCGTGAGAACGCCC

TTCACCCTT-TTTAGGACAAGGAGCCGGTATCAGGCACAG---AAC------CCTAGCCC

ATGACACCTAGCTTTGCCACACCCTCAAGGGTCTTCAGCAGTGATTAACATTGTTTATAA

GCGCCAGCTTGAACCAGTTAGAGAACCA--AGAGCCGGCCAATACGGTGCCAGCCGCCGC

GGCTACACCGCTAGGCTCAAGTTGATAGT-ACTCGGCGTTAAGCGTGTTTAAAGTGCTT-

AAACAGATTAAAATTAAACTTTAACCAAGCCGTGATACGCTTGTTCCTAAGAAAACCGCA

ATCGAAAGTTATTCTAACCAAACCACTTGAACCCACGACAGCTAGGAAACAAACTGAGAT

TAGGTACCTCACTATGCCTAGCCGTAAAATATTTACTCACACCT-TTAACCGCCCGGGAA

TTACGAGCCCAAGCTTAAAACCCAAAGGACTTGACGGTGTCCCACCCACCTAGAGGAGCC

TGTTCTATAA-TCGATTCCCCCC-GATCTACCTCACCATTTCTAGCCTC--TCAGCCTGT

ATACCTCCGTCGTAAGCTTACCATATGAACGTT-TCTCAGTGAGCTAAAAGAT-TTCTTC

ATAAATACGTCAGGTCAAGGTGCAGCCCACGAAATGGCAAGCAATGGGCTACAATTTCTA

CAA---TAGAACATAACGAAAGACTACATGCAA-CTCAGTCATGAAGGCGGATTTAGCAG

TAAAAAGAAAATAGAGAGTTCTTTTTAATTAGGCGCTGGGACGCGTACACACCGCCCGTC

ACCCTCTTCAAACGCTTTA-AACATG-TTTATAACACT-TTTATGCACCACAGAAGAGGT

AAGTCGTAACATGGTAAGTGTACTGGAAAGTGCACTTGGATAAA-CAAAATGTAGCTTAA

CTAAA-GCATCTCGCTTACACCGAGAACATGTCCGTTAAACTCAGATCATTTTGA--GCT

AAAAACCTAGCTTAC-CTTTATTCT-TATGAATACAC-CACCTTAA-CAAAA---CTAAA

TAAAACATTTTGTAC-ATTAAGTAAAGGCGATTAAAAAATGTCTC-AAAGCTATAGAAAC

TAGTACCGCAAGGGAAAAATGAAATATTAATGAAAAA--CCTCAAGCACAAAAAAGTAGA

GCTATAACCTCGTACCTTTTGCATCATGGTCTAACTAGTTCAACCAAGCAAAATGAATTT

TAAGTCTGACTTCCCGAAACTAGGTGAGCTACTTAAAAACAGCCTTCC-GGGCCAACCCG

TCTCTGTTGCAAAAGAGTGGGAAGATTTT-TAAGTAGAGGTGACAGACCTACCGAACCTA

GAGATAGCTGGTTATTCAGGAAAAGGATCTAAGTCCTACCTTAAGTTTATT-GTACA-AG

AACAATACACTATT-AAC--

----------------CGTTAAAGCTTCATTG---TTTTTATCAAAAA-TACCATCA-AT

TAACTGTAACCCTT-CATCT-GTACTGAATGATTTCATAATTATATGAAAGCCTATATGT

TAGAACTAGTAACAAGAAGA-AGCCCTTCTCCA-AAATGTAAGCATAAGCCAAAATGAAC

TACTCATTGGCATTTAAAGTTA-TAAACCTATTGTAGCAACTTA----ACAAGAAAAACC

TAC------------------AACTGCA-AACGTCAACCTTACACCAGCACATTTCTGGA

AAGATAAAAAGAATAGGAAGGAACTCGGCAAATAA-CTAACCCCGCCTGTTTACCAAAAA

CATCGCCTCCTGATCACCC---ATAGGAGGTCCAGCCTGCCCAGTGACTAA--ATTAAAC

GGCCGCGGTACACTAACCGTGCAAAGGTAGCGCAATCACTTGTTCTTTAAATGAGGACTA

GTATGAATGGCATCACGAGGGTTATGCTGTCTCCCTATTCCACTCAGTGAAACTGATCTC

CCCGTGAAGAAGCGGGGATAAAAATATAAGACGAGAAGACCCCATGGAGCTTTAAACTCA

GTACCAACTGCCTAATT--AAGAACCTTTCATCCCT-GCAG-CCATGGT-TACTAGTTTT

CGGTTGGGGTGACCACGGAGTAAAATTAAACCTCCATGATGAAAGGAACTAACAACCTAA

CCTATGAGCTACAGCTCTAAGCATCAAT--------AAATTGACTAA--TTGATCCAATC

AGTT--GATCAACGAACCTAGTTACCCTGGGGATAACAGCGCAATCCATTTCAAGAGCTC

ATATCGACAAATGGGTTTACG-ACC-TCGATGTTGGATCAGGGTATCCCAGTG-GCGCAG

CCGCTA-CTAA-------

>M. mymensinghensis MN534699, MN534491, MN534592

TTACTATCACCCCCTTCTCAACTTACACATGCAAGTATCAGCATACCCGTGAGAACGCCC

TTCATCCTT-TTTAGGACAAGGAGCCGGTATCAGGCACAG---AAT------TCTAGCCC

ATGACACCTAGCTTTGCCACACCCTCAAGGGTCTTCAGCAGTGATTAACATTGTTCATAA

GCGCCAGCTTGAATCAGTTAGAGAGCCA--AGAGCCGGCCAATACGGTGCCAGCCGCCGC

GGCTACACCGCTAGGCTCAAGTTGATAGT-ATTCGGCGTTAAGCGTGTTTAAAGTG-CCG

-AACAGATTAGAATTAAACTTTAACCAAGCCGTGATACGCTTGTTTCTAAGAAAACCACA

ATCGAAAGTTATTCTAACCAAACCACTTGAACCCACGACAGCTAGAAAACAAACTGGGAT

TAGGTACCCCACTATGCCTAGCCGTAAAATATTTACTTACACCT-TTAACCGCCTGGGAA

TTACGAGCCCAAGCTTAAAACCCAAAGGACTTGACGGTGTCCCACCCACCTAGAGGAGCC

TGTTCTATAA-TCGATTCCCCCC-GATATACCTCACCAAAAATAAAACT-TTCAGCCTGT

ATACCTCCGTCGTAAGCTTACCATATGAACGTT-TCTCAGTGAGCTAAAAGAT-TTCTTC

ATAAATACGTCAGGTCAAGGTGCAGCCCACGAAATGGCAAGCAATGGGCTACAATTTCTA

CAA---TAGAACATAACGAAAGACTACATGCAA-CTCAGTCATGAAGGCGGATTTAGCAG

TAAAAAGAAAATAGAGAGTTCTTTTTAATTAGGCGCTGGGACGCGTACACACCGCCCGTC

ACCCTCTTCAAACGCTTAT-AATATG-TCTGTAACACT-TTTATGCACCACAGAAGAGGT

AAGTCGTAACATGGTAAGTGTACTGGAAAGTGCACTTGGATAAA-CAAAATGTAGCTTAA

CTAAA-GCATCTCGCTTACACCGAGAACATGTCCGTGAAACTCAGATCATTTTGA--GCT

AAAAACCTAGCTTAA--CCCATTCT-TATGAATACAC-CTCCTAA--CAAAAA--CTGAA

TAAAACATTTTGTAT-ATTAAGTAAAGGCGATTAAAAAATATTTC-AAAGCTATAGAAAC

CAGTACCGCAAGGGAAAAGTGAAATATTAGTGAAAAA--TCTTAAGCACAAAAAAGTAGA

GTTATAGCCTCGTACCTTTTGCATCATGGTCTAACTAGTTCAACCAAGCAAAATGAATTT

TAAGTCTGACTTCCCGAAATTAGGTGAGCTACTTAAAGACAGCCTTCC-GGGCCAACCCG

TCTCTGTTGCAAAAGAGTGGGAAGATCTT-TAAGTAGAGGTGACAGACCTACCGAACCTA

AAGATAGCTGGTTATTCAGGAAAAGGATCTAAGTCCTACCTTAAGTTTATT-GTACA-AG

AATAATACACTACT-AACTTTAAGAGCTATTCAAATAAGGTACAGCCTATTTGAACCAGG

ATACAACCTCCACAATCGGGTAACATCTTTTTTTA-TCAACTAAGTGGGCCTAAAAGCAG

CCACCTTAC-AAAAAGCGTTAAAGCTTCATTG---TCCCTATTAAAAA-TACCATTA-AC

TAACTGTAACCCTT-CATTT-GTACTGAATGATCCCATAATTATATGAGAGCCTATATGT

TAGAACTAGTAACAAGAAGA-AGTTCTTCTCCA-AAATGTAAGCATAAGCCAAAATGAAC

TATTCACTGGCATTTAAAGTTA-TAAACCCATTGTAGCAACTTA----ACAAGAAAAACC

TAC------------------AACTGCA-AACGTTAACCTTACACCAGCGCATTTCTGGA

AAGATAAAAAGAATAGGAAGGAACTCGGCAAATAA-CTAACCCCGCCTGTTTACCAAAAA

CATCGCCTCCTGATCACCC---ATAGGAGGTCCAGCCTGCCCAGTGACTAA--GTTAAAC

GGCCGCGGTACCCTAACCGTGCAAAGGTAGCGCAATCACTTGTTCTTTAAATGAGGACTA

GTATGAACGGCATCACGAGGGTTATGCTGTCTCCCTATTCTATTCAGTGAAACTGATCTC

CCCGTGAAGAAGCGGGGATAAAAATATAAGACGAGAAGACCCCATGGAGCTTTAAACTCA

GTACCAACTGCCCAATT--AAAAACCTTTTACCCCT-GCAG-CTATGGT-TACTAGTTTT

CGGTTGGGGTGACCACGGAGTAAAAATCAACCTCCACGATGAAAGGAACTAACAACCTAA

CCTATGAGCTACAGCTCTAAGTATCAAT--------AAATTGACTAA--TTGATCCAATT

ACTT--GATCAACGAACCTAGTTACCCTGGGGATAACAGCGCAATCCATTTCAAGAGCTC

ATATCGACAAATGGGTTTACGTACC-TCGATGTTGGATCAGGGTATCCCAGTG-GCGCAG

CCGCTA-CTAA-CGGTTC

>Microhyla sp. 3 MN534700, MN534492, MN534593

TTATTATCATCTTTCCCTCGACTTACACATGCAAGTATCAGCATACCCGTGAGAACGCCC

TTTACCCTT-CTTAGGATAAGGAGCTGGTATCAGGCACAG---AAT------TCTAGCCC

ATGACACCTAGCTTTGCCACACCCTCAAGGGCCTTCAGCAGTGATTAACATTGTTCATAA

GCGCCAGCTTGAATCAGTCAGAGAACTT--AGAGCCGGCCAATACGGTGCCAGCCGCCGC

GGCTATACCGCTAGGCTCAAGTTGATATT-ATTCGGCGTTAAGCGTGTTTAAAGTGACC-

AAAA-GAATAAAATTAAATTTAAACCAAGCCGTGACACGCTTGTTTCTAAGAAAAACAGA

AACGAAAGTTATTTTAACCAACCCACTTGAACCCACGACAGCTAGAAAACAAACTGGGAT

TAGGTACCCCACTATGCCTAGCCGTAAAATATTTATTTACACCC-CTAACCGCCCGGGAA

TTACGAGCCCAAGCTTAAAACCCAAAGGACTTGACGGTGTCCCACCCACCTAGAGGAGCC

TGTTCTATAA-TCGATTCCCCCC-GATCTACCTCACCACTTCTAGCCTC--TCAGCCTGT

ATACCTCCGTCGTAAGCTTACCATATGAACGAT-TTTCAGTGAGCTAAAAGAT--TTTAC

ATAAATACGTCAGGTCAAGGTGCAGCCCACGATGTGGAAAGAAATGGGCTACAATTTCTA

TAA---TAGAACATAACGAAAGACTACATGCAA-CTCAGTCATGAAGGCGGATTTAGTAG

TAAAAAGAAAGTAGAGAGTTCTTTTTAACCAGGCGCTGGGACGCGTACACACCGCCCGTC

ACCCTCTTCAAACGCTTTA-ACTATG-TTTATAACACT-TTTATGCACTACAGAAGAGGT

AAGTCGTAACACGGTAAGTGTACTGGAAAGTGCACTTGGATAAA-CAAAATGTAGCTTAA

CTAAA-GCATCTCGCTTACACCGAGAACATGTCTGTGAAACCCAGATCATTTTGA--GCT

AAAAACTTAGCTTAT--CTAATTCT-TATGTACTCCC-CTCCCCCA-CAAATAAACTCAA

TAAAACATTTTTCAC-ATTAAGTAAAGGCGATTAAAAAATGTCTA-AAAGCTATAGAAAC

TAGTACCGCAAGGGAAAAGTGAAATAAAAATGAAAAA--CCTCAAGCACAAAAAAGCAGA

GTTACAATCTCGTACCTTTTGCATCATGGTCTAACTAGTCTAACCAAGCAAAATGAATTT

TAAGTTTGACCCCCCGAAACTAGATGAGCTACTTAAAAACAGCCTTT-AGGGCCAACCCG

TCCATGTTGCAAAATGGTGGGAAGATTTT-TAAGTAGAAGTGATAGACCTACCGAATCTA

GAGATAGCTGGTTATTCAGGAAAAGGATTTTAGTCCTACCTTAGGTTTACT-ATATAGTT

TACAATACTACCAC-AACCTTAAGAGCTATTCAAATAAGGTACAGCCTATTTGAAACAGG

ATACAACCTCTACT----------------------------AAGTGGGCCTAAAAGCAG

CCACCTTAC-AAAAAGCGTTAAAGCTTCATTGG---TTAAATTAAAAA-TTCCATTA-AT

TTTTTACAACCCTT-AATCT-GTACTGAATGATCTCATAATATTATGAGAGCCTATATGT

TAGAACTAGTAACAAGAAGA-AGTCCTTCTCCA-AAATGTAAGCATGAGCCAAAATGAAC

AATTCATTGGCAATTAAAGTTA-AATATTTATTGTAGTAACCCA----ACAAGAAAAACC

TAC-----------------TAATTATT-AGCGTCAACCTTACACTAGTACATTTCTGGA

AAGATTAAAAGAATGGGAAGGAACTCGGCAAATAA-TTAACCCCGCCTGTTTACCAAAAA

CATCGCCTCCTGATATTTT---ATAGGAGGTCCAGCCTGCCCAGTGACAAA--GTTAAAC

GGCCGCGGTACCCTAACCGTGCAAAGGTAGCGCAATCACTTGTTCTTTAAATGAGGACTA

GTATGAACGGCATCACGAGGGTTATGCTGTCTCCCCACTCTATTCAGTGAAACTGATTTT

CCCGTGAAGAAGCGGGAATAAAAATATAAGACGAGAAGACCCCATGGAGCTTTAAGCTCA

GTATCAACTGCCTAACT-AAAAAACCTTATATTTT--GCAG-CCATGCT-TACTAGATTT

CGGTTGGGGTGACCACGGAGTAAAACTAAACCTTCATGATGAAAGGAACTAATAACCTAA

CCTATGAGCTACAGCTCTAAGTATCAAC--------AAATTGACTAAA-TTGACCCAATT

ACTT--GATCAACAAACCAAGTTACCCTGGGGATAACAGCGCAATCCATTCCAAGAGCTC

ATATCGACAAATGGGTTTACG-ACC-TCGATGTTGGATCAGGGTATCCCAGTG-GCGCAG

CTGCTA-CTAA-CGGTTC

>Microhyla sp. 3 MN534701, MN534493, MN534594

TTATTATCATCTTTCCCTCGACTTACACATGCAAGTATCAGCATACCCGTGAGAACGCCC

TTTACCCTT-CTTAGGATAAGGAGCTGGTATCAGGCACAG---AAT------TCTAGCCC

ATGACACCTAGCTTTGCCACACCCTCAAGGGCCTTCAGCAGTGATTAACATTGTTCATAA

GCGCCAGCTTGAATCAGTCAGAGAACTT--AGAGCCGGCCAATACGGTGCCAGCCGCCGC

GGCTATACCGCTAGGCTCAAGTTGATATT-ATTCGGCGTTAAGCGTGTTTAAAGTGACC-

AAAA-GATTAAAATTAAATTTAAACCAAGCCGTGACACGCTTGTTTCTAAGAAAAACAAA

AACGAAAGTTATTTTAACCAAACCACTTGAACCCACGACAGCTAGGAAACAAACTGGGAT

TAGGTACCCCACTATGCCTAGCCGTAAAATATTTATTTACACCC-CTAACCGCCCGGGAA

TTACGAGCCCAAGCTTAAAACCCAAAGGACTTGACGGTGTCCCACCCACCTAGAGGAGCC

TGTTCTATAA-TCGATTCCCCCC-GATCTACCTCACCACTTCTAGCCTC--TCAGCCTGT

ATACCTCCGTCGTAAGCTTACCATATGAACGAT-TTTCAGTGAGCTAAAAGAT--TTTAC

ATAAATACGTCAGGTCAAGGTGCAGCCCACGATGTGGAAAGAAATGGGCTACAATTTCTA

TAA---TAGAACATAACGAAAGACTACATGCAA-CTCAGTCATGAAGGCGGATTTAGTAG

TAAAAAGAAAGTAGAGAGTTCTTTTTAACCAGGCGCTGGGACGCGTACACACCGCCCGTC

ACCCTCTTCAAACGCTTTA-ACTATG-TTTATAACACT-TTTATGCACTACAGAAGAGGT

AAGTCGTAACACGGTAAGTGTACTGGAAAGTGCACTTGGATAAA-CAAAATGTAGCTTAA

CTAAA-GCATCTCGCTTACACCGAGAACATGTCTGTGAAACCCAGATCATTTCGA--GCT

AAAAACTTAGCTTAT--CTAATTCT-TATGTACTCCC-CTCCCCCA-CAAATAAACTCAA

TAAAACATTTTTCAC-ATTAAGTAAAGGCGATTAAAAAATGTCTA-AAAGCTATAGAAAC

TAGTACCGCAAGGGAAAAGTGAAATAAAAATGAAAAA--CTTCAAGCACAAAAAAGCAGA

GTTACATTCTGGTACCTTTTGCATCATGGTCTAACTTGTCTAACCAAGCAAAATGAATTT

TAAGTTTGGCCCCCCGAAACTAGATGAGCTACTTAAAAACAGCCTTT-AGGGCCAACCCG

TCCATGTTGCAAAATGGTGGGAAGATTTT-TAAGTAGAGGTGATAGACCTACCGAATCTA

GAGATAGCTGGTTATTCAGGAAAAGGATTTTAGTCCTACCTTAGGTTTACT-ATATAGTT

TACAATACTACCAC-AACCTTAAGAGCTATTCAAATAAGGTACAGCCTATTTGAAACAGG

ATACAACCTCTACT----------------------------AAGTGGGCCTAAAAGCAG
CCACCTTAC-AAAAAGCGTTAAAGCTTCATTGG---TTAAATTAAAAA-TTTCATTAAAT
TAATTACAACCCCT-AATCT-GTACTGAATGATCTCATAATATTATGAGAGCCTATATGT
TAGAACTAGTAACAAGAAGA-AGTCCTTCTCCA-AAATGTAAGCATGAGCCAAAATGAAC
AATTCATTGGCAATTAAAGTTA-AATATTTATTGTAGTAACCCA----ACAAGAAAAACC
TAC-----------------TAATTATT-AGCGTCAACCTTACACTAGTACATTTCTGGA
AAGATTAAAAGAATGGGAAGGAACTCGGCAAATAA-TTAACCCCGCCTGTTTACCAAAAA
CATCGCCTCCTGATATTTT---ATAGGAGGTCCAGCCTGCCCAGTGACAAA--GTTAAAC
GGCCGCGGTACCCTAACCGTGCAAAGGTAGCGCAATCACTTGTTCTTTAAATGAGGACTA
GTATGAACGGCATCACGAGGGTTATGCTGTCTCCCCACTCTATTCAGTGAAACTGATTTT
CCCGTGAAGAAGCGGGAATAAAAATATAAGACGAGAAGACCCCATGGAGCTTTAAACTCA
GTATCAACTGCCTAACT-AAAAAACCTTATATTTT--GCAG-CCATGCT-TACTAGATTT
CGGTTGGGGTGACCACGGAGTAAAACTAAACCTCCATGATGAAAGGAACTAATAACCTAA
CCTATGAGCTACAGCTCTAAGTATCAAC--------AAATTGACTAAA-TTGACCCAATT
ACTT--GATCAACAAACCAAGTTACCCTGGGGATAACAGCGCAATCCATTTCAAGAGCTC
ATATCGACAAATGGGTTTACG-ACC-TCGATGTTGGATCAGGGTATCCCAGTG-GCGCAG
CTGCTA-CTAA-CGGTTC

>Microhyla sp. 3 MN534702, MN534494, MN534595

TTATTATCATCTTTCCCTCGACTTACACATGCAAGTATCAGCATACCCGTGAGAACGCCC
TTTACCCTT-CTTAGGATAAGGAGCTGGTATCAGGCACAG---AAT------TCTAGCCC
ATGACACCTAGCTTTGCCACACCCTCAAGGGCCTTCAGCAGTGATTAACATTGTTCATAA
GCGCCAGCTTGAATCAGTCAGAGAACTT--AGAGCCGGCCAATACGGTGCCAGCCGCCGC
GGCTATACCGCTAGGCTCAAGTTGATATT-ATTCGGCGTTAAGCGTGTTTAAAGTGACC-
AAAA-GATTAAAATTAAACTTAAACCAAGCCGTGACACGCTTGTTTCTAAGAAAAACAAA
AACGAAAGTTATTTTAACCAAACCACTTGAACCCACGACAGCTAGGAAACAAACTGGGAT
TAGGTACCCCACTATGCCTAGCCGTAAAATATTTATTTACACCC-CTAACCGCCCGGGAA
TTACGAGCCCAAGCTTAAAACCCAAAGGACTTGACGGTGTCCCACCCACCTAGAGGAGCC
TGTTCTATAA-TCGATTCCCCCC-GATCTACCTCACCACTTCTAGCCTC--TCAGCCTGT
ATACCTCCGTCGTAAGCTTACCATATGAACGAT-TTTCAGTGAGCTAAAAGAT--TTTAC
ATAAATACGTCAGGTCAAGGTGCAGCCCACGATGTGGAAAGAAATGGGCTACAATTTCTA

TAA---TAGAACATAACGAAAGACTACATGCAA-CTCAGTCATGAAGGCGGATTTAGTAG

TAAAAAGAAAGTAGAGAGTTCTTTTTAACCAGGCGCTGGGACGCGTACACACCGCCCGTC

ACCCTCTTCAAACGCTTTA-ACTATG-TTTATAACACT-TTTATGCACTACAGAAGAGGT

AAGTCGTAACACGGTAAGTGTACTGGAAAGTGCACTTGGATAAA-CAAAATGTAGCTTAA

CTAAA-GCATCTCGCTTACACCGAGAACATGTCTGTGAAACCCAGATCATTTTGA--GCT

AAAAACTTAGCTTAT--CTAATTCT-TATGTACTCCC-CCCCCCA-CAAATAAACTCAA

TAAAACATTTTTCAC-ATTAAGTAAAGGCGATTAAAAAATGTCTA-AAAGCTATAGAAAC

TAGTACCGCAAGGGAAAAGTGAAATAAAAATGAAAAA--CCTCAAGCACAAAAAAGCAGA

GTTACAATCTCGTACCTTTTGCATCATGGTCTAACTAGTCTAACCAAGCAAAATGAATTT

TAAGTTTGACCCCCCGAAACTAGATGAGCTACTTAAAAACAGCCTTT-AGGGCCAACCCG

TCCATGTTGCAAAATGGTGGGAAGATTTT-TAAGTAGAGGTGATAGACCTACCGAATCTA

GAGATAGCTGGTTATTCAGGAAAAGGATTTTAGTCCTACCTTAGGTTTACT-ATATAGTT

TACAATACTACCAC-AACCTTAAGAGCTATTCAAATAAGGTACAGCCTATTTGAAACAGG

ATACAACCTCTACT----------------------------AAGTGGGCCTAAAAGCAG

CCACCTTAC-AAAAAGCGTTAAAGCTTCATTGG--TTTAAATTAAAAA-TTCCATTAAAT

TTTTTACAACCCTT-AATCT-GTACTGAATGATCTCATAATATTATGAGAGCCTATATGT

TAGAACTAGTAACAAGAAGA-AGTCCTTCTCCA-AAATGTAAGCATGAGCCAAAATGAAC

AATTCATTGGCAATTAAAGTTA-AATATTTATTGTAGTAACCCA----ACAAGAAAAACC

TAC-----------------TAATTATT-AGCGTCAACCTTACACTAGTACATTTCTGGA

AAGATTAAAAGAATGGGAAGGAACTCGGCAAATAA-TTAACCCCGCCTGTTTACCAAAAA

CATCGCCTCCTGATATTTT---ATAGGAGGTCCAGCCTGCCCAGTGACAAA--GTTAAAC

GGCCGCGGTACCCTAACCGTGCAAAGGTAGCGCAATCACTTGTTCTTTAAATGAGGACTA

GTATGAACGGCATCACGAGGGTTATGCTGTCTCCCCACTCTATTCAGTGAAACTGATTTT

CCCGTGAAGAAGCGGGAATAAAAATATAAGACGAGAAGACCCCATGGAGCTTTAAACTCA

GTATCAACTGCCTAACT-AAAAAACCTTATATTTT--GCAG-CCATGCT-TACTAGATTT

CGGTTGGGGTGACCACGGAGTAAAACTAAACCTCCATGATGAAAGGAACTAATAACCTAA

CCTATGAGTTACAGCTCTAAGTATCAAC--------AAATTGACTAAA-TTGACCCAATT

ACTT--GATCAACAAACCAAGTTACCCTGGGGATAACAGCGCAATCCATTTCAAGAGCTC

ATATCGACAAATGGGTTTACG-ACC-TCGATGTTGGATCAGGGTATCCCAGTG-GCGCAG

CTGCTA-CTAA-CGGTTC

>Microhyla sp. 3 MN534703, MN534495, MN534596

TTATTATCATCTTTCCCTCGACTTACACATGCAAGTATCAGCATACCCGTGAGAACGCCC

TTTACCCTT-CTTAGGATAAGGAGCTGGTATCAGGCACAG---AAT------TCTAGCCC

ATGACACCTAGCTTTGCCACACCCTCAAGGGCCTTCAGCAGTGATTAACATTGTTCATAA

GCGCCAGCTTGAATCAGTCAGAGAACTT--AGAGCCGGCCAATACGGTGCCAGCCGCCGC

GGCTATACCGCTAGGCTCAAGTTGATATT-ATTCGGCGTTAAGCGTGTTTAAAGTGACC-

AAAA-GATTAAAATTAAACTTAAACCAAGCCGTGACACGCTTGTTTCTAAGAAAAACAAA

AACGAAAGTTATTTTAACTAAACCACTTGAACCCACGACAGCTAGGAAACAAACTGGGAT

TAGGTACCCCACTATGCCTAGCCGTAAAATATTTATTTACACCC-CTAACCGCCCGGGAA

TTACGAGCCCAAGCTTAAAACCCAAAGGACTTGACGGTGTCCCACCCACCTAGAGGAGCC

TGTTCTATAA-TCGATTCCCCCC-GATCTACCTCACCACTTCTAGCCTC--TCAGCCTGT

ATACCTCCGTCGTAAGCTTACCATATGAACGAT-TTTCAGTGAGCTAAAAGAT--TTTAC

ATAAATACGTCAGGTCAAGGTGCAGCCCACGATGTGGAAAGAAATGGGCTACAATTTCTA

TAA---TAGAACATAACGAAAGACTACATGCAA-CTCAGTCATGAAGGCGGATTTAGTAG

TAAAAAGAAAGTAGAGAGTTCTTTTTAACCAGGCGCTGGGACGCGTACACACCGCCCGTC

ACCCTCTTCAAACGCTTTA-ACTATG-TTTATAACACT-TTTATGCACTACAGAAGAGGT

AAGTCGTAACACGGTAAGTGTACTGGAAAGTGCACTTGGATAAA-CAAAATGTAGCTTAA

CTAAA-GCATCTCGCTTACACCGAGAACATGTCTGTGAAACCCAGATCATTTCGA--GCT

AAAAACTTAGCTTAT--CTAATTCT-TATGTACTCCC-CTCCCCCA-CAAATAAACTCAA

TAAAACATTTTTCAC-ATTAAGTAAAGGCGATTAAAAAATGTCTA-AAAGCTATAGAAAC

TAGTACCGCAAGGGAAAAGTGAAATAAAAATGAAAAA--CCTCAAGCACAAAAAAGCAGA

GTTACAATCTCGTACCTTTTGCATCATGGTCTAACTAGTCTAACCAAGCAAAATGAATTT

TAAGTTTGACCCCCCGAAACTAGATGAGCTACTTAAAAACAGCCTTT-AGGGCCAACCCG

TCCATGTTGCAAAATGGTGGGAAGATTTT-TAAGTAGAGGTGATAGACCTACCGAATCTA

GAGATAGCTGGTTATTCAGGAAAAGGATTTTAGTCCTACCTTAGGTTTACT-ATATAGTT

TACAATACTACCAC-AACCTTAAGAGCTATTCAAATAAGGTACAGCCTATTTGAAACAGG

ATACAACCTCTACT----------------------------AAGTGGGCCTAAAAGCAG

CCACCTTAC-AAAAAGCGTTAAAGCTTCATTGG---TTAAATTAAAAA-TTTCATTAAAT

TAATTACAACCCCT-AATCT-GTACTGAATGATCTCATAATATTATGAGAGCCTATATGT

TAGAACTAGTAACAAGAAGA-AGTCCTTCTCCA-AAATGTAAGCATGAGCCAAAATGAAC

AATTCATTGGCAATTAAAGTTA-AATATTTATTGTAGTAACCCA----ACAAGAAAAACC

TAC-----------------TAATTATT-AGCGTCAACCTTACACTAGTACATTTCTGGA

AAGATTAAAAGAATGGGAAGGAACTCGGCAAATAA-TTAACCCCGCCTGTTTACCAAAAA

CATCGCCTCCTGATATTTT---ATAGGAGGTCCAGCCTGCCCAGTGACAAA--GTTAAAC

GGCCGCGGTACCCTAACCGTGCAAAGGTAGCGCAATCACTTGTTCTTTAAATGAGGACTA

GTATGAACGGCATCACGAGGGTTATGCTGTCTCCCCACTCTATTCAGTGAAACTGATTTT

CCCGTGAAGAAGCGGGAATAAAAATATAAGACGAGAAGACCCCATGGAGCTTTAAACTCA

GTATCAACTGCCTAACT-AAAAAACCTTATATTTT--GCAG-CCATGCT-TACTAGATTT

CGGTTGGGGTGACCACGGAGTAAAACTAAACCTCCATGATGAAAGGAACTAATAACCTAA

CCTATGAGCTACAGCTCTAAGTATCAAC--------AAATTGACTAAA-TTGACCCAATT

ACTT--GATCAACAAACCAAGTTACCCTGGGGATAACAGCGCAATCCATTTCAAGAGCTC

ATATCGACAAATGGGTTTACG-ACC-TCGATGTTGGATCAGGGTATCCCAGTG-GCGCAG

CTGCTA-CTAA-CGGTTC

>M. okinavensis MN534704, MN534496, MN534597

TTATTATCACCTCCCCCTCAACTTACACATGCAAGTATCAGCACACCCGTGAGAACGCCC

TTTACCCTT-CTTAGGATAAGGAGCTGGTATCAGGCACAG---AAT------TCTAGCCC

ACGACACCTAGCTTTGCCACACCCTCAAGGGTATTCAGCAGTGATTAACATTGTTTATAA

GCGCCAGCTTGAATCAGTCAGAGGGCCC--AGAGCCGGCCAATACGGTGCCAGCCGCCGC

GGCTACACCGCTAGGCTCAAGTTGATATT-ATTCGGCGTTAAGCGTGTTTAAAGTGCCC-

AAAAAGATTAAAATTAAACTTAAACCAAGCCGTGACACGCTTGTTTCTAAGAAAAACAAA

AACGAAAGTTATTTTAACCAACCCACTTGAACCCACGACAGCTAGGAAACAAACTGGGAT

TAGGTACCCCACTATGCCTAGCCGTAAAATATTTACTCACATCC-TTAACCGCCTGGGAA

TTACGAGCCCAAGCTTAAAACCCAAAGGACTTGACGGTGTCCCACCCACCTAGAGGAGCC

TGTTCTATAA-TCGATTCCCCCC-GATCTACCTCACCACTTCTAGCCTC--TCAGCCTGT

ATACGTCCGTCGTAAGCTTACCATATGAACGAC--CCCAGTGAGCTAAACTAT--TATTC

ATAAATACGTCAGGTCAAGGTGCAGCCCACGATGTGGGAAGAAATGGGCTACAATTTCTA

CAT---TAGAACACAACGAAAGACTACATGCAA-CTCAGTCATGAAGGCGGATTTAGTAG

TAAAAAGAAAGTAGAGAGTTCTTTTTAATTAGGCGCTGGGACGCGTACACACCGCCCGTC

ACCCTCTTCAAACGCTTTA-ATTACG-TTTATAACACT-TTTATGCACCACAGAAGAGGT

AAGTCGTAACATGGTAAGTGTACTGGAAAGTGCACTTGGATAAA-CAAAATGTAGCTTAA

CTAAA-GCATCTCGCTTACACCGAGAACATATCTGTGAGATCCGGATCATTTTGA--GCC

AAAAACTTAGCTCAA--CTAATTCC-TATGTATTCCC-CACCCACG--TAATAAACCCAA

TAAAACATTTTACAT-GTTTAGTAAAGGCGATTAAAAAATATCTA-AAAGCTATAGAAAC

TAGTACCGCAAGGGAAAAGTGAAATAAAAATGAAAAA--CTTCAAGCACAAAAAAGTAGA

GCTATAATCTCGTACCTTTTGCATCATGGTCTAACTAGTCTACCCAAGCAAAATGAATCT

TAAGTTTGACCCCCCGAAACTAGATGAGCTACTTAAAAACAGCCTTT-AGGGCCAACCCG

TCTCTGTTGCAAAAGAGTGGGAAGATTTT-TAAGTAGAGGTGATAAACCTACCGAACCTA

GAGATAGCTGGTTATTCAGGAAAAGGATTTAAGTCCTACCTTAGGTTTTTT-GTACA-TT

TATAGTACACCCTC-AACCTTAAGAGCTATTCAAATAAGGTACAGCCTATTTGAAACAGG

ATACAACCTCCACCAACGGGTAA-------------------AAGTGGGCCTAAAAGCAG

CCACTTTAC-AAAAAGCGTTAAAGCTTAGTTG---TCCTAACTAAAAA-TTCCTATA-AT

TAATTATAACCCTT-AATCT-GTACTGAATAATCTCATAGTATTATGAGAGCCTGTATGT

TAGAACTAGTAACAAGAAGA-AGCCCTTCTCCA-AAATGTAAGCATGAGCCAAAATGAAC

CATTCATTGGTACTTAAAGTTA-TAAACCCACTGTAGTAACCCA---AACAAGAAAAATC

TAC-----------------AAGTTATT-AACGTCAACCTAACACCAGTACATTTCTGGA

AAGATTAAAAGAATGGGAAGGAACTCGGCAAATAA-TTAACCCCGCCTGTTTACCAAAAA

CATCGCCTCCTGCCCTATT---ATAGGAGGTCCAGCCTGCCCAGTGACAAA--GTTAAAC

GGCCGCGGTACCCTAACCGTGCAAAGGTAGCGCAATCACTTGTTCTTTAAATGAGGACTA

GTATGAACGGCATCACGAGGGTTATGCTGTCTCCCCACTCTATTCAGTGAAACTGATCTT

CCCGTGAAGAAGCGGGAATAAAAATATAAGACGAGAAGACCCCATGGAGCTTTAAACTCA

GTAGCAACTGCCTAAAT--AAAAACCTCACATTCT--GCAG-CCGTGCT-TGCTAGTTTT

CGGTTGGGGTGACCACGGAGTAAAACTAAACCTCCATGATGAAAGGAATTAATATCCTAA

CCCACGAGCTACAGCTCTAAGTATTAAT--------AAATTAACTAAA-TTGACCCAATT

ACTT--GATCAACGAACCAAGTTACCCTGGGGATAACAGCGCAATCCATTTCAAGAGCTC

ATATCGACAAATGGGTTTACG-ACC-TCGATGTTGGATCAGGGTATCCCAGTG-GCGCAG

CTGCTA-CTAA-TGGTT-

>M. okinavensis MN534705, MN534497, MN534598

TTATTATCACCTCCCCCTCAACTTACACATGCAAGTATCAGCACACCCGTGAGAACGCCC

TTTACCCTT-CTTAGGATAAGGAGCTGGTATCAGGCACAG---AAT------TCTAGCCC

ACGACACCTAGCTTTGCCACACCCTCAAGGGTATTCAGCAGTGATTAACATTGTTTATAA

GCGCCAGCTTGAATCAGTCAGAGGGCCC--AGAGCCGGCCAATACGGTGCCAGCCGCCGC

GGCTACACCGCTAGGCTCAAGTTGATATT-ATTCGGCGTTAAGCGTGTTTAAAGTGCCC-

AAAAAGATTAAAATTAAACTTAAACCAAGCCGTGACACGCTTGTTTCTAAGAAAAACAAA

AACGAAAGTTATTTTAACCAACCCACTTGAACCCACGACAGCTAGGAAACAAACTGGGAT

TAGGTACCCCACTATGCCTAGCCGTAAAATATTTACTCACATCC-TTAACCGCCTGGGAA

TTACGAGCCCAAGCTTAAAACCCAAAGGACTTGACGGTGTCCCACCCACCTAGAGGAGCC

TGTTCTATAA-TCGATTCCCCCC-GATCTACCTCACCACTTCTAGCCTC--TCAGCCTGT

ATACGTCCGTCGTAAGCTTACCATATGAACGAC--CCCAGTGAGCTAAACTAT--TATTC

ATAAATACGTCAGGTCAAGGTGCAGCCCACGATGTGGGAAGAAATGGGCTACAATTTCTA

CAT---TAGAACACAACGAAAGACTACATGCAA-CTCAGTCATGAAGGCGGATTTAGTAG

TAAAAAGAAAGTAGAGAGTTCTTTTTAATTAGGCGCTGGGACGCGTACACACCGCCCGTC

ACCCTCTTCAAACGCTTTA-ATTACG-TTTATAACACT-TTTATGCACCACAGAAGAGGT

AAGTCGTAACATGGTAAGTGTACTGGAAAGTGCACTTGGATAAA-CAAAATGTAGCTTAA

CTAAA-GCATCTCGCTTACACCGAGAACATATCTGTGAGATCCGGATCATTTTGA--GCC

AAAAACTTAGCTCAA--CTAACTCC-TATGTATTCCC-CACCCACG--TAATAAA-CCAA

TAAAACATTTTACAT-GTTTAGTAAAGGCGATTAAAAAATATCTA-AAAGCTATAGAAAC

TAGTACCGCAAGGGAAAAGTGAAATAAAAATGAAAAA--CTTCAAGCACAAAAAAGTAGA

GCTATAATCTCGTACCTTTTGCATCATGGTCTAACTAGTCTACCCAAGCAAAATGAATTT

TAAGTTTGACCCCCCGAAACTAGATGAGCTACTTAAAAACAGCCTTT-AGGGCCAACCCG

TCTCTGTTGCAAAAGAGTGGGAAGATTTT-TAAGTAGAGGTGATAAACCTACCGAACCTA

GAGATAGCTGGTTATTCAGGAAAAGGATTTAAGTCCTACCTTAGGTTTTTT-GTACA-TT

TATAGTACACCCTT-AACCTTAAGAGCTATTCAAATAAGGTACAGCCTATTTGAAACAGG

ATACAACCTCCACCAACGGGTAA-------------------AAGTGGGCCTAAAAGCAG

CCACATTAC-AAAAAGCGTTAAAGCTTAGTTG---TCCTAACTAAAAA-TTCCTATA-AT

TAATTATAACCCTT-AATCT-GTACTGAATAATCTCATAGTATTATGAGAGCCTATATGT

TAGAACTAGTAACAAGAAGA-AGCCCTTCTCCA-AAATGTAAGCATGAGCCAAAATGAAC

CATTCATTGGTACTTAAAGTTA-TAAACCCACTGTAGTAACCCA---AACAAGAAAAATC

TAC-----------------AAGTTATT-AACGTCAACCTAACACCAGTACATTTCTGGA

AAGATTAAAAGAATGGGAAGGAACTCGGCAAATAA-TTAACCCCGCCTGTTTACCAAAAA

CATCGCCTCCTGCCCTATT---ATAGGAGGTCCAGCCTGCCCAGTGACAAA--GTTAAAC

GGCCGCGGTACCCTAACCGTGCAAAGGTAGCGCAATCACTTGTTCTTTAAATGAGGACTA

GTATGAACGGCATCACGAGGGTTATGCTGTCTCCCCACTCTATTCAGTGAAACTGATCTT

CCCGTGAAGAAGCGGGAATAAAAATATAAGACGAGAAGACCCCATGGAGCTTTAAACTCA

GCAGCAACTGCCTAAAT--AAAAACCTCACATTCT--GCAG-CCGTGCT-TGCTAGTTTT

CGGTTGGGGTGACCACGGAGTAAAACTAAACCTCCATGATGAAAGGAATTAATATCCTAA

CCCACGAGCTACAGCTCTAAGTATTAAC--------AAATTAACTAAA-TTGACCCAATT

ACTT--GATCAACGAACCAAGTTACCCTGGGGATAACAGCGCAATCCATTTCAAGAGCTC

ATATCGACAAATGGGTTTACG-ACC-TTGATGTTGGATCAGGGTATCCCAGTG-GCGCAG

CCGCTA-ATAA-TGGTT-

>M. berdmorei MN534706, MN534498, MN534599

TTATCATCAGCTTACCCCTAACTTACACATGCAAGTATCAGCACGCCCGTGAGAACGCCC

TTAAACCTT-ATTAGGACAAGGAGCCGGTATCAGGCACAG---ACTA-----TCTAGCCC

ATGACACCTAGCTTTGCCACTCCCTCAAGGGTATTCAGCAGTGATTAACATTGTTTATAA

GCGCCAGCTTGAATCAGTTAAGGGGCAC--AGAGCCGGCCAATACGGTGCCAGCCGCCGC

GGCTACACCGCTAGGCTCAAGTTGATATT-ATGCGGCGTTAAGCGTGTTTAAAGTGCTA-

AAGA-GATTAAAATTAAAATATAACTAAGCTGGTACCCGCTTGGTAATAAGAAAAACAAA

TACCAAAGTTATTCCAACCCATCCCCTTGAACCCCCGACAGCTAAAAAACCAACTGGGAT

TAAGTACCCCACTATGCCTAACCGTAAAATATTTATTTACACCT-TTAACCGCCCGGGAA

TTACGAGCCCAAGCTTAAAACCCAAAGGACTTGACGGTGTCCCACCCACCTAGAGGAGCC

TGTTCTATAA-TCGATTCTCCCC-GATATACCTCACCGTTTCTAGCTTT--TCAGCCTGT

ATACCTCCGTCGTAAGCTTACCCTATGAACGCT-TTTTAGTGAGCTAAAAGAT-CTTCAC

ATAAATACGTCAGGTCAAGGTGCAGCCTACGAAACGGCAAGAAATGGGCTACAATTTCTA

CAA---TAGAACACAACGAAAGACTACATGCAA-CTTAGTCATGAAGGCGGATTTAGTAG

TAAAAAGAAAATAGAGAGTTCTTTTAAATTAGGCGCTGGGACGCGTACACACCGCCCGTC

ACCCTCTTCAAACGCTTAC-AACCAG-TCTATAACAAA-TTTTTGCACTACAGAAGAGGT

AAGTCGTAACATGGTAAGCGTACTGGAAAGTGCGCTTGGAATAA-CAAAATGTAGCTTAA

CTAAA-GCATCTCGCTTACACCGAGGACATATCTGTGAAACTCTGATCGTTTTGA--GCT

AAAAATCTAGCTCTT--CTCATTCT-TATGAATACTC-CACCTCTT-AACCAA---CTGA

TAAATCATTTTCTAC-ATAAAGTAAAGGCGATAGAAAAATGTTTA-GAAGCTATAGAAAG

TAGTACCGCAAGGGAAAAATGAAATAATAATGAAAAA-TTTACAAGCACCAAAAAGCAGA

GCTGTAGCCTCGTACCTTTTGCATCATGGTCTAACTAGTCTAATCAAGCAAAGTGCATTT

TTAGTTTGACCCCCCGAAACTAGGTGAGCTACTTAAAAACAGCCGTT-AGGGCCAACCCG

TCTCTGTTGCAAAAGAGTGGGAAGATTTT-TAAGTAGGGGTGACAGACCTATCGAACCTA

GAGATAGCTGGTTATTTAGGAAAAGGATTTAAGTCCTACCTTAAAATTTTT---------

--------------------TGTACTAAATAATTTTATAACTATATAAAAACGCATATGT

TAGAACTAGTAACAAGAAGA-AGTACTTCTCCA-AAATGTAAGCTTAAGCCAAAATGAAC

CCCTCACTGGCAATTAAAGTCAATAAACCCCTAGTAGTAACTCA----TCAAGAAAACCC

TAC------------------TACCACA-AACGTTAACCTTACACTAGCACATTACTGGA

AAGAAATAAAGAGGGGGAAGGAACTCGGCAAATAATTTAACCCCGCCTGTTTACCAAAAA

CATCGCCTCTTGCTATCT----ATAAGAGGTCCAGCCTGCCCAGTGACTAA--GTTAAAC

GGCCGCGGTACCCTAACCGTGCAAAGGTAGCGCAATCACTTGTTCTTTAAATGAGGACTA

GTATGAACGGCATCACGAGGGTTATACTGTCTCCCCCCTTTTTTCAGTGAAACTGATCTT

CCCGTGAAGAAGCGGGAATATTGTTATAAGACGAGAAGACCCCATGGAGCTTTAAACTCA

GAATCAACTGCCACAAA--CTTAGCCTAAAAATAAC-GCAG-AAATGAC-TTCTAGTTTT

CGGTTGGGGTGACCGCGGAGTAAAATAAAACCTCCACGACGAAAGGAACTAACCTCCTAA

CCTAAGAGCTACAGCTCTAAGTATTAAA--------ACATTAACCTAA-TTGATCCAATT

ACTT--GATCAACGAACCAAGTTACCCTGGGGATAACAGCGCAATCCATTTCAAGAGCTC

ATATCGACAAATGGGTTTACG-ACC-TCGATGTTGGATCAGGATATCCAAGTG-GCGCAG

CCGCTA-CTAA-CGGTTC

>M. berdmorei MN534707, MN534499, MN534600

TTATCATCAGCTTACCCCTAACTTACACATGCAAGTATCAGCACACCCGTGAGAACGCCC

TTAAACCTT-ATTAGGACAAGGAGCCGGTATCAGGCACAG---ACTA-----TCTAGCCC

ATGACACCTAGCTTTGCCACTCCCTCAAGGGTATTCAGCAGTGATTAACATTGTTTATAA

GCGCCAGCTTGAATCAGTTAAGGGGCAC--AGAGCCGGCCAATACGGTGCCAGCCGCCGC

GGCTACACCGCTAGGCTCAAGTTGATATC-ATGCGGCGTTAAGCGTGTTTAAAGTGCTA-

AAAA-GATTAGAATTAAAATATAACTAAGCTGTGACACGCTTGTTAGTAAGAAAAGCAGA

TACGAAAGTTATTCTAACCCACCCACTTGAACCCACGACAGCTAAGACACAAACTGGGAT

TAGGTACCCCACTATGCCTAGCCGTAAAATATTTATTTACACCT-TTAACCGCCCGGGAA
TTACGAGCCCAAGCTTAAAACCCAAAGGACTTGACGGTGTCCCACCCACCTAGAGGAGCC
TGTTCTATAA-TCGATTCTCCCC-GATATACCTCACCG----------------------

------------------------------AGGCGCTGGGACGCGTACACACCGCCCGTC
ACCCTCTTCAAACGCTTAC-AACCAG-TTTATAACAAA--TTTTGCATTACAGAAGAGGT
AAGTTGTAACATGGTAAGCGTACTGGAAAGTGCGCTTGGAATAA-CAAAATGTAGTTTAA
CTAAA-GCATCTCGCTTACACCGAGAATATGTTTGTGAGACCCTGATCGTTTTGA--GCT
AAAAATCTAGCTTTT--CTCATTCC-TATGAATACCC-CACCTCCT-AACCAA---TTAA
TAAATCATTTTTTAC-ATAAAGTAAAGGCGATAGAAAAATGCCTA-GAAGCTATAGAAAG
TAGTACCGCAAGGGAAAGATGAAATAATAATGAAAAA-TTTACAAGCACCAAAAAGCAGA
GCTGTAACCTTGTACCTTTTGCATCATGGTCTAACTAGTCTAATCAAGCAAAATGCATTT
TTAGTTTGACCCCCCGAAACTAGGTGAGCTACTTAAAAACAGCCGTT-AGGGCCAACCCG
TTTTTGTTGCAAAAGAGTGGGAAGATTTT-TAAGTAGGGGTGACAGACCTATCGAACCTA
GAGATAGCTGGTTATTCAGGAAAAGGATTTAAGTCCTACCTTAAAATTTTT-ATACC-CC
CCCAAGTATTCTCAAAATTTTAAGAGCTATTCAAATAAGGTACAGCCTATTTGAAACAGG
ATACAACCTCCATCAA--

---TAAGCCAGAAGGAAC
CCTTCACTGGCAATTAAAGTCAATAAACCCTTAGTAGTAACCAA----TCAAGAAAACCC
TAC------------------TACTACA-AACGTTAACCTTACACCAGCACATTTCCGGA
AAGAAATAAAGAGGAGGAAGGAACTCGGCAAATAATCTAACCCCGCCTGTTTACCAAAAA
CATCGCCTCTTGCCATCT----ATAAGAGGTCCAGCCTGCCCAGTGACAAA--GTTAAAC
GGCCGCGGTACCCTAACCGTGCAAAGGTAGCGCAATCACTTGTTCTTTAAATGAGGACTA
GTATGAACGGCATCACGAGGGTTATACTGTCTCCCCCCTTTTTTCAGTGAAACTGATCTT
CCCGTGAAGAAGCGGGAATATTAATATAAGACGAGAAGACCCCATGGAGCTTTAAACTCA
GAATCAACTGCCACAAA--ATTAACCTAATAATCAC-GCAG-AAGTGAC-TTCTAGTTTT

CGGTTGGGGTGACCGCGGAGTAAAATAAAACCTCCACGACGAAAGGAACTAACCTCCTAA

CCTAAGAGCCACAGCTCTAAGTATTAAA--------ACATTAACCTAA-TTGATCCAATT

ACTT--GATCAACGAACCAAGTTACCCTGGGGATAACAGCGCAATCCATTTCAAGAGCTC

ATATCGACAAATGGGTTTACG-ACC-TCGATGTTGGATCAGGATATCCAAGTG-GCGCAG

CCGCTA-CTAA-CGGTTC

>M. berdmorei MN534708, MN534500, MN534601

TTATCATCAGCTTACCCCTGACTTACACATGCAAGTATCAGCACACCCGTGAGAACGCCC

TTAAACCTT-ACTAGGACAAGGAGCCGGTATCAGGCACAG---ATTA-----TCTAGCCC

ATGACACCTAGCTTTGCCACTCCCTCAAGGGTACTCAGCAGTGATTAACATTGTTCATAA

GCGCCAGCTTGACTCAGTCAAGGGGTTT--AGAGCCGGCCAATACGGTGCCAGCCGCCGC

GGCTACACCGCTAGGCTCAAGTTGATTTT-ATGCGGCGTTAAGCGTGTTTAAAGTGCCA-

AAAA-GACTAGAATTAAAATATAACTAAGCTGTGACACGCTTGTTAATAAGAAAAACAGA

TACGAAAGTTATTCTAACCAACCCACTTGAACCCACGACAGCTAAGACACAAACTGGGAT

TAGGTACCCCACTATGCCTAGCCGTAAAATATTTATTTACACCT-TTAACCGCCCGGGAA

TTACGAGCCCAAGCTTAAAACCCAAAGGACTTGACGGTGTCCCACCCACCTAGAGGAGCC

TGTTCTATAA-TCGATTCTCCCC-GATATACCTCACCG----------------------

--------------AACGAAAGACTACATGCAA-CTTAGTCATGAAGGCGGATTTAGTAG

TAAAAAGAAAATAGAGAGTTCTTTTTAATTAGGCGCTGGGACGCGTACACACCGCCCGTC

ACCCTCATCAAACGCTTAC-AACCAG-TTTATAACAAA-TTTCTGCACCACAGAAGAGGT

AAGTCGTAACATGGTAAGCGTACTGGAAAGTGCGCTTGGAATAA-CAAAATGTAGCTTAA

CTAAA-GCATCTCGCTTACACCGAGAATATATCTGTGAAACTCTGATCGTTTTGAG-GCT

AAAAATCTAGCTTTT--CTTATTTT-TATGAATACCC-CACCCCTT-AACCTA----AAT

TAAATCATTTTTTAC-ATAAAGTAAAGGCGATAGAAAAATGCCTA-GAAGCTATAGAAAG

TAGT-CCGCAAGGGAAAGATGAAATAATAATGAAAAA--CTGCAAGCACCAAAAAGCAGA

GCTGCAACCTCGTACCTTTTGCATCATGGTCTAACTAGTCCAACCAAGCAAAATGCATTT

TTAGTTTGACCCCCCGAAACTAGGTGAGCTACTTAAAAACAGCCATT-AGGGCCAACCCG

TCTCTGTTGCAAAAGAGTGGGAAGATTTT-TAAGTAGGGGTGACAGACCTATCGAACCTA

GAGATAGCTGGTTATTTAGGAAAAGGATCTAAGTCCTACCTTAAAATTTT--GTACT-CC

ATCAAGTATTTCCG-AATTTTAAGAGCTATTCAAATAAGGTACAGCCTGTT-GAAATAGG
ATACAACCTCCATCAA--
---------AAAAAAGCGTTAAAGCTTCATTG----TCTTATTAAAAAATTCCACTA-AC
TACATAAAACCCTTTAACCCTGTACTAAATAATTTTATAACTCTATAAAAATCCATATGT
TGGAACTAGTAACAAGAAGA-AACTCTTCTCCA-AAATGTAAGCATAAGCCAGAATGAAC
CCCTCACTGGCAATTAAAGTCAATAAACCTCTAGTAGTAACCCA----TCAAGAAAACCC
TTC------------------TATTATG-AACGTTAACCTTACACTAGCACATTTCTGGA
AAGAAAGAAAGAGGGGGAAGGAACTCGGCAAATAATTTAACCCCGCCTGTTTACCAAAAA
CATCGCCTCTTGCCAACT----ATAAGAGGTCCAGCCTGCCCAGTGACAAA--GTTTAAC
GGCCGCGGTACCCTAACCGTGCAAAGGTAGCGCAATCACTTGTTCTTTAAATGAGGACTA
GTATGAACGGCATCACGAGGGTTATACTGTCTCCCCCCTTTTTTCAGTGAAACTGATCTT
CCCGTGAAGAAGCGGGAATATAAATATAAGACGAGAAGACCCCATGGAGCTTTAAACTCA
GAATCAACTGCTACAAA--CTTAGCCTAATAACTAC-GCAG-GAATGAC-TTCTAGTTTT
AGGTTGGGGTGACCGCGGAGTAAAATAAAACCTCCACGACGAAAGGAACTAACCGCCTAA
ACCAAGAGCCACAGCTCTAAGTATTAAA--------ACATTAACCCAA-TTGATCCAATT
ACTT--GATCAACGAACCAAGTTACCCTGGGGATAACAGCGCAATCCATTTCAAGAGCTC
ATATCGACAAATGGGTTTACGTACC-TCGATGTTGGATCAGGATATCCAAGTG-GCGCAG
CCGCTA-CTAA-CGGTTC
>M. berdmorei MN534709, MN534501, MN534602
TTATCATCAGCTTACCCCTGACTTACACATGCAAGTATCAGCACACCCGTGAGAACGCCC
TTAAACCTT-ACTAGGACAAGGAGCCGGTATCAGGCACAG---ATTA-----TCTAGCCC
ATGACACCTAGCTTTGCCACTCCCTCAAGGGTACTCAGCAGTGATTAACATTGTTCATAA
GCGCCAGCTTGACTCAGTCAAGGGGTTT--AGAGCCGGCCAATACGGTGCCAGCCGCCGC
GGCTACACCGCTAGGCTCAAGTTGATTTT-ATGCGGCGTTAAGCGTGTTTAAAGTGCCA-
AAAA-GACTAGAATTAAAATATAACTAAGCTGTGACACGCTTGTTAATAAGAAAAACAGA
TACGAAAGTTATTCTAACCAACCCACTTGAACCCACGACAGCTAAGACACAAACTGGGAT
TAGGTACCCCACTATGCCTAGCCGTAAAATATTTATTTACACCT-TTAACCGCCCGGGAA
TTACGAGCCCAAGCTTAAAACCCAAAGGACTTGACGGTGTCCCACCCACCTAGAGGAGCC
TGTTCTATAA-TCGATTCTCCCC-GATATACCTCACCG----------------------

-AA---TAGAACATAACGAAAGACTATATGCAA-CTTAGTCATGAAGGCGGATTTAGTAG

TAAAAAGAAAATAGAGAGTTCTTTTTAATTAGGCGCTGGGACGCGTACACACCGCCCGTC

ACCCTCTTCAAACGCTTAC-AACCAG-TGTATAACAAA-TTTCTGCACCACAGAAGAGGT

AAGTCGTAACATGGTAAGCGTACTGGAAAGTGCGCTTGGAATAA-CAAAATGTAGCTTAA

CTAAA-GCATCTCGCTTACACCGAGAATATATCTGTGAAACTCTGATCGTTTTGA--GCT

GAAAATCTAGCTCTT--CTTATTCT-TATGAATACCC-CACCCCTT-AACCTA---TAAA

TAAATCATTTTTTAC-ATAAAGTAAAGGCGATAGAAAAATGCCTA-GAAGCTATAGAAAG

TAGTACCGCAAGGGAAAGATGAAATAATAATGAAAAA--CTGCAAGCACCAAAAAGCAGA

GCTGCAACCTCGTACCTTTTGCATCATGGTCTAACTAGTCCAACCAAGCAAAATGCATTT

TTAGTTTGACCCCCCGAAACTAGGTGAGCTACTTAAAAACAGCCATT-AGGGCCAACCCG

TCTCTGTTGCAAAAGAGTGGGAAGATTTT-TGAGTAGGGGTGACAGACCTATCGAACCTA

GAGATAGCTGGTTATTTAGGAAAAGGATCTAAGTCCTACCTTAAAATTTTT-GTACT-CC

ATCAAGTATTTCCG-AATTTTAAGAGCTATTCAAATAAGGTACAGCCTGTTTGAAATAGG

ATACAACCTCCATCAATGG-------------------------------------CAG

CCACCTTTT-AAAAAGCGTTAAAGCTTCATTG----TCTTATTAAAAAATTCCACTA-AC

TACATAAAACCCTT-AACCCTGTACTAAATAATTTTATAACTCTATAAAAATCCATATGT

TGGAACTAGTAACAAGAAGA-AACCCTTCTCCA-AAATGTAAGCATAAGCCAGAATGAAC

CCCTCACTGGCAATTAAAGTCAATAAACCTCTAGTAGTAACCCA----TCAAGAAAACCC

TTC------------------TATTATG-AACGTTAACCTTACACTAGCACATTTCTGGA

AAGAAAGAAAGAGGGGGAAGGAACTCGGCAAATAATTTAACCCCGCCTGTTTACCAAAAA

CATCGCCTCTTGCCAACT----ATAAGAGGTCCAGCCTGCCCAGTGACAAA--GTTTAAC

GGCCGCGGTACCCTAACCGTGCAAAGGTAGCGCAATCACTTGTTCTTTAAATGAGGACTA

GTATGAACGGCATCACGAGGGTTATACTGTCTCCCCCCTTTTTTCAGTGAAACTGATCTT

CCCGTGAAGAAGCGGGAATATAAATATAAGACGAGAAGACCCCATGGAGCTTTAAACTCA

GAATCAACTGCTACAAA--CTTAGCCTAATAACTAC-GCAG-GAATGAC-TTCTAGTTTT

AGGTTGGGGTGACCGCGGAGTAAAATAAAACCTCCACGACGAAAGGAACTAACCGCCTAA

ACCAAGAGCCACAGCTCTAAGTATTAAA--------ACATTAACCTAA-TTGATCCAATT

ACTT--GATCAACGAACCAAGTTACCCTGGGGATAACAGCGCAATCCATTTCAAGAGCTC

ATATCGACAAATGGGTTTACG-ACC-TCGATGTTGGATCAGGATATCCAAGTG-GCGCAG

CCGCTA-CTAA-CGGTTC

>M. berdmorei MN534710, MN534502, MN534603

TTATCATCAGCTTACCCCTAACTTACACATGCAAGTATCAGCACACCCGTGAGAACGCCC

TTAAACCTT-ACTAGGACAAGGAGCCGGTATCAGGCACAG---ATTA-----TCTAGCCC

ATGACACCTAGCTTTGCCACTCCCTCAAGGGTACTCAGCAGTGATTAACATTGTTCATAA

GCGCCAGCTTGACTCAGTTAAGGGGTAT--AGAGCCGGCCAATACGGTGCCAGCCGCCGC

GGCTACACCGCTAGGCTCAAGTTGATTTT-ATGCGGCGTTAAGCGTGTTTAAAGTGCTA-

AAAA-GATTAGAATTAAAATATAGCCAAGCTGTGACACGCTTGTTAGTAAGAAAAACAGA

TACGAAAGTTATTCTAACCAACCCACTTGAACCCACGACAGCTAAGACACAAACTGGGAT

TAGGTACCCCACTATGCCTAGCCGTAAAATATTTATTTACACCT-TTAACCGCCCGGGAA

TTACGAGCCCAAGCTTAAAACCCAAAGGACTTGACGGTGTCCCACCCACCTAGAGGAGCC

TGTTCTATAA-TCGATTCTCCCC-GATATACCTCACCGCTTCTAGCTTT--TCAGCCTGT

ATACCTCCGTCGTAAGCTTACCCTATGAACGCT-TTTTAGTGAGCTAAAAGAT-CTTTAC

ATAAATACGTCAGGTCAAGGTGCAGCCTACGAAGCGGCAAGAAATGGGCTACAATTTCTA

CAA---TAGAACACAACGAAAGACTACATGCAA-CTTAGTCATGAAGGCGGATTTAGTAG

TAAAAAGAAAATAGAGAGTTCTTTTTAACCAGGCGCTGGGACGCGTACACACCGCCCGTC

ACCCTCTTCAAACGCTTAA-AACCAG-TTTATAACAAA-TTTCTGCACCACAGAAGAGGT

AAGTCGTAACATGGTAAGCGTACTGGAAAGTGCGCTTGGAATAA-CAAAATGTAGCTTAA

CTAAA-GCATCTCGCTTACACCGAGAATATGTCTGTGAAACCCTGATCGTTTTGA--GCT

AAAAATCTAGCTCTT--CTTATTCT-TATGAATACCC-CACCTTTT-AACCAA---TAAA

TAAATCATTTTTTAC-ATAAAGTAAAGGCGATAGAAAAATGCCTA-GAAGCTATAGAAAA

CAGTACCGCAAGGGAAAGATGAAATATTAATGAAAAA--TTGCAAGCACCAAAAAGCAGA

GCTGTAACCTCGTACCTTTTGCATCATGGTCTAACTAGTCTAACCAAGCAAAATGCATTT

TTAGTTTGACCCCCCGAAACTAGGTGAGCTACTTAAAAACAGCCATT-AGGGCCAACCCG

TCTCTGTTGCAAAAGAGTGGGAAGATTTTTTAAGTAGGGGTGACAGACCTATCGAACCTA

GAGATAGCTGGTTATTTAGGAAAAGGATTTAAGTCCTACCTTAAAATTTTT-ATACC-CC

ATCAAGTATTTTCC--

---TTCCATTA-AC

TACATAAAACCCTT-AACTCTGTACTAAATAATTTTATAACTGTATAAAAGCCCGTATGT

TGGAACTAGTAACAAGAAGA-AACCCTTCTCCA-AAATGTAAGCATAAGCCAGAATGAAC

CCCTCACTGGCAATTAAAGTCAATAAAACCCTAGTAGTAACTCA----TCAAGAAAACCC

TTC------------------TATTGTA-AACGTTAACCTTACACCAGCACATTTCTGGA

AAGAAAAAAAGAGGGGGAAGGAACTCGGCAAATA-TTTAACCCCGCCTGTTTACCAAAAA

CATCGCCTCTTGCTAACT----ATAAGAGGTCCAGCCTGCCCAGTGACAAA--GTTAAAC

GGCCGCGGTACCCTAACCGTGCAAAGGTAGCGCAATCACTTGTTCTTTAAATGAGGACTA

GTATGAACGGCATCACGAGGGTTATACTGTCTCCCCCCTTTTTTCAGTGAAACTGATCTT

CCCGTGAAGAAGCGGGAATATTAATATAAGACGAGAAGACCCCATGGAGCTTTAAACTCA

GAATCAACTGCCACAAA--CTTAACCTAATAACTAT-GCAG-GAGTGAC-TTCTAGTTTT

CGGTTGGGGTGACCGCGGAGTAAAATAAAACCTCCACGACGAAAGGAATTAACCACCTAA

ACCAAGAGCCACGGCTCTAAGTATTAAA--------ACATTAACCTAA-TTGATCCAATT

ACTT--GATCAACGAACCAAGTTACCCTGGGGATAACAGCGCAATCCATTTCAAGAGCTC

ATATCGACAAATGGGTTTACG-ACC-TCGATGTTGGATCAGGATATCCAAGTG-GCGCAG

CCGCTA-CTAA-CGGTTC

>M. berdmorei MN534711, MN534503, MN534604

TTATCATCAGCTTACCCCTAACTTACACATGCAAGTATCAGCACACCCGTGAGAACGCCC

TTAAACCTT-ACTAGGACAAGGAGCCGGTATCAGGCACAG---ATTA-----TCTAGCCC

ATGACACCTAGCTTTGCCACTCCCTCAAGGGTATTCAGCAGTGATTAACATTGTTCATAA

GCGCCAGCTTGACTCAGTTAAGGAGTAT--AGAGCCGGCCAATACGGTGCCAGCCGCCGC

GGCTACACCGCTAGGCTCAAGTTGATTTT-ATGCGGCGTTAAGCGTGTTTAAAGTGCTA-

AAAA-GATTAGAATTAAAATATAGCCAAGCTGTGACACGCTTGTTAGTAAGAAAAACAGA

TACGAAAGTTATTCTAACCAACCCACTTGAACCCACGACAGCTAAGACACAAACTGGGAT

TAGGTACCCCACTATGCCTAGCCGTAAAATATCTACTTACACCT-TTAACCGCCTGGGAA

TTACGAGCCCAAGCTTAAAACCCAAAGGACTTGACGGTGTCCCACCCACCTAGAGGAGCC

TGTTCTATAA-TCGATTCTCCCC-GATATACCTCACCGCTTCTAGCTTT-TTCAGCCTGT

ATACCTCCGTCGTAAGCTTACCCTATGAACGCT-TTTTAGTGAGCTAAAAGAT-CTTTAC

ATAAATACGTCAGGTCAAGGTGCAGCCTACGAAGCGGCAAGAAATGGGCTACAATTTCTA

CAA---TAGAACACAACGAAAGACTACATGCAA-CTTAGTCATGAAGGCGGATTTAGTAG

TAAAAAGAAAATAGAGAGTTCTTTTTAATCAGGCGCTGGGACGCGTACACACCGCCCGTC

ACCCTCTTCAAACGCTTAA-AACCAG-TTTATAACAAA-TTTCTGCACCACAGAAGAGGT

AAGTCGTAACATGGTAAGCGTACTGGAAAGTGCGCTTGGAATAA-CAAAATGTAGCTTAA
CTAAA-GCATCTCGCTTACACCGAGAACATGTCTGTGAAACCCTGATCGTTTTGA--GCT
AAAAATCTAGCTCTT--CTTATTCT-TATGAATACCC-CACCTTTT-AACCAA---TAAA
TAAATCATTTTTTAC-ATAAAGTAAAGGCGATAGAAAAATGCCTA-GAAGCTATAGAAAA
CAGTACCGCAAGGGAAAGATGAAATATTAATGAAAAA--TTGCAAGCACCAAAAAGCAGA
GCTGTAACCTCGTACCTTTTGCATCATGGTCTAACTAGTCTAACCAAGCAAAATGCATTT
TTAGTTTGACCCCCCGAAACTAGGTGAGCTACTTAAAAACAGCCATT-AGGGCCAACCCG
TCTCTGTTGCAAAAGAGTGGGAAGATTTT-TAAGTAGGGGTGACAGACCTATCGAACCTA
GAGATAGCTGGTTATTTAGGAAAAGGATTTAAGTCCTACCTTAAAATTTTT---------

--------AAAAAAAGCGTTAAAGCTTCATTG----TTTTATTAAAAAATTCCATTA-AC
TTCATAAAACCCTT-AACTCTGTACTAAATAATTTTATAACTGTATAAAAGCCCGTATGT
TAGAACTAGTAACAAGAAGA-AACCCTTCTCCA-AAATGTAAGCATAAGCCAGAATGAAC
CCCTCACTGGCAATTAAAGTCAATAAAACCCTAGCAGTAACTCA----TCAAGAAAACCC
TTC------------------TATTGTA-AACGTTAACCTTACACCAGTACATTTCTGGA
AAGAAAGAAAGAGGGGGAAGGAACTCGGCAAATAATTTAACCCCGCCTGTTTACCAAAAA
CATCGCCTCTTGCTAACT----ATAAGAGGTCCAGCCTGCCCAGTGACAAA--GTTAAAC
GGCCGCGGTACCCTAACCGTGCAAAGGTAGCGCAATCACTTGTTCTTTAAATGAGGACTA
GTATGAACGGCATCACGAGGGTTATACTGTCTCCCCCCTTTTTTCAGTGAAACTGATCTT
CCCGTGAAGAAGCGGGAATATTAATATAAGACGAGAAGACCCCATGGAGCTTTAAACTCA
GAATCAACTGCCACAAA--CTTAACCTAATAACCAT-GCAG-AAATGAC-TTCTAGTTTT
CGGTTGGGGTGACCGCGGAGTAAAATAAAACCTCCACGACGAAAGGAACTAACCACCTAA
ACCAAGAGCCACAGCTCTAAGTATTAAA--------ACATTAACCTAA-TTGATCCAATT
ACTT--GATCAACGAACCAAGTTACCCTGGGGATAACAGCGCAATCCATTTCAAGAGCTC
ATATCGACAAATGGGTTTACG-ACC-TCGATGTTGGATCAGGATATCCAAGTG-GCGCAG
CCGCTA-CTAA-CGGTTC
>M. berdmorei MN534712, MN534504, MN534605
TTATCATCAGCTTACCCCTAACTTACACATGCAAGTATCAGCACACCCGTGAGAACGCCC
TTAAACCTT-ACTAGGACAAGGAGCCGGTATCAGGCACAG---ATTA-----TCTAGCCC

ATGACACCTAGCTTTGCCACTCCCTCAAGGGTATTCAGCAGTGATTAACATTGTTCATAA

GCGCCAGCTTGACTCAGTTAAGGGGTAT--AGAGCCGGCCAATACGGTGCCAGCCGCCGC

GGCTACACCGCTAGGCTCAAGTTGATTTT-ATGCGGCGTTAAGCGTGTTTAAAGTGCTA-

AAAA-GATTAGAATTAAAATATAGCCAAGCTGTGACACGCTTGTTAGTAAGAAAAACAGA

TACGAAAGTTATTCTAACCAACCCACTTGAACCCACGACAGCTAAGACACAAACTGGGAT

TAGGTACCCCACTATGCCTAGCCGTAAAATATTTATTTACACCT-TTAACCGCCCGGGAA

TTACGAGCCCAAGCTTAAAACCCAAAGGACTTGACGGTGTCCCACCCACCTAGAGGAGCC

TGTTCTATAA-TCGATTCTCCCC-GATATACCTCACCGCTTCTAGCTTT-TTCAGCCTGT

ATACCTCCGTCGTAAGCTTACCCTATGAACGCT-TTTTAGTGAGCTAAAAGAT-CTTTAC

ATAAATACGTCAGGTCAAGGTGCAGCCTACGAAGCGGCAAGAAATGGGCTACAATTTCTA

CAA---TAGAACACAACGAAAGACTACATGCAA-CTTAGTCATGAAGGCGGATTTAGTAG

TAAAAAGAAAATAGAGAGTTCTTTTTAATCAGGCGCTGGGACGCGTACACACCGCCCGTC

ACCCTCTTCAAACGCTTAA-ACCCAG-TTTATAACAAA-TTTCTGCACCACAGAAGAGGT

AAGTCGTAACATGGTAAGCGTACTGGAAAGTGCGCTTGGAATAA-CAAAATGTAGCTTAA

CTAAA-GCATCTCGCTTACACCGAGAACATGTCTGTGAAACCCTGATCGTTTTGA--GCT

AAAAATCTAGCTCTT--CTTATTCT-TATGAATACCCCCACCTTTT-AACCAA---TAAA

TAAATCATTTTTTAC-ATAGAGTAAAGGCGATAGAAAAATGCCTA-GAAGCTATAGAAAA

CAGTACCGCAAGGGAAAGATGAAATATTAATGAAAAA--TTGCAAGCACCAAAAAGCAGA

GCTGCAACCTCGTACCTTTTGCATCATGGTCTAACTAGTCTAACCAAGCAAAATGCATTT

TTAGTTTGACCCCCCGAAACTAGGTGAGCTACTTAAAAACAGCCATT-AGGGCCAACCCG

TCTCTGTTGCAAAAGAGTGGGAAGATTTT-TAAGTAGGGGTGACAGACCTATCGAACCTA

GAGATAGCTGGTTATTTAGGAAAAGGATTTAAGTCCTACCTTAAAATTTTT-ATACC-CC

ATC---

------TT-AAAAAAGCGTTAAAGCTTCATTG-----TTTATTAAAAAATTCCATTA-AC

TACATAAAACCCTT-AACTCTGTACTAAATAATTTTATAACTATATAAAAGCCCGTATGT

TGGAACTAGTAACAAGAAGA-AATCCTTCTCCA-AAATGTAAGCATAAGCCAGAATGAAC

CCCTCACTGGCAATTAAAGTCAATAAAACCCTAGAAGTAACTTA----TCAAGAAAACCC

TTC------------------TACTGTA-AACGTTAACCTTACACCAGCACATTTCTGGA

AAGAAAAAAAGAGGGGGAAGGAACTCGGCAAATAATTTAACCCCGCCTGTTTACCAAAAA

CATCGCCTCTTGCTAACT----ATAAGAGGTCCAGCCTGCCCAGTGACAAA--GTTAAAC

GGCCGCGGTACCCTAACCGTGCAAAGGTAGCGCAATCACTTGTTCTTTAAATGAGGACTA

GTATGAACGGCATCACGAGGGTTATACTGTCTCCCCCCTTTTTTCAGTGAAACTGATCTT

CCCGTGAAGAAGCGGGAATATTAATATAAGACGAGAAGACCCCATGGAGCTTTAAACTCA

GAATCAACTGCCACAAA--CTTAACCTAATAACTAT-GCAG-GAATGAC-TTCTAGTTTT

CGGTTGGGGTGACCGCGGAGTAAAATAAAACCTCCACGACGAAAGGAACTAACCACCTAA

ACCAAGAGCCACAGCTCTAAGTATTAAA--------ACATTAACCTAA-TTGATCCAATT

ACTT--GATCAACGAACCAAGTTACCCTGGGGATAACAGCGCAATCCATTTCAAGAGCTC

ATATCGACAAATGGGTTTACG-ACC-TCGATGTTGGATCAGGATATCCTAGTG-GCGCAG

CCGCTA-CTAA-CGGTTC

>M. berdmorei MN534713, MN534606

TTATCATCAGCTTACCCCTAACTTACACATGCAAGTATCAGCACACCCGTGAGAACGCCC

TTAAACCTT-ACTAGGACAAGGAGCCGGTATCAGGCACAG---ATTA-----TCTAGCCC

ATGACACCTAGCTTTGCCACTCCCTCAAGGGTATTCAGCAGTGATTAACATTGTTCATAA

GCGCCAGCTTGACTCAGTTAAGGGGTAT--AGAGCCGGCCAATACGGTGCCAGCCGCCGC

GGCTACACCGCTAGGCTCAAGTTGATTTT-ATGCGGCGTTAAGCGTGTTTAAAGTGCTA-

AAAA-GATTAGAATTAAAATATAACCAAGCTGTGACACGCTTGTTAGTAAGAAAAACAAA

TACGAAAGTTATTCTAACCAACCCACTTGAACCCACGACAGCTAAGACACAAACTGGGAT

TAGGTACCCCACTATGCCTAGCCGTAAAATATTTATTTACACCT-TTAACCGCCCGGGAA

TTACGAGCCCAAGCTTAAAACCCAAAGGACTTGACGGTGTCCCACCCACCTAGAGGAGCC

TGTTCTATAA-TCGATTCTCCCC-GATATACCTCACCGCTTCTAGCTTT-TTCAGCCTGT

ATACCTCCGTCGTAAGCTTACCCTATGAACGCT-TTTTAGTGAGCTAAAAGAT-CTTTAC

ATAAATACGTCAGGTCAAGGTGCAGCCTACGAAGCGGCAAGAAATGGGCTACAATTTCTA

CAA---TAGAACACAACGAAAGACTACATGCAA-CTTAGTCATGAAGGCGGATTTAGTAG

TAAAAAGAAAATAGAGAGTTCTTTTTAATCAGGCGCTGGGACGCGTACACACCGCCCGTC

ACCCTCTTCAAACGCTTAA-ACCTAG-TTTATAACAAA-TTTCTGCACCACAGAAGAGGT

AAGTCGTAACATGGTAAGCGTACTGGAAAGTGCGCTTGGAATAA-CAAAATGTAGCTTAA

CTAAA-GCATCTCGCTTACACCGAGAACATGTCTGTGAAACCCTGATCGTTTTGA--GCT

AAAAATCTAGCTCTT--CTTATTCT-TATGAATACCC-CACCTTTT-AACCAA---TAAA

TAAATCATTTTTTAC-ATAAAGTAAAGGCGA----------------------------

---AGCAG
CCACCTTT-AAAAAAGCGTTAAAGCTTCATTG-----TTTATTAAAAAATTCCATTA-AC
TACATAAAACCCTT-AACTCTGTACTAAATAATTTTATAACTATATAAAAGCCCGTATGT
TGGAACTAGTAACAAGAAGA-AATCCTTCTCCA-AAATGTAAGCATAAGCCAGAATGAAC
CCCTCACTGGCAATTAAAGTCAATAAAACCCTAGAAGTAACTTA----TCAAGAAAACCC
TTC------------------TACTGTA-AACGTTAACCTTACACCAGCACATTTCTGGA
AAGAAAAAAAGAGGGGGAAGGAACTCGGCAAATAATTTAACCCCGCCTGTTTACCAAAAA
CATCGCCTCTTGCTAACT----ATAAGAGGTCCAGCCTGCCCAGTGACAAA--GTTAAAC
GGCCGCGGTACCCTAACCGTGCAAAGGTAGCGCAATCACTTGTTCTTTAAATGAGGACTA
GTATGAACGGCATCACGAGGGTTATACTGTCTCCCCCCTTTTTTCAGTGAAACTGATCTT
CCCGTGAAGAAGCGGGAATATTAATATAAGACGAGAAGACCCCATGGAGCTTTAAACTCA
GGATCAACTGCCACAAA--CTTAACCTAATAACCAT-GCAG-GAATGAC-TTCTAGTTTT
CGGTTGGGGTGACCGCGGAGTAAAATAAAACCTCCACGACGAAAGGAACTAACCGCCTAA
ACCAAGAGCCACAGCTCTAAGTATTAAA--------ACATTAACCTAA-TTGATCCAATT
ACTT--GATCAACGAACCAAGTTACCCTGGGGATAACAGCGCAATCCATTTCAAGAGCTC
ATATCGACAAATGGGTTTACG-ACC-TCGATGTTGGATCAGGATATCCAAGTG-GCGCAG
CCGCTA-CTAA-CGGTTC
>M. berdmorei MN534714, MN534505, MN534607
TTATCATCAGCTTACCCCTAACTTACACATGCAAGTATCAGCACACCCGTGAGAACGCCC
TTAAACCTT-ACTAGGACAAGGAGCCGGTATCAGGCACAG---ATTA-----TCTAGCCC
ATGACACTTAGCTTTGCCACTCCCTCAAGGGTATTCAGCAGTGATTAACATTGTTCATAA
GCGCCAGCTTGACTCAGTTAAGGGGTAT--AGAGCCGGCCAATACGGTGCCAGCCGCCGC
GGCTACACCGCTAGGCTCAAGTTGATTTT-ATGCGGCGTTAAGCGTGTTTAAAGTGCTA-
AAAA-GATTAGAATTAAAATATAGCCAAGCTGTGACACGCTTGTTAGTAAGAAAAACAGA

```
TACGAAAGTTATTCTAACCAACCCACTTGAACCCACGACAGCTAAGACACAAACTGGGAT
TAGGTACCCCACTATGCCTAGCCGTAAAATATTTATTTACACCT-TTAACCGCCCGGGAA
TTACGAGCCCAAGCTTAAAACCCAAAGGACTTGACGGTGTCCCACCCACCTAGAGGAGCC
TGTTCTATAA-TCGATTCTCCCC-GATATACCTCACCGCTTCTAGCTTT--TCAGCCTGT
ATACCTCCGTCGTAAGCTTACCCTATGAACGCT-TTTTAGTGAGCTAAAAGAT-CTTTAC
ATAAATACGTCAGGTCAAGGTGCAGCCTACGAAGCGGCAAGAAATGGGCTACAATTTCTA
CAA---TAGAACACAACGAAAGACTACATGCAA-CTTAGTCATGAAGGCGGATTTAGTAG
TAAAAAGAAAATAGAGAGTTCTTTTTAACCAGGCGCTGGGACGCGTACACACCGCCCGTC
ACCCTCTTCAAACGCTTAA-AACCAG-TTTATAACAAA-TTTCTGCACCACAGAAGAGGT
AAGTCGTAACATGGTAAGCGTACTGGAAAGTGCGCTTGGAATAA-CAAAATGTAGCTTAA
CTAAA-GCATCTCGCTTACACCGAGAACATGTCTGTGAAACCCTGATCGTTTTGA--GCT
AAAAATCTAGCTCTT--CTTATTCT-TATGAATACCC-CACCTTTT-AACCAA---TAAA
TAAATCATTTTTTAC-ATAAAGTAAAGGCGATAGAAAAATGCCTA-GAAGCTATAGAAAA
CAGTACCGCAAGGGAAAGATGAAATATTAATGAAAAA--TTGCAAGCACCAAAAAGCAGA
GCTGTAACCTCGTACCTTTTGCATCATGGTCTAACTAGTCTAACCAAGCAAAATGCATTT
TTAGTTTGACCCCCCGAAACTAGGTGAGCTACTTAAAAACAGCCATT-AGGGCCAACCCG
TCTCTGTTGCAAAAGAGTGGGAAGATTTT-TAAGTAGGGGTGACAGACCTATCGAACCTA
GAGATAGCTGGTTATTTAGGAAAAGGATTTAAGTCCTACCTTAAAATTTTT-ATACC---
------------------------------------------------------------
------------------------------------------------------------
------TT-AAAAAAGCGTTAAAGCTTCATTG----TTTAATTAAAAAATTTCATTA-AC
TACATAAAACCCTT-AACTCTGTACTAAATAATTTTATAACTGTATAAAAGCCCATATGT
TGGAACTAGTAACAAGAAGA-AACCCTTCTCCA-AAATGTAAGCATAAGCCAGAATGAAC
CCCTCACTGGCAATTAAAGTCAATAAAACCCTAGTAGTAACTCA----TCAAGAAAACCC
TTC------------------TATTGTA-AACGTTAACCTTACACCAGCACATTTCTGGA
AAGAAAAAAAGAGGGGGAAGGAACTCGGCAAATAATTTAACCCCGCCTGTTTACCAAAAA
CATCGCCTCTTGCTAACT----ATAAGAGGTCCAGCCTGCCCAGTGACAAA--GTTAAAC
GGCCGCGGTACCCTAACCGTGCAAAGGTAGCGCAATCACTTGTTCTTTAAATGAGGACTA
GTATGAACGGCATCACGAGGGTTATACTGTCTCCCCCCTTTTTTCAGTGAAACTGATCTT
CCCGTGAAGAAGCGGGAATATTAATATAAGACGAGAAGACCCCATGGAGCTTTAAACTCA
```

GAATCAACTGCCACAAA--CTTAACCTAATAACTAT-GCAG-AAGTGAC-TTCTAGTTTT

CGGTTGGGGTGACCGCGGAGTAAAATAAAACCTCCACGACGAAAGGAATTAACCACCTAA

ACCAAGAGCCACGGCTCTAAGTATTAAA--------ACATTAACCTAA-TTGATCCAATT

ACTT--GATCAACGAACCAAGTTACCCTGGGGATAACAGCGCAATCCATTTCAAGAGCTC

ATATCGACAAATGGGTTTACG-ACC-TCGATGTTGGATCAGGATATCCAAGTG-GCGCAG

CCGCTA-CTAA-CGGTTC

>M. pulchra MN534715, MN534508, MN534608

TTATTATCAGCTTATCCTTAACTTACACATGCAAGTATCAGCACACCCGTGAGAACGCCC

TTAAACCTT-ACTAGGACAAGGAGCCGGTATCAGGCACAG---ACCCA----TCTAGCCC

ATTACACCTAGCTTTGCCACTCCCTCAAGGGTCTTCAGCAGTGATTAACATTGTTCATAA

GCGACAGCTTGACTCAGTTAAAGGACAC--AGGGCCGGCTAATACGGTGCCAGCCGCCGC

GGCTACACCGCTAGGCTCAAGTTGATATT-ATTCGGCGTTAAGCGTGTTTAAAGTGCTT-

TTCAAGATTAGAACTAAACCCTAACCAAGCTGTGACACGCTTGCTATCAGGAAAAACAAA

TACGAAAGTTATTCTAACCCACCCACTTGAACCCACGACAGCTAGGACACAAACTGGGAT

TAGGTACCCCACTATGCCTAGCCGTAAAATATTTATTTACACCT-TTAACCGCCAGGGGA

TTACGAGCCCAAGCTTAAAACCCAAAGGACTTGACGGTGTCCCACCCACCTAGAGGAGCC

TGTTCTATAA-TCGATTCTCCCCCGTTATACCTCACCACTTCTAGCTTC--TCAGCCTGT

ATACCTCCGTCGTAAGCTTACCATATGAACGCC-TTTTAGTGAGCAAAAAGAT--TTTAC

ATAAATACGTCAGGTCAAGGTGCAGCCTACGAAATGGCAAGAAATGGGCTACAATTTCTA

TGAGCATAGAACACAACGAAAGACTACATGCAA-CTTAGTCATGAAGGCGGATTTAGTAG

TAAAAAGAAAATAGAGAGTTCTTTTTAATCAGGCGCTGGGACGCGTACACACCGCCCGTC

ACCCTCTTCAAACGCTTAA-ACTCAG-TTTATAACGAA-CTCATGCACCACAGAAGAGGT

AAGTCGTAACATGGTAAGCGTACTGGAAAGTGCGCTTGGAATAAACAAAATGTAGCTTAA

CTAAA-GCATCTCGCTTACACCGAGAACATGTCCGTGAAACTCAGATCATTTTGA--GCC

AAAAATCTAGCTCCC--CTAAACCT-AATGAACCTCC-CA-CAATT-CAAATA--CCAAA

TAAAACATTTTGTAC-ATAAAGTATAGGCGATAAAAAAATGTCTA-GAAGCAATAGAG-T

TAGTACCGTAAGGGAATGATGAAATAATAATGAAAAA--CTGCAAGCACAAAAAAGTAGA

GCTACAACCTCGTACCTTTTGCATCATGGTCTAACGAGTCCAACCAAGCAAAATGCACTT

TAAGTTTGACCCCCCGAAACTAGGTGAGCTACTCAAAAACAGCCTTT-AGGGCCAACCCG

TCTCTGTTGCAAAAGAGTGGGAAGATTCT-TGAGTAGGGGTGACAGACCTATCGAACCTA

GAGATAGCTGGTTATTCAGAAAAAGGATTTAAGTCCTGCCTTAAGGTTTTCCGTATAATC

--GAGCACTTTTTT-AACCTTAAGAGTTATTCAAATAAGGTACAGCCTATTTGAATTAGG

ATACAACCTCTATCAA--

------TT-AAAAAAGCGTTAAAGCTTTATTA---TTTTATTTAAAAA-TTCCCCCA-AC

TTTCTATAACCCTT-AACTTTGTACTGAATAATTTTATATTAATATAAAAACTTTTATGT

TAGAACTAGTAACAAGAAGC-AGCCCTTCTCCA-AAATGTAAGTTTAAGCCAAAATGAAC

AACTCATTGGCAATTAAAGTCATTAAACCTCTAGTAGTAACCCCC---TCAAGAAAAATC

TAC------------------TATATTTCAACGTTAATCTTACACTAGAACATTTCTGGA

AAGATAAAAAGAATGGGAAGGAACTCGGCAAACAACTTAACCCCGCCTGTTTACCAAAAA

CATCGCCTCTTGATAACCC---ATAAGAGGTCCAGCCTGCCCAGTGACAAA--GTTAAAC

GGCCGCGGTACCCTAACCGTGCAAAGGTAGCGCAATCACTTGTTCTTTAAATGAGGACCG

GTATGAACGGCATCACGAGGGTTATACTGTCTCCCCACTCAACTCAGTGAAACTGATCTT

CCCGTGAAGAAGCGGGAATAAAAATATAAGACGAGAAGACCCCATGGAGCTTAAAACTCA

GAATCAACTGCCACATT--TACAACCTCATAACCAC-GCAG-CCATGAC-CTCTAGTTTT

CGGTTGGGGTGACCGCGGAGTAAAACACAACCTCCATGATGAAAGGAACTAATACCCTAA

TCTAAGGGCGACAGCCCTAAGAATTAAT--------ACATTAACCCAA-TTGATCCAATA

ACTT--GATCAACGAACCAAGTTACCCTGGGGATAACAGCGCAATCCATTTCAAGAGCTC

ATATCGACAAATGGGTTTACG-ACC-TCGATGTTGGATCAGGATATCCCAGTG-GCGCAG

CCGCTA-CTAA-CGGTTC

>M. pulchra MN534716, MN534507, MN534609

TTATTATCAGCTTATCCTTAACTTACACATGCAAGTATCAGCACACCCGTGAGAACGCCC

TTAAACCTT-ACTAGGACAAGGAGCCGGTATCAGGCACAG---ACCCA----TCTAGCCC

ATTACACCTAGCTTTGCCACTCCCTCAAGGGTCTTCAGCAGTGATTAACATTGTTTATAA

GCGACAGCTTGACTCAGTTAAAGGACAC--AGGGCCGGCTAATACGGTGCCAGCCGCCGC

GGCTACACCGCTAGGCTCAAGTTGATATT-ATTCGGCGTTAAGCGTGTTTAAAGTGCTT-

TTCAAGATTAGAACTAAACCCTAACCAAGCTGTGACACGCTTGCTATCAGGAAAAACAAA

TACGAAAGTTATTCTAACCCACCCACTTGAACCCACGACAGCTAGGACACAAACTGGGAT

TAGGTACCCCACTATGCCTAGCCGTAAAATATTTATTTACACCT-TTAACCGCCAGGGGA

TTACGAGCCCAAGCTTAAAACCCAAAGGACTTGACGGTGTCCCACCCACCTAGAGGAGCC

TGTTCTATAA-TCGATTCTCCCCCGTTATACCTCACCACTTCTAGCTTC--TCAGCCTGT

ATACCTCCGTCGTAAGCTTACCATATGAACGCC-TTTTAGTGAGCAAAAAGAT--TTTAC

ATAAATACGTCAGGTCAAGGTGCAGCCTACGAAATGGCAAGAAATGGGCTACAATTTCTA

TGAGCATAGAACACAACGAAAGACTACATGCAA-CTTAGTCATGAAGGCGGATTTAGTAG

TAAAAAGAAAATAGAGAGTTCTTTTTAATCAGGCGCTGGGACGCGTACACACCGCCCGTC

ACCCTCTTCAAACGCTTAA-ACTCAG-TTTATAACAAA-CTCATGCACCACAGAAGAGGT

AAGTCGTAACATGGTAAGCGTACTGGAAAGTGCGCTTGGAATAAACAAAATGTAGCTTAA

CTAAA-GCATCTCGCTTACACCGAGAACATGTCCGTGAAACCCAGATCATTTTGA--GCC

AAAAATCTAGCTCCC--CTCAACCT-AATGAACCCCC-CA-CAATT-CAAATA--TTTAA

TAAAACATTTTATAC-ATAAAGTATAGGCGATAAAAAAATGTCTA-GAAGCAATAGAG-T

TAGTACCGTAAGGGAATGATGAAATAATAATGAAAAA--CTGCAAGCACAAAAAAGTAGA

GCTACAACCTCGTACCTTTTGCATCATGGTCTAACGAGTCCAACCAAGCAAAATGCACTT

TAAGTTTGACCCCCCGAAACTAGGTGAGCTACTCAAAAACAGCCTTT-AGGGCCAACCCG

TCTCTGTTGCAAAAGAGTGGGAAGATTCT-TGAGTAGGGGTGACAGACCTATCGAACCTA

GAGATAGCTGGTTATTCAGAAAAAGGATTTAAGTCCTGCCTTAAGATTTCCCGTACAATC

--GAGCACTTTTTT-AACCTTAAGAGTTATTCAAATAAGGTACAGCCTATTTGAATTAGG

ATACAACCTCCATCAA--

------TT-AAAAAAGCGTTAAAGCTTTATTAA--TTTTCTTTAAAAAATTCCCTCA-AC

TTTCTATAACCCTT-AACTTTGTACTGAATAATTTTATATTAGTATAAAAACTTTTATGT

TAGAACTAGTAACAAGAAGC-AGCCCTTCTCCA-AAATGTAAGTTTAAGCCAAAATGAAC

AACTCATTGGCAATTAAAGTCATTAAACCTCTAGTAGTAACCCC----TCAAGAAAAATC

TAC------------------TATATTTCAACGTTAATCTTACACTAGAACATTTCTGGA

AAGATAAAAAGAGTGGGAAGGAACTCGGCAAACAACTTAACCCCGCCTGTTTACCAAAAA

CATCGCCTCTTGATAACCC---ATAAGAGGTCCAGCCTGCCCAGTGACAAA--GTTAAAC

GGCCGCGGTACCCTAACCGTGCAAAGGTAGCGCAATCACTTGTTCTTTAAATGAGGACCG

GTATGAACGGCATCACGAGGGTTATACTGTCTCCCCACTCAACTCAGTGAAACTGATCTT

CCCGTGAAGAAGCGGGAATAAAAATATAAGACGAGAAGACCCCATGGAGCTTAAAACTCA

GAATCAACTGCCACATT--TACAACCTCATAACCAC-GCAG-CCATGAC-CTCTAGTTTT

CGGTTGGGGTGACCGCGGAGTAAAACACAACCTCCATGATGAAAGGAACTAATACCCTAA

TCTAAGGGCGACAGCCCTAAGAATTAAT--------ACATTAACCCAA-TTGATCCAATC

ACTT--GATCAACGAACCAAGTTACCCTGGGGATAACAGCGCAATCCATTTCAAGAGCTC

ATATCGACAAATGGGTTTACG-ACC-TCGATGTTGGATCAGGATATCCCAGTG-GCGCAG
CCGCTA-CTAA-CGGTTC

>M. pulchra MN534717, MN534506, MN534610

TTATTATCAGCTTATCCTTAACTTACACATGCAAGTATCAGCACACCCGTGAGAACGCCC
TTAAACCTT-ACTAGGACAAGGAGCCGGTATCAGGCACAG---ACCCA----TCTAGCCC
ATTACACCTAGCTTTGCCACTCCCTCAAGGGTCTTCAGCAGTGATTAACATTGTTTATAA
GCGACAGCTTGACTCAGTTAAAGGACAT--AGGGCCGGCCAATACGGTGCCAGCCGCCGC
GGCTACACCGCTAGGCTCAAGTTGATATT-ATTCGGCGTTAAGCGTGTTTAAAGTGCTT-
TTCAAGATTAGAACTAAACCCTAACCAAGCTGTGACACGCTTGCTATCAGGAAAAACAAA
TACGAAAGTTATTCTAACCCACCCACTTGAACCCACGACAGCTAGGACACAAACTGGGAT
TAGGTACCCCACTATGCCTAGCCGTAAAATATTTATTTACACCT-TTAACCGCCAGGGGA
TTACGAGCCCAAGCTTAAAACCCAAAGGACTTGACGGTGTCCCACCCACCTAGAGGAGCC
TGTTCTATAA-TCGATTCTCCCCCGTTATACCTCACCACTTCTAGCTTC--TCAGCCTGT
ATACCTCCGTCGTAAGCTTACCATATGAACGCC-TTTTAGTGAGCAAAAAGAT--TTTAC
ATAAATACGTCAGGTCAAGGTGCAGCCTACGAAATGGCAAGAAATGGGCTACAATTTCTA
TGAGCATAGAACACAACGAAAGACTACATGCAA-CTTAGTCATGAAGGCGGATTTAGTAG
TAAAAAGAAAATAGAGAGTTCTTTTTAATCAGGCGCTGGGACGCGTACACACCGCCCGTC
ACCCTCTTCAAACGCTTAA-ACTCAG-TTTATAACAAA-CTCATGCACCACAGAAGAGGT
AAGTCGTAACATGGTAAGCGTACTGGAAAGTGCGCTTGGAATAAACAAAATGTAGCTTAA
CTAAA-GCATCTCGCTTACACCGAGAACATGTCCGTGAAACTCAGATCATTTTGA--GCC
AAAAATCTAGCTCCC--CTCAACCT-AATGAACCCCC-CA-CAATT-CAAATA--CTAAA
TAAAACATTTTATAC-ATAAAGTATAGGCGATAAAAAAATGTCTA-GAAGCAATAGAG-T
TAGTACCGTAAGGGAATGATGAAATAATAATGAAAAA--CTGCAAGCACAAAAAAGTAGA
GCTACAACCTCGTACCTTTTGCATCATGGTCTAACGAGTCCAACCAAGCAAAATGCACTT
TAAGTTTGACCCCCCGAAACTAGGTGAGCTACTCAAAAACAGCCTTT-AGGGCCAACCCG
TCTCTGTTGCAAAAGAGTGGGAAGATTCT-TGAGTAGGGGTGACAGACCTATCGAACCTA
GAGATAGCTGGTTATTCAGAAAAAGGATTTAAGTCCTGCCTTAAGATTTTACGTACAATC
--GAGCACTTTTTT-AACCTTAAGAGTTATTCAAATAAGGTACAGCCTATTTGAATTAGG
ATACAACCTCCATCAA--
------TT-AAAAAAGCGTTAAAGCTTTATTAA--TTTTCTTTAAAAAATTCCCTCA-AC

TTTCTATAACCCTT-AACTTTGTACTGAATAATTTTATATTAATATAAAAACTTTTATGT

TAGAACTAGTAACAAGAAGC-AGCCCTTCTCCA-AAATGTAAGTTTAAGCCAAAATGAAC

AACTCATTGGCAATTAAAGTCATTAAACCTCTAGTAGTAACCCC----TCAAGAAAAATC

TAC------------------TGTATTTCAACGTTAATCTTACACTAGAACATTTCTGGA

AAGATAAAAAGAATGGGAAGGAACTCGGCAAACAACTTAACCCCGCCTGTTTACCAAAAA

CATCGCCTCTTGATAACCC---ATAAGAGGTCCAGCCTGCCCAGTGACAAA--GTTAAAC

GGCCGCGGTACCCTAACCGTGCAAAGGTAGCGCAATCACTTGTTCTTTAAATGAGGACCG

GTATGAACGGCATCACGAGGGTTATACTGTCTCCCCACTCAACTCAGTGAAACTGATCTT

CCCGTGAAGAAGCGGGAATAAAAATATAAGACGAGAAGACCCCATGGAGCTTAAAACTCA

GAATCAACTGCCACATT--TATAACCTCATAACCAC-GCAG-CCATGAC-CTCTAGTTTT

CGGTTGGGGTGACCGCGGAGTAAAACACAACCTCCATGATGAAAGGAACTAATACCCTAA

TCTAAGGACGACAGCCCTAAGAATTAAT--------ACATTAACCCAA-TTGATCCAATC

ACTT--GATCAACGAACCAAGTTACCCTGGGGATAACAGCGCAATCCATTTCAAGAGCTC

ATATCGACAAATGGGTTTACG-ACC-TCGATGTTGGATCAGGATATCCCAGTG-GCGCAG

CCGCTA-CTAA-CGGTTC

>M. picta MN534718, MN534509, MN534611

TTATTGTCATCTCTCCCCCAACTTACACATGCAAGTATCAGCACACCCGTGAGAACGCCC

TTGAACCTT-TTTAGGACAAGGAGCCGGTATCAGGCACAG---ACT------CCTGGCCC

ATAACACCTAGCTTTGCCACTCCCTCAAGGGTATTCAGCAGTGATTAACATTGTTCATAA

GCGCCAGCTTGATTCAGTCAGGGGGCAC--AGAGCCGGCCAATACGGTGCCAGCCGCCGC

GGCTACACCGCTAGGCTCAAGCTGACATT-ATTCGGCGTTAAACGTGTTTAAAGTGCCT-

TAAA-GATTAGAGTTAAACTTCAACCAAGCCGTGACACGCTTGTTATTAAGAAACCCACA

CACGAAAGTTACTCTAACAAACCCACTTGAATCCACGACAGCTAGGACACAAACTGGGAT

TAGGTACCCCACTATGCCTAGCCGTAAAATATCCACTCACACCT-TTAACCGCCAGGGAA

TTACGAGCCCAAGCTTAAAACCCAAAGGACTTGACGGTGCCCCACCCACCTAGAGGAGCC

TGTTCTATAA-TCGATTCTCCCC-GATAAACCCCACCACTTCTAGCCTC--TCAGCCTGT

ATACCTCCGTCGTAAGCTTACCCTATGAATGCC-TTTTAGTGAGCAAAATGAT-TTGTCC

ATAAATACGTCAGGTCAAGGTGCAGCCTACGAAGTGGTAAGAAATGGGCTACAATTTCTA

CAAG--TAGAACCAAACGAAAGACTACATGAAA-CCTGGTCATGAAGGCGGATTTAGTAG

TAAAAAGAAAATAGAGAGTTCTTTTTAATTCGGCAATGGGGCGCGTTCACACCGCCCGTC

ACCCTCTTCAAATGCCTAA-AATCAG-TTTTTAACTAA-CTACTGCACCATAGAAGAGGT

AAGTCGTAACATGGTAAGCGTACTGGAAAGTGCGCTTGGAATAA-CCAAATGTTGCTTAA

CCAAA-GCCTCTCGCTTACCCCGAGAAGATATCCGTGAAACTCAGATCATCTTGA--GCT

AAAAACTTAGCTCTT--CTAAACCC-TATGAATACCC-CCCCCCA--AAAAAA--TAAAA

TAAAACATTTTATAC-TTTAAGTAAAGGCGATAAAAAAATGTCTA-GAAGCTATAGAAAC

TAGTACCGCAAGGGAAAAGTGAAATAATAATGAAAAA--CCTCAAGCACAATAAAGCAGA

GCTTAAGCCTCGTACCTTTTGCATCATGGTCTAACTAGTCTAACCAAGCACAAAGTACTT

TAAGTTTGACCCCCCGAAACTAGGTGAGCTACTCAAAAACAGCCTCT-AGGGCCAACCCG

TCTCTGTTGCAAAAGAGTGGGAAGATTTT-TGAGTAGCGGTGACAGACCTATCGAACCTA

GAGATAGCTGGTTATTCAGAAAAAGGATTTTAGTCCTGCCTTAAGTTTCCAAGTACTTTT

--TAATACCACACG-AACCTTAAGAGTTATTCAAATAAGGTACAGCCTATTTGAAACAGG

GTACAACCTCCGC--AAAGCAG

CCACCTTCT-AAAAAGCGTTAAAGCTTTATTAA--CTTCACTAAAAAA-TTCCTCTA-AC

TACTTAAAACCCTC--ACTCTGTACTGAATAATTTTATAGAACTATAAAAACTATTATGT

TAGAACTAGTAACAAGAAGA-AACCCTTCTCCAAAAATGTAAGCATAGGCCAAAATGAAT

AATTCATTGGCAGTTAAAGTTTTTAAGCCCATAGTAGCAACTTA----CCAAGAAAAACC

TAC-------------------TTACTTCAACGTTAACCTTACACCAGCACATTTCTGGA

AAGATTAAAAGAATGGGAAGGAACTCGGCAAACTA--TAACCCCGCCTGTTTACCAAAAA

CATCGCCTCTTGATACCC----ATAAGAGGTCCAGCCTGCCCAGTGACTAA--GTTAAAC

GGCCGCGGTACCCTAACCGTGCAAAGGTAGCGCAATCACTTGTTCTTTAAATGAGGACTA

GTATGAATGGCATCACGAGGGTTACACTGTCTCCCCACTCAACTCAGTGAAACTGATCTT

CCCGTGAAGAAGCGGGAATAAAAATATAAGACGAGAAGACCCCATGGAGCTTAAAACTCA

GAGTTAACTGCCACTTC--TTTAGCCTAGTACCCAC-GCAG-AAATAAC-TTCTAGTTTT

CGGTTGGGGTGACCGCGGAGTAAAACACAACCTCCACGAAGAAAGGAACTTATACCCTAA

TCCAAGAGCTACAGCTCTAAGAATTAAA--------ACATTAACCTAA-TTGATCCAAGC

ACTT--GATCAACGAACCAAGTTACCCTGGGGATAACAGCGCAATCCATTTCAAGAGCTC

ATATCGACAAATGGGTTTACG-ACC-TTGATGTTGGATCAGGATATCCCAGTGTGCGCAG

C-----------------

>M. picta MN534719, MN534510, MN534612

TTATTGTCATCTCTCCCCCAACTTACACATGCAAGTATCAGCACACCCGTGAGAACGCCC

TTGAACCTT-TTTAGGACAAGGAGCCGGTATCAGGCACAG---ACT------CCTGGCCC
ATAACACCTAGCTTTGCCACTCCCTCAAGGGTATTCAGCAGTGATTAACATTGTTCATAA
GCGCCAGCTTGATTCAGTCAGGGGGCAC--AGAGCCGGCCAATACGGTGCCAGCCGCCGC
GGCTACACCGCTAGGCTCAAGCTGACATT-ATTCGGCGTTAAACGTGTTTAAAGTGCCT-
TAAA-GATTAGAGTTAAACTTCAACCAAGCCGTGACACGCTTGTTATTAAGAAACCCACA
CACGAAAGTTACTCTAACAAACCCACTTGAATCCACGACAGCTAGGACACAAACTGGGAT
TAGGTACCCCACTATGCCTAGCCGTAAAATATCCACTCACACCT-TTAACCGCCAGGGAA
TTACGAGCCCAAGCTTAAAACCCAAAGGACTTGACGGTGCCCCACCCACCTAGAGGAGCC
TGTTCTATAA-TCGATTCTCCCC-GATAAACCCCACCACTTCTAGCCTC--TCAGCCTGT
ATACCTCCGTCGTAAGCTTACCCTATGAATGCC-TTTTAGTGAGCAAAATGAT-TTGTCC
ATAAATACGTCAGGTCAAGGTGCAGCCTACGAAGTGGTAAGAAATGGGCTACAATTTCTA
CAAG--TAGAACACAACGAAAGACTACATGAAA-CATGGTCATGAAGGCGGATTTAGTAG
TAAAAAGAAAATAGAGAGTTCTTTTTAATCCGGCACTGGGGCGCGTACACACCGCCCGTC
ACCCTCTTCAAATGCCTAA-AATCAG-TTTATAACTAA-CTACTGCACCATAGAAGAGGT
AAGTCGTAACATGGTAAGCGTACTGGAAAGTGCGCTTGGAATAA-CAAAATGTAGCTTAA
CCAAA-GCATCTCGCTTACACCGAGAAGATATCCGTGAAACTCAGATCATCTTGA--GCT
AAAAACTTAGCTCTT--CTAAACCC-TATGAATACCC-CCCCCCA--AAAAAA--TAAAA
TAAAACATTTTATAC-TTTAAGTAAAGGCGATAAAAAAATGTCTA-GAAGCTATAGAAAC
TAGTACCGCAAGGGAAAAGTGAAATAATAATGAAAAA--CCTCAAGCACAATAAAGCAGA
GCTTAAGCCTCGTACCTTTTGCATCATGGTCTAACTAGTCTAACCAAGCACAAAGTACTT
TAAGTTTGACCCCCCGAAACTAGGTGAGCTACTCAAAAACAGCCTCT-AGGGCCAACCCG
TCTCTGTTGCAAAAGAGTGGGAAGATTTT-TGAGTAGCGGTGACAGACCTATCGAACCTA
GAGATAGCTGGTTATTCAGAAAAAGGATTTTAGTCCTGCCTTAAGTTTCCAAGTACTTTT
--TAATACCACACG-AACCTTAAGAGTTATTCAAATAAGGTACAGCCTATTTGAAACAGG
GTACAACATCCGC--------------------------------------TTAAAGCAG
CCACCTTCT-AAAAAGCGTTAAAGCTTTATTAA--CTTCACTAAAAAA-TTCCTCTA-AC
TACTTAAAACCCTC--ACTCTGTACTGAATAATTTTATAGAACTATAAAAACTATTATGT
TAGAACTAGTAACAAGAAGA-AACCCTTCTCCAAAAATGTAAGCATAGGCCAAAATGAAT
AATTCATTGGCAGTTAAAGTTTTTAAGCCCATAGTAGCAACTTA----CCAAGAAAAACC
TAC-------------------TTACTTCAACGTTAACCTTACACCAGCACATTTCTGGA

```
AAGATTAAAAGAATGGGAAGGAACTCGGCAAACTA--TAACCCCGCCTGTTTACCAAAAA
CATCGCCTCTTGATACCC----ATAAGAGGTCCAGCCTGCCCAGTGACTAA--GTTAAAC
GGCCGCGGTACCCTAACCGTGCAAAGGTAGCGCAATCACTTGTTCTTTAAATGAGGACTA
GTATGAATGGCATCACGAGGGTTACACTGTCTCCCCACTCAACTCAGTGAAACTGATCTT
CCCGTGAAGAAGCGGGAATAAAAATATAAGACGAGAAGACCCCATGGAGCTTAAAACTCA
GAGTTAACTGCCACTTC--TTTAGCCTAGTACCCAC-GCAG-AAATAAC-TTCTAGTTTT
CGGTTGGGGTGACCGCGGAGTAAAACACAACCTCCACGAAGAAAGGAACTTATACCCTAA
TCCAAGAGCTACAGCTCTAAGAATTAAA--------ACATTAACCTAA-TTGATCCAAGC
ACTT--GATCAACGAACCAAGTTACCCTGGGGATAACAGCGCAATCCATTTCAAGAGCTC
ATATCGACAAATGGGTTTACG-ACC-TTGATGTTGGATCAGGATATCCCAGTGTGCGCAG
C-----------------
>M. nilphamariensis MN534720, MN534613
------ACAGCTCCCTCTAAATTTACACATGCAAGTATCAGCATACCCGTGAGAACGCCC
TTTAACCTT-TATAGGACAAGGAGCCGGTATCAGGCACAG---ACAA------CTAGCCC
ATGACACCTAGCTTTGCCACACCCTCAAGGGTACTCAGCAGTGATTAACATTGTTTATAC
GCGCCAGCTTGAATCAGTTACAGAGCAA--AGAGCCGGCCAATACGGTGCCAGCCGCCGC
GGCTACACCGCTAGGCTCAAGTTGATATT-ATCCGGCGTTAAGCGTGTTTTAAGTGCCT-
AAAA-AATTAGAATTAAACTTAAACCAAGCCGTGACACGCTTGTTCCTAAGAAAAACAAA
AACGAAAGTTATTCTAACCCAACCACTTGAACCCACGACAGCTAGGGCACAAACTGGGAT
TAGGTACCCCACTATGCCTAGCCGTAAAACATACACTTACACCT-TTAATCGCCAGGGAA
TTACGAGCCCTAGCTTAAAACCCAAAGGACTTGACGGTGTCCCACCCACCTAGAGGAGCC
TGTTCTATAA-TCGATTCCCCCC-------------------------------------
------------------------------------------------------------
------------------------------------------------------------
------------------------------------------------------------
------------------------------------------------------------
------------------------------------------------------------
------------------------------------------------------------
------------------------------------------------------------
------------------------------------------------------------
```


-----------TCTGACCGTGCAAAGGTAGCGTAATCACTTGTTCTTTAAATGAGGACTA
GTATGAACGGCATCACGAGGGTTACACTGTCTCCCTATTCCATTCAGTGAAACTGATCTT
CCCGTGAAGAAGCGGGAATATAAATATAAGACGAGAAGACCCCATGGAGCTTTAAACTCA
GTGTCATCTGCCACTTC--AATACCCTCTTAATTAC-GCAGACCATGAT-AACTAGTTTT
CGGTTGGGGTGACCGCGGAGTAAAACAAAACCTCCACAACGAAAGGAACTAACAACCTAA
TCCAAGAGCAACATCTCTAAGAATCAAC--------AAATTGACTTAA-TTGATCCAATT
ACTT--GATCAACGAACCAAGTTACCCTGGGGATAACAGCGCAATCCATTTCAAGAGCTC
ATATCGACAAATGGGTTTACG-ACC-TCGATGTTGGATCAGGGTATCCCAGTG-GCGCAG
CAGCTA-CTAA-CGGTTC
>M. nilphamariensis MN534721, MN534614
TTATTCACAGCTCCCTCTAAATTTACACATGCAAGTATCAGCATACCCGTGAGAACGCCC
TTTAACCTT-TATAGGACAAGGAGCCGGTATCAGGCACAG---ACAA------CTAGCCC
ATGACACCTAGCTTTGCCACACCCTCAAGGGTACTCAGCAGTGATTAACATTGTTTATAA
GCGCCAGCTTGAATCAGTTACAGAGCAA--AGAGCCGGCCAATACGGTGCCAGCCGCCGC
GGCTACACCGCTAGGCTCAAGTTGATATT-ATCCGGCGTTAAGCGTGTTTTAAGTGCCT-

AAAA-GATTAGAATTAAACTTAAACCAAGCCGTGACACGCTTGTTCCTAAGAAAAACAAA

AACGAAAGTTATTCTAACCCAACCACTTGAACCCACGACAGCTAGGGCACAAACTGGGAT

TAGGTACCCCACTATGCCTAGCCGTAAAACATACACTTACACCT-TTAATCGCCAGGGAA

TTACGAGCCCATGCTTAAAACCCAAAGGACTTGACGGTGTCCCACCCACCTAGAGGAGCC

TGTTCTATAA-TCGATTCCCCCC-------------------------------------

-----------TCTGACCGTGCAAAGGTAGCGTAATCACTTGTTCTTTAAATGAGGACTA

GTATGAACGGCATCACGAGGGTTACACTGTCTCCCTATTCCATTCAGTGAAACTGATCTT

CCCGTGAAGAAGCGGGAATATAAATATAAGACGAGAAGACCCCATGGAGCTTTAAACTCA

GTGTCATCTGCCACTTC--AATACCCTCTTAATTAC-GCAGACCATGAT-AACTAGTTTT

CGGTTGGGGTGACCGCGGAGTAAAACAAAACCTCCACAACGAAAGGAACTAACAACCTAA

TCCAAGAGCAACATCTCTAAGAATCAAC--------AAATTGACTTAA-TTGATCCAATT

ACTT--GATCAACGAACCAAGTTACCCTGGGGATAACAGCGCAATCCATTTCAAGAGCTC

ATATCGACAAATGGGTTTACG-ACC-TCGATGTTGGATCAGGGTATCCCAGTG-GCGCAG

CAGCTA-CTAA-CGGTTC

>M. ornata MN534722, MN534511, MN534615

TTATTATCAGCTTCCTCTAAACTTACACATGCAAGTATCAGCATACCCGTGAGAACGCCC

TTCAACCTT-AATAGGACAAGGAGCTGGTATCAGGCACAG---ACAA------CTAGCCC

ATAACACCTAGCTTTGCCACACCCCCAAGGGTACTCAGCAGTGATTAACATTGTTCATAA

GCGCCAGCTTGACTCAGTTATAGAGTGT--AGAGCCGGCCAATACGGTGCCAGCCGCCGC

GGCTACACCGCTAGGCTCAAGTTGATATT-ATCCGGCGTTAAGCGTGTTTTAAGT-ACT-

AAAAAGATTAGGGTTAAACTTAAACCAAGCCGTGACACGCTTGTTCTTAAGAAAAACAAA

AACGAAAGTTACTCTAACTAAACCACTTGAACCCACGACAGCTAGGGCACAAACTGGGAT

TAGGTACCCCACTATGCCTAGCCGTAAAACGTAAACTTACACCT-TTAATCGCCAGGGGA

TTACGAGCCCAAGCTTAAAACCCAAAGGACTTGACGGTGTCCCACCCACCTAGAGGAGCC

TGTTCTATAA-TCGATTATCCCC-GATACACCTCACCATTTTTAGCCTT--TCAGCCTGT

ATACCTCCGTCGTAAGCTTACCATATGAACGCC-TTTCTGTGAGCTAAAAGAT--CTTTC

ATAAGCACGTCAGGTCAAGGTGCAGCCAACGAAATGGCAAGCAATGGGCTACAATTTCTA

GCCA--TAGAACACA-CGAAAGACTACATGCAA-TCCGGTCATGAAGGCGGATTTAGAAG

TAAAAAGAAAATAGAGAGTTCTTTTTAATTAGGCGCTGGGACGCGTACACACCGCCCGTC

ACCCTCTTCAAATGCCCAA-CCTTAG-TAACTAACAAC-CCAAAGCACCACAGAAGAGGT

AAGTCGTAACACGGTAAGCGTACTGGAAAGTGCGCTTGGATTAA-CAAAATGTACTTTAA

TTAAA-ACATTTCGCTTACACCGAAAACATATCTGTTGAACTCAGATCATTTTGA--GCT

AAAAACTTAGCTCTT--CTACATCA-AATGCCCACAC-CTCTTAA-CTATTA---CTAAA

TAAAACATTTTATCC-ATTTAGTAACGGCGATTAAAAAATGTCTA-GAAGCTATAGAAAC

CAGTACCGCAAGGGAAAAGTGAAATAAAAATGAAATA-TTGTAAAGCATAACATAGCAGA

GATTACCCCTCGTACCTTTTGCATCATGGTCTAACTAGTCTAACCAAGCACAATGCATTT

TAAGTTTGACATCCCGAAACTAGGTGAGCTACTTAAGAACAGCCTTC-AGGGCCAACCCG

TCTCTGTTGCAAAAGAGTGGGAAGATTCT-TTAGTAGAGGTGACAGACCTATCGAACCTA

GAGATAGCTGGTTATTCAAGAAAAGGATTTTAGTCCTACCTAAGGTTCCAC-ATATA-TT

A-TAATACACAACA-AACCTTTAGAGCTATTCAAATAAGGTACAGCCTATTTGATACAGG

ATACAACCTCA---

-----TTA-AAAAAAGCGTTAAAGCTTCATTG---TAAAAACCAAAAA-TTCCACCA-AT

AAACTACAACCCTT-TATTTCGTATTGAATGACTTCATACCCCTATGAAGAACTATATGT

TAGAACTAGTAACAAGAAGACAGCCCTTCTCCA-AAATGTAAGCTTGCGCCATAATGAAC

TAATCACTGGCACTTAAAGTCTTT--ACCAATAGTAAC-TCTTT----TCTAGAAAACCC

TAT------------------TTATTAA-TACGTCAACCTTACACAAGCACATCTCTGGA

AAGATTAAAAGAATAGGAAGGAACTCGGCAAATT--CTAACCCCGCCTGTTTACCAAAAA

CATCGCCTTCTGATACAC----ATAGAAGGTCCAGCCTGCCCAGTGACTAA--GTTAAAC

GGCCGCGGTACCCTAACCGTGCAAAGGTAGCGCAATCACTTGTTCTTTAAATGAGGACTA

GTATGAACGGCATCACGAGGGTTATACTGTCTCCCTATTCCAATCAGTGAAACTGATCTT

CCCGTGAAGAAGCGGGAATAAATATATAAGACGAGAAGACCCCATGGAGCTTTAAACTCA

GTACCACCTGCTATTTT--CAAATCCTATTAATCAC-GCAG-ACATGGT-AACTAGTTTT

CGGTTGGGGTGACCGCGGAGCAAAACAAAACCTCCATAACGAAAGGAACTAACAACCTAA

TCCAAGAGCAACATCTCTAAGAATTAAA--------AAATTAACTTAA-TTGATCCAATT

ACTT--GATCAACGAACCAAGTTACCCTGGGGATAACAGCGCAATCCATTTAAAGAGCTC

TTATCGACAAATGGGTTTACGAACC-TCGATGTTGGATCAGGGTATCCCAGTG-GCGCAG

CAGCTA-CTAA-AGGTTC

>M. ornata MN534723, MN534512, MN534616

TTATTATCAGCTTCCTCTAAACTTACACATGCAAGTATCAGCATACCCGTGAGAACGCCC

TTCAACCTT-AATAGGACAAGGAGCTGGTATCAGGCACAG---ACAA------CTAGCCC

ATAACACCTAGCTTTGCCACACCCCCAAGGGTACTCAGCAGTGATTAACATTGTTCATAA

GCGCCAGCTTGACTCAGTTATAGAGTGT--AGAGCCGGCCAATACGGTGCCAGCCGCCGC

GGCTACACCGCTAGGCTCAAGTTGATATT-ATCCGGCGTTAAGCGTGTTTTAAGT-ACT-

AAAAAGATTAGGGTTAAACTTAAACCAAGCCGTGACACGCTTGTTCTTAAGAAAAACAAA

AACGAAAGTTACTCTAACTAAACCACTTGAACCCACGACAGCTAGGGCACAAACTGGGAT

TAGGTACCCCACTATGCCTAGCCGTAAAACGTAAACTTACACCT-TTAATCGCCAGGGGA

TTACGAGCCCAAGCTTAAAACCCAAAGGACTTGACGGTGTCCCACCCACCTAGAGGAGCC

TGTTCTATAA-TCGATTATCCCC-GATACACCTCACCATTTTTAGCCTT--TCAGCCTGT
ATACCTCCGTCGTAAGCTTACCATATGAACGCC-TTTCTGTGAGCTAAAAGAT--CTTTC
ATAAGCACGTCAGGTCAAGGTGCAGCCAACGAAATGGCAAGCAATGGGTTACAATTTCTA
GCCA--TAGAACACA-CGAAAGACTACATGCAA-TCCGGTCATGAAGGCGGATTTAGAAG
TAAAAAGAAAATAGAGAGTTCTTTTTAATTAGGCGCTGGGACGCGTACACACCGCCCGTC
ACCCTCTTCAAATGCCCAA-CCTTAG-TAACTAACAAC-CCACAGCACCACAGAAGAGGT
AAGTCGTAACACGGTAAGCGTACTGGAAAGTGCGCTTGGATTAA-CAAAATGTACTTTAA
TTAAA-ACATTTCGCTTACACCGAATACATATCTGTTGAACTCAGATCATTTTGA--GCT
AAAAACTTAGCTCTT--CTACATCA-AATGCCCACAC-CTCTTAA-CTATTA---TTAAA
TAAAACATTTTATCC-ATTTAGTAACGGCGATTAAAAAATGTCTA-GAAGCTATAGAAAC
CAGTACCGCAAGGGAAAAGTGAAATAAAAATGAAATA-TTGTAAAGCATAACATAGCAGA
GACTACCCCTCGTACCTTTTGCATCATGGTCTAACTAGTCTAACCAAGCACAATGCATTT
TAAGTTTGACATCCCGAAACTAGGTGAGCTACTTAAGAACAGCCTTC-AGGGCTAACCCG
TCTGTGTTGCAAAAGAGTGGGAAGATTCT-TTAGTAGAGGTGACAGACCTATCGAACCTA
GAGATAGGTGGTTATTCAAGAAAAGGATTTTAGTCCTACCTAAGGTTCCAC-ATATA-TT
A-TAATACACAACA-AACCTTTAGAGCTATTCAAATAAGGTACAGCCTATT-GATACAGG
ATACAACCTCA--
-----TTA-AAAAAAGCGTTAAAGCTTCATTG---TAAAAACCAAAAA-TTCCACCA-AT
AAACTACAACCCTT-TATTTCGTATTGAATGACTTCATACCCCTATGAAGAACTATATGT
TAGAACTAGTAACAAGAAGACAGCCCTTCTCCA-AAATGTAAGCTTGCGCCATAATGAAC
TAATCACTGGCACTTAAAGTCTTT--ACCAATAGTAAC-TCTTT----TCTAGAAAACCC
TAT------------------TTATTAA-TACGTCAACCTTACACAAGCACATCTCTGGA
AAGATTAAAAGAATAGGAAGGAACTCGGCAAATT--CTAACCCCGCCTGTTTACCAAAAA
CATCGCCTTCTGATACAC----ATAGAAGGTCCAGCCTGCCCAGTGACTAA--GTTAAAC
GGCCGCGGTACCCTAACCGTGCAAAGGTAGCGCAATCACTTGTTCTTTAAATGAGGACTA
GTATGAACGGCATCACGAGGGTTATACTGTCTCCCTATTCCAATCAGTGAAACTGATCTT
CCCGTGAAGAAGCGGGAATAAATATATAAGACGAGAAGACCCCATGGAGCTTTAAACTCA
GTACCACCTGCTATTTT--CAAATCCTATTAATCAC-GCAG-ACATGGT-AACTAGTTTT
CGGTTGGGGTGACCGCGGAGCAAAACAAAACCTCCATAACGAAAGGAACTAACAACCTAA
TCCAAGAGCAACATCTCTAAGAATTAAA--------AAATTAACTTAA-TTGATCCAATT

ACTT--GATCAACGAACCAAGTTACCCTGGGGATAACAGCGCAATCCATTTAAAGAGCTC

TTATCGACAAATGGGTTTACG-ACC-TCGATGTTGGATCAGGGTATCCCAGTG-GCGCAG

CAGCTA-CTAA-AGGTTC

>M. mihintalei MN534724, MN534513, MN534617

TTATTATCAACTTTCCCTAAACTTACACATGCAAGTATCAGCACACCCGTGAGAACGCCC

TTCAACCTTTATTAGACCAAGGAGCCGGTATCAGGCACAG---TTTA-----TCTAGCCC

ATGACACCTAGCTTTGCCACACCCTCAAGGGTACTCAGCAGTGATTAACATTGTTAATAA

GCGCCAGCTTGACTCAGTTATAGAGAAGT-AGAGCCGGCCAATACGGTGCCAGCCGCCGC

GGCTACACCGCTAGGCTCAAGTTGATGTT-ATCCGGCGTTAAGCGTGTTTTAAGTGATT-

AAAA-ATTTAGAATTAAACTTAAACCAAGCCGTGACACGCTTGTTTCTAAAAAAAACAAA

AACGAAAGTTATTCTAATCCACCCACTTGAACCCCCGACAGCAAAGACACAGACTGGGAT

TAGGTACCCCACTATGCCTAGCCGTAAAATATTTAATTACACCT-TTAACCGCCAGGGAA

TTACGAGCTCAAGCTTAAAACCCAAAGGACTTGACGGTGCCCCACCCACCTAGAGGAGCC

TGTTCTATAA-TCGATTCCCCCCGATCTACCTTACCATTTTTAGCCTA--TCAGCCTGT

ATACCTCCGTCGTAAGCTTACCATATGAACGCT-TTTTAGTGAGCTAAAAGAT--TTTAC

ATAAATACGTCAGGTCAAGGTGCAGCCAACGAAATGGCAAGCAATGGGCTACAATTTCTA

-CCA--TAGAACACA-CGAAAGACTACATGCAA-CTCAGTCATGAAGGCGGATTTAGAAG

TAAAAAGAAAATAGAGAGTTCTTTTTAATTAGGCGCTGGGACGCGTACACACCGCCCGTC

ACCCTCTTCAAATGCTTTA-CTGAAG-TATATAACAAC-CCACTGCACCACAGAAGAGGT

AAGTCGTAACACGGTAAGCGTACTGGAAAGTGCGCTTGGTTTAA-CAAAATGTACTTTAA

TTAAA-ACATTTCGCTTACACCGAAAACATATCTGTTGAAGTCAGATCATTTTGA--GCC

AAAAACTCAGCTCTAATTTATTTCA-AATGCATTTTC-TAATTAA-TCCACAA---CAAA

TAAAACATTTTATCA-ATCTAGTAAAGGCGATTAAAAAATTCTTA-GAAGCTATAGAAAC

AAGTACCGCAAGGGAAAAGTGAAATAAAAATGAAATA-TCTTAAAGCACTAAATAGCAGA

GATTTAATCTTGTACCTTTTGCATCATGGTCTAACTAGTCCAACCAAGCGAAACGCACTT

TAAGTTTGACCCCCCGAAACTAGGTGAGCTACTTAAGAACAGCCTTT-AGGGCCAACCCG

TCTCTGTTGCAAAAGAGTGGGAAGATTCT-TTAGTAGAGGTGATAGACCTATCGAACCTA

GAGATAGCTGGTTATTCAGGAAAAGGATTTAAGTCCTACCTTAGGTTTTTT-ATACC-TT

AAAAATACTTAATT-AACCTTAAGAGCTATTCAGATAAGGTACAGCCTATCTGAAACAGG

ATACAACCTCAACCAA--------------------------------------AAGCAG

CCACCTTT-AAGAAAGCGTTAAAGCTTCATTA---TCTTAACCAAAAA-TTCCACCA-CC
CAAACTAAACCCTT-AGACTTATACTGAATGACTTCATATTATTATGAAGAACTATATGT
TAGAACTAGTAACAAGAAGT-AGACCTTCTCCAAAAATGTAAGCTTGAGCCATAATGAAC
CCCTCACTGGCATTTAAAGTATAAATTCTACTAGTAGTAACTTC---AACAAGAAAACCC
TAC------------------TTAATTA-TACGTCAATCTTACACCAGTACATTCCTGGA
AAGATTAAAAGAGTGGGAAGGAACTCGGCAAATAA-TTAACCCCGCCTGTTTACCAAAAA
CATCGCCTCTTGACCCCC----ATAAGAGGTCCAGCCTGCCCAGTGACCAA--GTTAAAC
GGCCGCGGTACCCTAACCGTGCAAAGGTAGCGCAATCACTTGTTCTTTAAATGAGGACTA
GTATGAACGGCATCACGAGGGTTACACTGTCTCCCCACTCCACTCAGTGAAACTGATCCC
CCCGTGAAGAAGCGGGGATAAAAATATAAGACGAGAAGACCCCATGGAGCTTTAAACTCA
GTATCATCTGCTACTAC--AATAACCAAGTAACAAC-GCAA-CAATGAC-AACTGGTTTT
TGGTTGGGGTGACCGCGGAGTAAAACAAAACCTCCACAACGAAAGGAACTAACAACCTAA
TCTAAGAGCGACAGCTCTAAGAATCATAT-------AAATTGACTTAA-TTGATCCAATC
ACTT--GATCAACGAACCAAGTTACCCTGGGGATAACAGCGCAATCCATTTAAAGAGCCC
ATATCGACAAATGGGTTTACG-ACC-TCGATGTTGGATCAGGGTATCCCAGTG-GCGCAG
CAGCTA-CTAA-CGGTTC

>M. mihintalei MN534725, MN534514, MN534618

TTATTATCAACTTTCCCTAAACTTACACATGCAAGTATCAGCACACCCGTGAGAACGCCC
TTCAACCTTTATTAGACCAAGGAGCCGGTATCAGGCACAG---TTTA-----TCTAGCCC
ATGACACCTAGCTTTGCCACACCCTCAAGGGTACTCAGCAGTGATTAACATTGTTAATAA
GCGCCAGCTTGACTCAGTTATAGAGAAGT-AGAGCCGGCCAATACGGTGCCAGCCGCCGC
GGCTACACCGCTAGGCTCAAGTTGATGTT-ATCCGGCGTTAAGCGTGTTTTAAGTGATT-
AAAA-ATTTAGAATTAAACTTAAACCAAGCCGTGACACGCTTGTTTCTAAGAAAAACAAA
AACGAAAGTTATTCTAATCCACCCACTTGAACCCACGACAGCTAGGACACAAACTGGGAT
TAGGTACCCCACTATGCCTAGCCGTAAAATATTTAATTACACCT-TTAACCGCCAGGGAA
TTACGAGCTCAAGCTTAAAACCCAAAGGACTTGACGGTGCCCCACCCACCTAGAGGAGCC
TGTTCTATAA-TCGATTCCCCCCGATCTACCTTACCATTTTTAGCCTA--TCAGCCTGT
ATACCTCCGTCGTAAGCTTACCATATGAACGCT-TTTTAGTGAGCTAAAAGAT--TTTAC
ATAAATACGTCAGGTCAAGGTGCAGCCAACGAAATGGCAAGCAATGGGCTACAATTTCTA
-CCA--TAGAACACA-CGAAAGACTACATGCAA-CTCAGTCATGAAGGCGGATTTAGAAG

TAAAAAGAAAATAGAGAGTTCTTTTTAATTAGGCGCTGGGACGCGTACACACCGCCCGTC

ACCCTCTTCAAATGCTTTA-CTGAAG-TATATAACAAC-CCACTGCACCACAGAAGAGGT

AAGTCGTAACACGGTAAGCGTACTGGAAAGTGCGCTTGGTTTAA-CAAAATGTACTTTAA

TTAAA-ACATTTCGCTTACACCGAAAACATATCTGTTGAAGTCAGATCATTTTGA--GCC

AAAAACTCAGCTCTAATTTATTTCA-AATGCATTTTC-TAATTAA-TCCACAA---CAAA

TAAAACATTTTATCA-ATCTAGTAAAGGCGATTAAAAGATTCTTA-GAAGCTATAGAAAC

AAGTACCGCAAGGGAAAAGTGAAATAAAAATGAAATA-TCTTAAAGCACTAAATAGCAGA

GATTTAATCTTGTACCTTTTGCATCATGGTCTAACTAGTCCAACCAAGCGAAACGCACTT

TAAGTTTGACCCCCCGAAACTAGGTGAGCTACTTAAGAACAGCCTTT-AGGGCCAACCCG

TCTCTGTTGCAAAAGAGTGGGAAGATTCT-TTAGTAGAGGTGATAGACCTATCGAACCTA

GAGATAGCTGGTTATTCAGGAAAAGGATTTAAGTCCTACCTTAGGTTTTTT-ATACC-TT

AAAAATACTTAATT-AACCTTAAGAGCTATTCAGATAAGGTACAGCCTATCTGAAACAGG

ATACAACCTCAAC---AAGCAG

CCACCTTT-AAGAAAGCGTTAAAGCTTCACTA---TCTTAACCAAAAA-TTCCACCA-CC

CAAACTAAACCCTT-AGACTTATACTGAATGACTTCATATTATTATGAAGAACTATATGT

TAGAACTAGTAACAAGAAGT-AGACCTTCTCCAAAAATGTAAGCGTGAGCCATAATGAAC

CCCTCACTGGCATTTAAAGTATAAATTCTACTAGTAGTAACTTC---AACAAGAAAACCC

TAC------------------TTAATTA-TACGTCAATCTTACACCAGTACATTCCTGGA

AAGATTAAAAGAGTGGGAAGGAACTCGGCAAATAA-TTAACCCCGCCTGTTTACCAAAAA

CATCGCCTCTTGACCCCC----ATAAGAGGTCCAGCCTGCCCAGTGACCAA--GTTAAAC

GGCCGCGGTACCCTAACCGTGCAAAGGTAGCGCAATCACTTGTTCTTTAAATGAGGACTA

GTATGAACGGCATCACGAGGGTTACACTGTCTCCCCACTCCACTCAGTGAAACTGATCCC

CCCGTGAAGAAGCGGGGATAAAAATATAAGACGAGAAGACCCCATGGAGCTTTAAACTCA

GTATCATCTGCTACTAC--AATAACCAAGTAACAAC-GCAA-CAATGAC-AACTGGTTTT

TGGTTGGGGTGACCGCGGAGTAAAACAAAACCTCCACAACGAAAGGAACTAACAACCTAA

TCTAAGAGCGACAGCTCTAAGAATCATAT-------AAATTGACTTAA-TTGATCCAATC

ACTT--GATCAACGAACCAAGTTACCCTGGGGATAACAGCGCAATCCATTTAAAGAGCCC

ATATCGACGAATGGGTTTACG-ACC-TCGATGTTGGATCAGGGTATCCCAGTG-GCGCAG

CAGCTA-CTAA-CGGTTC

>M. mihintalei MN534726, MN534515, MN534619

TTATTATCAACTTTCCCTAAACTTACACATGCAAGTATCAGCACACCCGTGAGAACGCCC

TTCAACCTTTATTAGACCAAGGAGCCGGTATCAGGCACAG---TTTA-----TCTAGCCC

ATGACACCTAGCTTTGCCACACCCTCAAGGGTACTCAGCAGTGATTAACATTGTTAATAA

GCGCCAGCTTGACTCAGTTATAGAGAAGT-AGAGCCGGCCAATACGGTGCCAGCCGCCGC

GGCTACACCGCTAGGCTCAAGTTGATGTT-ATCCGGCGTTAAGCGTGTTTTAAGTGATT-

AAAA-ATTTAGAATTAAACTTAAACCAAGCCGTGACACGCTTGTTTCTAAGAAAAACAAA

AACGAAAGTTATTCTAATCCACCCACTTGAACCCACGACAGCTAGGACACAAACTGGGAT

TAGGTACCCCACTATGCCTAGCCGTAAAATATTTAATTACACCT-TTAACCGCCAGGGAA

TTACGAGCTCAAGCTTAAAACCCAAAGGACTTGACGGTGCCCCACCCACCTAGAGGAGCC

TGTTCTATAA-TCGATTTCCCCCCGATCTACCTTACCATTTTTAGCCTA--TCAGCCTGT

ATACCTCCGTCGTAAGCTTACCATATGAACGCT-TTTTAGTGAGCTAAAAGAT--TTTAC

ATAAATACGTCAGGTCAAGGTGCAGCCAACGAAATGGCAAGCAATGGGCTACAATTTCTA

-CCA--TAGAACACA-CGAAAGACTACATGCAA-CTCAGTCATGAAGGCGGATTTAGAAG

TAAAAAGAAAATAGAGAGTTCTTTTTAATTAGGCGCTGGGACGCGTACACACCGCCCGTC

ACCCTCTTCAAATGCTTTA-CTGAAG-TATATAACAAC-CCACTGCACCACAGAAGAGGT

AAGTCGTAACACGGTAAGCGTACTGGAAAGTGCGCTTGGTTTAA-CAAAATGTACTTTAA

TTAAA-ACATTTCGCTTACACCGAAAACATATCTGTTGAAGTCAGATCATTTTGA--GCC

AAAAACTCAGCTCTAATTTATTTCA-AATGCATTTTC-TAATTAA-TCCACAA---CAAA

TAAAACATTTTATCA-ATCTAGTAAAGGCGATTAAAAAATTCTTA-GAAGCTATAGAAAC

AAGTACCGCAAGGGAAAAGTGAAATAAAAATGAAATA-TCTTAAAGCACTAAATAGCAGA

GATTTAATCTTGTACCTTTTGCATCATGGTCTAACTAGTCCAACCAAGCGAAACGCACTT

TAAGTTTGACCCCCCGAAACTAGGTGAGCTACTTAAGAACAGCCTTT-AGGGCCAACCCG

TCTCTGTTGCAAAAGAGTGGGAAGATTCT-TTAGTAGAGGTGATAGACCTATCGAACCTA

GAGATAGCTGGTTATTCAGGAAAAGGATTTAAGTCCTACCTTAGGTTTTTT-ATACC-TT

AAAAATACTTAATT-AACCTTAAGAGCTATTCAGATAAGGTACAGCCTATCTGAAACAGG

ATACAACCTCAAC---AAGCAG

CCACCTTT-AAGAAAGCGTTAAAGCTTCATTA---TCTTAACCAAAAA-TTCCACCA-CC

CAAACTAAACCCTTTAGACTTATACTGAATGACTTCATATTATTATGAAGAACTATATGT

TAGAACTAGTAACAAGAAGT-AGACCTTCTCCAAAAATGTAAGCTTGAGCCATAATGAAC

CCCTCACTGGCATTTAAAGTATAAATTCTACTAGTAGTAACTTC---AACAAGAAAACCC

TAC------------------TTAATTA-TACGTCAATCTTACACCAGTACATTCCTGGA
AAGATTAAAAGAGTGGGAAGGAACTCGGCAAATAA-TTAACCCCGCCTGTTTACCAAAAA
CATCGCCTCTTGACCCCC----ATAAGAGGTCCAGCCTGCCCAGTGACCAA--GTTAAAC
GGCCGCGGTACCCTAACCGTGCAAAGGTAGCGCAATCACTTGTTCTTTAAATGAGGACTA
GTATGAACGGCATCACGAGGGTTACACTGTCTCCCCACTCCACTCAGTGAAACTGATCCC
CCCGTGAAGAAGCGGGGATAAAAATATAAGACGAGAAGACCCCATGGAGCTTTAAACTCA
GTATCATCTGCTACTAC--AATAACCAAGTAACAAC-GCAA-CAATGAC-AACTGGTTTT
TGGTTGGGGTGACCGCGGAGTAAAACAAAACCTCCACAACGAAAGGAACTAACAACCTAA
TCTAAGAGCGACAGCTCTAAGAATCATAT-------AAATTGACTTAA-TTGATCCAATC
ACTT--GATCAACGAACCAAGTTACCCTGGGGATAACAGCGCAATCCATTTAAAGAGCCC
ATATCGACAAATGGGTTTACG-ACC-TCGATGTTGGATCAGGGTATCCCAGTG-GCGCAG
CAGCTA-CTAA-CGGTTC

>M. aurantiventris MN534727

TTACTGTCAGCTAATTCTCAACTTACACATGCAAGTATCCGCACACCCGTGAGAACGCCC
TTTAACCTC-TCTAGGTAAAGGAGCCGGTATCAGGCACAG---ACCT-----TCTAGCCC
ACAACACCTAGCTTTGCCACACCCTCAAGGGAATTCAGCAGTGATTAACATTGTTCATAA
GCGACAGCTTGACTCAGTTAAAGAACTA--AAAGCCGGCTAATACGGTGCCAGCCGCCGC
GGCTACACCGCTAGGCTCAAGTTGACGCT-ATCCGGCGTTAAGCGTGTTTTAAGTGCTC-
AAATAGATTAGAATTAAACCCCAACCAAGCTGTGACACGCTTGTTACTAAAAAAAACAAA
AACGAAAGTTATTCTAACCAGTCCACTTGACCCCACGACAGCTAGGACACAAACTGGGAT
TAGGTACCCCACTATGCCTAGCTATAAAATATTTATTCACACCT-TAAACCGCCAGGGGA
TTACGAGCTCAAGCTTAAAACCCAAAGGACTTGACGGTGCCCCACCCACCTAGAGGAGCC
TGTTCTATAA-TCGATTCTCCCC-GATATACCTCACCGCTTCTAGCCTT--TCAGCCTGT
ATACCTCCGTCGTAAGCTTACCATATGAACGCC-TCTTAGTGAGCTAAATGAT--TTCTC
ATATACACGTCAGGTCAAGGTGCAGCCCACGAAGCGGCAAGCAATGGGCTACAATTTCTA
TTGA--TAGAACACA-CGAAAGACTACATGCAA-CTTAGTCATGAAGGCGGATTTAGTAG
TAAAAAGAAAATAGAGAGTTCTTTTTAACCAGGCGCTGGGGCGCGTACACACCGCCCGTC
ACCCTCTTCAAACGCCACA-GTCTAG-TATTTAACAAC-CTAACGCATATTAGAAGAGGT
AAGTCGTAACACGGTAAGCGTACTGGAAAGTGCGCTTGGATTAA-CAAAATATAGCTTAA
CTTAAAGCACCTCGCTTACACCGAAGACATGTCTGTTTAACTCAGATTATTTTGAA-GCC

AAAAACTTAGCTCTC---TTCTCCT-TATGAGCCACT--CACAATT--TACTTT-TAAAA

TAAAACATTTTTCAT-TTTAGGTAAAGGCGATTAAAAAATATCTA-GAAGCTATAGAAAC

CAGTACCGCAAGGGAATAGTGAAATAATAATGAAAA---CCTTCAGCACCAAATAGCAGA

GCTTCTACCTCGTACCTTTTGCATCATGGTCTAACAAGTCTAACCAAGCAAAACGCATCT

AAAGTTTGACACCCCGAAACCAAGCGAGCTACTCAAAAACAGCCTTAT-GGGCGAACCCG

TCTCTGTTGCAAAAGAGTGGGACGATTTT-TAAGTAGAGGTGATAAACCTACCGAGCTTG

GAGATAGCTGGTTATTCAGGAAAAGGATATAAGTCCTACCTTAAGTTTATT-GTACT-AA

T-TAATACGCCTCA-AAACTATAGAGTTATTCAAATAAGGTACAGCCTATT-GAAACAGG

ATACAACCTCAACAAC-------------------------------------AAGCAG

CCACCTTTCTAAAAAGCGTTAAAGCTTTACTG----TTTATTCAAAAAATCCCCTTA-AC

TTTCTTAAACCCTT-TTATTAATACTGAATGACTTCATAATTATATGAAGAACCTTATGT

TAGAACTAGTAACAAGAAGA-AGCCCTTCTCCT-AAATGTAAGCATGAGCCAAAACGAAC

AAATCATTGGCATTTAAAATTTGTAAGCCCTTAGTAGTAACCCA----CCAAGAAAAGCC

TAC-----------------TAAACAT-CATGTCAACCTTACACTAGCACATTTATGGA

AAGATTAAAAGAATGGGAAGGAACTCGGCAAATAA-CTAACCCCGCCTGTTTACCAAAAA

CATCGCCTCCTGAATCTC----ATAGGAGGTCTAGCCTGCCCAGTGATAAA--ATTAAAC

GGCCGCGGTACCCTAACCGTGCAAAGGTAGCGCAATCACTTGTTCTTTAAATGAGGACTA

GTATGAACGGCATCACGAGGGTTACACTGTCTCCCCACTCTATTCAGTGAAACTGATTTT

CCCGTGAAGAAGCGGGAATAAAAATATAAGACGAGAAGACCCCATGGAGCTTAAAACTCA

GTATCACTTGCCACTTT--TAAAGCCTTTAAACCAC-GCAA-CCCTGAT-TACTAGTTTT

CGGTTGGGGTGACCGCGGAGAAAAACAAAACCTCCACGACGAAAGGACCTAACAACCTAA

TCCAAGAGCTACACCTCCAAGAATCAAC--------ACATTGACTAAA-TTGATCCATTT

TTT---GACCAACGAACCAAGTTACCCTGGGGATAACAGCGCAATCCATTTCAAGAGCCC

CTATCGACAAATGGGTTTACG-ACC-TCGATGTTGGATCAGGGTATCCAAGTG-GCGCAG

CCGCTA-CTAA-------

>M. aurantiventris MN534728

TTACTGTCAGCTAATTCTCAACTTACACATGCAAGTATCCGCACACCCGTGAGAACGCCC

TTTAACCTC-TCTAGGTAAAGGAGCCGGTATCAGGCACAG---ACCT-----TCTAGCCC

ACAACACCTAGCTTTGCCACACCCTCAAGGGAATTCAGCAGTGATTAACATTGTTCATAA

GCGACAGCTTGACTCAGTTAGAGAACTA--AGAGCCGGCTAATACGGTGCCAGCCGCCGC

GGCTACACCGCTAGGCTCAAGTTGACGCT-ATCCGGCGTTAAGCGTGTTTTAAGTGCTC-

AAATAGATTAGAATTAAACCCCAACCAAGCTGTGACACGCTTGTTACTAAGAAAAACAAA

AACGAAAGTTATTCTAACCAGTCCACTTGACCCCACGACAGCTAGGACACAAACTGGGAT

TAGGTACCCCACTATGCCTAGCTATAAAATATTTATTCACACCT-TAAACCGCCAGGGGA

TTACGAGCTCAAGCTTAAAACCCAAAGGACTTGACGGTGCCCCACCCACCTAGAGGAGCC

TGTTCTATAA-TCGATTCTCCCC-GATATACCTCACCGCTTCTAGCCTT--TCAGCCTGT

ATACCTCCGTCGTAAGCTTACCATATGAACGCC-TCTTAGTGAGCTAAATGAT--TTCTC

ATATACACGTCAGGTCAAGGTGCAGCCCACGAAGCGGCAAGCAATGGGCTACAATTTCTA

TTGA--TAGAACACA-CGAAAGACTACATGCAA-CTTAGTCATGAAGGCGGATTTAGTAG

TAAAAAGAAAATAGAGAGTTCTTTTTAACCAGGCGCTGGGGCGCGTACACACCGCCCGTC

ACCCTCTTCAAACGCCACA-GTCTAG-TATTTAACAAC-CTAACGCATATTAGAAGAGGT

AAGTCGTAACACGGTAAGCGTACTGGAAAGTGCGCTTGGATTAA-CAAAATATAGCTTAA

CTTAAAGCACCTCGCTTACACCGAAGACATGTCTGTTTAACTCAGATTATTTTGA--GCC

AAAAACTTAGCTCTC---TTCTCCT-TATGAGCCACT--CACAATT--TACTTT-TAAAA

TAAAACATTTTTCAT-TTTAGGTAAAGGCGATTAAAAAATATCTA-GAAGCTATAGAAAC

CAGTACCGCAAGGGAATAGTGAAATAATAATGAAAA---CCTTCAGCACCAAATAGCAGA

GCTTCTACCTCGTACCTTTTGCATCATGGTCTAACAAGTCTAACCAAGCAAAACGCATCT

AAAGTTTGACACCCCGAAACCAAGCGAGCTACTCAAAAACAGCCTTAT-GGGCGAACCCG

TCTCTGTTGCAAAAGAGTGGGACGATTTT-TAAGTAGAGGTGATAAACCTACCGAGCTTG

GAGATAGCTGGTTATTCAGGAAAAGGATATAAGTCCTACCTTAAGTTTATT-GTACT-AA

T-TAATACGCCTCA-AAACTATAGAGTTATTCAAATAAGGTACAGCCTATT-GAAACAGG

ATACAACCTCAACAAC--------------------------------------AAGCAG

CCACCTTTCTAAAAAGCGTTAAAGCTTTACTG----TTTATTCAAAAAATCCCCTTA-AC

TTTCTTAAACCCTT-TTATTAATACTGAATGACTTCATAATTATATGAAGAACCTTATGT

TAGAACTAGTAACAAGAAGA-AGCCCTTCTCCT-AAATGTAAGCATGAGCCAAAACGAAC

AAATCATTGGCATTTAAAATTTGTAAGCCCTTAGTAGTAACCCA----CCAAGAAAAGCC

TAC------------------TAAACAT-CATGTCAACCTTACACTAGCACATTTATGGA

AAGATTAAAAGAATGGGAAGGAACTCGGCAAATAA-CTAACCCCGCCTGTTTACCAAAAA

CATCGCCTCCTGAATCTC----ATAGGAGGTCTAGCCTGCCCAGTGATAAA--ATTAAAC

GGCCGCGGTACCCTAACCGTGCAAAGGTAGCGCAATCACTTGTTCTTTAAATGAGGACTA

GTATGAACGGCATCACGAGGGTTACACTGTCTCCCCACTCTATTCAGTGAAACTGATTTT

CCCGTGAAGAAGCGGGAATAAAAATATAAGACGAGAAGACCCCATGGAGCTTAAAACTCA

GTATCACTTGCCACTTT--TAAAGCCTTTAAACCAC-GCAA-CCCTGAT-TACTAGTTTT

CGGTTGGGGTGACCGCGGAGAAAAACAAAACCTCCACGACGAAAGGACCTAACAACCTAA

TCCAAGAGCTACACCTCCAAGAATCAAC--------ACATTGACTAAA-TTGATCCATTT

TTT---GACCAACGAACCAAGTTACCCTGGGGATAACAGCGCAATCCATTTCAAGAGCCC

CTATCGACAAATGGGTTTACG-ACC-TCGATGTTGGATCAGGGTATCCAAGTG-GCGCAG

CCGCTA-CTAA-------

>M. butleri MN534729, MN534516, MN534620

TTATTATCAGCTACCTCTCGACTTACACATGCAAGTATCAGCACACCCGTGAGAACGCCC

TTCAACCTC-ACCAGGACAAGGAGCTGGTATCAGGCGCAG---ATTT-----TCTAGCCC

ATTACACCTAGCTTTGCCACACCCTCAAGGGAATTCAGCAGTGATTAACATTGTTAATAA

GCGTCAGCTTGACTCAGTCAGAGAACCA--AGAGCCGGCTAATACGGTGCCAGCCGCCGC

GGCTACACCGCTAGGCTCAAGTTGATATT-ACTCGGCGTTAAGCGTGTTTAAAGTGCCT-

TTAA-GATTAGGGTTAAACCACAACCAAGCTGTGACACGCTTGTTTAAA-GAAACACAAA

AACGAAAGTTACTCTAACCAAACCACTTGACCCCACGACAGCTAGGGCACAAACTGGGAT

TAGGTACCCCACTATGCCTAGCCGTAAAATATTTACCTACACCT-TTAACCGCCCGGGAA

TTACGAGCCCAAGCTTAAAACCCAAAGGACTTGACGGTGCCCCACCCACCTAGAGGAGCC

TGTTCTATAA-TCGATTCTCCCC-GATATACCTCACCACTTCTAGCATA--TCAGCCTGT

ATACCTCCGTCGTAAGCTTACCATATGAACGTC-TACTAGTGAGCCAAATGAT--TTTCA

ATAAATACGTCAGGTCAAGGTGCAGCCCACGAAGTGGTAAGCAATGGGCTACAATTTCTA

CTAA--TAGAACACAACGAAAGACTATGTGCAA-TCTAGTCATGAAGGCGGATTTAGCAG

TAAAAAGAAAATAGAGAGTTCTTTTTAACCTGGCGCTGGGACGCGTACACACCGCCCGTC

ACCCTCTTCAAACGCTTCT-GCAAAG-TTAATAACAAA-ATTATGCACACCAGAAGAGGT

AAGTCGTAACACGGTAAGCGTACTGGAAAGTGCGCTTGGATTAA-CAAAATGTAGCTTAA

CTAAA-GCATCTTGCTTACACCAAGAACATGTCCGTTTAACTCAGATCATTTTGA--GCC

AAAAACTTAGCTCTC---TACCCCT-AATGAAATTTT--TTCAACT--AACAA--CCTTA

TAAAACATTTTACAC-TTCAAGTAAAGGCGATTGAAAAATGTCTA-GAAGCTATAGAAAC

TAGTACCGCAAGGGAAAAGTGAAATAACAGTGAAAA---TTTTAAGCACAAAACAGCAGA

GCCTCAACCTCGTACCTTTTGCATCATGGTTTAACAAGTCTAACCAAGCAAA-CGCACCT

TAAGTTTGACTTCCCGAAACTAGACGAGCTACTTAAGAACAGCCTTGT-GGGCCAACCCG

TCTCTGTTGCAAAAGAGTGGGACGATTTTTTAAGTAGAGGTGATAAACCTACCGAGCCTA

GAGATAGCTGGTTATTCAGGAAAAGGATCTAAGTCCTACCTTAAATTTTAT-GTA-----

--AAGCAG

CCACCTTTT-AAAAAGCGTTAAAGCTTTATTG----TTTACCAAAAAAATACCATAA-AC

CTAACTCAACCCTT-TACTA-ATACTGAATGACTTCATACCTTTATGAAGAACCTTATGT

TAGAACTAGTAACAAGAAGA-AGCCCTTCTCCA-AAATGTAAACATGAACCAAAATGAAC

AAATCATTGGTATTTAAAATTTTTTAGCTACAAGTAACAACTCA----TCAAGAAAACCT

TAC------------------TGCCCAA-CATGTCAACCTTACACAAGTACATTTATGGA

AAGAGTAAAAGAATGGGAAGGAACTCGGCAAATAA-CTAACCCCGCCTGTTTACCAAAAA

CATCGCCTCCTGCTACTTT---ATAGGAGGTCCAGCCTGCCCAGTGATAAA--ATTAAAC

GGCCGCGGTACCCTAACCGTGCAAAGGTAGCGCAATCACTTGTTCTTTAAATGAGGACTA

GTATGAACGGCATCACGAGGGTTACACTGTCTCCCCACTCCACTCAGTGAAACTGATCCC

CCCGTGAAGAAGCGGGGATAAATATATAAGACGAGAAGACCCCATGGAGCTTAAAACTCA

GTCTTACCTGCCATCTA--AAAAACCTAGAAACCAC-GCAG-TACTAAT-GACTAGTTTT

CGGTTGGGGTGACCGCGGAGAAAAACAAATCCTCCACGACGAAAGGACCTATCCACCTAA

CCCAAGAGTTACACCTCTAAGAATCAAC--------ACATTGACCAA--CTGATCCAATT

TTTT--GATCAGCGGACCAAGTTACCCTGGGGATAACAGCGCAATCCATTTCAAGAGCTC

ATATCGACAAATGGGTTTACG-ACC-TCGATGTTGGATCAGGGTATCCAAGTG-GCGCAG

CCGCTA-CTAA-CGGTTC

>M. butleri MN534730, MN534517, MN534621

TTATTATCAGCTACCTCTCGACTTACACATGCAAGTATCAGCACACCCGTGAGAACGCCC

TTCAACCTC-ACCAGGACAAGGAGCTGGTATCAGGCGCAG---ATTT-----TCTAGCCC

ATTACACCTAGCTTTGCCACACCCTCAAGGGAATTCAGCAGTGATTAACATTGTTAATAA

GCGTCAGCTTGACTCAGTCAGAGAACCA--AGAGCCGGCTAATACGGTGCCAGCCGCCGC

GGCTACACCGCTAGGCTCAAGTTGATATT-ACTCGGCGTTAAGCGTGTTTAAAGTGCCT-

TTAA-GATTAGGGTTAAACCACAACCAAGCTGTGACACGCTTGTTTAAA-GAAACACAAA

AACGAAAGTTACTCTAACCAAACCACTTGACCCCACGACAGCTAGGGCACAAACTGGGAT

TAGGTACCCCACTATGCCTAGCCGTAAAATATTTACCCACACCT-TTAACCGCCCGGGAA

TTACGAGCCCAAGCTTAAAACCCAAAGGACTTGACGGTGCCCCACCCACCTAGAGGAGCC

TGTTCTATAA-TCGATTCTCCCC-GATATACCTCACCACTTCTAGCATA--TCAGCCTGT

ATACCTCCGTCGTAAGCTTACCATATGAACGTC-TACTAGTGAGCCAAATGAT--TTTCA

ATAAATACGTCAGGTCAAGGTGCAGCCCACGAAGTGGTAAGCAATGGGCTACAATTTCTA

CTAA--TAGAACACAACGAAAGACTATGTGCAA-TCTAGTCATGAAGGCGGATTTAGCAG

TAAAAAGAAAATAGAGAGTTCTTTTTAACCTGGCGCTGGGACGCGTACACACCGCCCGTC

ACCCTCTTCAAACGCTTCT-GCAAAG-TTAATAACAAA-ATTATGCACACCAGAAGAGGT

AAGTCGTAACACGGTAAGCGTACTGGAAAGTGCGCTTGGATTAA-CAAAATGTAGCTTAA

CTAAA-GCATCTTGCTTACACCAAGAACATGTCCGTTTAACTCAGATCATTTTGA--GCC

AAAAACTTAGCTCTC---TACCTCT-AATGAAATTTT-TTTCAACT--AACAA--CTCTA

TAAAACATTTTACAC-TTCAAGTAAAGGCGATTGAAAAATGTCTA-GAAGCTATAGAAAC

TAGTACCGCAAGGGAAAAGTGAAATAACAGTGAAAA---TTTTAAGCACAAAACAGCAGA

GCCTCAACCTCGTACCTTTTGCATCATGGTTTAACAAGTCTAACCAAGCAAAACGCACCT

TAAGTTTGACTTCCCGAAACTAGACGAGCTACTTAAGAACAGCCTTGTGGGGCCAACCCG

TCTCTGTTGCAAAAGAGTGGGACGATTTTTTAAGTAGAGGTGATAAACCTACCGAGCCTA

GAGATAGCTGGTTATTCAGGAAAAGGATCTAAGTCCTACCTTAAATTTTA----------

--AAGCAG

CCACCTTTT-AAAAAGCGTTAAAGCTTTATTGT---CATACCAAAAAAATACCATAA-AC

CTAACTCAACCCTT-TACTA-ATACTGAATGACTTCATACCTTTATGAAGAACCTTATGT

TAGAACTAGTAACAAGAAGA-AGCCCTTCTCCA-AAATGTAAACATGAACCAAAATGAAC

AAATCATTGGTATTTAAAATTTTTTAGCTACAAGTAACAACTCA----TCAAGAAAACCT

TAC------------------TGCCCAA-CATGTCAACCTTACACAAGTACATTTATGGA

AAGAGTAAAAGAATGGGAAGGAACTCGGCAAATAA-CTAACCCCGCCTGTTTACCAAAAA

CATCGCCTCCTGCTACTTT---ATAGGAGGTCCAGCCTGCCCAGTGATAAA--ATTAAAC

GGCCGCGGTACCCTAACCGTGCAAAGGTAGCGCAATCACTTGTTCTTTAAATGAGGACTA

GTATGAACGGCATCACGAGGGTTACACTGTCTCCCCACTCTACTCAGTGAAACTGATCCC

CCCGTGAAGAAGCGGGGATAAATATATAAGACGAGAAGACCCCATGGAGCTTAAAACTCA

GTCTTACCTGCCATCTA--AAAAACCTAGAAACCAC-GCAG-TACTAAT-GACTAGTTTT

CGGTTGGGGTGACCGCGGAGAAAAACAAATCCTCCACGACGAAAGGACCTATCCACCTAA

CCCAAGAGGTACACCTCTAAGAATCAAC--------ACATTGACCAA--CTGATCCAATT

TTTT--GATCAACGGACCAAGTTACCCTGGGGATAACAGCGCAATCCATTTCAAGAGCTC

ATATCGACAAATGGGTTTACG-ACC-TCGATGTTGGATCAGGGTATCCAAGTG-GCGCAG

CCGCTA-CTAA-CGGTTC

>M. butleri MN534731, MN534518, MN534622

TTATTATCAGCTACCTCTCGACTTACACATGCAAGTATCAGCACACCCGTGAGAACGCCC

TTCAACCTC-ACCAGGACAAGGAGCTGGTATCAGGCGCAG---ATTT-----TCTAGCCC

ATTACACCTAGCTTTGCCACACCCTCAAGGGAATTCAGCAGTGATTAACATTGTTAATAA

GCGTCAGCTTGACTCAGTCAGAGAACCA--AGAGCCGGCTAATACGGTGCCAGCCGCCGC

GGCTACACCGCTAGGCTCAAGTTGATATT-ACTCGGCGTTAAGCGTGTTTAAAGTGCCT-

TTAA-GATTAGGGTTAAACCACAACCAAGCTGTGACACGCTTGTTTAAA-GAAACACAAA

AACGAAAGTTACTCTAACCAAACCACTTGACCCCACGACAGCTAGGGCACAAACTGGGAT

TAGGTACCCCACTATGCCTAGCCGTAAAATATTTACCTACACCT-TTAACCGCCCGGGAA

TTACGAGCCCAAGCTTAAAACCCAAAGGACTTGACGGTGCCCCACCCACCTAGAGGAGCC

TGTTCTATAA-TCGATTCTCCCC-GATATACCTCACCACTTCTAGCATA--TCAGCCTGT

ATACCTCCGTCGTAAGCTTACCATATGAACGTC-TACTAGTGAGCCAAATGAT--TTTCA

ATAAATACGTCAGGTCAAGGTGCAGCCCACGAAGTGGTAAGCAATGGGCTACAATTTCTA

CTAA--TAGAACACAACGAAAGACTATGTGCAA-TCTAGTCATGAAGGCGGATTTAGCAG

TAAAAAGAAAATAGAGAGTTCTTTTTAACCTGGCGCTGGGACGCGTACACACCGCCCGTC

ACCCTCTTCAAACGCTTCT-GCAAAG-TTAATAACAAA-ATTATGCACACCAGAAGAGGT

AAGTCGTAACACGGTAAGCGTACTGGAAAGTGCGCTTGGATTAA-CAAAATGTAGCTTAA

CTAAA-GCATCTTGCTTACACCAAGAACATGTCCGTTTAACTCAGATCATTTTGA--GCC

AAAAACTTAGCTCTC---TACCCCT-AATGAAATTTT--TTCAACT--AACAA--CCTTA

TAAAACATTTTACAC-TTCAAGTAAAGGCGATTGAAAAATGTCTA-GAAGCTATAGAAAC

TAGTACCGCAAGGGAAAAGTGAAATAACAGTGAAAA---TTTTAAGCACAAAACAGCAGA

GCCTCAACCTCGTACCTTTTGCATCATGGTTTAACAAGTCTAACCAAGCAAAACGCACCT

TAAGTTTGACTTCCCGAAACTAGACGAGCTACTTAAGAACAGCCTTGT-GGGCCAACCCG

TCTCTGTTGCAAAAGAGTGGGACGATTTTTTAAGTAGAGGTGATAAACCTACCGAGCCTA

GAGATAGCTGGTTATTCAGGAAAAGGATCTAAGTCCTACCTTAAATTTTA----------

---CCAAAAAAATACCATAA-AC
CTAACTCAACCCTT-TACTA-ATACTGAATGACTTCATACCTTTATGAAGAACCTTATGT
TAGAACTAGTAACAAGAAGA-AGCCCTTCTCCA-AAATGTAAACATGAACCAAAATGAAC
AAATCATTGGTATTTAAAATTTTTTAGCTACAAGTAACAACTCA----TCAAGAAAACCT
TAC------------------TGCCCAA-CATGTCAACCTTACACAAGTACATTTATGGA
AAGAGTAAAAGAATGGGAAGGAACTCGGCAAATAA-CTAACCCCGCCTGTTTACCAAAAA
CATCGCCTCCTGCTACTTT---ATAGGAGGTCCAGCCTGCCCAGTGATAAA--ATTAAAC
GGCCGCGGTACCCTAACCGTGCAAAGGTAGCGCAATCACTTGTTCTTTAAATGAGGACTA
GTATGAACGGCATCACGAGGGTTACACTGTCTCCCCACTCCACTCAGTGAAACTGATCCC
CCCGTGAAGAAGCGGGGATAAATATATAAGACGAGAAGACCCCATGGAGCTTAAAACTCA
GTCTTACCTGCCATCTA--AAAAACCTAGAAACCAC-GCAG-TACTAAT-GACTAGTTTT
CGGTTGGGGTGACCGCGGAGAAAAACAAATCCTCCACGACGAAAGGACCTATCCACCTAA
CCCAAGAGTTACACCTCTAAGAATCAAC--------ACATTGACCAA--CTGATCCAATT
TTT---GATCAGCGGACCAAGTTACCCTGGGGATAACAGCGCAATCCATTTCAAGAGCTC
ATATCGACAAATGGGTTTACG-ACC-TCGATGTTGGATCAGGGTATCCAAGTG-GCGCAG
CCGCTA-CTAA-CGGTTC
>M. butleri MN534732, MN534519, MN534623
TTATTATCAGCTATCTCCCGACTTACACATGCAAGTATCAGCACACCCGTGAGAACGCCC
TTCAACCTC-ACCAGGACAAGGAGCCGGTATCAGGCACAG---ACTT-----TCTAGCCC
ATGACACCTAGCTTTGCCACACCCTCAAGGGAATTCAGCAGTGATTAACATTGTTTATAA
GCGTCAGCTTGACTCAGTCAGAGAACTA--AGAGCCGGCTAATACGGTGCCAGCCGCCGC
GGCTACACCGCTAGGCTCAAGTTGATATT-ACTCGGCGTTAAGCGTGTTTAAAGTGCCC-
TTAA-GATTAGAGTTAAACCACAACCAAGCTGTGACACGCTTGTTTAAA-GAAACACAAA
AACGAAAGTTACTCTAACCAAACCACTTGACCCCACGACAGCTAGGGCACAAACTGGGAT
TAGGTACCCCACTATGCCTAGCCGTAAAATATTTACCTACACCT-TTAACCGCCCGGGAA
TTACGAGCCCAAGCTTAAAACCCAAAGGACTTGACGGTGCCCCACCCACCTAGAGGAGCC
TGTTCTATAA-TCGATTCTCCCC-GATATACCTCACCACTTCTAGCCCA--TCAGCCTGT
ATACCTCCGTCGTAAGCTTACCATATGAACGTC-TACCAGTGAGCCAAATGAT--TTTCA
ATAAATACGTCAGGTCAAGGTGCAGCCCACGAAGTGGTAAGCAATGGGCTACAATTTCTA

TCAA--TAGAACACAACGAAAGACTATGTGAAA-CCTAGTCATGAAGGCGGATTTAGTAG
TAAAAAGAAAATAGAGAGTTCTTTTTAACCTGGCGCTGGGACGCGTACACACCGCCCGTC
ACCCTCTTCAAACGCTTCT-GCAAAG-TTAATAACAAA-ATTATGCACACCAGAAGAGGT
AAGTCGTAACACGGTAAGCGTACTGGAAAGTGCGCTTGGATTAA-CAAAATGTAGCTTAA
CTAAA-GCATCTTGCTTACACCAAGAACATGTCCGTTTAACTCAGATCATTTTGA--GCC
AAAAACTTAGCTCTC---TTCCTCA-AATGAAATTTT-TTTAAACT--AACAA--CTTTA
TAAAACATTTTACAC-TTCAAGTAAAGGCGATTGAAAAATGTCTA-GAAGCTATAGAAAC
CAGTACCGCAAGGGAAAAGTGAAATAATAATGAAAA---TTTTAAGCACAAAACAGCAGA
GCCTAAACCTCGTACCTTTTGCATCATGGTTTAACGAGTCTAACCAAGCAAAACGCACCT
TAAGTTTGACTTCCCGAAACTAGACGAGCTACTTAAGAACAGCCTTAT-GGGCCAACCCG
TCTCTGTTGCAAAAGAGTGGGACGATTTT-TAAGTAGAGGTGACAAACCTACCGAGTTTA
GAGATAGCTGGTTATTCAGGAAAAGGATCTAAGTCCTACCTTAAATTTTCT-GTATC-TT
C-TAATACAC--
--AAGCAG
CCACCTTTT-AAAAAGCGTTAAAGCTTTATTGC----TTGCCAAAAAAATACCACAA-AC
TTAACTTAACCCTT-TAATA-ATACTGAATGACTTCATATTTTTATGAAGAACCTTATGT
TAGAACTAGTAACAAGAAGA-AGCCCTTCTCCA-AAATGTAGGCATGAACCAAAATGAAC
AAATCATTGGTACTTAAAATTTTTTAACTACAAGTAACAACTCA----TCAAGAAAACCT
TAC------------------TACCCAA-CATGTCAACCTTACACAAGTACATTTATGGA
AAGAGTAAAAGAATGGGAAGGAACTCGGCAAATAA-CTAACCCCGCCTGTTTACCAAAAA
CATCGCCTCCTGCTACTTT---ATAGGAGGTCCAGCCTGCCCAGTGATAAA--ATTAAAC
GGCCGCGGTACCCTAACCGTGCAAAGGTAGCGCAATCACTTGTTCTTTAAATGAGGACTA
GTATGAACGGCATCACGAGGGTTACACTGTCTCCCCACTCTACTCAGTGAAACTGATCCC
CCCGTGAAGAAGCGGGGATAAAAATATAAGACGAGAAGACCCCATGGAGCTTAAAACTCA
GTCTTACCTGCCACCTA-AATAAACCTAGAAACCAC-GCAG-TACTAAT-GACTAGTTTT
CGGTTGGGGTGACCGCGGAGAAAAACAAATCCTCCACGACGAAGGGACCTACTCACCTAA
TCCAAGAGTTACACCTCTAAGAATCAAC--------ACATTGACCAA--CTGATCCAATT
TTTT--GATCAACGGACCAAGTTACCCTGGGGATAACAGCGCAATCCATTTCAAGAGCTC
ATATCGACAAATGGGTTTACG-ACC-TCGATGTTGGATCAGGGTATCCAAGTG-GCGCAG
CCGCTA-CTAA-CGGTTC

>M. butleri MN534733, MN534520, MN534624

TTATTATCAGCTACCTCCCGACTTACACATGCAAGTATCAGCACACCCGTGAGAACGCCC

TTCAACCTC-ACCAGGACAAGGAGCCGGTATCAGGCACAG---ACTT-----TCTAGCCC

ATGACACCTAGCTTTGCCACACCCTCAAGGGAATTCAGCAGTGATTAACATTGTTTATAA

GCGTCAGCTTGACTCAGTCAGAGAACTA--AGAGCCGGCTAATACGGTGCCAGCCGCCGC

GGCTACACCGCTAGGCTCAAGTTGATATT-ACTCGGCGTTAAGCGTGTTTAAAGTGCCT-

TTAA-GATTAGAGTTAAACCACAACCAAGCTGTGACACGCTTGTTTAAA-GAAACACAAA

AACGAAAGTTACTCTAACCAAACCACTTGACCCCACGACAGCTAGGGCACAAACTGGGAT

TAGGTACCCCACTATGCCTAGCCGTAAAATATTTACCTACACCT-TTAACCGCCCGGGAA

TTACGAGCCCAAGCTTAAAACCCAAAGGACTTGACGGTGCCCCACCCACCTAGAGGAGCC

TGTTCTATAA-TCGATTCTCCCC-GATATACCTCACCACTTCTAGCCCA--TCAGCCTGT

ATACCTCCGTCGTAAGCTTACCATATGAACGTC-TACCAGTGAGCCAAATGAT--TTTCA

ATAAATACGTCAGGTCAAGGTGCAGCCCACGAAGTGGTAAGCAATGGGCTACAATTTCTA

TCAA--TAGAACACAACGAAAGACTATGTGAAA-CCTAGTCATGAAGGCGGATTTAGTAG

TAAAAAGAAAATAGAGAGTTCTTTTTAACCTGGCGCTGGGACGCGTACACACCGCCCGTC

ACCCTCTTCAAACGCTTCT-GCAAAG-TTAATAACAAA-ATTATGCACACCAGAAGAGGT

AAGTCGTAACACGGTAAGCGTACTGGAAAGTGCGCTTGGATTAA-CAAAATGTAGCTTAA

CTAAA-GCATCTTGCTTACACCAAGAACATGTCCGTTTAACTCAGATCATTTTGA--GCC

AAAAACTTAGCTCTC---TTCCTCA-AATGAAATTTT-TTTAAACT--AACAA--CTTTA

TAAAACATTTTACAC-TTCAAGTAAAGGCGATTGAAAAATGTCTA-GAAGCTATAGAAAC

CAGTACCGCAAGGGAAAAGTGAAATAATAATGAAAA---CTTTAAGCACAAAACAGCAGA

GCCTAAACCTCGTACCTTTTGCATCATGGTTTAACAAGTCTAACCAAGCAAAACGCACCT

TAAGTTTGACTTCCCGAAACTAGACGAGCTACTTAAGAACAGCCTTAT-GGGCCAACCCG

TCTCTGTTGCAAAAGAGTGGGACGATTTTTTAAGTAGAGGTGATAAACCTACCGAGTTTA

GAGATAGCTGGTTATTCAGGAAAAGGATCTAAGTCCTACCTTAAATTTT-----------

--AAGCAG

CCACCTTTT-AAAAAGCGTTAAAGCTTTATTGT----TTACCAAAAAAATACCACAA-AC

TTAACTTAACCCTC-TACTA-ATACTGAATGACTTCATATCTTTATGAAGAACCTTATGT

TAGAACTAGTAACAAGAAGA-AGCCCTTCTCCA-AAATGTAAGCATGAACCAAAATGAAC

AAATCATTGGTGCTTAAAATTTTTTAACTACAAGTAACAACTCA----TCAAGAAAATCT

TAC------------------TACCCAA-CATGTCAACCTTACACAAGTACATTTATGGA

AAGAGTAAAAGAATGGGAAGGAACTCGGCAAATAA-CTAACCCCGCCTGTTTACCAAAAA

CATCGCCTCCTGCTACTTT---ATAGGAGGTCCAGCCTGCCCAGTGATAAA--ATTAAAC

GGCCGCGGTACCCTAACCGTGCAAAGGTAGCGCAATCACTTGTTCTTTAAATGAGGACTA

GTATGAACGGCATCACGAGGGTTACACTGTCTCCCCGCTCTACTCAGTGAAACTGATCTC

CCCGTGAAGAAGCGGGGATAAAAATATAAGACGAGAAGACCCCATGGAGCTTAAAACTCA

GTCTTACCTGCCACCTA--AAAAACCTAGAAACCAC-GCAG-TACTAAT-GACTAGTTTT

CGGTTGGGGTGACCGCGGAGAAAAACAAATCCTCCACGACGAAGGGACCTCCTCACCTAA

TCCAAGAGTTACACCTCTAAGAATCAAC--------ACATTGACCAA--CTGATCCAATT

TTTT--GATCAACGGACCAAGTTACCCTGGGGATAACAGCGCAATCCATTTCAAGAGCTC

ATATCGACAAATGGGTTTACG-ACC-TCGATGTTGGATCAGGGTATCCAAGTG-GCGCAG

CCGCTA-CTAA-CGGTTC

>M. butleri MN534734, MN534521, MN534625

TTATTATCAACTACCTCTCGACTTACACATGCAAGTATCAGCACACCCGTGAGAACGCCC

TTCAACCTC-ACCAGGACAAGGAGCTGGTATCAGGCACAG---ATTT-----TCTAGCCC

ATAACACCTAGCTTTGCCACACCCTCAAGGGAATTCAGCAGTGATTAACATTGTTTATAA

GCGTAAGCTTGACTCAGTCAGAGAACTA--AGAGCCGGCTAATACGGTGCCAGCCGCCGC

GGCTACACCGCTAGGCTCAAGTTGATACT-ACTCGGCGTTAAGCGTGTTTAAAGTGCCT-

CTAA-GATTAGAGTTAAACCACAACCAAGCTGTGACACGCTTGTTTAAA-GAAGCACAAA

AACGAAAGTTACTCTAACCAAACCACTTGACCCCACGACAGCTAGGGTACAAACTGGGAT

TAGGTACCCCACTATGCCTAGCCGTAAAATATTTACCTACACCT-TTAACCGCCCGGGAA

TTACGAGCCCAAGCTTAAAACCCAAAGGACTTGACGGTGCCCCACCCACCTAGAGGAGCC

TGTTCTATAA-TCGATTCTCCCC-GATATACCTCACCACTTCTAGCNNN--TCAGCCTGT

ATACCTCCGTCGTAAGCTTACCCTATGAACGTC-TACCAGTGAGCCAAATGAT--TTTCA

ATAAATACGTCAGGTCAAGGTGCAGCCCACGAAGTGGCAAGAAATGGGCTACAATTTCTA

CTAA--TAGAACATAACGAAAGACTACGTGCAA-TCTAGTCATGAAGGCGGATTTAGCAG

TAAAAAGAAAATAGAGAGTTCTTTTTAACTTGGCGCTGGGACGCGTACACACCGCCCGTC

ACCCTCTTCAAACGCTTCT-GCAAAG-TTAATAACAAA-ATTATGCACACCAGAAGAGGT

AAGTCGTAACACGGTAAGCGTACTGGAAAGTGCGCTTGGATTAA-CAAAATGTAGCTTAA

TTAAA-GCATCTTGCTTACACCAAGAACATGTCCGTTTAACTCAGATCATTTTGA--GCC

AAAAACTTAGCTCTA---TACCCCC-AATGAAAATTT--TCTAACT--AACAA--CCCCA

TAAAACATTTTACAC-TTCAAGTAGAGGCGATTGAAAAATGTCTA-GAAGCTATAGAAAC

CAGTACCGCAAGGGAAAAGTGAAATAACAGTGAAAA---CTTAAAGCACAAAACAGCAGA

GACTTAACCTCGTACCTTTTGCATCATGGTTTAACGAGTCTAACCAAGCAAAACGCACCT

TAAGTTTGACTTCCCGAAACTAGACGAGCTACTTAAGAACAGCCTTAT-GGGCCAACCCG

TCTCTGTTGCAAAAGAGTGGGACGATTTT-TAAGTAGAGGTGATAAACCTACCGAGTCTA

GAGATAGCTGGTTATTCAGGAAAAGGATCTAAGTCCTACCTTAAATTTTTT-GTATT-CT

C-TAATACACCCTA-AAATTTACA--CTATTCAAATAAGGTACAGCCTATT-GAGAAAGG

ATACAACCTCAAAT---------------------------------------AAAGCAG

CCACCTTTTTAAAAAGCGTTAAAGCTTTATTGT----TTATCAAAAAAATATCATAA-AC

TTAACTTAACCCTT-CACTA-ATACTGAATGACTTCATATCTTTATGAAGAACCTTATGT

TAGAACTAGTAACAAGAAGA-AGCCCTTCTCCA-AAATATAAGCATGAACCAAAATGAAC

AAATCATTGGTACTTAAAATTTTTTAACTTCAAGTAACAACCCA----TCAAGAAAACCT

TAC------------------TACCTAA-CATGTCAACCTTACACAAGTATATTTATGGA

AAGAGTAAAAGAATGGGAAGGAACTCGGCAAATTA-CTAACCCCGCCTGTTTACCAAAAA

CATCGCCTCCTGCTACTTT---ATAGGAGGTCCAGCCTGCCCAGTGATAAA--ATTAAAC

GGCCGCGGTACCCTAACCGTGCAAAGGTAGCGCAATCACTTGTTCTTTAAATGAGGACTA

GTATGAACGGCATCACGAGGGTTACACTGTCTCCCCACTCTACTCAGTGAAACTGATCTC

CCCGTGAAGAAGCGGGGATAAAAATATAAGACGAGAAGACCCCATGGAGCTTAAAACTCA

GTCTTACCTGCCACCTA--AAAAACCTAGAAACCAC-GCAG-TACTAAT-GACTAGTTTT

CGGTTGGGGTGACCGCGGAGAAAAACAAATCCTCCACGACGAAAGGACCTATTTACCTAA

TCCAAGAGCTACACCTCTAAGAATCAAC--------ACATTGACCAA--CTGATCCAATT

TTT---GATCAGCGGACCAAGTTACCCTGGGGATAACAGCGCAATCCATTTCAAGAGCTC

ATATCGACAAATGGGTTTACG-ACC-TCGATGTTGGATCAGGGTATCCAAGTG-GCGCAG

CCGCTA-CTAA-CGGTTC

>M. butleri MN534735, MN534626

TTATTATCAGCTACCTCTCGACTTACACATGCAAGTATCAGCACACCCGTGAAAACGCCC

TTCAACCTC-CCCAGGACAAGGAGCCGGTATCAGGCACAG---ATTT-----TCTAGCCC

ATAACACCTAGCTTTGCCACACCCTCAAGGGAATTCAGCAGTGATTAACATTGTTAATAA

GCGTCAGCTTGACTCAGTCAGAGAACTA--AGAGCCGGCTAATACGGTGCCAGCCGCCGC

GGCTACACCGCTAGGCTCAAGTTGATACT-ACTCGGCGTTAAGCGTGTTTAAAGTGCCT-

TTAA-GATTAGAATTAAACCACAACCAAGCTGTGACACGCTTGTTACAA-GAAATACAAA

AACGAAAGTTATTCTAACCAAACCACTTGACTCCACGACAGCTAGGGCACAAACTGGGAT

TAGGTACCCCACTATGCCTAGCCGTAAAATATTTACCTACACCT-CTAACCGCCCGGGAA

TTACGAGCCCAAGCTTAAAACCCAAAGGACTTGACGGTGCCCCACCCACCTAGAGGAGCC

TGTTCTATAA-TCGATTCTCCCC-GATCTACCTCACCACTTTTAGCCCA--TCAGCCTGT

ATACCTCCGTCGTAAGCTTACCATATGAACGCC-CATCAGTGAGCCAAATGAT-TCTTCA

ATAAATACGTCAGGTCAAGGTGCAGCCTACGAAGTGGTAAGCAATGGGCTACAATTTCTA

CCAA--TAGAACATA-CGAAAGACTACGTGCAA-CCTAGTCATGAAGGCGGATTTAGCAG

TAAAAAGAAAATAGAGAGTTCTTTTTAACCTGGCGCTGGGACGCGTACACACCGCCCGTC

ACCCTCTTCAAACGCTTCT-GCAAAG-TTAATAACAAG-ATTATGCACACCAGAAGAGGT

AAGTCGTAACACGGTAAGCGTACTGGAAAGTGCGCTTGGATTAA-CAAAATGTAGCTTAC

CTAAA-GCATCTTGCTTACACCAAGAACATGTCCGTTTAACTCAGATCATTTTGA--GCC

AAAAACTTAGCTCTC---TACCT-------------------------------------

---AAAGCAG

CCACCTTTTAAAAAAGCGTTAAAGCTTCATTGT----TTACTAAAAAAATTTCATAA-AC

CTAATATAACCCTT-CACCT-CTACTGAATGACTTCATAACTTTATGAAGAACCTTATGT

TAAAACTAGTAACAAGAAGT-AGCCCTTCTCCA-AAATGTAAGCATGAACCAAAATGAAC

AATTCATTGGTATTTAAAATTTTTTAGCCTCAAGTAATAACCCA----CCAAGAAAATCT

TAC------------------TGCCTAA-CATGTCAACCTTACACCAGCACATTCATGGA

AAGAGTAAAAGAATGGGAAGGAACTCGGCAAATAA-CTAACCCCGCCTGTTTACCAAAAA

CATCGCCTCCTGCTACTTT---ATAGGAGGTCCAGCCTGCCCAGTGATATA--ATTAAAC

GGCCGCGGTACCCTAACCGTGCAAAGGTAGCGCAATCACTTGTTCTTTAAATGAGGACTA
GTATGAACGGCATCACGAGGGTTACACTGTCTCCCCACTCTACTCAGTGAAACTGATCTC
CCCGTGAAGAAGCGGGGATAAAAATATAAGACGAGAAGACCCCATGGAGCTTAAAACTCA
GTCTTACCTGCCACCTA--AAAAACCTAGAAACCAC-GCAA-TACTAAT-GACTAGTTTT
CGGTTGGGGTGACCGCGGAGAAAAACAAATCCTCCACGACGAAAGGACCTATT-ACCTAA
TCTAAGAGCCACTCCTCTAAGAATCAAC--------ACATTGACTAA--CTGATCCAATT
TTTT--GATCAACGAACCAAGTTACCCTGGGGATAACAGCGCAATCCATTTCAAGAGCTC
ATATCGACAAATGGGTTTACG-ACC-TCGATGTTGGATCAGGGTATCCAAGTG-GCGCAG
CCGCTA-CTAA-CGGTTC

>M. zeylanica MN534736, MN534522, MN534627

TTATTATCAACTCGATCTCAACTTACACATGCAAGTATCAGCACACCCGTGAGAACGCCC
TTC-ACCTT-AATAGGACAAGGAGCTGGTATCAGGCACAGGTCCTTACAACCCCTAGCCC
ATAACACCTAGCTTTGCCACACCCTCAAGGGTACTCAGCAGTGATCAATATTGTATATAA
GCGCCAGCTTGACTCAGTAAGAGAATTT--AGAGCCGGCCAATACGGTGCCAGCCGCCGC
GGCTACACCGCTAGGCTCAAGTTGATAAT-ACTCGGCGTTAAGCGTGTTTAAAGTTATA-
--AA-GATTAGGGTTAAACTCTAACCAAGTTGTGACACACTTGTTTCTTAGAAATCCA-A
AACGAAAGTTATCCTAACCAGACCACTTGAACCCACGACAGCTAGGGCACAAACTGGGAT
TAGGTACCCCACTATGCCTAGCCGTAAAATATTTACTCACACAA-CCAACCGCCGGGGTA
TTACGAGCCCAAGCTTAAAACCCAAAGGACTTGACGGTGTCCCACCCACCTAGAGGAGCC
TGTTCTATAA-TCGATTCCCCCC-GATTCACCTCACCCCTTCTAGCCTA--TCAGCCTGT
ATACCTCCGTCGTAAGCTTACCATATGAACGAT---TTAGTGAGCCAAAAGAT--TTTCC
GTAAATACGTCAGGTCAAGGTGCAGCCCACGAAGTGGCAAGAAATGGGCTACAATTTCTA
TAA---TAGAACACAACGAAAGACTACATGCAA-CAAAGTCATGAAGGCGGATTTAGTAG
TAAAAAGGAACTAGAGAGTCCATTTTAATTAGGCGCTGGGACGCGTACACACCGCCCGTC
ACCCTCTTCAAATGCCTAA-GATTAG-TTAATAACGAACCATATGCAAAACAGAAGAGGT
AAGTCGTAACACGGTAAGCGTACTGGAAAGTGTGCTTGGATAAA-CAAAATGTAGTTTAA
TTAAA-GCATTTCGCTTACACCGAAAACATGTCTGTTTAAATCAGATCATTTTGA--GCC
AAAAACTTAGATCCA--TCACCCGT-AATGGCCACC--CATCAACTCATTTAA--TAAAA
TAAAACATTTTATCT-TTATAGTAAAGGCGATTAAAAAATATCTT-GAAGCTATAGAAAC
TAGTACCGCAAGGGAATAGTGAAATAAAGATGAAAA--CCTTTAAGCACCAAAAAGCAGA

GCCACAACCTCGTACCTTTTGCATCATGGTTTAACTAGTCTAATCAGACAAAGCGCACTT

TAAGCCTGACCCCCCGAAACTAGGTGAGGTACTTAAAAACAGCCTTT-AGAGCCAACCCG

TCTCTGTTGCAAAAGAGTGGGAAGATTAT-TAAGTAGAGGTGATAGACCTACCGAACCTA

GAGATAGCTGGTTATTCAGGAAAAGAACTTAAGTCCTACCTTAGGTTTATT-ATAAT-AT

--GAGTATTCAACA-AACCTTAAGAGCTATTCAAACAAGGTACAGCCTGCTTGAAAAAGG

ATACAACCTCAACTAG---------------------------------------AGCAG

CCACCTTTT-AAAAAGCGTTAAAGCTTCATTA---CTAGTGCAAAAAAA-TTTCATTAAC

CAACTAAAACCCCTAAACCC-CTACTGAATGAATTCATAATTTTATGAAAACCTATCTGT

TAAAACTAGTAACAAGAAGA-AGACCTTCTCCA-AAATGCAAGTATAAGCCGCAATGAAC

AAGTCATCGGCACTTAAAGCCTGTAATCTACCAGTAGCAACCCA----CCAAGAAAACCC

TAC------------------TGATTAC-AGCGTTAACCTTACACAAGCACATTTTTGGA

AAGATTAAAAAAACAGGAAGGAACTCGGCAAATTT-TTAACCCCGCCTGTTTACCAAAAA

CATCGCCTCTTGGAATCCC---ATAAGAGGTCCAGCCTGCCCAGTGACAAA--GTTAAAC

GGCCGCGGTACCCTAACCGTGCAAAGGTAGCGCAATCACTTGTTCTTTAAATGAGGACTA

GTATGAATGGCATCACGAGGGTTAAGCTGTCTCCCTGCTTTAATCAGTAAAACTGATTTT

CCCGTGAAGAAGCGGGAATAAACCTATAAGACGAGAAGACCCCATGGAGCTTAAAACTTA

GTATCACCTGCTACATA--CACATCTAATAAAAAAT-GCAA-CGCTGAT-TTCTAGTTTT

CGGTTGGGGTGACCACGGAGTAAAATTAAACCTCCGCAACGAAAGGACCTAGCTTCCTAA

CCTAAGAGCTACAGCTCTAAGAATCAAC--------AAATTGACTAAA-CTGACCCAATT

AATT--GATCAATGAACCAAGTTACCCTGGGGATAACAGCGCAATCCATTTCAAGAGCCC

TTATCGACAAATGGGTTTACG-ACC-TCGATGTTGGATCAGGGCATCCAAGTG-GCGCAG

CCGCTA-CTAA-CGGTTC

>M. zeylanica MN534737, MN534523, MN534628

TTATTATCAACTCGATCTCAACTTACACATGCAAGTATCAGCACACCCGTGAGAACGCCC

TTC-ACCTT-AATAGGACAAGGAGCTGGTATCAGGCACAGGTCCTTACAACCCCTAGCCC

ATAACACCTAGCTTTGCCACACCCTCAAGGGTACTCAGCAGTGATCAATATTGTATATAA

GCGCCAGCTTGACTCAGTAAGAGAATTT--AGAGCCGGCCAATACGGTGCCAGCCGCCGC

GGCTACACCGCTAGGCTCAAGTTGATAAT-ACTCGGCGTTAAGCGTGTTTAAAGTTATA-

--AA-GATTAGGGTTAAACTCTAACCAAGTTGTGACACACTTGTTTCTTAGAAATCCA-A

AACGAAAGTTATCCTAACCAGACCACTTGAACCCACGACAGCTAGGGCACAAACTGGGAT

TAGGTACCCCACTATGCCTAGCCGTAAAATATTTACTCACACAA-CCAACCGCCGGGGTA
TTACGAGCCCAAGCTTAAAACCCAAAGGACTTGACGGTGTCCCACCCACCTAGAGGAGCC
TGTTCTATAA-TCGATTCCCCCC-GATTCACCTCACCCCTTCTAGCCTA--TCAGCCTGT
ATACCTCCGTCGTAAGCTTACCATATGAACGAT---TTAGTGAGCCAAAAGAT--TTTCC
GTAAATACGTCAGGTCAAGGTGCAGCCCACGAAGTGGCAAGAAATGGGCTACAATTTCTA
TAA---TAGAACACAACGAAAGACTACATGCAA-CAAAGTCATGAAGGCGGATTTAGTAG
TAAAAAGGAACTAGAGAGTCCATTTTAATTAGGCGCTGGGACGCGTACACACCGCCCGTC
ACCCTCTTCAAATGCCTAA-GATTAG-TTAATAACGAACCATATGCAAAACAGAAGAGGT
AAGTCGTAACACGGTAAGCGTACTGGAAAGTGTGCTTGGATAAA-CAAAATGTAGCTTAA
TTAAA-GCATTTCGCTTACACCGAAAACATGTCTGTTTAAATCAGATCATTTTGA--GCC
AAAAACTTAGCTCCA--TCACCCCT-AATGCCCACC--CATCAACTCATTTAA--TAAAA
TAAAACATTTTATCT-TTATAGTAAAGGCGATTAAAAAATATCTT-GAAGCTATAGAAAC
TAGTACCGCAAGGGAATAGTGAAATAAAGATGAAAA--CCTTTAAGCACCAAAAAGCAGA
GCCACAACCTCGTACCTTTTGCATCATGGTTTAACTAGTCTAATCAGACAAAGCGCACTT
TAAGCCTGACCCCCCGAAACTAGGTGAGCTACTTAAAAACAGCCTTT-AGAGCCAACCCG
TCTCTGTTGCAAAAGAGTGGGAAGATTAT-TAAGTAGAGGTGATAGACCTACCGAACCTA
GAGATAGCTGGTTATTCAGGAAAAGAACTTAAGTCCTACCTTAGGTTTATT-ATAAT-AT
--GAGTATTCAACA-AACCTTAAGAGCTATTCAAACAAGGTACAGCCTGCTTGAAAAAGG
ATACAACCTCAACTAG---------------------------------------AGCAG
CCACCTTTTAAAAAAGCGTTAAAGCTTCATTA---CTAGTGCAAAAAA--TTTCATTAAC
CAACTAAAACCCCTAAACCC-CTACTGAATGAATTCATAATTTTATGAAAACCTATCTGT
TAAAACTAGTAACAAGAAGA-AGACCTTCTCCA-AAATGCAAGTATAAGCCGCAATGAAC
AAGTCATCGGCACTTAAAGCCTGTAATCTACCAGTAGCAACCCA----CCAAGAAAACCC
TAC------------------TGATTAC-AGCGTTAACCTTACACAAGCACATTTTTGGA
AAGATTAAAAAAACAGGAAGGAACTCGGCAAATTT-TTAACCCCGCCTGTTTACCAAAAA
CATCGCCTCTTGGAATCCC---ATAAGAGGTCCAGCCTGCCCAGTGACAAA--GTTAAAC
GGCCGCGGTACCCTAACCGTGCAAAGGTAGCGCAATCACTTGTTCTTTAAATGAGGACTA
GTATGAATGGCATCACGAGGGTTAAGCTGTCTCCCTGCTTTAATCAGTAAAACTGATTTT
CCCGTGAAGAAGCGGGAATAAACCTATAAGACGAGAAGACCCCATGGAGCTTAAAACTTA
GTATCACCTGCTACATA--CACATCTAATAAAAAAT-GCAA-CGCTGAT-TTCTAGTTTT

CGGTTGGGGTGACCACGGAGTAAAATTAAACCTCCGCAACGAAAGGACCTAGCTTCCTAA

CCTAAGAGCTACAGCTCTAAGAATCAAC--------AAATTGACTAAA-CTGACCCAATT

AATT--GATCAATGAACCAAGTTACCCTGGGGATAACAGCGCAATCCATTTCAAGAGCCC

TTATCGACAAATGGGTTTACG-ACC-TCGATGTTGGATCAGGGCATCCAAGTG-GCGCAG

CCGCTA-CTAA-CGGTTC

>M. karunaratnei MN534738, MN534524, MN534629

TTATTATCAACCTAATCTCAACTTACACATGCAAGTATCAGCATACCCGTGAGAACGCCC

TTTTACCTT-AATAGGACAAGGAGCCGGTATCAGGCACAGGTCCTCACAAC-CCTAGCCC

ACGACACCTAGCTTTGCCACACCCTCAAGGGTACTCAGCAGTGATCAATATTGAATATAA

GCGCCAGCTTGACTCAGTTAGAGAATTT--AGAGCCGGCCAATACGGTGCCAGCCGCCGC

GGCTATACCGCTAGGCTCAAGTTGACAGT-ATTCGGCGTTAAGCGTGTTTAAAGTTCTA-

-AAA-GATTAGGGTTAAAATCCAACCAAGTTGTGACACACTTGTTCCTTAGAAAATCA-A

AACGAAAGTTACCCTAACCAGACCACTTGAACCCACGACAGCTAGGACACAAACTGGGAT

TAGGTACCCCACTATGCCTAGCCGTAAAATATTTACTCACACAA-CCAATCGCCTGGGTA

TTACGAGCTCAAGCTTAAAACCCAAAGGACTTGACGGTGTCCCACCCACCTAGAGGAGCC

TGTTCTAAAA-TCGATTCCCCCC-GATCTACCTCACCACTTCTTGCTTA--TCAGCCTGT

ATACCTCCGTCGTAAGCTTACCATATGAACGAT---CTAGTGAGCTAAAGGAT--TTTAC

ATAAATACGTCAGGTCAAGGTGCAGCCCACGAAGCGGTAAGAAATGGGCTATTTTTTGTA

TAA---TAGAACACAACGAAAGACTACATGCAA-CAAAGTCATGAAGGCGGATTTAGTAG

TAAAAAGGAACTAGAGAGTCCATTTTAATTAGGCGCTGGGACGCGTACACACCGCCCGTC

ACCCTCTTCAAATGCCTAA-AATTAG-TTAATAACAAACTGCATGCAAAACAGAAGAGGT

AAGTCGTAACACGGTAAGCGTACTGGAAAGTGTGCTTGGATAAA-CAATATGTAGCTTAA

TTAAA-GCATTTCGCTTACACCGAAAACATGTCTGTGTAAATCAGATCATCTTGA--GCC

AAAAACTTAGCTCCA--CCACCCCT-AATGCCCACT--CACCAACTTGTAAAA--TAAAA

TAAAACATTTTACCT-ATTTAGTAAAGGCGATTAAAAAATAACTA-AAAGCTATAGAAAC

TAGTACCGCAAGGGAATAGTGAAATAAAAATGAAAA--CCTTAAAGCATAAAAAAGCAGA

GCCACAACCTCGTACCTTTTGCATCATGGTTTAACTAGTCTAATCAGACAAAGCGCACTT

TAAGCCTGACCCCCCGAAACTAGGTGAGCTACTTAAAAACAGCCTTT-AGGGCCAACCCG

TCTCTGTTGCAAAAGAGTGGGAAGATTAT-TAAGTAGAGGTGACAGACCTACCGAACCTA

GAGATAGCTGGTTATTCAGGAAAAGAACTTAAGTCCTACCTTAGGTTTATT-TTAAT-AC

--AAATATTCAACA-AACCTTAAGAGCTATTCAAACAAGGTACAGCCTGTTTGAAATAGG
ATACAACCTAAACTA-----------------------TATAAAGTGGGCCTAAAAGCAG
CCACCTTCC-AAAAAGCGTTAAAGCTTCATTA---CCCATGCAAAAAA--TCTCATTAAC
CAACTAAAACCCCTAACCTA-CTACTGAATGATTTCATAATCATATGAAAACCTATCTGT
TAAAACTAGTAACAAGAAGA-AGACCTTCTCCA-AAATGCAAGTATAAGCCGCAATGAAC
AAAACATCGGCATTTAAAGCCTGTAATTTACCAGTAGCAACTCA----CCAAGAAAACCC
TAC------------------TGATTAC-AACGTTAACCTTACACCAGCACATTTATGGA
AAGATTAAAAAAACAGGAAGGAACTCGGCAAACTA-TAAACCCCGCCTGTTTACCAAAAA
CATCGCCTCTTGAAACCAC---ATAAGAGGTCCAGCCTGCCCAGTGACAAA--GTTAAAC
GGCCGCGGTACCCTAACCGTGCAAAGGTAGCACAATCACTTGTTCTTTAAATGAGGACTA
GTATGAACGGCATCACGAGGGTTAAGCTGTCTCCCTGCTTTAATCAGTTAAACTGATCTT
CCCGTGAAGAAGCGGGAATAAACTTATAAGACGAGAAGACCCCATGGAGCTTAAAACTCA
GTATCACCTGCTATTTTA-TAAACCCAATAAACAAT-GCAA-TACTGAT-TCCTAGTTTT
CGGTTGGGGTGACCACGGAGTAAAACAAAACCTCCGCAACGAAAGGACCTAATTACCTAA
TCCAAGAGCTACAACTCTAAGAATCAAT--------AAATTGACTAAA-CTGACCCAATT
ATTT--GATCAATGAACCAAGTTACCCTGGGGATAACAGCGCAATCCATTTCAAGAGCCC
CTATCGACAAATGGGTTTACG-ACC-TCGATGTTGGATCAGGACATCCAAGTG-GCGCAG
CCGCTA-CTAA-AGGTTC
>M. karunaratnei MN534739, MN534525, MN534630
TTATTATCAACCTAATCTCAACTTACACATGCAAGTATCAGCATACCCGTGAGAACGCCC
TTTTACCTT-AATAGGACAAGGAGCCGGTATCAGGCACAGGTCCTCACAAC-CCTAGCCC
ACGACACCTAGCTTTGCCACACCCTCAAGGGTACTCAGCAGTGATCAATATTGAATATAA
GCGCCAGCTTGACTCAGTTAGAGAATTT--AGAGCCGGCCAATACGGTGCCAGCCGCCGC
GGCTATACCGCTAGGCTCAAGTTGACAGT-ATTCGGCGTTAAGCGTGTTTAAAGTTCTA-
-AAA-GATTAGGGTTAAACTCCAACCAAGTTGTGACACACTTGTTCCTTAGAAAATCA-A
AACGAAAGTTACCCTAACCAGACCACTTGAACCCACGACAGCTAGGACACAAACTGGGAT
TAGGTACCCCACTATGCCTAGCCGTAAAATATTTACTTACACAA-CCAATCGCCTGGGTA
TTACGAGCTCAAGCTTAAAACCCAAAGGACTTGACGGTGTCCCACCCACCTAGAGGAGCC
TGTTCTAAAA-TCGATTCCCCCC-GATCTACCTCACCACTTCTTGCTTA--TCAGCCTGT
ATACCTCCGTCGTAAGCTTACCATATGAACGAT---CTAGTGAGCTAAAGGAT--TTTAC

ATAAATACGTCAGGTCAAGGTGCAGCCCACGAAGCGGTAAGAAATGGGCTACAATTTCTA

TAA---TAGAACACAACGAAAGACTACATGCAA-CAAAGTCATGAAGGCGGATTTAGTAG

TAAAAAGGAACTAGAGAGTCCATTTTAATTAGGCGCTGGGACGCGTACACACCGCCCGTC

ACCCTCTTCAAATGCCTAA-AATTAG-TTAATAACAAAATGCATGCAAAACAGAAGAGGT

AAGTCGTAACACGGTAAGCGTACTGGAAAGTGTGCTTGGATAAA-CAATATGTAGCTTAA

TTAAA-GCATTTCGCTTACACCGAAAACATGTCTGTGTAAATCAGACCATCTTGA--GCC

AAAAACTTAGCTCCA--CCACCCCT-AATGCCCACT--CACCAACTTGTAAAA--TAAAA

TAAAACATTTTACCT-ATTTAGTAAAGGCGATTAAAAAATAACTA-AAAGCTATAGAAAC

TAGTACCGCAAGGGAATAGTGAAATAAAAATGAAAA--CCTTAAAGCATCAAAAAGCAGA

GCCACAACCTCGTACCTTTTGCATCATGGTTTAACTAGTCTAATCAGACAAAGCGCACTT

TAAGCCTGACCCCCCGAAACTAGGTGAGCTACTTAAAAACAGCCTTT-AGGGCCAACCCG

TCTCTGTTGCAAAAGAGTGGGAAGATTAT-TAAGTAGAGGTGACAGACCTACCGAACCTA

GAGATAGCTGGTTATTCAGGAAAAGAACTTAAGTCCTACCTTAGGTTTATT-TTAAT-AC

--AAATATTCAACA-AACCTTAAGAGCTATTCAAACAAGGTACAGCCTGTTTGAAATAGG

ATACAACCTAAACTA----------------------TATAAAGTGGGCCTAAAAGCAG

CCACCTTCC-AAAAAGCGTTAAAGCTTCATTA---CCCATGCAAAAAA--TCTCATTAAC

CAACTAAAACCCCTAACCTA-CTACTGAATGATTTCATAATCATATGAAAACCTATCTGT

TAAAACTAGTAACAAGAAGA-AGACCTTCTCCA-AAATGCAAGTATAAGCCGCAATGAAC

AAAACATCGGCATTTAAAGCCTGTAATTTACCAGTAGCAACTCA----CCAAGAAAACCC

TAC------------------TGATTAC-AACGTTAACCTTACACCAGCACATTTATGGA

AAGATTAAAAAAACAGGAAGGAACTCGGCAAACTA-TAAACCCCGCCTGTTTACCAAAAA

CATCGCCTCTTGAAACCAC---ATAAGAGGTCCAGCCTGCCCAGTGACAAA--GTTAAAC

GGCCGCGGTACCCTAACCGTGCAAAGGTAGCGCAATCACTTGTTCTTTAAATGAGGACTA

GTATGAACGGCATCACGAGGGTTAAGCTGTCTCCCTGCTTTAATCAGTTAAACTGATCTT

CCCGTGAAGAAGCGGGAATAAACTTATAAGACGAGAAGACCCCATGGAGCTTAAAACTCA

GTATCACCTGCTATTTTA-TAAACCCAATAAACAAT-GCAA-TACTGAT-TCCTAGTTTT

CGGTTGGGGTGACCACGGAGTAAAACAAAACCTCCGCAACGAAAGGACCTAATTACCTAA

TCCAAGAGCTACAACTCTAAGAATCAAT--------AAATTGACTAAA-CTGACCCAATT

ATTT--GATCAATGAACCAAGTTACCCTGGGGATAACAGCGCAATCCATTTCAAGAGCCC

CTATCGACAAATGGGTTTACG-ACC-TCGATGTTGGATCAGGGCATCCAAGTG-GCGCAG

CCGCTA-CTAA-AGGTTC

>Microhyla sp. 2 MN534740, MN534526, MN534631

TTATTATCAACTCATCCTCAATTTACACATGCAAGTATCAGCACACCCGTGAGAACGCCC

TTC-ACCTT-TTCAGGACAAGGAGCTGGTATCAGGCACAG---ATTTCAAC-TCTAGCCC

ATAACACCTAGCTTTGCCACACCCTCAAGGGTATTCAGCAGTGATTAACCTTGTTAATAA

GCGCCAGCTTGACTCAGTTAGAGGACTT--AGAGCCGGCTAATACGGTGCCAGCCGCCGC

GGCTACACCGCTAGGCTCAAGTTGATAAT-ACTCGGCGTTAAGCGTGTTTAAAGTACCA-

-AAA-GACTAGAACTAAACTTCAACCAAGTTGTGACACACTTGTTTTAAAGAAACCCGAA

AACGAAAGTTGTTCTAACCTGACTACTTGAACCCACGACAGCTAGGGCACAAACTGGGAT

TAGGTACCCCACTATGCCTAGCCGTAAAATATTAACCTACACCACTTAACCGCCAGGGAA

TTACGAGCCCAAGCTTAAAACCCAAAGGACTTGACGGTGTCCCACCCCCCTAGAGGAGCC

TGTTCTATAA-TCGATTCTCCCC-GATATACCCGACCATTTTTGGCCTA--TCAGCCTGT

ATACCTCCGTCGTAAGCTTACCATATGAACGAC---TTAGTGAGCTAAAAGAT--TTTTC

GTAAATACGTCAGGTCAAGGTGCAGCCCACGAAATGGCAAGCAATGGGCTACAATTTCTA

TGC---TAGAACACAACGAAAGGCTGTGTGCAA-CCCAGTCTTGAAGGCGGATTTAGTAG

TAAAAAGGAAGTAGAGAGTCCTTTTTAATTAGGCGCTGGGACGCGTACACACCGCCCGTC

ACCCTCTTCATAAGTATTA-AACAAG-TTTTTAACAAG-ATTTTTCTATGCAGAAGAGGT

AAGTCGTAACACGGTAAGTGTACTGGAAAGTGCACTTGGATTAA-CAAAATGTAGCTTAA

CTAAA-GCATTTCGCTTACACCGAAAACATGTCTTTTTAAACAGGACCATTTTGA--GCC

TAAAACCTAGCCCTA--CTAACATACAATGCCCACT--CAACAACAACCCCCA--TCAAA

TAAAACATTTTACTA-ATTTAGTAAAGGCGATTGAAAAATATTTA-GGAGCTATAGAGAC

CAGTACCGCAAGGGAAAAGTGAAATATTTATGAAAAA-CTTTTAAGCACTATAAAGCAGA

GCCACAACCTCGTACCTTTTGCATCATGGTTTAACTAGTTTAACCAGACAAAACGCAACT

TTAGCCTGACTTCCCGAAACTAGGTGAGCTACTTAAAAACAGCCTGTT-GGGCCAACCCG

TCTCTGTTGCAAAAGAGTGGGAAGATTAT-TAAGTAGTGGTGACAAACCTACCGAACCTA

GAGATAGCTGGTTATTCAGGAAAAGAATTTAAGTTCTACCTTAGGTTTTAT-GTAAC-AC

--AAATACTAAATA-AACCTTAAGAGCTATTCAAACAAGGTACAGCCTGTTTGAAATAGG

GTACAACCTCCTTTAGCGGGTAAC---------------------------------CAG

CCACCTTT-AAAAAAGCGTTAAAGCTTTCTTA---CTTCAAGTCAAAA--TCCCTC-CAC

ACCCTTAAACCCCTAATTTA-CTACTGAATGACTTCATAATTTTATGAAAATCTATATGT

TAAAACTAGTAACAAGAAGA-AGCCCTTCTCCA-AAATGTAAGCTTAAGCCACAATGAAC

AAAGCACTGGCACTTAAAGTCTATAATCCACTAGTAACAACCGCC---TCAAGAAAACAC

TAC------------------TGCTTAT-AACGTTAACCTTACACTAGTACATTTTTGGA

AAGATTAAAAAAATGGGAAGGAACTCGGCAAATAA-TTAACCCCGCCTGTTTACCAAAAA

CATCGCCTTCTGAAGCCT----ATAGAAGGTCCAGCCTGCCCAGTGACAAA--GTTAAAC

GGCCGCGGTACCCTAACCGTGCAAAGGTAGCGCAATCACTTGTTCTTTAAATGAGGACTA

GTATGAATGGCATCACGAGGGTTACACTGTCTCCCCACTTTAATCAGTGAAACTGATCTC

CCCGTGAAGAAGCGGGGATAAACATATAAGACGAGAAGACCCCATGGAGCTTTAAACTCA

GTACCACCTGCCACTTA--AAAAGCCTATAAACCAC-GCAG-CTCTGAT-TACTAGTTTT

TGGTTGGGGTGACCGCGGAGTAAAACAAAACCTCCACAACGAAAGGAACTGATATCCTAA

TACAAGAGCTACACCTCTAAGAATTAAT--------AAATTAACTAAA-CTGACCCAATT

AATT--GATCAATGAACCAAGTTACCCTGGGGATAACAGCGCAATCCATTTCAAGAGCCC

CTATCGACAAATGGGTTTACG-ACC-TCGATGTTGGATCAGGGTGTCCAAGTG-GCGCAG

CCGCTA-CTAA-TGGTTC

>Microhyla sp. 2 MN534741, MN534527, MN534632

TTATTATCAACTCATCCTCAATTTACACATGCAAGTATCAGCACACCCGTGAGAACGCCC

TTC-ACCTT-TTCAGGACAAGGAGCTGGTATCAGGCACAG---ATTTCAAC-TCTAGCCC

ATAACACCTAGCTTTGCCACACCCTCAAGGGTATTCAGCAGTGATTAACCTTGTTAATAA

GCGCCAGCTTGACTCAGTTAGAGGACTT--AGAGCCGGCTAATACGGTGCCAGCCGCCGC

GGCTACACCGCTAGGCTCAAGTTGATAAT-ACTCGGCGTTAAGCGTGTTTAAAGTACCA-

-AAA-GACTAGAACTAAACTTCAACCAAGTTGTGACACACTTGTTTTAAAGAAACCCGAA

AACGAAAGTTGTTCTAACCTGACTACTTGAACCCACGACAGCTAGGGCACAAACTGGGAT

TAGGTACCCCACTATGCCTAGCCGTAAAATATTAACCTACACCACTTAACCGCCAGGGAA

TTACGAGCCCAAGCTTAAAACCCAAAGGACTTGACGGTGTCCCACCCCCCTAGAGGAGCC

TGT-CTATAA-TCGATTCTCCCC-GATATACCCGACCATTTTTGGCCTA--TCAGCCTGT

ATACCTCCGTCGTAAGCTTACCATATGAACGAC---TTAGTGAGCTAAAAGAT--TTTTC

GTAAATACGTCAGGTCAAGGTGCAGCCCACGAAATGGCAAGCAATGGGCTACAATTTCTA

TGC---TAGAACACAACGAAAGGCTGTGTGCAA-CCCAGTCTTGAAGGCGGATTTAGTAG

TAAAAAGGAAGTAGAGAGTCCTTTTTAATTAGGCGCTGGGACGCGTACACACCGCCCGTC

ACCCTCTTCATAAGTATTA-AACAAG-TTTTTAACAAG-ATTTTTCTATGCAGAAGAGGT

AAGTCGTAACACGGTAAGTGTACTGGAAAGTGCACTTGGATTAA-CAAAATGTAGCTTAA

CTAAA-GCATTTCGCTTACACCGAAAACATGTCTTTTTAAACAGGACCATTTTGA--GCC

TAAAACCTAGCCCTA--CTAACATACAATGCCCACT--CAACAACAACCCCCA--TCAAA

TAAAACATTTTACTA-ATTTAGTAAAGGCGATTGAAAAATATTTA-GGAGCTATAGAGAC

CAGTACCGCAAGGGAAAAGTGAAATATTTATGAAAAA-CTTTTAAGCACTATAAAGCAGA

GCCACAACCTCGTACCTTTTGCATCATGGTTTAACTAGTTTAACCAGACAAAACGCAACT

TTAGCCTGACTTCCCGAAACTAGGTGAGCTACTTAAAAACAGCCTGTT-GGGCCAACCCG

TCTCTGTTGCAAAAGAGTGGGAAGATTAT-TAAGTAGTGGTGACAAACCTACCGAACCTA

GAGATAGCTGGTTATTCAGGAAAAGAATTTAAGTTCTACCTTAGGTTTTAT-GTAAC-AC

--AAATACTAAATA-AACCTTAAGAGCTATTCAAACAAGGTACAGCCTGTTTGAAATAGG

GTACAACCTCCTTTAGCGGGTAAC---------------------------------CAG

CCACCTTT-AAAAAAGCGTTAAAGCTTTCTTA---CTTCAAGTCAAAA--TCCCTCACAC

ACCCTTAAACCCCTAATTTA-CTACTGAATGACTTCATAATTTTATGAAAATCTATATGT

TAAAACTAGTAACAAGAAGA-AGCCCTTCTCCA-AAATGTAAGCTTAAGCCACAATGAAC

AAAGCACTGGCACTTAAAGTCTATAATCCACTAGTAACAACCGCC---TCAAGAAAACAC

TAC------------------TGCTTAT-AACGTTAACCTTACACTAGTACATTTTTGGA

AAGATTAAAAAAATGGGAAGGAACTCGGCAAATAA-TTAACCCCGCCTGTTTACCAAAAA

CATCGCCTTCTGAAGCCT----ATAGAAGGTCCAGCCTGCCCAGTGACAAA--GTTAAAC

GGCCGCGGTACCCTAACCGTGCAAAGGTAGCGCAATCACTTGTTCTTTAAATGAGGACTA

GTATGAATGGCATCACGAGGGTTACACTGTCTCCCCACTTTAATCAGTGAAACTGATCTC

CCCGTGAAGAAGCGGGGATAAACATATAAGACGAGAAGACCCCATGGAGCTTTAAACTCA

GTACCACCTGCCACTTA--AAAAACCTATAAACCAC-GCAG-CTCTGAT-TACTAGTTTT

TGGTTGGGGTGACCGCGGAGTAAAACAAAACCTCCACAACGAAAGGAACTGATATCCTAA

TACAAGAGCTACACCTCTAAGAATTAAT--------AAATTAACTAAA-CTGACCCAATT

AATT--GATCAATGAACCAAGTTACCCTGGGGATAACAGCGCAATCCATTTCAAGAGCCC

CTATCGACAAATGGGTTTACG-ACC-TCGATGTTGGATCAGGGTGTCCAAGTG-GCGCAG

CCGCTA-CTAA-CGGTTC

>Microhyla sp. 2 MN534742, MN534528, MN534633

TTATTATCAACTCATCCTCAACTTACACATGCAAGTATCAGCACACCCGTGAGAACGCCC

TTC-ACCTT-TTCAGGACAAGGAGCTGGTATCAGGCACAG---ACTTCAAC-TCTAGCCC

ATAACACCTAGCTTTGCCACACCCTCAAGGGTATTCAGCAGTGATTAACCTTGTTAATAA

GCGCCAGCTTGACTCAGTTAGAGGACTT--AGGGCCGGCTAATACGGTGCCAGCCGCCGC

GGCTACACCGCTAGGCTCAAGTTGATAAT-ACTCGGCGTTAAGCGTGTTTAAAGTACCA-

-AAA-GACTAGAACTAAACTTCAACCAAGTTGTGACACACTTGTTTTAAAGAAACCCGAA

AACGAAAGTTGTTCTAACCTGACTACTTGAACCCACGACAGCTAGGGCACAAACTGGGAT

TAGGTACCCCACTATGCCTAGCCGTAAAATATTAACCTACACCACTTAACCGCCAGGGAA

TTACGAGCCCAAGCTTAAAACCCAAAGGACTTGACGGTGTCCCACCCCCCTAGAGGAGCC

TGT-CTATAA-TCGATTCTCCCC-GATACACCCGACCATTTTTGGCCTA--TCAGCCTGT

ATACCTCCGTCGTAAGCTTACCATATGAACGAC---TTAGTGAGCTAAAAGAT--TTTTC

GTAAATACGTCAGGTCAAGGTGCAGCCCACGAAATGGCAAGCAATGGGCTACAATTTCTA

TGC---TAGAACACAACGAAAGGCTGTGTGCAA-CCCAGTCTTGAAGGCGGATTTAGTAG

TAAAAAGGAAGTAGAGAGTCCTTTTTAATTAGGCGCTGGGACGCGTACACACCGCCCGTC

ACCCTCTTCATAAGTATTA-AACAAG-TTTTTAACAAG-ATTTTTCTATGCAGAAGAGGT

AAGTCGTAACACGGTAAGTGTACTGGAAAGTGCACTTGGATTAA-CAAAATGTAGCTTAA

CTAAA-GCATTTCGCTTACACCGAAAACATGTCTTTTTAAACAGGACCATTTTGA--GCC

TAAAACCTAGCCCTA--CTAACATACAATGCCCACT--CAACAACAACCCCCA--TCAAA

TAAAACATTTTACTA-ATTTAGTAAAGGCGATTGAAAAATATTTA-GGAGCTATAGAGAC

CAGTACCGCAAGGGAAAAGTGAAATATTTATGAAAAA-CTTTTAAGCACTATAAAGCAGA

GCCACAACCTCGTACCTTTTGCATCATGGTTTAACTAGTTTAACCAGACAAAACGCAACT

TTAGCCTGACTTCCCGAAACTAGGTGAGCTACTTAAAAACAGCCTGTT-GGGCCAACCCG

TCTCTGTCGCAAAAGAGTGGGAAGATTAT-TAAGTAGTGGTGACAAACCTACCGAACCTA

GAGATAGCTGGTTATTCAGGAAAAGAATTTAAGTTCTACCTTAGGTTTTAT-GTAAC-AC

--AAATACTAAATA-AACCTTAAGAGCTATTCAAACAAGGTACAGCCTGTTTGAAATAGG

GTACAACCTCCTTTAGCGGGTAAC---------------------------------CAG

CCACATTT-AAAAAAGCGTTAAAGCTTTCTTA---CTTCAAGTCAAAA--TCCCTCACAC

ACCCTTAAACCCCTAATTTA-CTACTGAATGACTTCATAATTTTATGAAAATCTATATGT

TAAAACTAGTAACAAGAAGA-AGCCCTTCTCCA-AAATGTAAGCTTAAGCCACAATGAAC

AAAGCACTGGCACTTAAAGTCTATAATCCACTAGTAACAACCGCC---TCAAGAAAACAC

TAC------------------TGCTTAT-AACGTTAACCTTACACTAGTACATTTTTGGA

AAGATTAAAAAAATGGGAAGGAACTCGGCAAATAA-TTAACCCCGCCTGTTTACCAAAAA

CATCGCCTTCTGAAGCCT----ATAGAAGGTCCAGCCTGCCCAGTGACAAA--GTTAAAC

GGCCGCGGTACCCTAACCGTGCAAAGGTAGCGCAATCACTTGTTCTTTAAATGAGGACTA

GTATGAATGGCATCACGAGGGTTACACTGTCTCCCCACTTTAATCAGTGAAACTGATCTC

CCCGTGAAGAAGCGGGGATAAACATATAAGACGAGAAGACCCCATGGAGCTTTAAACTCA

GTACCACCTGCCACTTA--AAAAACCTATAAACCAC-GCAG-CTCTGAT-TACTAGTTTT

TGGTTGGGGTGACCGCGGAGTAAAACAAAACCTCCACAACGAAAGGAACTGATATCCTAA

TACAAGAGCTACACCTCTAAGAATTAAT--------AAATTAACTAAA-CTGACCCAATT

AATT--GATCAATGAACCAAGTTACCCTGGGGATAACAGCGCAATCCATTTCAAGAGCCC

CTATCGACAAATGGGTTTACG-ACC-TCGATGTTGGATCAGGGTGTCCAAGTG-GCGCAG

CCGCTA-CTAA-CGGTTC

>Microhyla sp. 2 MN534743, MN534529, MN534634

TTATTATCAGCCCACCCTCAATTTACACATGCAAGTATCAGCACACCCGTGAGAACGCCC

TTC-ACCTT-TTCAGGACAAGGAGCTGGTATCAGGCACAG---ACTTAAAC-TCTAGCCC

ATAACACCTAGCTTTGCCACACCCTCAAGGGTATTCAGCAGTGATTAACCTTGTTAATAA

GCGCCAGCTTGACTCAGTTAGAGGACTT--AGAGCCGGCTAATACGGTGCCAGCCGCCGC

GGCTACACCGCTAGGCTCAAGTTGATAAT-ACTCGGCGTTAAGCGTGTTTAAAGTACCA-

-AAA-GACTAGAACTAAACTTCAACCAAGTTGTGACACACTTGTTTTAAAGAAACCCGAA

AACGAAAGTTGTTCTAACCTGACTACTTGAACCCACGACAGCTAGGGCACAAACTGGGAT

TAGGTACCCCACTATGCCTAGCCGTAAAATATTAACCTACACCACTTAACCGCCAGGGAA

TTACGAGCCCAAGCTTAAAACCCAAAGGACTTGACGGTGTCCCACCCCCCTAGAGGAGCC

TGTTCTAAAA-TCGATTCTCCCC-GATATACCCGACCATTTTTGGCCTA--TCAGCCTGT

ATACCTCCGTCGTAAGCTTACCATATGAACGAC---TTAGTGAGCTAAAAGAT--TTTTC

GTAAATACGTCAGGTCAAGGTGCAGCCCACGAAATGGCAAGCAATGGGCTACAATTTCTA

TGC---TAGAACACAACGAAAGGCTGTGTGCAA-CCCAGTCTTGAAGGCGGATTTAGTAG

TAAAAAGGAAGTAGAGAGTCCTTTTTAATTAGGCGCTGGGACGCGTACACACCGCCCGTC

ACCCTCTTCATAAGTACTA-AACAAG-TTTTTAACAAG-ATTTTTCTATGCAGAAGAGGT

AAGTCGTAACACGGTAAGTGTACTGGAAAGTGCACTTGGATTAA-CAAAATGTAGCTTAA

CTAAA-GCATTTCGCTTACACCGAAAACATGTCTTTTTAAACAGGACCATTTTGA--GCC

TAAAACCTAGCCCTA--CTAACATACAATGCCCACT--CAACAACAACCCTCA--TCAAA

TAAAACATTTTACTA-ATTTAGTAAAGGCGATTGAAAAATATTTA-GGAGCTATAGAAAC

CAGTACCGCAAGGGAAAGGTGAAATATTTATGAAAAA-CTTTTAAGCACTATAAAGCAGA

GCCACAACCTCGTACCTTTTGCATCATGGTTTAACTAGTTTAACCAGACAAAACGCAACT

TTAGCCTGACTTCCCGAAACTAGGTGAGCTACTTAAAAACAGCCTGTT-GGGCCAACCCG

TCTCTGTTGCAAAAGAGTGGGAAGATTAT-TAAGTAGTGGTGACAAACCTACCGAACCTA

GAGATAGCTGGTTATTCAGGAAAAGAATTTAAGTTCTACCTTAGGTTTCAT-GTAAC-AC

--AAATACTAAATA-AACCTTAAGAGCTATTCAAACAAGGTACAGCCTGTTTGAAATAGG

ATACAACCTCC-TTAGCGGGTAAC------------------------------------

---------AAAAAAGCGTTAAAGCTTTCTTA---CTTCAAGTCAAAA--TTTCTTACAC

ACCCTCAAACCCGTAATTTA-CTACTGAATGACTTCATAATTTTATGAAAACCTATATGT

TAAAACTAGTAACAAGAAGA-AGCCCTTCTCCA-AAATGTAAGCTTAAGCCACAATGAAC

AAAGCACTGGCACTTAAAGTCTATAATCCACTAGTAGTAACCCC----TCAAGAAAACAC

TAC------------------TGCTTAT-AACGTCAACCTTACACTAGTACATTTTTGGA

AAGATTAAAAAAATGGGAAGGAACTCGGCAAATAA-TTAACCCCGCCTGTTTACCAAAAA

CATCGCCTTCTGAAACCT----ATAGAAGGTCCAGCCTGCCCAGTGACAAA--GTTAAAC

GGCCGCGGTACCCTAACCGTGCAAAGGTAGCGCAATCACTTGTTCTTTAAATGAGGACTA

GTATGAATGGCATCACGAGGGTTACACTGTCTCCCCACTTTAATCAGTGAAACTGATCTC

CCCGTGAAGAAGCGGGGATAAACATATAAGACGAGAAGACCCCATGGAGCTTTAAACTCA

GTACCACCTGCCACTTA--AAAAACCTATAAACCAC-GCAG-CTTTGAT-TACTAGTTTT

TGGTTGGGGTGACCGCGGAGTAAAATAAAACCTCCACAACGAAAGGAACTAATATCCTAA

TATAAGAGCTACACCTCTAAGAATTAAT--------AAATTAACTAAA-CTGACCCAATT

AATT--GATCAATGAACCAAGTTACCCTGGGGATAACAGCGCAATCCATTTCAAGAGCCC

CTATCGACAAATGGGTTTACG-ACC-TCGATGTTGGATCAGGGTGTCCAAGTG-GCGCAG

CCGCTA-CTAA-CGGTTC

>M. superciliaris MN534744, MN534530, MN534635

TTACTATCAACCCCCTCTCGACTTACACATGCAAGTATCAGCACCCCCGTGAGAACGCCC

TTT-ACCTT-TTTAGGACAAGGAGCTGGTATCAGGCACAG---AAT------TCTAGCCC

ATAACACCTAGCTTTGCCACACCCTCAAGGGTATTCAGCAGTGATTAACATTGTCCATAA

GCGCCAGCTTGACACAGTCAGAGAGCTG--AGGGCCGGCCAATACGGTGCCAGCCGCCGC

GGCTACACCGCGAGGCTCAAGTTGATAAT-ATTCGGCGTTAAGCGTGTTTAAAGTGCCA-

ATAA-GACTAGAATTAAACTTCAACCAAGTTGTGACACACTTGTTCTTAAGAAAGCCAAA

AACGAAAGTTATTCTAACCAGACCACTTGAACCCACGACAGCTAAGATACAAACTGGGAT

TAGGTACCCCACTATGCTTAGCCGTAAAACATTCACTTACACCCCTTAACCGCCCGGGAA

TTACGAGCCCAAGCTTAAAACCCAAAGGACTTGACGGTGTCCCACCCACCTAGAGGAGCC

TGATCTAAAA-TCGATTCTCCCC-GATCTACCTCACCACTTCTAGCCTA--TCAGCCTGT

ATACCTCCGTCGTAGGCTTACCATATGAACGAC---TCAGTGAGCTAAAAGAT--TATTC

ATAAATACGTCAGGTCAAGGTGCAGCTTACGAAGCGGCAAGTAATGGGCTACAATTTCTA

CAA---TAGAACACA-CGAAAGACTACATGCAA-CCCAGTCATGAAGGCGGATTTAGAAG

TAAAAAGGAAACAGAGAGTCCTTTTTAATTAGGCGCTGGGACGCGTACACACCGCCCGTC

ACCCTCTTCAAATACTACA-GCAAAGCTATATAACAAA-CTAACACCAAACAGAAGAGGT

AAGTCGTAACACGGTAAGCGTACTGGAAAGTGCGCTTGGATTAA-CAAAGTGTAGCTTAA

CCAAA-GCATCTCGCTTACACCGAGAATATATCTGTTCAACTCGGATCACCTTGA--GCC

AAAAACCTAGCTCCC--CTACTTTAC-CTATACTCGCCCAACAGTTACCACAG--CATAA

TAAAACATTTTACCC-ATTTAGTAAAGGCGATTAAAAAATGTCTA-GAAGCTATAGAAAC

CAGTACCGCAAGGGAAAAGTGAAATAAAAGTGAAAAA-TTTTAAAGCATTAAAAAGAAGA

GCTACAACCTCGTACCTTTTGCATCATGGTTTAACTAGTTTAACCAGACAAAACGCAATT

TCAGCCTGACATCCCGAAACTAGGTGAGCTACTTAAAAACAGCCTCA-AGGGCAAACCCG

TCTCTGTTGCAAAAGAGTGGGAAGATTAT-TAAGTAGTGGTGACAGACCTACCGAACCTA

GAGATAGCTGGTTACTCAAAAAAAGAATTTAAGTTCTACCTTAGGTTTTAC-GTAAC-AC

--AAATACCCCCCA-AACCTTAAGAGCTATTCAAACAAGGTACAGCCTGTTTGAAATAGG

GTACAACCTCGCCTAGCGGGTAAC----------------ATAAGTGGGCCTAAAAGCAG

CCACCTTCTAAAAAAGCGTTAAAGCTTCATTA---CCCTTAATAAAAA-TACCGCCA-AC

TAACTTTAACCCCTAAACCC-CTATTGAGTGACTTCATAAATTTATGAAAATCTATCTGT

TAAAACTAGTAACAAGAAGA-AGCCCTTCTCCA-AAATGTAAGTATAAGCCACAATGAAC

AAAGCATCGGCACTTAAAGCCAATAAACATCCAGTAACAACCCC----GCAAGAAAACCC

TAC------------------TGCCAAC-AACGTTAATCTTACACCAGCACATTTATGGA

AAGATTTAAAGAGTAGGAAGGAACTCGGCAAACAA-CTAACCCCGCCTGTTTACCAAAAA

CATCGCCTCCTGAAACCC----ATAAGAGGTCCAGCCTGCCCAGTGACTTA--GTTAAAC

GGCCGCGGTACCCTAACCGTGCAAAGGTAGCGCAATCACTTGTTCTTTAAATGAGGACTA

GTATGAACGGCATCACGAGGGTTACACTGTCTCCCTACTTTAATCAGTGAAACTAATCCC

CCCGTGAAGAAGCGGGGATAAACATATAAGACGAGAAGACCCCATGGAGCTTTAAACTCA

GTACCACCTGCCACTAC--TAAAACTTATAAACCAC-GCAG-TGCTGTC-TACTGGTTTT
CGGTTGGGGTGACCACGGAGTAAAACAAATCCTCCACAACGAAAGGAACTAACAACCTAA
TCTAAGAGCCACACCTCTAAGAATCAAT--------AAACTGACTAAA-CTGATCCAATT
ATTT--GATCAATGAACCAAGTTACCCTGGGGATAACAGCGCAATCCATTTCAAGAGCCC
ATATCGACAAATGGGTTTACG-ACC-TCGATGTTGGATCAGGGTATCCAAGTG-GCGCAG
CCGCTA-CTAA-AGGTTC

>M. superciliaris MN534745, MN534636

TTACTATCAACCCCCTCTCGACTTACACATGCAAGTATCAGCACCCCCGTGAGAACGCCC
TTT-ACCTT-TTTAGGACAAGGAGCTGGTATCAGGCACAG---AAT------TCTAGCCC
ATAACACCTAGCTTTGCCACACCCTCAAGGGTATTCAGCAGTGATTAACATTGTCCATGA
GCGCCAGCTTGACACAGTCAGAGAGCTG--AGGGCCGGCCAATACGGTGCCAGCCGCCGC
GGCTACACCGCGAGGCTCAAGTTGATAAT-ATTCGGCGTTAAGCGTGTTTAAAGTGCCA-
ATAA-GACTAGAATTAAACTTTAACCAAGTTGTGACACACTTGTTCTTAAGAAACTCAAA
AACGAAAGTTATTCTAACCAGACCACTTGAACCCACGACAGCTAAGATACAAACTGGGAT
TAGGTACCCCACTATGCTTAGCCGTAAAACATTTACTTACACCCCTTAACCGCCCGGGAA
TTACGAGCCCAAGCTTAAAACCCAAAGGACTTGACGGTGTCCCACCCACCTAGAGGAGCC
TGTTCTAAAA-TCGATTCTCCCC-GATCTACCTCACCACTTCTAGCCTA--TCAGCCTGT
ATACCTCCGTCGTAAGCTTACCATATGAACGAC---TCAGTGAGCTAAAAGAT--TATTC
ATAAATACGTCAGGTCAAGGTGCAGCCTACGAAGCGGCAAGTAATGGGCTACAATTTCTA
CAA---TAGAACACA-CGAAAGACTACATGCAA-CCCAGTCATGAAGGCGGATTTAGAAG
TAAAAAGGAAACAGAGAGTCCTTTTTAATTAGGCGCTGGGACGCGTACACACCGCCCGTC
ACCCTCTTCAAATACTACA-GCAAAGCTATATAACAAA-CTAACACCAAACAGAAGAGGT
AAGTCGTAACACGGTAAG--

--AA
TAAAACATTTTACCC-ATTTAGTAAAGGCGATTAAAAAATTTTTA-GAAGCTATAGAAAC
CAGTACCGCAAGGTAAAAGTGAAATAAAAATGAAAAA-TTTTTAAGCATTAAAAAGCAGA
GCTACAACCTCGTACCTTTTGCATCATGGTTTAACTAGTCTAACCAGACAAAACGCAATT
TCAGCCTGACATCCCGAAACTAGGTGAGCTACTTAAAAACAGCCTCA-AGGGCAAACCCG
TCTCTGTTGCAAAAGAGTGGGAAGATTAT-TAAGTAGTGGTGACAGACCTACCGAACCTA

GAGATAGCTGGTTACTCAAAAAAAGAATTTAAGTTCTACCTTAGGTTTTAC-GTAAC-AC

--AAATACCCCACA-AACCTTAAGAGCTATTCAAACAAGGTACAGCCTGTTTGAAATAGG

GTACAACCTCGCCTAGCGGG-------------------ATAAGTGGGCCTAAAAGCAG

CCACCTTCTAAAAAAGCGTTAAAGCTTCATTA---CCCTTAATAAAAA-TACCGCCA-AC

TAACTTTAACCCCTAAACCC-CTATTGAGTGACTTCATAAATTTATGAAAATCTATCTGT

TAAAACTAGTAACAAGAAGA-AGCCCTTCTCCA-AAATGTAAGTATAAGCCACAATGAAC

AAAGCATCGGCACTTAAAGCCAATAAACATCCAGTAACAACCCC----GCAAGAAAACCC

TAC------------------TGCCAAC-AACGTTAATCTTACACCAGCACATTTATGGA

AAGATTTAAAGAGTTGGAAGGAACTCGGCAAACAA-CTAACCCCGCCTGTTTACCAAAAA

CATCGCCTCCTGAAACCT----ATAAGAGGTCCAGCCTGCCCAGTGACTTA--GTTAAAC

GGCCGCGGTACCCTAACCGTGCAAAGGTAGCACAATCACTTGTTCTTTAAATGAGGACTA

GTATGAACGGCATCACGAGGGTTACACTGTCTCCCTACTTTAATCAGTGAAACTAATCCC

CCCGTGAAGAAGCGGGGATAAACATATAAGACGAGAAGACCCCATGGAGCTTTAAACTCA

GTACCACCTGCCACTAC--TAAAACTTATAAACCAC-GCAG-TATTGTC-TACTGGTTTT

CGGTTGGGGTGACCACGGAGTAAAACAAATCCTCCACAACGAAAGGAACTAACAACCTAA

TCTAAGAGCCACACCTCTAAGAATCAAT--------AAACTGACTAAA-CTGATCCAATT

ATTT--GATCAATGAACCAAGTTACCCTGGGGATAACAGCGCAATCCATTTCAAGAGCCC

ATATCGACAAATGGGTTTACG-ACC-TCGATGTTGGATCAGGGTATCCAAGTG-GCGCAG

CCGCTA-CTAA-AGGTTC

>M. annectens MN534746, MN534531, MN534637

TTATTGTCAGCTGTTTATTAACTTACACATGCAAGCATCAACACCCCCGTGAGAACGCCC

CC-TACCTCCACCAGGGTGGGGAGCTGGTATCAGGCACAG-----TT-----TCTGGCCC

ACAACACCTAGCTTTGCCACACCCCCAAGGGTCATCAGCAGTGATTAACATTGTCCATGA

GCGCCAGCTCGACTCAGTTAAATAAAAC--AGGGCCGGCCAATCCGGTGCCAGCCGCCGC

GGCTACACCGTCGGGCTCAAGTTGACAATTATTCGGCGTTAAGCGTGTTTAAAGTGACAA

ACAATACCTAGAATTAAACTTAAACCAAGTTGTGACACACTTGTTTTTAAGAAAGCCATA

AACGAAAGTTATTCTAGTCAAACCACTTGAATCCACGACAGCTAGGGCACAAACTGGGAT

TAGGTACCCCACTATGCCTAGCCGTAAAATATTTACTTACACCT--AACCCGCCAGGGAA

TTACGAGCCCAAGCTTAAAACCCAAAGGACTTGACGGTGTCCCACCCATCTAGAGGAGCC

TGTTCTATAA-TCGATTC--

---TACACACCGCCCGTC
ACCCTTTTCAAATGTAACATCCCAAG-TTTTTAACAAT-TCAACTACC-ACAGAAGAGGT
AAGTCGTAACACGGTAAGGGTACTGGAAAGTGGGCTTGGATCAA-CAAAGTGTAGCTTAA
TCAAA-GCATTTCGCTTACACCGAGAATATGTTTAATAATATCAGATCACCTTGAACGCT
TAAAACCTAGCTTTACCCCCCCCCCGCATGAA--CAC-CCAAAACTACCCAC--CCACAA
TAAAACATTTTTCATCATCTAGTAGAAGCGATCAAAAAATGTCTT-AAAGCTATAAAAA-
TAGTACCGCAAGGGAAAGGTGAAATAAAAATGAAACAACTATTAAGCCCCAAAAAGCAGA
GCTTCAACCTCGTACCTTTTGCATCATGGTTTAGCTAGTCTAACCAAGCAAAACGCACTT
TTAGTTTGACATCCCGAAACTGGACGAGCTACTTTGAAACAGCTTTT-AGAGCAAACCCG
TCTCTGTTGCAAAAGAGTGGGAAGATTTC-TAAGTAGAGGCGATAAACCTACCGAGCCCA
GAGATAGCTGGTTATTCAGGAAAAGGATCTAAGTCCTACCTTAAGTT-TCC-CTATA-AC
CTCAAATATCTTACTAAACTTAAGAGCTATTCAAATAAGGTACAGCCTATTTGAACCAGG
ATACAACCTACCCCG---------------------------------------AAGCAG
CCACCTTCCAAAAAAGCGTTAAAGCTTTATTATA--ATTACATTTGAA-TACCCCAT-TC
TTATCAAAACCCTCCAACC--ATACTGAATGACTTCATACCCCTATGAAGGACCATATGC
TAAAACTAGTAACAAGAAGA-AGACCTTCTCCA-AAATGTAAGCGTAAGTCATAATGAAC
AATTCACTGACACTTAACGTCTATGAGCCTACAGTGGCAACCCAC--ATCAAGAAAACCC
CAC------------------TTTTCAT-CACGTTAACCTTACACTAGAACATTTACGGA
AAGATTAAAAGAGGAAGAAGGAACTCGGCAAACTA-CTAACCCCGCCTGTTTACCAAAAA
CATCGCCTCCTGACTAACC---ATAGGAGGTCCAGCCTGCCCAGTGACCCA--GTTAAAC
GGCCGCGGTACCCTAACCGTGCAAAGGTAGCACAATCACTTGTTCTTTAAATGAGGACTA
GTATGAATGGCATCACGAGGGTTATACTGTCTCCCCCCTCTAATCAGTGAAACTGATCTC
CCCGTGAAGAAGCGGGGATATTATTATAAGACGAGAAGACCCCATGGAGCTTTAAACTCA
GTATCACCTGCCAACCA-CTCCAGCCTATTAACCAC-GCAG-TTCTGAC-TACTAGTTTT
CGGTTGGGGTGACCGCGGAGTAAAATAAAACCTCCTCGATGAATGGAGCTAACCTCCTTA
TCCAAGAGCCACAACTCTAAGAATCAAA---------TCATTGACAAAATTGATCCAATT
TATATTGATCAACGAACCAAGTTACCCTGGGGATAACAGCGCAATCTACTTCAAGAGCTC

CTATCGACAAGTGGGTTTACG-ACC-TCGATGTTGGATCAGGGTATCCCAGTG-GCGCAG

CCGCTA-CTAA-AGGTTC

>M. annectens MN534747, MN534532, MN534638

TTATTGTCAGCTGTTTATTAACTTACACATGCAAGCATCAACACCCCCGTGAGAACGCCC

CC-TACCTCCACCAGGGTGGGGAGCTGGTATCAGGCACAG-----TT-----TCTGGCCC

ACAACACCTAGCTTTGCCACACCCCCAAGGGTCATCAGCAGTGATTAACATTGTCCATGA

GCGCCAGCTCGACTCAGTTAAATAAAAC--AGGGCCGGCCAATCCGGTGCCAGCCGCCGC

GGCTACACCGTCGGGCTCAAGTTGACAATTATTCGGCGTTAAGCGTGTTTAAAGTGACAA

ACAATACCTAGAATTAAACTTAAACCAAGTTGTGACACACTTGTTTTTAAGAAAGCCATA

AACGAAAGTTATTCTAGTCAAACCACTTGAATCCACGACAGCTAGGGCACAAACTGGGAT

TAGGTACCCCACTATGCCTAGCCGTAAAATATTTACTTACACCT--AACCCGCCAGGGAA

TTACGAGCCCAAGCTTAAAACCCAAAGGACTTGACGGTGTCCCACCCATCTAGAGGAGCC

TGTTCTATAA-TCGATTC--

---TACACACCGCCCGTC

ACCCTTTTCAAATGTAACATCCCAAG-TTTTTAACAAT-TCAACTACC-ACAGAAGAGGT

AAGTCGTAACACGGTAAGGGTACTGGAAAGTGGGCTTGGATCAA-CAAAGTGTAGCTTAA

TCAAA-GCATTTCGCTTACACCGAGAATATGTTTAATAATATCAGATCACCTTGAACGCT

TAAAACCTAGCTTTA-------------------CAC-CCAAAACTACCCAC--CCACAA

TAAAACATTTTTCATCATCTAGTAGAAGCGATCAAAAAATGTCTT-AAAGCTATAAAAA-

TAGTACCGCAAGGGAAAGGTGAAATAAAAATGAAACAACTATTAAGCCCCAAAAAGCAGA

GCTTCAACCTCGTACCTTTTGCATCATGGTTTAGCTAGTCTAACCAAGCAAAACGCACTT

TTAGTTTGACATCCCGAAACTGGACGAGCTACTTTGAAACAGCTTTT-AGAGCAAACCCG

TCTCTGTTGCAAAAGAGTGGGAAGATTTC-TAAGTAGAGGCGATAAACCTACCGAGCCCA

GAGATAGCTGGTTATTCAGGAAAAGGATCTAAGTCCTACCTTAAGTT-TCC-CTATA-AC

CTCAAATATCTTACTAAACTTAAGATCTATTCAAATAAGGTACAGCCTATTTGAACCAGG

ATACAACCTACCCCG---------------------------------------AAGCAG

CCACCTTCCAAAAAAGCGTTAAAGCTTTATTATA--ATTACATCTGAA-TACCCCAT-TC

TTATCAAAACCCTCCAACC--ATACTGAATGACTTCATACCCCTATGAAGGACCATATGC
TAAAACTAGTAACAAGAAGA-AGACCTTCTCCA-AAATGTAAGCGTAAGTCATAATGAAC
AATTCACTGACACTTAACGTCTATGAGCCTACAGTGGCAACCCGC--ATCAAGAAAACCC
CAC------------------TTTTCAT-CACGTTAACCTTACACTAGAACATTTACGGA
AAGATTAAAAGAGGAAGAAGGAACTCGGCAAACTA-CTAACCCCGCCTGTTTACCAAAAA
CATCGCCTCCTGACTAACC---ATAGGAGGTCCAGCCTGCCCAGTGACCCA--GTTAAAC
GGCCGCGGTACCCTAACCGTGCAAAGGTAGCACAATCACTTGTTCTTTAAATGAGGACTA
GTATGAATGGCATCACGAGGGTTATACTGTCTCCCCCCTCTAATCAGTGAAACTGATCTC
CCCGTGAAGAAGCGGGGATATTATTATAAGACGAGAAGACCCCATGGAGCTTTAAACTCA
GTATCACCTGCCAACCA-CTCCAGCCTATTAACCAC-GCAG-TTCTGAC-TACTAGTTTT
CGGTTGGGGTGACCGCGGAGTAAAATAAAACCTCCTCGATGAATGGAGCTAACCTCCTTA
TCCAAGAGCCACAACTCTAAGAATCAAA--------TCATTGACAAAAATTGATCCAATT
TATATTGATCAACGAACCAAGTTACCCTGGGGATAACAGCGCAATCTACTTCAAGAGCTC
CTATCGACAAGTGGGTTTACG-ACC-TCGATGTTGGATCAGGGTATCCCAGTG-GCGCAG
CCGCTA-CTAA-AGGTTC
>M. annamensis MN534748, MN534533, MN534639
TTATTATCAACTACTTATCAACTTACACATGCAAGTCTCAGCACCCCCGTGAGAACGCCC
TTCAACCTCGGCCAGGACAAGGAGCCGGTATCAGGCACAG----AAAA----TCTTGCCC
ACAACACCTAGCTTTGCCACTCCCCCAAGGGTCTTCAGCAGTGATTAACATTGTATATGA
GCGCCAGCTCGACTCAGTTAGATAAAAC--AGAGCCGGCTAATCCGGTGCCAGCCGCCGC
GGCTACACCGTTGGGCCCAAGTTGACAATCATTCGGCGTTAAGCGTGTTTAAAGTTTCT-
ACATTTATTAGGATTAAACTTAAACCAAGTTGTGACACACTTGTTTATAAGAAAGCCAGA
AACGAAAGTTATTCTAATAAAACCACTTGAACCCACGACAGCTAGGACACAAACTGGGAT
TAGGTACCCCACTATGCCTAGCCGTAAAATATTTACTTACACCA--AACCCGCCTGGG-A
TTACGAGCTCAAGCTTAAAACCCAAAGGACTTGACGGTGTCCCACCCATCTAGAGGAGCC
TGTTCTATAA-TCGATTCTCCCC-GATACACCTCACCACTTCTAGCCTC--TCAGCCTGT
ATACCTCCGTCGCAAGCTTACCATATGAACGAA-TCTTAGTGAGCTTAAACAT--CAACC
ATAAACACGTCAGGTCAAGGTGCAGCCAACGATGTGGGAAGCAATGGGCTACAATTTCTA
CCAA--TAGAACAAA-CGAAAGACTACATGCAA-CTCGGTCATGAAGGCGGATTTAGCAG
TAAAAAGAAAATAGAGAGTTCTTTTTAACTAGGCACTGGGACGCGTACACACCGCCCGTC

ACCCTCTTCAAATGAAACCCCCACAG-TTTTTAACTTC-ATTGCTCACCACAGAAGAGGT

AAGTCGTAACACGGTAAGCGTACTGGAAAGTGCGCTTGGATTAA-CAAAGTGTAGCTTAA

CCAAA-GCACCTCGCTTACACCGAGAATATGTCTGTTAAACTCAGATCACCTTGAA-GCC

TAAAACCTAGCTTTAAAACCTCCCCCAATACCCCCAC-CCCCAATTTTTCCC---CACAA

TAAAACATTTTCCATCATTTAGTAAAGGCGATTAAAAAATGTCTT-AAAGCTACAAAAA-

TAGTACCGCAAGGGAAAGGTGAAATAGAAATGAAACAACTCTTAAGCCCCAAAAAGCAGA

GCTAAAACCTCGTACCTTTTGCATTATGGTTTAGCCAGTCCAACCAAGCAAAATGCACTT

TCAGTTTGACCCCCCGAAATTAAGCGAGCTACTTCAAAACAGCCTTTTAGGGCAAACCCG

TCTCTGTTGCAAAAGAGTGGGAAGATTTT-CAAGTAGGGGTGATAAACCTACCGAGCTTA

AAGATAGCTGGTTATTCAGGAAAAGGATTTTAGTCCTACCTTAAGTTTTTT---------

--------------------------------------TTTTAACTCAA-TACCCCCACC

CAAACGAAACCCTCCATCT--ATACTGAATGACTTCATA-CCCTATGAAGGACCATATGC

TAAAACTAGTAACAAGAAGA-AGACCTTCTCCA-AAATGCAAGCGTAAGTCATTATGAAC

AACTCACTGACACTTAACGTTTTTGAATCTAAAGTAGTAACTTA----TCAAGAAAATTC

TAC------------------TAGACAT-CACGTCAACCTTACACCAGAGCATTACCGGA

AAGATTAAAAGAAGGGGAAGGAACTCGGCAAATA--TTAACCCCGCCTGTTTACCAAAAA

CATCGCCTCCTGACTACTCT--ATAGGAGGTCTAGCCTGCCCAGTGACTTA--GTTAAAC

GGCCGCGGTACCCTAACCGTGCAAAGGTAGCGCAATCACTTGTTCTTTAAATGAGGACTA

GTATGAACGGCATCACGAGGGTTACACTGTCTCCCCCCTCTAATCAGTGAAACTGATCTC

CCCGTGAAGAAGCGGGGATAAACATATAAGACGAGAAGACCCCATGGAGCTTTAAACTCA

GTATCACCTGCCTTACT-CTATACCCATTTAATCAC-GCAG-TTTTGAA-TACTAGTTTT

CGGTTGGGGTGACCGCGGAGTAAAACAAAACCTCCTCGATGAACGGAACAATACTCCTAA

TCCAAGAGCTACAGCTCTAAGAATCAAA--------AAATTGACAAAAATTGATCCAAAT

ATATTTGATCAACGAACCAAGTTACCCTGGGGATAACAGCGCAATCTACTTCAAGAGCTC

CTATCGACAAGTGGGTTTACG-ACC-TCGATGTTGGATCAGGGTATCCTAGTG-GCGCAG

CCGCTA-CTAA-AGGTTC

>M. annamensis MN534749, MN534534, MN534640

TTATTATCAACTACTTATCAACTTACACATGCAAGTCTCAGCACCCCTGTGAGAACGCCC

```
TTCAACCTCAACCAGGACAAGGAGCCGGTATCAGGCACAG----AAAA----TCTTGCCC
ACAACACCTAGCTTTGCCACTCCCCCAAGGGTCCTCAGCAGTGATTAACATTGTATATGA
GCGCCAGCTCGACTCAGTTAGATAAAAC--AGAGCCGGCTAATCCGGTGCCAGCCGCCGC
GGCTACACCGTTGGGCCCAAGTTGACAATCATTCGGCGTTAAGCGTGTTTAAAGTTTTT-
ACATTTATTATGATTAAACTTAAACCAAGTTGTGACCCACTTGTTTATAAGAAAGCCAGA
AACGAAAGTTATTCTAATAAAACCACTTGAACCCACGACAGCTAGGACACAAACTGGGAT
TAGGTACCCCACTATGCCTAGCCGTAAAATATTTACTTACACCA--AACCCGCCCGGG-A
TTACGAGCTCAAGCTTAAAACCCAAAGGACTTGACGGTGTCCCACCCATCTAGAGGAGCC
TGTTCTATAA-TCGATTCTCCCC-GATACACCTCACCACTTCTAGCCTC--TCAGCCTGT
ATACCTCCGTCGCAAGCTTACCATATGAACGAA-TCCTAGTGAGCTTAAACAT--CAACC
ATAAACACGTCAGGTCAAGGTGCAGCCAACGATGTGGGAAGCAATGGGCTACAATTTCTA
CCAA--TAGAACAAA-CGAAAGACTACATGCAA-CTCGGTCATGAAGGCGGATTTAGCAG
TAAAAAGAAAATAGAGAGTTCTTTTTAATTAGGCACTGGGACGCGTACACACCGCCCGTC
ACCCTCTTCAAATGAAACCCCCACAG-TTTTTAACTTC-ATTACTCACCACAGAAGAGGT
AAGTCGTAACACGGTAAGCGTACTGGAAAGTGCGCTTGGATTAA-CAAAGTGTAGCTTAA
CCAAA-GCACCTCGCTTACACCGAGAATATGTCTGTTAAACTCAGATCACCTTGAA-GCC
TAAAACCTAGCTTTAAAACCTCCCCCAATACCCCCAC-CCCCAATTTTTCCC---CACAA
TAAAACATTTTCCATCATTTAGTAAAGGCGATTAAAAAATGTCTT-AAAGCTACAAAAA-
TAGTACCGCAAGGGAAAGGTGAAATAGAAATGAAATAACTCTTAAGCCCCAAAAAGCAGA
GCTAAAACCTCGTACCTTTTGCATTATGGTTTAGCCAGTCCAACCAAGCAAAATGCACTT
TCAGTTTGACCCCCCGAAATTAAGCGAGCTACTTCAAAACAGCCTTTTAGGGCAAACCCG
TCTCTGTTGCAAAAGAGTGGGAAGATTTT-CAAGTAGTGGTGATAAACCTACCGAGCTTA
AAGATAGCTGGTTATTCAGGAAAAGGATTTTAGTCCTACCTTAAGTTTTTT-T-------
------------------------------------------------------------
------------------------------------------------------------
--------------------------------------TTTTAACTCAA-TACCCCCACC
CAAACGAAACCCTCCATCT--ATACTGAATGACTTCATA-CCCTATGAAGGACCATATGC
TAAAACTAGTAACAAGAAGA-AGACCTTCTCCA-AAATGCAAGCGTAAGTCATTATGAAC
AACTCACTGACACTTAACGTTTTTGAATCTAAAGTAGTAACTTA----CCGAGAAAATTC
TAC------------------TAAACAT-CACGTCAACCTTACACCAGAGCATTACCGGA
```

AAGATTAAAAGAAGGGGAAGGAACTCGGCAAATA--TTAACCCCGCCTGTTTACCAAAAA

CATCGCCTCCTGACTACTCT--ATAGGAGGTCTAGCCTGCCCAGTGACTTA--GTTAAAC

GGCCGCGGTACCCTAACCGTGCAAAGGTAGCGCAATCACTTGTTCTTTAAATGAGGACTA

GTATGAACGGCATCACGAGGGTTACACTGTCTCCCCCTCTAATCAGTGAAACTGATCTC

CCCGTGAAGAAGCGGGGATAAACATATAAGACGAGAAGACCCCATGGAGCTTTAAACTCA

GTATCACCTGCCTTACT-CTATACCCACTTAATCAC-GCAG-TTTTGAA-TACTAGTTTT

CGGTTGGGGTGACCGCGGAGTAAAACAAAACCTCCTCGATGAATGGAATAATACTCCTAA

TCCAAGAGCTACAGCTCTAAGAATCAAA--------AAATTGACAAAAATTGATCCACAT

ATATTTGATCAACGAACCAAGTTACCCTGGGGAGAACAACGCAATCTACTTCAAGAGCTC

CTATCGACAAATGGGTTTACG-ACC-TCGATGTTGGATCAGGGTATCCTAGTG-GCGCAG

CCGCTA-CTAA-AGGTTC

>M. marmorata MN534750, MN534535, MN534641

TTATTGTCAACTATTTATCAACTTACACATGCAAGTCTCAGCATCCCTGTGAGAACGCCC

TTTTACCTCATCAAGGAAAAGGAGCTGGTATCAGGCACAG-----TA-----TCTCGCCC

ACAACACCTAGCTTTGCCACACCCCCAAGGGTACTCAGCAGTGATTAACATTGATTATGA

GCGCCAGCTCGACTCAGTTAGATAAAAT--AGAGCCGGCTAATCCGGTGCCAGCCGCCGC

GGCTACACCATCAGGCCCAAGCTGACAATCATTCGGCGTTAAGCGTGTTTAAAGTGTTTC

CCCTATATTAGAATTAAACTTAAACCAAGTCGTGACACACTTGTTTATAAGAAAGCCTCA

AACGAAAGTTATTCTAACCAAACCACTTGAATCCACGACAGCTAGGACACAAACTGGGAT

TAGGTACCCCACTATGCCTAGCCGTAAAATATTTACTTACACCA--AACCCGCCGGGGAA

TTACGAGCTCAAGCTTAAAACCCAAAGGACTTGACGGTGTCCCACCCATCTAGAGGAGCC

TGTTCTATAA-TCGATTCTCCCC-GATATACCTCACCATATCTAGCCTT--TCAGCCTGT

ATACCTCCGTCGCAAGCTTACCATATGAACGCA-TCCTAGTGAGCTTAAATAT--ATATC

ATAAATACGTCAGGTCAAGGTGCAGCCAACGTCATGGAAAGTAATGGGCTACAATTTCTA

CCA---TAGAACAAA-CGAAAGACTACATGCAA-CTTAGTCATGAAGGCGGATTTAGCAG

TAAAAAGAAAATAGAGAGTTCTTTTTAATTAGGCACTGGGACGCGTACACACCGCCCGTC

ACCCTCTTCAAATGAAACTACTAAAG-TTCTTAACAAT-ATTATTCACCACAGAAGAGGT

AAGTCGTAACACGGTAAGCGTACTGGAAAGTGTGCTTGGATTAA-CAAAGTGTAGCTTAA

CTAAA-GCGTCTCGCTTACACCGAGAATATGTCTGTTAAACCCTGATCACCTTGAACGCC

TAAAACCTAGCTTTAAAAACTCA---TATGTAACTCC-TTTAACTCTTTGAC---ACCAA

TAAAACATTTTTCATTACTTAGTAGAGGCGATCAAAAAATATCTT-AAAGCTACAAAAA-

TAGTACCGCAAGGGAAAGGTGAAATAGAAATGAAACAATTTTTAAGCTATAAGAAGCAGA

GTTTTAATCTCGTACCTTTTGCATCATGGTTTAGCTAGTCTAACCAAGCAAAACGCATTT

TAAGTTTGACCCCCCGAAATTAAGCGAGCTACTTCAAAACAGCCTTTTAGGGCAAACCCG

TCTCTGTTGCAAAAGAGTGGGAAGATTTT-CAAGTAGGGGTGATAAACCTACCGAGCTTA

AAGATAGCTGGTTATTCAGGAAAAGGATTTTAGTCCTACCTTAAGTTTTTT--TATAACT

TCTAATATTTATTATAAACTTAAGAGCTATTCAAATAAGGTACAGCCTATTTGAATTAGG

GTACAACCTCCTACG---

---------------------------TATTATACTTTTTTAACCTAA-TTTCTACC-CC

CTAACGAAACCCTCCAACC--ATACTGAATGACTTCATA-TTATATGAAGAACCATATGC

TAAAACTAGTAACAAGAAGA-AGACCTTCTCTA-AAATGCAAGCGTAAGTCATAATGAAC

AACTCACTGACACTTAACGTTTATGAACACTAAATAGCAACTCA----TCAAGAAAATAC

TAT------------------TTAACCA-CACGTCAACCTTACACTAGAGCATTTCCAGA

AAGATTAAAAGAAGTGGAAGGAACTCGGCAAACA--TTAACCTCGCCTGTTTACCAAAAA

CATCGCCTCCTGACTAACT---ATAGGAGGTCCAGCCTGCCCAGTGACCAA--GTTAAAC

GGCCGCGGTACCCTAACCGTGCAAAGGTAGCGCAATCACTTGTTCTTTAAATGAGGACTA

GTATGAACGGCATCACGAGGGTTATACTGTCTCCCCCCTCTAATCAGTGAAACTGATCTC

CCCGTGAAGAAGCGGGGATAAACTTATAAGACGAGAAGACCCCATGGAGCTTTAAACTCA

GTATCACCTGCCTCCTA-CCACAACCTATTAATTAT-GCAG-TTTTGAT-TACTAGTTTT

CGGTTGGGGTGACCGCGGAGTAAAACAAAACCTCCTCGATGAACGGAACTATCCTCCTTA

TCCAAGAGTTACAACTCTAAGAATCAAT--------AAATTGACAAAAATTGATCCAAAT

AATTTTGATCAACGAACCAAGTTACCCTGGGGATAACAGCGCAATCTACTTCAAGAGCTC

CTATCGACAAGTGGGTTTACG-ACC-TCGATGTTGGATCAGGGTATCCCAGTG-GCGCAG

CCGCTA-CTAA-AGGTTC

>M. marmorata MN534751, MN534536, MN534642

TTATTGTCAACTATTTATCAACTTACACATGCAAGTCTCAGCATCCCTGTGAGAACGCCC

TTTTACCTCATCAAGGAAAAGGAGCTGGTATCAGGCACAG-----TA-----TCTCGCCC

ACAACACCTAGCTTTGCCACACCCCCAAGGGTACTCAGCAGTGATTAACATTGATTATGA

GCGCCAGCTCGACTCAGTTAGATAAAAT--AGAGCCGGCTAATCCGGTGCCAGCCGCCGC

GGCTACACCATCAGGCCCAAGCTGACAATCATTCGGCGTTAAGCGTGTTTAAAGTGTTTC

CCCCACATTAGAATTAAACTTAAACCAAGTCGTGACACACTTGTTTATAAGAAAGCCTCA
AACGAAAGTTATTCTAACCAAACCACTTGAATCCACGACAGCTAGGACACAAACTGGGAT
TAGGTACCCCACTATGCCTAGCCGTAAAATATTTACTTACACCA--AACCCGCCCGGGAA
TTACGAGCTCAAGCTTAAAACCCAAAGGACTTGACGGTGTCCCACCCATCTAGAGGAGCC
TGTTCTATAA-TCGATTCTCCCC-GATATACCTCACCATATCTAGCCTT--TCAGCCTGT
ATACCTCCGTCGCAAGCTTACCATATGAACGCA-TCCTAGTGAGCTTAAATAT--ATATC
ATAAATACGTCAGGTCAAGGTGCAGCCAACGTCATGGAAAGTAATGGGCTACAATTTCTA
CCA---TAGAACAAA-CGAAAGACTACATGCAA-CTTAGTCATGAAGGCGGATTTAGCAG
TAAAAAGAAAATAGAGAGTTCTTTTTAATTAGGCACTGGGACGCGTACACACCGCCCGTC
ACCCTCTTCAAATGAAACTACTAAAG-TTCTTAACAAT-ATTATTCACCACAGAAGAGGT
AAGTCGTAACACGGTAAGCGTACTGGAAAGTGTGCTTGGATTAA-CAAAGTGTAGCTTAA
CTAAA-GCGTCTCGCTTACACCGAGAATATGTCTGTTAAACCCTGATCACCTTGAACGCC
TAAAACCTAGCTTTAAAAACTTA---TATATAACTCC-TTTAACTCTTTGAC---ACCAA
TAAAACATTTTTCATTACTTAGTAGAGGCGATCAAAAAATATCTT-AAAGCTACAAAAA-
TAGTACCGCAAGGGAAAGGTGAAATAGAAATGAAACAATTTTTAAGCTATAAGAAGCAGA
GTTTTAATCTCGTACCTTTTGCATCATGGTTTAGCTAGTCTAACCAAGCAAAACGCATTT
TAAGTTTGACCCCCCGAAATTAAGCGAGCTACTTCAAAACAGCCTTTTAGGGCAAACCCG
TCTCTGTTGCAAAAGAGTGGGAAGATTTTTCAAGTAGGGGTGATAAACCTACCGAGCTTA
AAGATAGCTGGTTATTCAGGAAAAGGATTTTAGTCCTACCTTAAGTTTTTT-TTATAACT
TCTAATATTTAT--
---AAAGCAG
CCATCTTTC-AAAAAGCGTTAAAGCTTTATTATACTTTTTTAACCTAA-TTTCTACC-CC
CTAACGAAACCCTCCAACC--ATACTGAATGACTTCAT--TTATATGAAGAACCATATGC
TAAAACTAGTAACAAGAAGA-AGACCTTCTCTA-AAATGCAAGCGTAAGTCATAATGAAC
AACTCACTGACACTTAACGTTTATGAACACTAAATAGCAACTCA----CCAAGAAAATAC
TAT------------------TTAACCA-CACGTCAACCTTACACTAGAGCATTTCCAGA
AAGATTAAAAGAAGTGGAAGGAACTCGGCAAACA--TTAACCTCGCCTGTTTACCAAAAA
CATCGCCTCCTGACTAACT---ATAGGAGGTCCAGCCTGCCCAGTGACCAA--GTTAAAC
GGCCGCGGTACCCTAACCGTGCAAAGGTAGCGCAATCACTTGTTCTTTAAATGAGGACTA
GTATGAACGGCATCACGAGGGTTATACTGTCTCCCCCCTCTAATCAGTGAAACTGATCTC

CCCGTGAAGAAGCGGGGATAAACTTATAAGACGAGAAGACCCCATGGAGCTTTAAACTCA

GTATCACCTGCCTCCCA-CCACAACCTATTAATTAT-GCAG-TTTTGAT-TACTAGTTTT

CGGTTGGGGTGACCGCGGAGTAAAACAAAACCTCCTCGATGAACGGAACTATCCTCCTTA

TCCAAGAGTTACAACTCTAAGAATCAAT--------AAATTGACAAAAATTGATCCAAAT

AATTTTGATCAACGAACCAAGTTACCCTGGGGATAACAGCGCAATCTACTTCAAGAGCTC

CTATCGACAAGTGGGTTTACG-ACC-TCGATGTTGGATCAGGGTATCCCAGTG-GCGCAG

CCGCTA-CTAA-AGGTTC

>M. marmorata MN534752, MN534537, MN534643

TTATTGTCAACTATTTATCAACTTACACATGCAAGTCTCAGCATCCCTGTGAGAACGCCC

TTTTACCTCAACAAGGAAAAGGAGCTGGTATCAGGCACAG-----TA-----TCTCGCCC

ACAACACCTAGCTTTGCCACACCCCCAAGGGTACTCAGCAGTGATTAACATTGATTATGA

GCGCCAGCTCGACTCAGTTAGATAAAAT--AGAGCCGGCTAATCCGGTGCCAGCCGCCGC

GGCTACACCATCAGGCCCAAGCTGACAATCATTCGGCGTTAAGCGTGTTTAAAGTGCTTC

CCCCATATTAGAATTAAACTTAAACCAAGTCGTGACACACTTGTTTATAAGAAAGCCTCA

AACGAAAGTTATTCTAACCAAACCACTTGAATCCACGACAGCTAGGACACAAACTGGGAT

TAGGTACCCCACTATGCCTAGCCGTAAAATATTTACTTACACCA--AACCCGCCGGGGAA

TTACGAGCTCAAGCTTAAAACCCAAAGGACTTGACGGTGTCCCACCCATCTAGAGGAGCC

TGTTCTATAA-TCGATTCTCCCC-GATATACCTCACCATATCTAGCCTT--TCAGCCTGT

ATACCTCCGTCGCAAGCTTACCATATGAACGCA-TCCTAGTGAGCTTAAATAT--ATATC

ATAAATACGTCAGGTCAAGGTGCAGCCAACGTCATGGAAAGTAATGGGCTACAATTTCTA

CCA---TAGAACAAA-CGAAAGACTACATGCAA-CTTAGTCATGAAGGCGGATTTAGCAG

TAAAAAGAAAATAGAGAGTTCTTTTTAATTAGGCACTGGGACGCGTACACACCGCCCGTC

ACCCTCTTCAAATGAAACTACTGAAG-TCCTTAACAAT-ATTATTCACCACAGAAGAGGT

AAGTCGTAACACGGTAAGCGTACTGGAAAGTGTGCTTGGATTAA-CAAAGTGTAGCTTAA

CTAAA-GCGTCTCGCTTACACCGAGAATATGTCTGTTAAACCCTGATCACCTTGAACGCC

TAAAACCTAGCTTTAAAAACTCA---TATGTAACCCC-TTTAACTCTTTGAC---ACCAA

TAAAACATTTTTCATTACTTAGTAGAGGCGATCAAAAAATATCTT-AAAGCTACAAAAA-

TAGTACCGCAAGGGAAAGGTGAAATAGAAATGAAACAATTTTTAAGCCGTAAGAAGCAGA

GTTTTAATCTCGTACCTTTTGCATCATGGTTTAGCTAGTCTAACCAAGCAAAACGCATTT

TAAGTTTGACCCCCCGAAATTAAGCGAGCTACTTCAAAACAGCCTTTTAGGGCAAACCCG

TCTCTGTTGCAAAAGAGTGGGAAGATTTTTCAAGTAGAGGTGATAAACCTACCGAGCTTA

AAGATAGCTGGTTATTCAGGAAAAGGATTTTAGTCCTACCTTAAGTTTCTT--TATAACT

TCTAATATTTAT--

---AAAGCAG

CCATCTTTC-AAAAAGCGTTAAAGCTTTATTATACTTTTTTAACATAA-TTTCTACC-CC

CTAACGAAACCCTCCAACC--ATACTGAATGACTTCATA-TTATATGAAGAACCATATGC

TAAAACTAGTAACAAGAAGA-AGACCTTCTCTA-AAATGCAAGCGTAAGTCATAATGAAC

AACTCACTGACACTTAACGTTTATGAACACTAAATAGCAACTCA----TCAAGAAAATAC

TAT------------------TTAACCA-TACGTCAACCTTACACCAGAGCATTTCCAGA

AAGATTAAAAGAAGTGGAAGGAACTCGGCAAACA--TTAACCTCGCCTGTTTACCAAAAA

CATCGCCTCCTGACTAACT---ATAGGAGGTCCAGCCTGCCCAGTGACCAA--GTTAAAC

GGCCGCGGTACCCTAACCGTGCAAAGGTAGCGCAATCACTTGTTCTTTAAATGAGGACTA

GTATGAACGGCATCACGAGGGTTATACTGTCTCCCCCCTCTAATCAGTGAAACTGATCTC

CCCGTGAAGAAGCGGGGATAAGCTTATAAGACGAGAAGACCCCATGGAGCTTTAAACTCA

GTATCACCTGCCTCCTG-CCACAACCTATTAATTAT-GCAG-TTTTGGT-TACTAGTTTT

CGGTTGGGGTGACCGCGGAGTAAAACAAAACCTCCTCGATGAACGGAGCTATCCTCCTTA

TCCAAGAGTTACAACTCTAAGAATTAAT--------AAATTGACAAAAATTGATCCAAAT

AATTTTGATCAACGAACCAAGTTACCCTGGGGATAACAGCGCAATCTACTTCAAGAGCTC

CTATCGACAAGTGGGTTTACG-ACC-TCGATGTTGGATCAGGGTATCCCAGTG-GCGCAG

CCGCTA-CTAA-AGGTTC

>M. marmorata MN534753, MN534538, MN534644

TTATTGTCAACTATTTATCAACTTACACATGCAAGTCTCAGCATCCCTGTGAGAACGCCC

TTTTACCTCATCAAGGAAAAGGAGCTGGTATCAGGCACAG-----TA-----TCTCGCCC

ACAACACCTAGCTTTGCCACACCCCCAAGGGTACTCAGCAGTGATTAACATTGATTATGA

GCGCCAGCTCGACTCAGTTAGATAAAAT--AGAGCCGGCTAATCCGGTGCCAGCCGCCGC

GGCTACACCATCAGGCCCAAGCTGACAATCATTCGGCGTTAAGCGTGTTTAAAGTGTTTC

CCCTATATTAGAATTAAACTTAAACCAAGTCGTGACACACTTGTTTATAAGAAAGCCTCA

AACGAAAGTTATTCTAACCAAACCACTTGAATCCACGACAGCTAGGACACAAACTGGGAT

TAGGTACCCCACTATGCCTAGCCGTAAAATATTTACTTACACCA--AACCCGCCGGGGAA

TTACGAGCTCAAGCTTAAAACCCAAAGGACTTGACGGTGTCCCACCCATCTAGAGGAGCC

TGTTCTATAA-TCGATTCTCCCC-GATATACCTCACCATATCTAGCCTT--TCAGCCTGT

ATACCTCCGTCGCAAGCTTACCATATGAACGCA-TCCTAGTGAGCTTAAATAT--ATATC

ATAAATACGTCAGGTCAAGGTGCAGCCAACGTCATGGAAAGTAATGGGCTACAATTTCTA

CCA---TAGAACAAA-CGAAAGACTACATGCAA-CTTAGTCATGAAGGCGGATTTAGCAG

TAAAAAGAAAATAGAGAGTTCTTTTTAATTAGGCACTGGGACGCGTACACACCGCCCGTC

ACCCTCTTCAAATGAAACTACTAAAG-TTCTTAACAAT-ATTATTCACCACAGAAGAGGT

AAGTCGTAACACGGTAAGCGTACTGGAAAGTGTGCTTGGATTAA-CAAAGTGTAGCTTAA

CTAAA-GCGTCTCGCTTACACCGAGAATATGTCTGTTAAACCCTGATCACCTTGAACGCC

TAAAACCTAGCTTTAAAAACTCA---TATGTAACTCC-TTTAACTCTTTGAC---ACCAA

TAAAACATTTTTCATTACTTAGTAGAGGCGATCAAAAAATATCTT-AAAGCTACAAAAA-

TAGTACCGCAAGGGAAAGGTGAAATAGAAATGAAACAATTTTTAAGCTATAAGAAGCAGA

GTTTTAATCTCGTACCTTTTGCATCATGGTTTAGCTAGTCTAACCAAGCAAAACGCATTT

TAAGTTTGACCCCCCGAAATTAAGCGAGCTACTTCAAAACAGCCTTTTAGGGCAAACCCG

TCTCTGTTGCAAAAGAGTGGGAAGATTTT-CAAGTAGAGGTGATAAACCTACCGAGCTTA

AAGATAGCTGGTTATTCAGGAAAAGGATTTTAGTCCTACCTTAAGTTTTTT-TTATAACT

TCTAATATTTAT--

---AAAGCAG

CCATCTTTCAAAAAAGCGTTAAAGCTTTATTATACTTTTTTAACCTAA-TTTCTACC-CC

CTAACGAAACCCTCCAACC--ATACTGAATGACTTCATA-TTATATGAAGAACCATATGC

TAAAACTAGTAACAAGAAGA-AGACCTTCTCTA-AAATGCAAGCGTAAGTCATAATGAAC

AACTCACTGACACTTAACGTTTATGAACACTAAATAGCAACTCA----TCAAGAAAATAC

TAT------------------TTAACCA-CACGTCAACCTTACACTAGAGCATTTCCAGA

AAGATTAAAAGAAGTGGAAGGAACTCGGCAAACA--TTAACCTCGCCTGTTTACCAAAAA

CATCGCCTCCTGACTAACT---ATAGGAGGTCCAGCCTGCCCAGTGACCAA--GTTAAAC

GGCCGCGGTACCCTAACCGTGCAAAGGTAGCGCAATCACTTGTTCTTTAAATGAGGACTA

GTATGAACGGCATCACGAGGGTTATACTGTCTCCCCCCTCTAATCAGTGAAACTGATCTC

CCCGTGAAGAAGCGGGGATAAACTTATAAGACGAGAAGACCCCATGGAGCTTTAAACTCA

GTATCACCTGCCTCCTA-TCACAACCTATTAATTAT-GCAG-TTTTGAT-TACTAGTTTT

CGGTTGGGGTGACCGCGGAGTAAAACAAAACCTCCTCGATGAACGGAACTATCCTCCTTA

TCCAAGAGTTACAACTCTAAGAATCAAT--------AAATTGACAAAAATTGATCCAAAT

AATTTTGATCAACGAACCAAGTTACCCTGGGGATAACAGCGCAATCTACTTCAAGAGCTC

CTATCGACAAGTGGGTTTACG-ACC-TCGATGTTGGATCAGGGTATCCCAGTG-GCGCAG

CCGCTA-CTAA-AGGTTC

>M. marmorata MN534754, MN534645

TTATTGTCAACTATTTATCAACTTACACATGCAAGTCTCAGCATCCCTGTGAGAACGCCC

TTTTACCTCATCAAGGAAAAGGAGCTGGTATCAGGCACAG-----TA-----TCTCGCCC

ACAACACCTAGCTTTGCCACACCCCCAAGGGTACTCAGCAGTGATTAACATTGATTATGA

GCGCCAGCTCGACTCAGTTAGATAAAAT--AGAGCCGGCTAATCCGGTGCCAGCCGCCGC

GGCTACACCATCAGGCCCAAGCTGACAATCATTCGGCGTTAAGCGTGTTTAAAGTGTTTC

CCCCCTATTAAAATTAAACTTAAACCAAGTCGTGGCACACTTGTTTATAAAAAAGCCTCC

AACGAAAGTTATTCTAACCAAACCACTTGAATCCACGACAGCTAGGAAACAAACTGGGAT

TAAGTACCCCACTATGCCTAACCGTAAAATATTTACTTACACCA--AACCCGCCGGGGAA

TTACTAGCTCTAGCTTAAAACCCAAAGGACTTGACGGTGTCCCACCCATCTAGAGGAGCC

TGTTCTATAA--

---AAAGCAG

CCATCTTTC-AAAAAGCGTTAAAGCTTTATTATACTTTTTTAACCTAA-TTTCTACC-CC

CTAACGAAACCCTCCAACC--ATACTGAATGACTTCATA-TTATATGAAGAACCATATGC

TAAAACTAGTAACAAGAAGA-AGACCTTCTCTA-AAATGCAAGCGTAAGTCATAATGAAC

AACTCACTGACACTTAACGTTTATGAACACTAAATAGCAACTCA----TCAAGAAAATAC

TAT------------------TTAACCA-CACGTCAACCTTACACTAGAGCATTTCCAGA

AAGATTAAAAGAAGTGGAAGGAACTCGGCAAACA--TTAACCTCGCCTGTTTACCAAAAA

CATCGCCTCCTGACTAACT---ATAGGAGGTCCAGCCTGCCCAGTGACCAA--GTTAAAC

GGCCGCGGTACCCTAACCGTGCAAAGGTAGCGCAATCACTTGTTCTTTAAATGAGGACTA

GTATGAACGGCATCACGAGGGTTATACTGTCTCCCCCCTCTAATCAGTGAAACTGATCTC

CCCGTGAAGAAGCGGGGATAAACTTATAAGACGAGAAGACCCCATGGAGCTTTAAACTCA

GTATCACCTGCCTCCTA-CCACAACCTATTAATTAT-GCAG-TTTTGGT-TACTAGTTTT

CGGTTGGGGTGACCGCGGAGTAAAACAAAACCTCCTCGATGAACGGAACTATCCTCCTTA

TCCAAGAGTTACAACTCTAAGAATCAAT--------AAATTGACAAAAATTGATCCAAAT

AATTTTGATCAACGAACCAAGTTACCCTGGGGATAACAGCGCAATCTACTTCAAGAGCTC

CTATCGACAAGTGGGTTTACG-ACC-TCGATGTTGGATCAGGGTATCCCAGTG-GCGCAG

CCGCTA-CTAA-AGGTTC

>M. pulverata MN534755, MN534539, MN534646

TTATTGTCAACTATTTATCAACTTACACATGCAAGTCTCAGCATCCCTGTGAGAACGCCC

TTTTACCTCATCAAGGAAAAGGAGCTGGTATCAGGCACAG-----TA-----TCTCGCCC

ACAACACCTAGCTTTGCCACACCCCCAAGGGTACTCAGCAGTGATTAACATTGATTATGA

GCGCCAGCTCGACTCAGTTAGATAAAAT--AGAGCCGGCTAATCCGGTGCCAGCCGCCGC

GGCTACACCATCAGGCCCAAGCTGACAATCATTCGGCGTTAAGCGTGTTTAAAGTGTTTC

CCCTATATTAGAATTAAACTTAAACCAAGTCGTGACACACTTGTTTATAAGAAAGCCTCA

AACGAAAGTTATTCTAACCAAACCACTTGAATCCACGACAGCTAGGACACAAACTGGGAT

TAGGTACCCCACTATGCCTAGCCGTAAAATATTTACTTACACCA--AACCCGCCGGGGAA

TTACGAGCTCAAGCTTAAAACCCAAAGGACTTGACGGTGTCCCACCCATCTAGAGGAGCC

TGTTCTATAA-TCGATTCTCCCC-GATATACCTCACCATATCTAGCCTT--TCAGCCTGT

ATACCTCCGTCGCAAGCTTACCATATGAACGCA-TCCTAGTGAGCTTAAATAT--ATATC

ATAAATACGTCAGGTCAAGGTGCAGCCAACGTCATGGAAAGTAATGGGCTACAATTTCTA

CCA---TAGAACAAA-CGAAAGACTACATGCAA-CTTAGTCATGAAGGCGGATTTAGCAG

TAAAAAGAAAATAGAGAGTTCTTTTTAATTAGGCACTGGGACGCGTACACACCGCCCGTC

ACCCTCTTCAAATGAAACTACTAAAG-TTCTTAACAAT-ATTATTCACCACAGAAGAGGT

AAGTCGTAACACGGTAAGCGTACTGGAAAGTGTGCTTGGATTAA-CAAAGTGTAGCTTAA

CTAAA-GCGTCTCGCTTACACCGAGAATATGTCTGTTAAACCCTGATCACCTTGAACGCC

TAAAACCTAGCTTTAAAAACTCA---TATGTAACTCC-TTTAACTCTTTGAC---ACCAA

TAAAACATTTTTCATTACTTAGTAGAGGCGATCAAAAAATATCTTTAAAGCTACAAAAA-

TAGTACCGCAAGGGAAAGGTGAAATAGAAATGAAACAATTTTTAAGCTATAAGAAGCAGA

GTTTTAACCTCGTACCTTTTGCATCATGGTTTAGCTAGTCTAACCAAGCAAAACGCATTT

TAAGTTTGACCCCCCGAAATTAAGCGAGCTACTTCAAAACAGCCTTTTAGGGCAAACCCG

TCTCTGTTGCAAAAGAGTGGGAAGATTTT-CAAGTAGGGGTGATAAACCTACCGAGCTTA

AAGATAGCTGGTTATTCAGGAAAAGGATTTTAGTCCTACCTTAAGTTTTTT--TATAACT

TCTAATATTTATCATAAACTTAAGAGCTATTCAAATAAGGTACAGCCTATTTGAATTAGG

GTACAACCTCCTACG--------------------------------------AAAGCAG

CCATCTTTC-AAAAAGCGTTAAAGCTTTATTGTACTTTTT-AACCTAA-TTTCTACC-CC

CTAACGAAACCCTCCAACC--ATACTGAATGACTTCATA-TTATATGAAGAACCATATGC

TAAAACTAGTAACAAGAAGA-AGACCTTCTCTA-AAATGCAAGCGTAAGTCATAATGAAC

AACTCACTGACACTTAACGTTTATGAACACTAAATAGCAACTCA----TCAAGAAAATAC

TAT------------------TTAACCA-CACGTCAACCTTACACTAGAGCATTTCCAGA

AAGATTAAAAGAAGCGGAAGGAACTCGGCAAACA--TTAACCTCGCCTGTTTACCAAAAA

CATCGCCTCCTGACTAACT---ATAGGAGGTCCAGCCTGCCCAGTGACCAA--GTTAAAC

GGCCGCGGTACCCTAACCGTGCAAAGGTAGCGCAATCACTTGTTCTTTAAATGAGGACTA

GTATGAACGGCATCACGAGGGTTATACTGTCTCCCCCCTCTAATCAGTGAAACTGATCTC

CCCGTGAAGAAGCGGGGATAAACTTATAAGACGAGAAGACCCCATGGAGCTTTAAACTCA

GTATCACCTGCCTCCTA-CCACAACCTATTAATTAT-GCAG-TTTTGAT-TACTAGTTTT

CGGTTGGGGTGACCGCGGAGTAAAACAAAACCTCCTCGATGAACGGAACTATCCTCCTTA

TCCAAGAGTTACAACTCTAAGAATCAAT--------AAATTGACAAAAATTGATCCAAAT

AATTTTGATCAACGAACCAAGTTACCCTGGGGATAACAGCGCAATCTACTTCAAGAGCTC

CTATCGACAAGTGGGTTTACG-ACC-TCGATGTTGGATCAGGGTATCCCAGTG-GCGCAG

CCGCTA-CTAA-AGGTTC

>M. pulverata MN534756, MN534540, MN534647

TTATTGTCAACTATTTATCAACTTACACATGCAAGTCTCAGCATCCCTGTGAGAACGCCC

TTTTACCTCATTAAGGAAAAGGAGCTGGTATCAGGCACAG-----TA-----TCTCGCCC

ACAACACCTAGCTTTGCCACACCCCCAAGGGTACTCAGCAGTGATTAACATTGATTATGA

GCGCCAGCTCGACTCAGTTAGATAAAAT--AGAGCCGGCTAATCCGGTGCCAGCCGCCGC

GGCTACACCATCAGGCCCAAGCTGACAATCATTCGGCGTTAAGCGTGTTTAAAGTGTTTC

CCCTATATTAGAATTAAACTTAAACCAAGTCGTGACACACTTGTTTATAAGAAAGCCTCA

AACGAAAGTTATTCTAACCAAACCACTTGAATCCACGACAGCTAGGACACAAACTGGGAT

TAGGTACCCCACTATGCCTAGCCGTAAAATATTTACTTACACCA--AACCCGCCGGGGAA

TTACGAGCTCAAGCTTAAAACCCAAAGGACTTGACGGTGTCCCACCCATCTAGAGGAGCC

TGTTCTATAA-TCGATTCTCCCC-GATATACCTCACCATATCTAGCCTT--TCAGCCTGT

ATACCTCCGTCGCAAGCTTACCATATGAACGCA-TCCTAGTGAGCTTAAATAT--ATATC

ATAAATACGTCAGGTCAAGGTGCAGCCAACGTCATGGAAAGTAATGGGCTACAATTTCTA

CCA---TAGAACAAA-CGAAAGACTACATGCAA-CTTAGTCATGAAGGCGGATTTAGCAG

TAAAAAGAAAATAGAGAGTTCTTTTTAATTAGGCACTGGGACGCGTACACACCGCCCGTC

ACCCTCTTCAAATGAAACTACTAAAG-TTCTTAACAAT-ATTATTCACCACAGAAGAGGT

AAGTCGTAACACGGTAAGCGTACTGGAAAGTGTGCTTGGATTAA-CAAAGTGTAGCTTAA

CTAAA-GCGTCTCGCTTACACCGAGAATATGTCTGTTAAACCCTGATCACCTTGAACGCC

TAAAACCTAGCTTTAAAAACTCA---TATGTAACTCC-TTTAACTCTTTGAC---ACCAA

TAAAACATTTTTCATTACTTAGTAGAGGCGATCAAAAAATATCTT-AAAGCTACAAAAA-

TAGTACCGCAAGGGAAAGGTGAAATAGAAATGAAACAATTTTTAAGCTATAAGAAGCAGA

GCTTTAATCTCGTACCTTTTGCATCATGGTTTAGCTAGTCTAACCAAGCAAAACGCATTT

TAAGTTTGACCCCCCGAAATTAAGCGAGCTACTTCAAAACAGCCTTTTAGGGCAAACCCG

TCTCTGTTGCAAAAGAGTGGGAAGATTTT-CAAGTAGGGGTGATAAACCTACCGAGCTTA

AAGATAGCTGGTTATTCAGGAAAAGGATTTTAGTCCTACCTTAAGTTTTTT--TATAACT

TCTAATATTTATTATAAACTTAAGAGCTATTCAAATAAGGTACAGCCTATTTGAATTAGG

GTACAACCTCCTACGACG-----------------------------------AAAGCAG

CCATCTTTC-AAAAAGCGTTAAAGCTTTATTATACTTTTT-AACCTAA-TTTCTACC-CC

CTAACGAAACCCTCCAACC--ATACTGAATGACTTCATA-TTATATGAAGAACCATATGC

TAAAACTAGTAACAAGAAGA-AGACCTTCTCTA-AAATGCAAGCGTAAGTCATAATGAAC

AACTCACTGACACTTAACGTTTATGAACACTAAATAGCAACTCA----TCAAGAAAATAC

TAT------------------TTAACCA-CACGTCAACCTTACACTAGAGCATTTCCAGA

AAGATTAAAAGAAGTGGAAGGAACTCGGCAAACA--TTAACCTCGCCTGTTTACCAAAAA

CATCGCCTCCTGACTAACT---ATAGGAGGTCCAGCCTGCCCAGTGACCAA--GTTAAAC

GGCCGCGGTACCCTAACCGTGCAAAGGTAGCGCAATCACTTGTTCTTTAAATGAGGACTA

GTATGAACGGCATCACGAGGGTTATACTGTCTCCCCCCTCTAATCAGTGAAACTGATCTC

CCCGTGAAGAAGCGGGGATAAACTTATAAGACGAGAAGACCCCATGGAGCTTTAAACTCA

GTATCACCTGCCTCCTA-CCACAACCTATTAATTAT-GCAG-TTTTGAT-TACTAGTTTT

CGGTTGGGGTGACCGCGGAGTAAAACAAAACCTCCTCGATGAACGGAACTATCCTCCTTA

TCCAAGAGTTACAACTCTAAGAATCAAT--------AAATTGACAAAAATTGATCCAAAT

AATTTTGATCAACGAACCAAGTTACCCTGGGGATAACAGCGCAATCTACTTCAAGAGCTC

CTATCGACAAGTGGGTTTACG-ACC-TCGATGTTGGATCAGGGTATCCCAGTG-GCGCAG

CCGCTA-CTAA-AGGTTC

>M. nanapollexa MN534757, MN534541, MN534648

TTATTGTCAGCTACTTATCTACCTACACATGCAAGTCTCAGCACCCCTGTGAGAACGCCC

TTTTACCTACACCAGGAAAAGGAGCCGGTATCAGGCACAA-----TA-----TCTTGCCC

AAGACACCTAGCTTTGCCACACCCTCAAGGGTATTCAGCAGTGATTAACATTGTTCATAA

GCGCCAGCTTGACTCAGTTAAATAAAAT--AGAGCCGGCTAATCCGGTGCCAGCCGCCGC

GGCTACACCGCTAGGCCCAAGTTGACAATTACTCGGCGTTAAGAGTGTTTTAAGTGCCC-

TAAACCATTAGAATTAAACTTTAACCAAGTTGTGACACACTTGTTCTTAAAAAAAACATA

AACGAAAGTTATTCTAATTTGACCACTTGAACCCACGACAGCTAAGACACAAACTGGGAT

TAGGTACCCCACTATGCCTAGCCGTAAAATATTAACTTACACCT--CATTCGCCAGGGAA

TTACGAGCGCAAGCTTAAAACCCAAAGGACTTGACGGTGTCCCACCCACCTAGAGGAGCC

TG---TATAA-TCGATTTTCCCCCGATTCACCTCACCACTTCTAGCCAC--TCAGCCTGT

ATACCTCCGTCGTAAGCTTACCATATGAACGCATACTTAGTGAGCCTAAAGAT--TTACC

ATAAACACGTCAGGTCAAGGTGCAGCCCACGATGTGGAAAGCAATGGGCTACAATTTCTA

TAC---TAGAACAAA-CGAAAGACTACATGAAA-CTCAGTCATGAAGGCGGATTTAGCAG

TAAAAAGAAAATAGAGTGTTCTTTTTAACAAGGCCCTGGGACGCGTACACACCGCCCGTC

ACCCTCTTCAAATGTACATA--ACAG-TTTTTAACTAC-ACTTTACTCTACAGAAGAGGT

AAGTCGTAACACGGTAAGCGTACTGGAAAGTGCGCTTGGATTAA-CAAAGTGTAGCTTAA

TTAAA-GCATCTCGCTTACACCGAGAATATGTCTACTTACTACAGACCACCTTGAACGCC

AAAAACCTAGCTTTATTTTCTCT---TATGCA-CTAC-TGTAATTTTACTAT---AAAAA
TAAAACATTTTTAATTATTTAGTAGAAGTGATCAAAAAATATTTCTAAAGCTATAAAAA-
TAGTACCGCAAGGGAAAGGTGAAATAACAATGAAATAATTTTTAAGCACAAAAAAGCAGA
GCTTAAATCTCGTACCTTTTGCATCATGGTTTAATTAGTCTAACCAAGCAAAATGAATTT
TTAGTTTGACCCCCCGAAACTAAGCGAGCTACTTTGAAACAGCCCTA-AGAGCCAACCCA
TCTCTGTTGCAAAAGAGTGGGAAGATTTT-TAAGTAGAGGTGATAAACCTACCGAGCTTA
GAGATAGCTGGTTATTCAGGAAAAGGATTTTAGTCCTACCTTAAGTTTCTT--ATTAACT
TTGATTAATTCCAC-AAACTTAAGAGTTATTCAAATAAGGTACAGCCTATTTGAAAAAGG
ATACAACCTA--

----CGAAACCCTCCACCA--ATACTGAATGACTTCATA-CTTTATGAAGGACCATATGT
TAAAACTAGTAACAAGAAGA-AGACCTTCTCCA-AAATGCAAGCGTAAGTCATAATGAAC
AATTCACTGACACTTAACGTTTATGAATCATAAGTAACAACCCA---CACAAGAAAACCT
TAC------------------CTAAATT-AACGTCAACCTTACACTAGAGCATTTCATGA
AAGATTAAAAGAAAGGGAAGGAACTCGGCAAATT--TTAACCTCGCCTGTTTACCAAAAA
CATCGCCTCCTGATAATT----ATAGGAGGTCCAGCCTGCCCAGTGACAAA--GTTAAAC
GGCCGCGGTACCCTAACCGTGCAAAGGTAGCACAATCACTTGTTCTTTAAATGAGGACTA
GTATGAACGGCATCACGAAGGTTATACTGTCTCCCCCCTCCAATCAGTGAAACTGACCTC
CCCGTGAAGAAGCGGGGATATTATTATAAGACGAGAAGACCCCATGGAGCTTTAAACTCA
GAATCACCTGCTTCAAC-CCACAGCCTCCCAACTAA-GCAG-TCCTGACTCTCTAGTTTT
CGGTTGGGGTGACCGCGGAGTAAAAATAAACCTCCACGACGAAAGGAACTACTCTCCTAA
TCCAAGAGCTACAGCTCTAAGAATCAAA--------TTATTGACAAAA-TTGATCCAAAT
TTCTTTGATCAACGAACCAAGTTACCCTGGGGATAACAGCGCAATCCATTTCAAGAGCCC
CTATCGACAAATGGGTTTACG-ACC-TCGATGTTGGATCAGGGTATCCTAGTG-GCGCAG
CCGCTA-CTAA-AGGTTC

>M. arboricola MN534758, MN534542, MN534649

TTATTGTTAACTGTTTGTCAACTTACACATGCAAGTCTCAGCATCCCCGTGAGAACGCCC
TTTCACCTCCATCAGGATAAGGAGCTGGTATCAGGCACAG-----TT-----TCTTGCCC
ACAACACCTAGCTTTGCCACACCCTCAAGGGTACTCAGCAGTGATTAACATTGTTCATGA
GCGCCAGCTCGACTCAGTTAGACAAAAC--AGAGCCGGCTAATCCGGTGCCAGCCGCCGC

GGCTACACCATTAGGCTCAAGTTTACAATTACTCGGCGTTAAGCGTGTTTTAAGTGCCT-

AAACTACTTAGAATTAAAATTTAACCAAGTTGTGACACACTTGTTCTTAAGAAAAACATA

AACGAAAGTTATTCTAACCTAACCACTTGAACCCACGACAGCTAGGATACAAACTGGGAT

TAGGTACCCCACTATGCCTAGCCGTAAAACATTAACTTACACCC-CCCACCGCCAGGGAA

TTACGAGCCCAAGCTTAAAACCCAAAGGACTTGACGGTGTCCCACCCACCTAGAGGAGCC

TGTTCTATAA-TCGATTCTCCCC-GATTTACCTCACCACTTCTAGCCTC--TCAGCCTGT

ATACCTCCGTCGTAAGCTTACCATGTGAACGCA-CCACAGTGAGCAAGAAGAT--CTTCC

ATAAATACGTCAGGTCAAGGTGCAGCCCACGATGTGGGAAGTAATGGGCTACAATTTCTA

CAAT--TAGAACAAA-CGAAAGACTACATGCAA-TTTAGTCATGAAGGCGGATTTAGTAG

TAAAAAGAAAATAGAGAGTTCTTTTTAACTAGGCACTGGGACGCGTACACACCGCCCGTC

ACCCTCTTCAAATGTAACTTCAATAG-TTTTTAACAAT-TTTGTACCACACAGAAGAGGT

AAGTCGTAACACGGTAAGCGTACTGGAAAGTGCGCTTGGATCAA-CAAAGTGTAGCTTAA

TTAAA-GCATCTCGCTTACACCGAAAATATATCTGTTTAACCCAGATCACCTTGAACGCC

TAAAACCTAGCTTTACTTCCCCA---CACATA-CTCT-CCTAACTTTTTCCC----TAAA

TAAAACATTTTTACT-ATTTAGTAGAGGCGATCAAAAAATATCTT-AAAGCTATAAAAA-

TAGTACCGCAAGGGAAAGGTGAAATAGTAATGAAATAACCCTTAAGCCAAAAAAAGCAGA

GATTAAACCTCGTACCTTTTGCATCATGGTTTAGCTAGTCTAACCAAGCAAAATGCATTA

T-AGTTTGACCCCCCGAAACTAAGCGAGCTACTTTAAAACAGCCTTA-AGGGCAAACCCG

TCTCTGTTGCAAAAGAGTGGGAAGATTTT-AAAATAGAGGTGATAAACCTACCGAGCTTA

GAGATAGCTGGTTATTCAGGAGACGGATTTAAGTCCTACCTTAAGTTTTCC--CATAACT

TCTAATACTTC---

--GCAG

CCACCTTTT-AAAAAGCGTTAAAGCTTTCTTTT--AATCTCGCATTAA-TTCCCCCA-TT

TTACCGAAACCCTCCACCC--ATACTGAATGACTTCATA-CAATATGAAAGACCATATGC

TAAAACTAGTAACAAGAAGA-AGAACTTCTCCA-AAATGCAAGCGTAAGTCATAATGAAC

AATTCACTGACAATTAACGTCTATTAGTCCTAAGTAGTAACCTC---CTCAAGAAAACCC

TAC------------------TTAAAAC-AACGTCAACCTTACACCGGAGCATTTACAGA

AAGATTAAAAGAGCCGGAAGGAACTCGGCAAATTACTTAACCCCGCCTGTTTACCAAAAA

CATCGCCTCCTGACAAACT---ATAGGAGGTCCAGCCTGCCCAGTGACAAA--GTTAAAC

GGCCGCGGTACCCTAACCGTGCAAAGGTAGCACAATCACTTGTTCTTTAAATGAGGACTA

GTATGAATGGCATCACGAGGGTTATACTGTCTCCCCCCTCTAATCAGTGAAACTGACCCC

CCCGTGAAGAAGCGGGGATACTAATATAAGACGAGAAGACCCCATGGAGCTTTAAACTCA

GTATCACCTGCCACATA-CCACAGCCTCTCACCCAC-GCAG-TTCTGAT-AACTAGTTTT

CGGTTGGGGTGACCGCGGAGTAAAACAAAACCTCCTCGATGAAAGGAATTTAATTCCTAA

TCCAAGAGCCACAACTCTAAGAATCAAA--------TTATTGACAAAAATTGATCCAAGC

ACTATTGATCAACGAACCAAGTTACCCTGGGGATAACAGCGCAATCCATTTCAAGAGCTC

ATATCGACAAATGGGTTTACG-ACC-TCGATGTTGGATCAGGGTATCCCAGTG-GCGCAG

CCGCTA-CTAA-AGGTTC

>M. arboricola MN534759, MN534543, MN534650

TTATTGTTAACTGTTTGTCAACTTACACATGCAAGTCTCAGCATCCCCGTGAGAACGCCC

TTTCACCTCCATCAGGATAAGGAGCTGGTATCAGGCACAG-----TT-----TCTTGCCC

ACAACACCTAGCTTTGCCACACCCTCAAGGGTACTCAGCAGTGATTAACATTGTTCATGA

GCGCCAGCTCGACTCAGTTAGACAAAAC--AGAGCCGGCTAATCCGGTGCCAGCCGCCGC

GGCTACACCATTAGGCTCAAGTTTACAATTACTCGGCGTTAAGCGTGTTTTAAGTGCCT-

AAACTACTTAGAATTAAAATTTAACCAAGTTGTGACACACTTGTTCTTAAGAAAAACATA

AACGAAAGTTATTCTAACCTAACCACTTGAACCCACGACAGCTAGGATACAAACTGGGAT

TAGGTACCCCACTATGCCTAGCCGTAAAACATTAACTTACACCC-CCCCCCGCCAGGGAA

TTACGAGCCCAAGCTTAAAACCCAAAGGACTTGACGGTGTCCCACCCACCTAGAGGAGCC

TGTTCTATAA-TCGATTCTCCCC-GATTTACCTCACCACTTCTAGCCTC--TCAGCCTGT

ATACCTCCGTCGTAAGCTTACCATGTGAACGCA-CCACAGTGAGCAAGAAGAT--CTTCC

ATAAATACGTCAGGTCAAGGTGCAGCCCACGATGTGGGAAGTAATGGGCTACAATTTCTA

CAAT--TAGAACAAA-CGAAAGACTACATGCAA-TTTAGTCATGAAGGCGGATTTAGTAG

TAAAAAGAAAATAGAGAGTTCTTTTTAACTAGGCACTGGGACGCGTACACACCGCCCGTC

ACCCTCTTCAAATGTAACTTCAATAG-TTTTTAACAAT-TTTGTACCACACAGAAGAGGT

AAGTCGTAACACGGTAAGCGTACTGGAAAGTGCGCTTGGATCAA-CAAAGTGTAGCTTAA

TTAAA-GCATCTCGCTTACACCGAAAATATATCTGTTAAACCCAGATCATCTTGAACGCC

TAAAACCTAGCTTTACTTCCCCA---CACATA-CTCT-CCTAACTTTTTCCC----TAAA

TAAAACATTTTTACT-ATTTAGTAGAGGCGATCAAAAAATATCTT-AAAGCTATAAAAA-

TAGTACCGCAAGGGAAAGGTGAAATAGTAATGAAATAACCCTTAAGCCAAAAAAAGCAGA

GATTAAACCTCGTACCTTTTGCATCATGGTTTAGCTAGTCTAACCAAGCAAAATGCATTA

T-AGTTTGACCCCCCGAAACTAAGCGAGCTACTTTAAAACAGCCTTA-AGGGCAAACCCG

TCTCTGTTGCAAAAGAGTGGGAAGATTTT-AAAATAGAGGTGATAAACCTACCGAGCTTA

GAGATAGCTGGTTATTCAGGAGACGGATTTAAGTCCTACCTTAAGTTTTCC--CATAACT

TCTAATACTTC---

--GCAG

CCACCTTTT-AAAAAGCGTTAAAGCTTTCTTTT--AATCTCGCATTAA-TTCCCCCA-TT

TTACCGAAACCCTCCACCC--ATACTGAATGACTTCATA-CAATATGAAGGACCATATGC

TAAAACTAGTAACAAGAAGA-AGAACTTCTCCA-AAATGCAAGCGTAAGTCATAATGAAC

AATTCACTGACAATTAACGTCTATTAGTCCTAAGTAGTAACCTC---CTCAAGAAAACCC

TAC------------------TTAAAAC-AACGTCAACCTTACACCAGAGCATTTACAGA

AAGATTAAAAGAGCCGGAAGGAACTCGGCAAATTACTTAACCCCGCCTGTTTACCAAAAA

CATCGCCTCCTGACAAACT---ATAGGAGGTCCAGCCTGCCCAGTGACAAA--GTTAAAC

GGCCGCGGTACCCTAACCGTGCAAAGGTAGCACAATCACTTGTTCTTTAAATGAGGACTA

GTATGAATGGCATCACGAGGGTTATACTGTCTCCCCCCTCTAATCAGTGAAACTGACCCC

CCCGTGAAGAAGCGGGGATACTAATATAAGACGAGAAGACCCCATGGAGCTTTAAACTCA

GTATCACCTGCCACATA-CCACAGCCTCTCACCCAC-GCAG-TTCTGAT-AACTAGTTTT

CGGTTGGGGTGACCGCGGAGTAAAACAAAACCTCCTCGATGAAAGGAATTTAATTCCTAA

TCCAAGAGCCACAACTCTAAGAATCAAA--------TTATTGACAAAAATTGATCCAAGC

ACTATTGATCAACGAACCAAGTTACCCTGGGGATAACAGCGCAATCCATTTCAAGAGCTC

ATATCGACAAATGGGTTTACG-ACC-TCGATGTTGGATCAGGGTATCCCAGTG-GCGCAG

CCGCTA-CTAA-AGGTTC

>M. arboricola MN534760, MN534544, MN534651

TTATTGTTAACTGTTTGTCAACTTACACATGCAAGTCTCAGCATCCCCGTGAGAACGCCC

TTTCACCTCTTTCAGGATAAGGAGCTGGTATCAGGCACAG-----TA-----TCTTGCCC

ACAACACCTAGCTTTGCCACACCCTCAAGGGTACTCAGCAGTGATTAACATTGTTCATGA

GCGCCAGCTCGACTCAGTCAGACAAAACTTAGAGCCGGCTAATCCGGTGCCAGCCGCCGC

GGCTACACCATCAGGCTCAAGTTTACAATTACTCGGCGTTAAGCGTGTTTTAAGTACCC-

AAAC-TGTTAGAATTAAAATTTAACCAAGTTGTGACACACTTGTTCTTAAGAAAAACATA

AACGAAAGTTATTCTAACCCAACCACTTGAACCCACGACAGCTAGGATACAAACTGGGAT

TAGGTACCCCACTATGCCTAGCCGTAAAACATTAACTTACACCCACACCCCGCCAGGGAA

TTACGAGCCCAAGCTTAAAACCCAAAGGACTTGACGGTGTCCCACCCACCTAGAGGAGCC

TGTTCTATAA-TCGATTCTCCCC-GATTCACCTCACCACTTCTAGCCTC--TCAGCCTGT

ATACCTCCGTCGTAAGCTTACCATGTGAACGCA-CCACAGTGAGCCAGAAGAT--TTACC

ATAAATACGTCAGGTCAAGGTGCAGCCCACGATGTGGAAAGTAATGGGCTACAATTTCTA

CCG---TAGAACAAA-CGAAAGACTACATGCAATCTTAGTCATGAAGGCGGATTTAGTAG

TAAAAAGAAAATAGAGAGTTCTTTTTAACTAGGCCCTGGGACGCGTACACACCGCCCGTC

ACCCTCTTCAAATGTAATCTCTATAG-TTTTTAACAAC-TTTATACCCCACAGAAGAGGT

AAGTCGTAACACGGTAAGCGTACTGGAAAGTGCGCTTGGATTAA-CAAAGTGTAGCTTAA

TTAAA-GCATCTCGCTTACACCGAAAATATCTCTGTTAAACCCAGATCACCTTGAGCGCC

TAAAACCTAGCTTTACTTACACG---CATAAA-CACCCAACAACTTTTTTCC---TACGA

TAAAACATTTTTTCT-ATTTAGTAGAGGCGATCAAAAAATATCTT-AAAGCTATAAAAG-

TAGTACCGCAAGGGAAAGGTGAAATAGTAATGAAATAACCTTTAAGCCAAAAAAAGCAGA

GATAAAACCTCGTACCTTTTGCATCATGGTTTAGCTAGTCTAACCAAGCAAAATGCATTT

T-AGTTTGACCCCCCGAAACTAAGCGAGCTACTTTAAAACAGCCTTC-AGAGCTAACCCG

TCTCTGTTGCAAAAGAGTGGGAAGATTTT-AAAGTAGAGGTGATAAACCTACCGAGCTTA

GAGATAGCTGGTTATTCAGGAAAGGGATTTAAGTCCTACCTTAAGTTTTTC--TATAACT

TCTAATACTTC---

--GCAG

CCACCTTTT-AAAAAGCGTTAAAGCTTTCTTTT---ATCTC-CACTAA-TTCCTCAA-TT

TTACCGAAACCCCCCACCC--ATACTGAATGACTTCCTA-TTATATGAAGGACCATATGC

TAAAACTAGTAACAAGAAGA-AGAACTTCTCTA-AAATGCAAGTGTAAGTCATAATGAAC

AATTCACTGACAATTAACGTCTATTGACCCAAAGTAGCAACCCC----GCAAGAAAACCC

TAC------------------TTAAAC-AACGTCAACCTTACACCAGAGCATTAACAGA

AAGATTTAAAGAGCCGGAAGGAACTCGGCAAATTA-CTAACCCCGCCTGTTTACCAAAAA

CATCCCCTCCTGATAAACC---ATAGGAGGTCCAGCCTGCCCAGTGACAAA--GTTAAAC

GGCCGCGGTACCCTAACCGTGCAAAGGTAGCACAATCACTTGTTCTTTAAATGAGGACTA

GTATGAACGGCATCACGAGGGTTATACTGTCTCCCCCCTCTAATCAGTGAAACTGACCCC

CCCGTGAAGAAGCGGGGATAAAAATATAAGACGAGAAGACCCCATGGAGCTTTAAACTCA

TTATCACCTGCCACATA-CTACAACCTCTCACCCAC-GCAG-CCCTGAT-AACTAGTTTT

CGGTTGGGGTGACCGCGGAGCAAAACAAAACCTCCTCGATGAAAGGAACTGACCTCCTAA

TCCAAGAGCCACAACTCTAAGAATCAAA--------TTATTGACAAAAATTGATCCAAAT

ACTATTGATCAACGAACCAGGTTACCCTGGGGATAACAGCGCAATCCATTTCAAGAGCTC

ATATCGACAAATGGGTTTACG-ACC-TCGATGTTGGATCAGGGTATCCCAGTG-GCGCAG

CCGCTA-CTAA-AGGTTC

>M. arboricola MN534761, MN534545, MN534652

TTATTGTTAACTGTTTGTCAACTTACACATGCAAGTCTCAGCATCCCCGTGAGAACGCCC

TTTCACCTCTTTCAGGATAAGGAGCTGGTATCAGGCACAG---ACTA-----TCTTGCCC

ACAACACCTAGCTTTGCCACACCCTCAAGGGTACTCAGCAGTGATTAACATTGTTCATGA

GCGCCAGCTCGACTCAGTTAGACAAAACTTAGAGCCGGCTAATCCGGTGCCAGCCGCCGC

GGCTACACCATCAGGCTCAAGTTTACAATTACTCGGCGTTAAGCGTGTTTTAAGTACCC-

AAAC-TGTTAGAATTAAAATTTAACCAAGTTGTGACACACTTGTTCTTAAGAAAAACATA

AACGAAAGTTATTCTAACCCAACCACTTGAACCCACGAGAGCTAGGATACAAACTGGGAT

TACGTACCCCACTATGCCTAGCCGTAAAACATTAACTTACACCCACACCCCGCCAGGGAA

TTACGAGCCCAAGCTTAAAACCCAAAGGACTTGACGGTGTCCCACCCACCTAGAGGAGCC

TGTTCTATAA-TCGATTCTCCCC-GATTCACCTCACCACTTCTAGCCTC--TCAGCCTGT

ATACCTCCGTCGTAAGCTTACCATGTGAACGCA-CCACAGTGAGCCAGAAGAT--TTACC

ATAAATACGTCAGGTCAAGGTGCAGCCCACGATGTGGAAAGTAATGGGCTACAATTTCTA

CCG---TAGAACAAA-CGAAAGACTACATGCAATCTTAGTCATGAAGGCGGATTTAGTAG

TAAAAAGAAAATAGAGAGTTCTTTTTAACTAGGCCCTGGGACGCGTACACACCGCCCGTC

ACCCTCTTCAAATGTAATCTCTATAG-TTTTTAACAAC-TTTATACCCCACAGAAGAGGT

AAGTCGTAACACGGTAAGCGTACTGGAAAGTGCGCTTGGATTAA-CAAAGTGTAGCTTAA

TTAAA-GCATCTCGCTTACACCGAAAATATCTCTGTTAAACCCAGATCACCTTGAGCGCC

TAAAACCTAGCTTTACTTACACG---CATAAA-CACCCAACAACTTTTTTCC---TACGA

TAAAACATTTTTTCT-ATTTAGTAGAGGCGATCAAAAAATATCTT-AAAGCTATAAAAG-

TAGTACCGCAAGGGAAAGGTGAAATAGTAATGAAATAACCTTTAAGCCAAAAAAAGCAGA

GATAAAACCTCGTACCTTTTGCATCATGGTTTAGCTAGTCTAACCAAGCAAAATGCATTT

T-AGTTTGACCCCCCGAAACTAAGCGAGCTACTTTAAAACAGCCTTT-AGAGCTAACCCG

TCTCTGTTGCAAAAGAGTGGGAAGATTTT-AAAGTAGAGGTGATAAACCTACCGAGCTTA

GAGATAGCTGGTTATTCAGGAAAGGGATTTAAGTCCTACCTTAAGTTTTTC--TATAACT

TCTAATACTTC---

--GGCCCTAAAAGCAG

CCACCTTTTAAAAAAGCGTTAAAGCTTTCTTTT--AATCTCACACTAA-TTCCTCAA-TT

TTACCGAAACCCCCCACCC--ATACTGAATGACTTCATA-TTATATGAAGGACCATATGC

TAAAACTAGTAACAAGAAGA-AGAACTTCTCTA-AAATGCAAGTGTAAGTCATAATGAAC

AATTCACTGACAATTAACGTCTATTGACCCAAAGTAGCAACCCC----GCAAGAAAACCC

TAC------------------TTAAAAC-AACGTCAACCTTACACCAGAGCATTAACAGA

AAGATTTAAAGAGCCGGAAGGAACTCGGCAAATTA-CTAACCCCGCCTGTTTACCAAAAA

CATCGCCTCCTGATAAACC---ATAGGAGGTCCAGCCTGCCCAGTGACAAA--GTTAAAC

GGCCGCGGTACCCTAACCGTGCAAAGGTAGCACAATCACTTGTTCTTTAAATGAGGACTA

GTATGAACGGCATCACGAGGGTTATACTGTCTCCCCCCTCTAATCAGTGAAACTGACCCC

CCCGTGAAGAAGCGGGGATAAAAATATAAGACGAGAAGACCCCATGGAGCTTTAAACTCA

GTATCACCTGCCACATA-CTACAACCTCTCACCCAC-GCAG-CCCTGAT-AACTAGTTTT

CGGTTGGGGTGACCGCGGAGCAAAACAAAACCTCCTCGATGAAAGGAACTGACCTCCTAA

TCCAAGAGCCACAACTCTAAGAATCAAA--------TTATTGACAAAAATTGATCCAAAT

ACTATTGATCAACGAACCAAGTTACCCTGGGGATAACAGCGCAATCCATTTCAAGAGCCC

ATATCGACAAATGGGTTTACG-ACC-TCGATGTTGGATCAGGGTATCCCAGTG-GCGCAG

CCGCTA-CTAA-AGGTTC

>M. pulchella MN534762, MN534546, MN534653

TTATTGTTAGCTGTTTGTCGACTTACACATGCAAGTCTCAGCATCCCTGTGAGAACGCCC

TTTCACCTCTATCAGGATAAGGAGCTGGTATCAGGCACAG---ACTA-----TCTTGCCC

ACAACACCTAGCTCTGCCACACCCTCAAGGGTACTCAGCAGTGATTAACATTGTTTATGA

GCGCCAGCTCGACTCAGTCAGACAAAGTA-AGAGCCGGCTAATCCGGTGCCAGCCGCCGC

GGCTACACCATTAGGCTCAAGTTTACAATTATTCGGCGTTAAGCGTGTTTTAAGTTTCC-

TCATTTATTAGAATTAAAATTTAACCAAGTTGTGACACACTTGTTCATAAGAAAAACATA

AACGAAAGTTATTCTAACCAAACCACTTGAATCCACGACAGCTAGGACACAAACTGGGAT

TAGGTACCCCACTATGCCTAGCCGTAAAATATTAACCTACACCT-TCCTTCGCCAGGGAA

TTACGAGCCCAAGCTTAAAACCCAAAGGACTTGACGGTGTCCCACCCATCTAGAGGAGCC

TGTTCTATAA-TCGATTCTCCCC-GATTCACCTCACCACTTCTAGCCTC--TCAGCCTGT

ATACCTCCGTCGTAAGCTTACCATGTGAACGCA-CCATAGTGAGCCCAAAGAT--TTCCC

ATAAATACGTCAGGTCAAGGTGCAGCCCACGATGTGGAAAGTAATGGGCTACAATTTCTA

TCAT--TAGAACAAA-CGAAAGACTACATGCAA-TTCAGTCATGAAGGCGGATTTAGTAG

TAAAAAGAAAATAGAGAGTTCTTTTTAACTAGGCACTGGGACGCGTACACACCGCCCGTC

ACCCTCTTCAAATGTAACTTCCCACGGTTTTTAACAAT-TCCATACCCCACAGAAGAGGT

AAGTCGTAACACGGTAAGCGTACTGGAAAGTGCGCTTGGATTAA-CAAAGTGTAGCTTAA

TTAAA-GCATCTCGCTTACACCGAAAATATATCTGTTAAACCCGGATCACCTTGAA-GCC

TAAAACCTAGCTTTACTTCCTCG---CATAGA-CACT-CTTAACTTTTCTTC---AACAA

TAAAACATTTTTAATTATTTAGTACAGGCGATCAAAAAATACCTT-AAAGCTACAAAAA-

TAGTACCGCAAGGGAAAGGTGAAATAGTAATGAAATAACTCTTAAGCCAAAAAAAGCAGA

GATAAAACCTCGTACCTTTTGCATCATGGTTTAGCAAGTCTAACCAAGCAAAACGCATTT

TTAGTTTGACCCCCCGAAACTAAGCGAGCTACTTTAAAACAGCTTTT-AGAGCAAACCCG

TCTCTGTTGCAAAAGAGTGGGAAGATTTT-AAAGTAGAGGTGATAAACCTACCGAGCTTA

GAGATAGCTGGTTATTCAGGAAAGGGATTTAAGTCCTACCTTAAGTCTCCT--TATAATT

TCTAATACTTCAAT-AGACTTAAGAGCCATTCAAATAAGGTACAGCCTATTTGAACCAGG

GTACAACCTACT-------------------------------------CCTAAAAGCAG

CCACCTTTG-AAAAAGCGTTAAAGCTTTATTTC--AATCTCTCATCAA-TTCCTCAA-TT

TTACCGAAACCCCCCACCCCCATACTGAATGACTTCATA-CTTTATGAAGGACCATATGC

TAAAACTAGTAACAAGAAGA-AGAACTTCTCCA-AAATGCAAGCGTAAATCATAATGAAC

AACTCACTGACAATTAACGTCCATAAGCCCAAAGTAGTAACTCA----CCAAGAAAACTC

TAC------------------TTAAAAC-AACGTTAACCTTACACCAGCGCATTCACAGA

AAGATTAAAAGAATCGGAAGGAACTCGGCAAATTA-CTAACCCCGCCTGTTTACCAAAAA

CATCGCCTCCTGATAAACT---ATAGGAGGTCCAGCCTGCCCAGTGACAAA--GTTAAAC

GGCCGCGGTACCCTAACCGTGCAAAGGTAGCGCAATCACTTGTTCTTTAAATGAGGACTA

GTATGAATGGCATCACGAGGGTTATACTGTCTCCCCACTCTAATCAGTGAAACTGACCTC

CCCGTGAAGAAGCGGGGATATTAATATAAGACGAGAAGACCCCATGGAGCTTTAAACTCA

GTATCACCTGCCACACA-TTACAACCTCTCACCCAC-GCAG-TCCTGAT-AACTAGTTTT

CGGTTGGGGTGACCGCGGAGTAAAACAAAACCTCCTCGATGAAAGGAATTAACTTCCTAA

TCCAAGAGCCACAACTCTAAGAATCAAA--------TTATTGACAAAAATTGATCCAAAT

ACTATTGATCAACGAACCAAGTTACCCTGGGGATAACAGCGCAATCCATTTCAAGAGCTC

ATATCGACAAATGGGTTTACG-ACC-TCGATGTTGGATCAGGGTATCCCAGTG-GCGCAG

CCGCTA-CTAA-AGGTTC

>M. pulchella MN534763, MN534547, MN534654

TTATTGTTAGCTGTTTGTCGACTTACACATGCAAGTCTCAGCATCCCTGTGAGAACGCCC

TTTCACCTCTATCAGGATAAGGAGCTGGTATCAGGCACAG---ACTA-----TCTTGCCC

ACAACACCTAGCTCTGCCACACCCTCAAGGGTACTCAGCAGTGATTAACATTGTTTATGA

GCGCCAGCTCGACTCAGTCAGACAAAGTATAGAGCCGGCTAATCCGGTGCCAGCCGCCGC

GGCTACACCATTAGGCTCAAGTTTACAATTATTCGGCGTTAAGCGTGTTTTAAGTACCC-

TAAACTATTAGAATTAAAATTTAACCAAGTTGTGACACACTTGTTCATAAGAAAAACATA

AACGAAAGTTATTCTAACCAAACCACTTGAATCCACGACAGCTAGGACACAAACTGGGAT

TAGGTACCCCACTATGCCTAGCCGTAAAATATTAACCTACACCT-TCCTTCGCCAGGGAA

TTACGAGCCCAAGCTTAAAACCCAAAGGACTTGACGGTGTCCCACCCATCTAGAGGAGCC

TGTTCTATAA-TCGATTCTCCCC-GATTCACCTCACCACTTCTAGCCTC--TCAGCCTGT

ATACCTCCGTCGTAAGCTTACCATGTGAACGCA-CCATAGTGAGCCCAAAGAT--TTCCC

ATAAATACGTCAGGTCAAGGTGCAGCCCACGATGTGGAAAGTAATGGGCTACAATTTCTA

TCAT--TAGAACAAA-CGAAAGACTACATGCAA-TTCAGTCATGAAGGCGGATTTAGTAG

TAAAAAGAAAATAGAGAGTTCTTTTTAACTAGGCACTGGGACGCGTACACACCGCCCGTC

ACCCTCTTCGAATGTAACTTCCCACGGTTTTTAACAAT-TCCATACCCCACAGAAGAGGT

AAGTCGTAACACGGTAAGCGTACTGGAAAGTGCGCTTGGATTAA-CAAAGTGTAGCTTAA

TTAAA-GCATCTCGCTTACACCGAAAATATATCTGTTAAACCCGGATCACCTTGAA-GCC

TAAAACCTAGCTTTACTTCCTCG---CATAAA-CACT-CTTAACTTTTCTTC---AACAA

TAAAACATTTTTAATTATTTAGTACAGGCGATCAAAAAATACCTT-AAAGCTACAAAAA-

TAGTACCGCAAGGGAAAGGTGAAATAGTAATGAAATAACTCTTAAGCCAAAAAAAGCAGA

GATAAAACCTCGTACCTTTTGCATCATGGTTTAGCTAGTCTAACCAAGCAAAACGCATTT

TTAGTTTGACTCCCCGAAACTAAGCGAGCTACTTTAAAACAGCTTTT-AGAGCAAACCCG

TCTCTGTTGCAAAAGAGTGGGAAGATTTT-AAAGTAGAGGTGATAAACCTACCGAGCTTA

GAGATAGCTGGTTATTCAGGAAAGGGATTTAAGTCCTACCTTAAGTCTCCT--TATAATT

TCTAATACTTCAAT-AGACTTAAGAGCCATTCAAATAAGGTACAGCCTATTTGAACCAGG

GTACAACCTACTCG-----------------------------------CCTAAAAGCAG

CCACCTTTG-AAAAAGCGTTAAAGCTTTATTTC--AATCTCTCATCAA-TTCCTCAA-TT

TTACCGAAACCCCCCACCCC-ATACTGAATGACTTCATA-CTTTATGAAGGACCATATGC

TAAAACTAGTAACAAGAAGA-AGAACTTCTCCA-AAATGCAAGCGTAAATCATAATGAAC

AACTCACTGACAATTAACGTCCATAAGCCCAAAGTAGTAACTCA----CCAAGAAAACTC

TAC------------------TTAAAAC-AACGTTAACCTTACACCAGCGCATTCACAGA

AAGATTAAAAGAATCGGAAGGAACTCGGCAAATTA-CTAACCCCGCCTGTTTACCAAAAA

CATCGCCTCCTGATAAACT---ATAGGAGGTCCAGCCTGCCCAGTGACAAA--GTTAAAC

GGCCGCGGTACCCTAACCGTGCAAAGGTAGCGCAATCACTTGTTCTTTAAATGAGGACTA

GTATGAATGGCATCACGAGGGTTATACTGTCTCCCCACTCTAATCAGTGAAACTGACCTC

CCCGTGAAGAAGCGGGGATATTAATATAAGACGAGAAGACCCCATGGAGCTTTAAACTCA

GTATCACCTGCCACACA-TTACAACCTCTCACCCAC-GCAG-TCCTGAT-AACTAGTTTT

CGGTTGGGGTGACCGCGGAGTAAAACAAAACCTCCTCGATGAAAGGAATTAACTTCCTAA

TCCAAGAGCCACAACTCTAAGAATCAAA--------TTATTGACAAAAATTGATCCAAAT

ACTATTGATCAACGAACCAAGTTACCCTGGGGATAACAGCGCAATCCATTTCAAGAGCTC

ATATCGACAAATGGGTTTACG-ACC-TCGATGTTGGATCAGGGTATCCCAGTG-GCGCAG

CCGCTA-CTAA-AGGTTC

>M. pulchella MN534764, MN534548, MN534655

TTATTGTTAGCTGTTTGTCGACTTACACATGCAAGTCTCAGCATCCCTGTGAGAACGCCC

TTTCACCTCTATCAGGATAAGGAGCTGGTATCAGGCACAG---ACTA-----TCTTGCCC

ACAACACCTAGCTCTGCCACACCCTCAAGGGTACTCAGCAGTGATTAACATTGTTTATGA

GCGCCAGCTCGACTCAGTCAGACAAAGTA-AGAGCCGGCTAATCCGGTGCCAGCCGCCGC

GGCTACACCATTAGGCTCAAGTTTACAATTATTCGGCGTTAAGCGTGTTTTAAGTACCC-

TAAACTATTAGAATTAAAATTTAACCAAGTTGTGACACACTTGTTCATAAGAAAAACATA

AACGAAAGTTATTCTAACCAAACCACTTGAATCCACGACAGCTAGGACACAAACTGGGAT

TAGGTACCCCACTATGCCTAGCCGTAAAATATTAACCTACACCT-TCCTTCGCCAGGGAA

TTACGAGCCCAAGCTTAAAACCCAAAGGACTTGACGGTGTCCCACCCATCTAGAGGAGCC

TGTTCTATAA-TCGATTCTCCCC-GATTCACCTCACCACTTCTAGCCTC--TCAGCCTGT

ATACCTCCGTCGTAAGCTTACCATGTGAACGCA-CCATAGTGAGCCCAAAGAT--TTCCC

ATAAATACGTCAGGTCAAGGTGCAGCCCACGATGTGGAAAGTAATGGGCTACAATTTCTA

TCAT--TAGAACAAA-CGAAAGACTACATGCAA-TTCAGTCATGAAGGCGGATTTAGTAG

TAAAAAGAAAATAGAGAGTTCTTTTTAACTAGGCACTGGGACGCGTACACACCGCCCGTC

ACCCTCTTCAAAGGTAACTTCCCACGGTTTTTAACAAT-TTCATACCCCACAGAAGAGGT

AAGTCGTAACACGGTAAGCGTACTGGAAAGTGCGCTTGGATTAA-CAAAGTGTAGCTTAA

TTAAA-GCATCTCGCTTACACCGAAAATATATCTGTTAAACCCGGATCACCTTGAA-GCC

TAAAACCTAGCTTTACTTCCTCG---CATAAA-CACT-CTTAACTTTTCTTC---AACAA

TAAAACATTTTTAATTATTTAGTACAGGCGATCAAAAAATACCTT-AAAGCTACAAAAA-

TAGTACCGCAAGGGAAAGGTGAAATAGTAATGAAATAACTCTTAAGCCAAAAAAAGCAGA

GATAAAACCTCGTACCTTTTGCATCATGGTTTAGCAAGTCTAACCAAGCAAAACGCATTT

TTAGTTTGACCCCCCGAAACTAAGCGAGCTACTTTAAAACAGCTTTT-AGAGCAAACCCG

TCTCTGTTGCAAAAGAGTGGGAAGATTTT-AAAGTAGAGGTGATAAACCTACCGAGCTTA

GAGATAGCTGGTTATTCAGGAAAGGGATTTAAGTCCTACCTTAAGTCTCCT--TATAATT

TCTAATACTTCAAT-AGACTTAAGAGCCATTCAAATAAGGTACAGCCTATTTGAACCAGG

GTACAAACTACTGCGAC--------------------------------CCTAAAAGCAG

CCACCTTTG-AAAAAGCGTTAAAGCTTTATTTC--AATCTCTCATCAA-TTCCTCAA-TT

TTACCGAAACCCCCCACCCC-ATACTGAATGACTTCATA-CTTTATGAAGGACCATATGC

TAAAACTAGTAACAAGAAGA-AGAACTTCTCCA-AAATGCAAGCGTAAATCATAATGAAC

AACTCACTGACAATTAACGTCCATAAGCCCAAAGTAGTAACTCA----CCAAGAAAACTC

TAC------------------TTAAAAC-AACGTTAACCTTACACCAGCGCATTCACAGA

AAGATTAAAAGAATCGGAAGGAACTCGGCAAATTA-CTAACCCCGCCTGTTTACCAAAAA

CATCGCCTCCTGATAAACT---ATAGGAGGTCCAGCCTGCCCAGTGACAAA--GTTAAAC

GGCCGCGGTACCCTAACCGTGCAAAGGTAGCGCAATCACTTGTTCTTTAAATGAGGACTA

GTATGAATGGCATCACGAGGGTTATACTGTCTCCCCACTCTAATCAGTGAAACTGACCTC

CCCGTGAAGAAGCGGGGATATTAATATAAGACGAGAAGACCCCATGGAGCTTTAAACTCA

GTATCACCTGCCACACA-TTACAACCTCTCACCCAC-GCAG-TCCTGAT-AACTAGTTTT

CGGTTGGGGTGACCGCGGAGTAAAACAAAACCTCCTCGATGAAAGGAATTAACTTCCTAA

TCCAAGAGCCACAACTCTAAGAATCAAA--------TTATTGACAAAAATTGATCCAAAT

ACTATTGATCAACGAACCAAGTTACCCTGGGGATAACAGCGCAATCCATTTCAAGAGCTC

ATATCGACAAATGGGTTTACG-AAC-TTGATGTTGGATCAGGGTATCCCAGTG-GCGCAG

CCGCTA-CTAA-AGGTTC

>M. pulchella MN534765, MN534549, MN534656

TTATTGTTAGCTGTTTGTCGACTTACACATGCAAGTCTCAGCATCCCTGTGAGAACGCCC

TTTCACCTCTATCAGGATAAGGAGCTGGTATCAGGCACAG---ACTA-----TCTTGCCC

ACAACACCTAGCTCTGCCACACCCTCAAGGGTACTCAGCAGTGATTAACATTGTTTATGA

GCGCCAGCTCGACTCAGTCAGACAAAGTA-AGAGCCGGCTAATCCGGTGCCAGCCGCCGC

GGCTACACCATTAGGCTCAAGTTTACAATTATTCGGCGTTAAGCGTGTTTTAAGTACCC-

CAAACTATTAGAATTAAAATTTAACCAAGTTGTGACACACTTGTTCATAAGAAAAACATA

AACGAAAGTTATTCTAACCAAACCACTTGAATCCACGACAGCTAGGACACAAACTGGGAT

TAGGTACCCCACTATGCCTAGCCGTAAAATATTAACCTACACCT-TCCTTCGCCAGGGAA

TTACGAGCCCAAGCTTAAAACCCAAAGGACTTGACGGTGTCCCACCCATCTAGAGGAGCC

TGTTCTATAA-TCGATTCTCCCC-GATTCACCTCACCACTTCTAGCCTC--TCAGCCTGT

ATACCTCCGTCGTAAGCTTACCATGTGAACGCA-CCATAGTGAGCCCAAAGAT--TTCCC

ATAAATACGTCAGGTCAAGGTGCAGCCCACGATGTGGAAAGTAATGGGCTACAATTTCTA

TCAT--TAGAACAAA-CGAAAGACTACATGCAA-TTCAGTCATGAAGGCGGATTTAGTAG

TAAAAAGAAAATAGAGAGTTCTTTTTAACTAGGCACTGGGACGCGTACACACCGCCCGTC

ACCCTCTTCAAATGTAACTTCCCACGGTTTTTAACAAT-TCCATACCCCACAGAAGAGGT

AAGTCGTAACACGGTAAGCGTACTGGAAAGTGCGCTTGGATTAA-CAAAGTGTAGCTTAA

TTAAA-GCATCTCGCTTACACCGAAAATATATCTGTTAAACCCGGATCACCTTGAA-GCC

TAAAACCTAGCTTTACTTCCTCG---CATAAA-CACT-CTTGACTTTTCTTC---AACAA

TAAAACATTTTTAATTATTTAGTACAGGCGATCAAAAAATACCTT-AAAGCTACAAAAA-

TAGTACCGCAAGGGAAAGGTGAAATAGTAATGAAATAACTCTTAAGCCAAAAAAAGCAGA

GATAAAACCTCGTACCTTTTGCATCATGGTTTAGCAAGTCTAACCAAGCAAAACGCATTT

TTAGTTTGACCCCCCGAAACTAAGCGAGCTACTTTAAAACAGCTTTT-AGAGCAAACCCG

TCTCTGTTGCAAAAGAGTGGGAAGATTTT-AAAGTAGAGGTGATAAACCTACCGAGCTTA

GAGATAGCTGGTTATTCAGGAAAGGGATTTAAGTCCTACCTTAAGTCTCCT--TATAATT

TCTAATACTTCAAT-AGACTTAAGAGCCATTCAAATAAGGTACAGCCTATTTGAACCAGG

GTACAACCTACTTCGCT-GGTAAC-------------------------CCTAAAAGCAG

CCACCTTTG-AAAAAGCGTTAAAGCTTTATTTC--AATCTCTCATCAA-TTCCTCAA-TT

TTACCGAAACCCCCCACCCCCATACTGAATGACTTCATA-CTTTATGAAGGACCATATGC

TAAAACTAGTAACAAGAAGA-AGAACTTCTCCA-AAATGCAAGCGTAAATCATAATGAAC

AACTCACTGACAATTAACGTCCATAAGCCCAAAGTAGTAACTCA----CCAAGAAAACTC

TAC------------------TTAAAAC-AACGTTAACCTTACACCAGCGCATTCACAGA

AAGATTAAAAGAATCGGAAGGAACTCGGCAAATTA-CTAACCCCGCCTGTTTACCAAAAA

CATCGCCTCCTGATAAACT---ATAGGAGGTCCAGCCTGCCCAGTGACAAA--GTTAAAC

GGCCGCGGTACCCTAACCGTGCAAAGGTAGCGCAATCACTTGTTCTTTAAATGAGGACTA

GTATGAATGGCATCACGAGGGTTATACTGTCTCCCCACTCTAATCAGTGAAACTGACCTC

CCCGTGAAGAAGCGGGGATATTAATATAAGACGAGAAGACCCCATGGAGCTTTAAACTCA

GTATCACCTGCCACACA-TTACAACCTCTCACCCAC-GCAG-TCCTGAT-AACTAGTTTT

CGGTTGGGGTGACCGCGGAGTAAAACAAAACCTCCTCGATGAAAGGAATTAACTTCCTAA

TCCAAGAGCCACAACTCTAAGAATCAAA--------TTATTGACAAAAATTGATCCAAAT

ACTATTGATCAACGAACCAAGTTACCCTGGGGATAACAGCGCAATCCATTTCAAGAGCTC

ATATCGACAAATGGGTTTACG-ACC-TCGATGTTGGATCAGGGTATCCCAGTG-GCGCAG

CCGCTA-CTAA-AGGTTC
