# Supplementary material for: A little frog leaps a long way: compounded colonizations of the Indian Subcontinent discovered in the tiny Oriental frog genus Microhyla (Amphibia: Microhylidae)
Source: PeerJ. 2020 Jul 3;8:e9411. doi: 10.7717/peerj.9411 (PMC7337035; doi:10.7717/peerj.9411)
Supplement: Supplemental Information 19 — See Supplemental Table S1 for sequence details. [file peerj-08-9411-s019.pdf]

Supplemental File 4. Raw data file - aligned newly generated nuDNA sequences (*BDNF* gene) (see Supplemental Table S1 for sequence details).

>M. nepenthicola MN534393

--------------------AGTTGCATGAAAGCTGCCCCAATGAAAGAAGCCAATGTCA

GAGGACAAAGTGGCCTGGCTTTTCCGGGTCTTCGGACCCACGGTACTCTGGAGAACATAG

GTGGGCCGATAGGTGGTGGTTCAAGAGGAGGTGGACTTCCATCGCTGACAGATACCTTTG

AGCAGGTTATAGAGGAACTCCTGGAAGAAGAACAGACCATGAGGCAGAGTGAAGAGAACA

AGGAC---GACATGTATTCATCTAGAGTAATGCTGAGCACTCAAGTGCCTTTGGAGCCAC

CTTTGCTTTTTCTCCTTGAGGAGTACAAAAACTACTTGGATGCTGCAAACATGTCCATGA

GAGTGAGGCGCCACTCTGACCCCGCTCGGCGCGGGGAACTCAGCGTCTGTGACAGTATTA

GCGAATGGGTGACTGCGGCCGACAAGAAAACTGCAGTGGACATGGCGGGGCAAACGGTTA

CCGTTCTGGAAAAAGTCCCAGTACCCAAAGGTCAACTGAAACAATATTTCTATGAGACCA

AATGCAATCCTATGGGCTACATGAAGGAAGGTTGCAGGGGCATTGACAAAAGGTACTGGA

ACTCGCAATGCCGAACTACCCAGTCTTATGTGCGTGCGCTCACCATGGATAGCAAAAAAA

AAGTTGGCTGGCGCTTTATAAGGATAGACACTTCTTGTGTATGTACACTGACCATTAAA-

>M. borneensis MN534394

--------------------AGTTGCATGAAAGCTGCCCCAATGAAAGAAGCCAATGTCA

GAGGACAAAGTGGCCTGGCTTTTCCGGGTCTTCGGACCCACGGTACTCTGGAGAACATAG

GTGGGCCGATAGGTGGTGGTTCAAGAGGAGGTGGACTTCCATCGCTGACAGATACCTTTG

AGCAGGTTATAGAGGAACTCCTGGAAGAGGAACAGACCATGAGGCAGAGTGAAGAGAACA

AGGAC---GACATGTATTCATCTAGAGTAATGCTGAGCACTCAAGTGCCTTTGGAGCCAC

CTTTGCTTTTTCTCCTTGAGGAGTACAAAAACTACTTGGATGCTGCAAACATGTCCATGA

GAGTGAGGCGCCACTCTGACCCCGCTCGGCGCGGGGAACTCAGCGTCTGTGACAGTATTA

GCGAATGGGTGACTGCGGCCGACAAGAAAACTGCAGTGGACATGGCGGGGCAAACGGTTA

CCGTTCTGGAAAAAGTCCCAGTACCCAAAGGTCAACTGAAACAATATTTCTATGAGACCA

AATGCAATCCTATGGGCTACATGAAGGAAGGTTGCAGGGGCATTGACAAAAGGTACTGGA

ACTCGCAATGCCGAACTACCCAGTCTTATGTGCGTGCGCTCACCATGGATAGCAAAAAAA

AAGTTGGCTGGCGCTTTATAAGGATAGACACTTCTTGTGTATGTACACTGACCATTAAA-

>Microhyla sp. 1 MN534395

--------------------AGTTGCATGAAAGCTGCCCCAATGAAAGAAGCCAATGTCA

GAGGACAAAGTGGCCTGGCTTTTCCGGGTCTTCGGACCCACGGTACTCTGGAGAACATAG

GTGGGCCGATAGGTGGTGGTTCAAGAGGAGGTGGACTTCCATCGCTGACAGATACCTTTG

AGCAGGTTATAGAGGAACTCCTGGAAGAGGAACAGACCATGAGGCAGAGTGAAGAGAACA

AGGAC---GACATGTATTCATCTAGAGTAATGCTGAGCACTCAAGTGCCTTTGGAGCCAC

CTTTGCTTTTTCTCCTTGAGGAGTACAAAAACTACTTGGATGCTGCAAACATGTCCATGA

GAGTGAGGCGCCACTCTGACCCCGCTCGGCGCGGGGAACTCAGCGTCTGTGACAGTATTA

GCGAATGGGTGACTGCGGCCGACAAGAAAACTGCAGTGGACATGGCGGGGCAAACGGTTA

CCGTTCTGGAAAAAGTCCCAGTACCCAAAGGTCAACTGAAACAATATTTCTATGAGACCA

AATGCAATCCTATGGGCTACATGAAGGAAGGTTGCAGGGGCATTGACAAAAGGTACTGGA

ACTCGCAATGCCGAACTACCCAGTCTTATGTGCGTGCGCTCACCATGGATAGCAAAAAAA

AAGTTGGCTGGCGCTTTATAAGGATAGACACTTCTTGTGTATGTACACTGACCATTAAA-

>M. malang MN534396

--------------------AGTTGCATGAAAGCTGCCCCAATGAAAGAAGCCAATGTCA

GAGGACAAAGTGGCCTGGCTTTTCCGGGTCTTCGGACCCACGGTACTCTGGAGAACATAG

GTGGGCCGATAGGTGGTGGTTCAAGAGGAGGTGGACTTCCATCGCTGACCGATACCTTTG

AGCAGGTTATAGAGGAACTCCTGGAAGAGGAACAGACCATGAGGCAGAGTGAAGAGAACA

AGGAC---GACATGTATTCATCTAGAGTAATGCTGAGCACTCAAGTGCCTTTGGAGCCAC

CTTTGCTTTTTCTCCTTGAGGAGTACAAAAACTACTTGGATGCTGCAAACATGTCCATGA

GAGTGAGGCGCCACTCTGACCCCGCTCGGCGCGGGGAACTCAGCGTCTGTGACAGTATTA

GCGAATGGGTGACTGCGGCCGACAAGAAAACTGCAGTGGACATGGCGGGGCAAACGGTTA

CCGTTCTGGAAAAAGTCCCAGTACCCAAAGGTCAACTGAAACAATATTTCTATGAGACCA

AATGCAATCCTATGGGCTACATGAAGGAAGGTTGCAGGGGCATTGACAAAAGGTACTGGA

ACTCGCAATGCCGAACTACCCAGTCTTATGTGCGTGCGCTCACCATGGATAGCAAAAAAA

AAGTTGGCTGGCGCTTTATAAGGATAGACACTTCTTGTGTATGTACACTGACCATTAAA-

>M. orientalis MN534397

--ATGGTTATTTCATACTTAAGTTGCATGAAAGCTGCCCCAATGAAAGAAGCCAATGTCA

GAGGACAAAGTGGCCTGGCTTATCCGGGTCTTCGGACCCACGGTACTCTGGAGAACATAG

GTGGGCCGATAGGTGG---TTCAAGAGGAGGTGGACTTCCATCGCTGACAGATACCTTTG

AGCAGGTTATAGAGGAACTCCTGGAAGAGGAACAGACCATGAGGCAGAGTGAAGAGAACA

AGGAC---GACATGTATTCATCTAGAGTAATGCTGAGCACTCAAGTGCCTTTGGAGCCAC

CTTTGCTTTTTCTCCTTGAGGAGTACAAAAACTACTTGGATGCCGCAAACATGTCCATGA

GGGTGAGGCGCCACTCTGACCCTGCTCGGCGCGGGGAACTGAGCGTCTGTGACAGTATTA

GCGAATGGGTGACTGCGGCCGACAAGAAAACTGCAGTGGACATGGCGGGGCAAACGGTTA

CCGTTCTGGAAAAAGTCCCAGTACCCAAAGGTCAACTGAAACAATATTTCTATGAGACCA

AATGCAATCCTATGGGCTACATGAAGGAAGGTTGCAGGGGCATTGACAAAAGGTACTGGA

ACTCGCAATGCCGAACTACCCAGTCTTATGTGCGTGCGCTCACCATGGATAGCAAAAAAA

AAGTTGGCTGGCGCTTTATAAGGATAGACACTTCTTGTGTATGTACACTGACCATTAAA-

>M. mantheyi KM509398

--------------------AGTTGCATGAAAGCTGCCCCAATGAAAGAAGCCAATGTCA

GAGGACAAAGTGGCCTGGCTTATCCGGGTCTTCGAACCCACGGTACTCTGGAGAACATAG

GTGGGCCAATAGGTGG---TTCAAGAGGAGGTGGACTTCCATCGCTGACAGACACCTTTG

AGCAGGTTATAGAGGAACTCCTGGAAGAGGAACAGACCATGAGGCAGAGTGAAGAGAACA

AGGAC---GACATGTATTCATCTAGAGTAATGCTGAGCACTCAAGTGCCTTTGGAGCCAC

CTTTGCTTTTTCTTCTTGAGGAGTACAAAAACTACTTGGATGCTGCAAACATGTCCATGA

GGGTGAGGCGCCACTCTGACCCCGCTAGGCGCGGGGAACTGAGCGTCTGTGACAGTATTA

GTGAATGGGTGACTGCGGCCGACAAGAAAACTGCAGTGGACATGGCGGGGCAAACGGTTA

CCGTTCTGGAAAAAGTCCCAGTACCCAAAGGTCAACTGAAACAATATTTCTATGAGACCA

AATGCAATCCTATGGGCTACATGAAGGAAGGTTGCAGGGGCATTGACAAAAGGTACTGGA

ACTCGCAATGCCGAACTACCCAGTCTTATGTGCGTGCGCTCACCATGGATAGCAAAAAAA

AAGTTGGCTGGCGCTTTATAAGGATAGACACTTCTTGTGTATGTACACTGACCATTAAA-

>M. pineticola MN534399

CTATGGTTATTTCATACTTAAGTTGCATGAAAGCTGCCCCAATGAAAGAAGCCAATGTCA

GAGGACAAAGTGGCCTGGCTTATCCGGGTCTTCGGACCCACGGTACTCTGGAGAACATAG

GTGGGCCGATAGGTGG---TTCAAGAGGAGGTGGACTTCCATCGCTGACAGATACCTTTG

AGCAGGTTATAGAGGAACTCCTAGAAGAGGAACAGACCATGAGGCAGAGTGAAGAGAACA

AGGAC---GACATGTATACATCTAGAGTAATGCTGAGCACTCAAGTGCCTTTGGAGCCAC

CTTTGCTTTTTCTCCTTGAGGAGTACAAAAACTACTTGGATGCTGCAAACATGTCTATGA

GGGTGAGGCGCCACTCTGACCCCGCTCGGCGTGGGGAACTGAGCGTCTGTGACAGTATTA

GTGAATGGGTGACTGCGGCCGACAAGAAAACTGCAGTGGACATGGCGGGGCAAACGGTTA

CCGTTCTGGAAAAAGTCCCAGTACCCAAAGGTCAACTGAAACAATATTTCTATGAGACCA

AATGCAATCCTATGGGCTACATGAAGGATGGTTGCAGGGGCATTGACAAAAGGTACTGGA

ACTCGCAATGCCGAACTACCCAGTCTTATGTGCGTGCGCTCACCATGGATAGCAAAAAAA

AAGTTGGCTGGCGCTTTATAAGGATAGACACTTCTTGTGTATGTACACTGACCATTAAA-

>M. minuta MN534400

--------------------AGTTGCATGAAAGCTGCCCCAATGAAAGAAGCCAATGTCA

GAGGACAAAGTGGCCTGGCTTATCCGGGTCTTCGGACCCACGGTACTCTGGAGAACATAG

GTGGGCCGATAGGTGG---TTCAAGAGGAGGTGGACTTCCATCGCTGACAGATACCTTTG

AGCAGGTTATAGAGGAGCTCCTGGAAGAGGAACAGACCATGAGGCAGAGTGAAGAGAACA

AGGAC---GACATGTATTCATCTAGAGTAATGCTGAGCACTCAAGTGCCTTTGGAGCCAC

CTTTGCTTTTTCTCCTTGAGGAGTACAAAAACTACTTGGATGCTGCAAACATGTCCATGA

GGGTGAGGCGCCACTCTGACCCCGCTCGGCGTGGGGAACTGAGCGTCTGTGACAGTATTA

GCGAATGGGTGACTGCGGCCGACAAGAAAACTGCAGTGGACATGGCGGGGCAAACGGTTA

CCGTTCTGGAAAAAGTCCCAGTACCCAAAGGTCAACTGAAACAATATTTCTATGAGACCA

AATGCAATCCTATGGGCTACATGAAGGAAGGTTGCAGGGGCATTGACAAAAGGTACTGGA

ACTCGCAATGCCGAACTACCCAGTCTTATGTGCGTGCGCTCACCATGGATAGCAAAAAAA

AAGTGGGCTGGCGCTTTATAAGGATAGACACTTCTTGTGTATGTACACTGACCATTAAA-

>M. fodiens MN534401

-------TATTTCATACTTAAGTTGCATGAAAGCTGCCCCAATGAAAGAAGCCAATGTCA

GAGGACAAAGTGGCCTGGCTTATCCGGGTCTTCGGACCCACGGTACTTTGGAGAACATAG

GTGGGCCGATAGGTGG---TTCAAGAGGAGGTGGACTTCCATCGCTGACAGATACCTTTG

AGCAGGTTATAGAGGAACTCCTAGAAGAGGAACAGACCATGAGGCAGAGTGAAGAGAACA

AGGAC---GACATGTATTCATCTAGAGTAATGCTGAGCACTCAAGTGCCTTTGGAGCCAC

CTTTGCTTTTTCTCCTTGAGGAGTACAAAAACTACCTGGATGCAGCAAACATGTCCATGA

GGGTGAGGCGCCACTCCGACCCCGCTCGGCGCGGGGAACTGAGCGTCTGTGACAGTATTA

GCGAATGGGTGACTGCGGCCGACAAAAAAACCGCAGTGGACATGGCGGGGCAAACGGTTA

CCGTTCTGGAAAAAGTCCCAGTACCCAAAGGTCAACTGAAACAATATTTCTATGAGACCA

AATGCAATCCTATGGGCTACATGAAGGAAGGTTGCAGGGGCATTGACAAAAGGTACTGGA

ACTCGCAATGCCGAACTACCCAGTCTTATGTGCGTGCGCTCACCATGGATAGCAAAAAAA

AAGTTGGCTGGCGCTTTATAAGGATAGACACTTCTTGTGTATGTACACTGACCATTAAA-

>M. achatina MN534402

--------------------AGTTGCATGAAAGCTGCCCCAATGAAAGAAGCCAATGTCA

GAGGACAAAGTGGCCTGGCTTATCCGGGTCTTCGGACCCACGGTACTCTGGAGAACATAG

GTGGGCCGATAGGTGG---TTCAAGAGGAGGTGGACTTCCATCGCTGACAGATACCTTTG

AGCAGGTTATAGAGGAACTCCTGGAAGAGGAACAGACCATGAGGCAGAGTGAAGAGAACA

AGGAC---GACATGTATTCATCTAGAGTAATGCTGAGCACTCAAGTGCCTTTGGAGCCAC

CTTTGCTTTTTCTCCTTGAGGAGTACAAAAACTACTTGGATGCTGCAAACATGTCCATGA

GGGTGAGGCGCCACTCTGACCCCGCTCGGCGCGGGGAACTGAGCGTCTGTGACAGTATTA

GTGAATGGGTGACTGCGGCCGACAAGAAAACTGCAGTGGACATGGCGGGGCAAACGGTTA

CTGTTCTGGAAAAAGTCCCGGTACCCAAAGGTCAACTGAAACAATATTTCTATGAGACCA

AATGCAATCCTATGGGCTACATGAAGGAAGGTTGCAGGGGCATTGATAAAAGGTACTGGA

ACTCGCAATGCCGAACTACCCAGTCTTATGTGCGTGCGCTCACCATGGATAGCAAAAAAA

AAGTGGGCTGGCGCTTTATAAGGATAGACACTTCTTGTGTATGTACACTGACCATTAAA-

>M. irrawaddy MN534403

--ATGGTTATTTCATACTTAAGTTGCATGAAAGCTGCCCCAATGAAAGAAGCCAATGTCA

GAGGACAAAGTGGCCTGGCTTATCCGGGTCTCCGGACCCACGGTACTCTGGAGAACATAG

GTGGGCCGATAGGTGG---TTCAAGAGGAGGTGGACTTCCATCGCTGACAGATACCTTTG

AGCAAGTCATAGAGGAACTCCTAGAAGAGGAACAGACCATGAGGCAGAGTGAAGAGAACA

AGGAC---GACATGTATTCATCTAGAGTCATGCTGAGCACTCAAGTGCCTTTGGAGCCAC

CTTTGCTTTTTCTCCTTGAGGAGTACAAAAACTACTTGGATGCTGCAAACATGTCCATGA

GGGTGAGGCGCCACTCTGACCCCGCTCGGCGCGGGGAACTGAGCGTCTGTGACAGTATTA

GCGAATGGGTGACTGCGGCCGACAAGAAAACTGCAGTGGACATGGCGGGGCAAACGGTTA

CTGTTCTGGAAAAAGTCCCAGTACCCAAAGGTCAACTGAAACAATATTTCTATGAGACCA

AATGCAATCCTATGGGCTACATGAAGGAAGGTTGCAGGGGCATTGACAAAAGGTACTGGA

ACTCGCAATGCCGAACTACCCAGTCTTATGTGCGTGCGCTCACCATGGATAGCAAAAAAA

AAGTTGGCTGGCGCTTTATAAGGATAGACACTTCTTGTGTATGTACACTGACCATTAAA-

>M. heymonsi MN534404

CTATGGTTATTTCATACTTAAGTTGCATGAAAGCTGCCCCAATGAAAGAAGCCAATGTCA

GAGGACAAAGTGGCCTGGCTTATCCGGGTCTTCGGACCCACGGTACTCTGGAGAACATAG

GTGGGCCGATAGGTGG---TTCAAGAGGAGGTGGACTTCCATCGCTGACAGATACCTTTG

AGCAGGTTATAGAGGAACTCCTAGAAGAGGAACAGACCATGAGGCAGAGTGAAGAGAACA

AGGAC---GACATGTATTCATCTAGAGTAATGCTGAGCACTCAAGTGCCTTTGGAGCCAC

CTTTGCTTTTTCTCCTTGAGGAGTACAAAAACTACTTGGATGCTGCAAACATGTCCATGA

GGGTGAGGCGCCACTCCGACCCCGCTCGGCGCGGGGAACTGAGCGTCTGTGACAGTATTA

GTGAATGGGTGACTGCGGCCGACAAGAAAACTGCAGTGGACATGGCGGGGCAAACGGTTA

CTGTTCTGGAAAAAGTCCCAGTACCCAAAGGTCAACTGAAACAATATTTCTATGAGACCA

AATGCAATCCTATGGGCTACATGAAGGAAGGTTGCAGGGGCATTGACAAAAGGTACTGGA

ACTCGCAATGCCGAACTACCCAGTCTTATGTGCGTGCGCTCACCATGGATAGCAAAAAAA

AAGTTGGCTGGCGCTTTATAAGGATAGACACTTCTTGTGTATGTACACTGACCATTAAA-

>M. heymonsi MN534405

CTATGGTTATTTCATACTTAAGTTGCATGAAAGCTGCCCCAATGAAAGAAGCCAATGTCA

GAGGACAAAGTGGCCTGGCTTATCCGGGTCTTCGGACCCACGGTACTCTGGAGAACATAG

GTGGGCCGATAGGTGG---TTCAAGAGGAGGTGGACTTCCATCGCTGACAGATACCTTTG

AGCAGGTTATAGAGGAACTCCTAGAAGAGGAACAGACCATGAGGCAGAGTGAAGAGAACA

AGGAC---GACATGTATTCATCTAGAGTAATGCTGAGCACTCAAGTGCCTTTGGAGCCAC

CTTTGCTTTTTCTCCTTGAGGAGTACAAAAACTACTTGGATGCTGCAAACATGTCCATGA

GGGTGAGGCGCCACTCCGACCCCGCTCGGCGCGGGGAACTGAGCGTCTGTGACAGTATTA

GTGAATGGGTGACTGCGGCCGACAAGAAAACTGCAGTGGACATGGCGGGGCAAACGGTTA

CTGTTCTGGAAAAAGTCCCAGTACCCAAAGGTCAACTGAAACAATATTTCTATGAGACCA

AATGCAATCCTATGGGCTACATGAAGGAAGGTTGCAGGGGCATTGACAAAAGGTACTGGA

ACTCGCAATGCCGAACTACCCAGTCTTATGTGCGTGCGCTCACCATGGATAGCAAAAAAA

AAGTTGGCTGGCGCTTTATAAGGATAGACACTTCTTGTGTATGTACACTGACCATTAAA-

>M. heymonsi MN534406

CTATGGTTATTTCATACTTAAGTTGCATGAAAGCTGCCCCAATGAAAGAAGCCAATGTCA

GAGGACAAAGTGGCCTGGCTTATCCGGGTCTTCGGACCCACGGTACTCTGGAGAACATAG

GTGGGCCGATAGGTGG---TTCAAGAGGAGGTGGACTTCCATCGCTGACAGATACCTTTG

AGCAGGTTATAGAGGAACTCCTAGAAGAGGAACAGACCATGAGGCAGAGTGAAGAGAACA

AGGAC---GACATGTATTCATCTAGAGTAATGCTGAGCACTCAAGTGCCTTTGGAGCCAC

CTTTGCTTTTTCTCCTTGAGGAGTACAAAAACTACTTGGATGCTGCAAACATGTCCATGA

GGGTGAGGCGCCACTCCGACCCCGCTCGGCGCGGGGAACTGAGCGTCTGTGACAGTATTA

GTGAATGGGTGACTGCGGCCGACAAGAAAACTGCAGTGGACATGGCGGGGCAAACGGTTA

CTGTTCTGGAAAAAGTCCCAGTACCCAAAGGTCAACTGAAACAATATTTCTATGAGACCA

AATGCAATCCTATGGGCTACATGAAGGAAGGTTGCAGGGGCATTGACAAAAGGTACTGGA

ACTCGCAATGCCGAACTACCCAGTCTTATGTGCGTGCGCTCACCATGGATAGCAAAAAAA

AAGTTGGCTGGCGCTTTATAAGGATAGACACTTCTTGTGTATGTACACTGACCATTAAA-

>M. heymonsi MN534407

CTATGGTTATTTCATACTTAAGTTGCATGAAAGCTGCCCCAATGAAAGAAGCCAATGTCA

GAGGACAAAGTGGCCTGGCTTATCCGGGTCTTCGGACCCACGGTACTCTGGAGAACATAG

GTGGGCCGATAGGTGG---TTCAAGAGGAGGTGGACTTCCATCGCTGACAGATACCTTTG

AGCAGGTTATAGAGGAACTCCTAGAAGAGGAACAGACCATGAGGCAGAGTGAAGAGAACA

AGGAC---GACATGTATTCATCTAGAGTAATGCTGAGCACTCAAGTGCCTTTGGAGCCAC

CTTTGCTTTTTCTCCTTGAGGAGTACAAAAACTACTTGGATGCTGCAAACATGTCCATGA

GGGTGAGGCGCCACTCCGACCCCGCTCGGCGCGGGGAACTGAGCGTCTGTGACAGTATTA

GTGAATGGGTGACTGCGGCCGACAAGAAAACTGCAGTGGACATGGCGGGGCAAACGGTTA

CTGTTCTGGAAAAAGTTCCAGTACCCAAAGGTCAACTGAAACAATATTTCTATGAGACCA

AATGCAATCCTATGGGCTACATGAAGGAAGGTTGCAGGGGCATTGACAAAAGGTACTGGA

ACTCGCAATGCCGAACTACCCAGTCTTATGTGCGTGCGCTCACCATGGATAGCAAAAAAA

AAGTTGGCTGGCGCTTTATAAGGATAGACACTTCTTGTGTATGTACACTGACCATTAAA-

>M. heymonsi MN534408

CTATGGTTATTTCATACTTAAGTTGCATGAAAGCTGCCCCAATGAAAGAAGCCAATGTCA

GAGGACAAAGTGGCCTGGCTTATCCGGGTCTTCGGACCCACGGTACTCTGGAGAACATAG

GTGGGCCGATAGGTGG---TTCAAGAGGAGGTGGACTTCCATCGCTGACAGATACCTTTG

AGCAGGTTATAGAGGAACTCCTAGAAGAGGAACAGACCATGAGGCAGAGTGAAGAGAACA

AGGAC---GACATGTATTCATCTAGAGTAATGCTGAGCACTCAAGTGCCTTTGGAGCCAC

CTTTGCTTTTTCTCCTTGAGGAGTACAAAAACTACTTGGATGCTGCAAACATGTCCATGA

GGGTGAGGCGCCACTCTGACCCCGCTCGGCGCGGGGAACTGAGCGTCTGTGACAGTATTA

GTGAATGGGTGACTGCGGCCGACAAGAAAACTGCAGTGGACATGGCGGGGCAAACGGTTA

CTGTTCTGGAAAAAGTCCCTGTACCCAAAGGTCAACTGAAACAATATTTCTATGAGACCA

AATGCAATCCTATGGGCTACATGAAGGAAGGTTGCAGGGGCATTGACAAAAGGTACTGGA

ACTCGCAATGCCGAACTACCCAGTCTTATGTGCGTGCGCTCACCATGGATAGCAAAAAAA

AAGTTGGCTGGCGCTTTATAAGGATAGACACTTCTTGTGTATGTACACTGACCATTAAA-

>M. heymonsi MN534409

CTATGGTTATTTCATACTTAAGTTGCATGAAAGCTGCCCCAATGAAAGAAGCCAATGTCA

GAGGACAAAGTGGCCTGGCCTATCCGGGTCTTCGGACCCACGGTACTCTGGAGAACATAG

GTGGGCCGATAGGTGG---TTCAAGAGGAGGTGGACTTCCATCGCTGACAGATACCTTTG

AGCAGGTTATAGAGGAACTCCTAGAAGAGGAACAGACCATGAGGCAAAGTGAAGAGAACA

AGGAC---GACATGTATTCATCTAGAGTAATGCTGAGCACTCAAGTGCCTTTGGAGCCAC

CTTTGCTTTTTCTCCTTGAGGAGTACAAAAACTACTTGGATGCTGCAAACATGTCCATGA

GGGTGAGGCGCCACTCCGACCCCGCTCGGCGCGGGGAACTGAGCGTCTGTGACAGTATTA

GTGAATGGGTGACTGCGGCCGACAAGAAAACTGCAGTGGACATGGCGGGGCAAACGGTTA

CTGTTCTGGAAAAAGTCCCAGTACCCAAAGGTCAACTGAAACAATATTTCTATGAGACCA

AATGCAATCCTATGGGCTACATGAAGGAAGGTTGCAGGGGCATTGACAAAAGGTACTGGA

ACTCGCAATGCCGAACTACCCAGTCTTATGTGCGTGCGCTCACCATGGATAGCAAAAAAA

AAGTTGGCTGGCGCTTTATAAGGATAGACACTTCTTGTGTATGTACACTGACCATTAAA-

>M. heymonsi MN534410

CTATGGTTATTTCATACTTAAGTTGCATGAAAGCTGCCCCAATGAAAGAAGCCAATGTCA

GAGGACAAAGTGGCCTGGCCTATCCGGGTCTTCGGACCCACGGTACTCTGGAGAACATAG

GTGGGCCGATAGGTGG---TTCAAGAGGAGGTGGACTTCCATCGCTGACAGATACCTTTG

AGCAGGTTATAGAGGAACTCCTAGAAGAGGAACAGACCATGAGGCAGAGTGAAGAGAACA

AGGAC---GACATGTATTCATCTAGAGTAATGCTGAGCACTCAAGTGCCTTTGGAGCCAC

CTTTGCTTTTTCTCCTTGAGGAGTACAAAAACTACTTGGATGCTGCAAACATGTCCATGA

GGGTGAGGCGCCACTCCGACCCCGCTCGGCGCGGGGAACTGAGCGTCTGTGACAGTATTA

GTGAATGGGTGACTGCGGCCGACAAGAAAACTGCAGTGGACATGGCGGGGCAAACGGTTA

CTGTTCTGGAAAAAGTCCCAGTACCCAAAGGTCAACTGAAACAATATTTCTATGAGACCA

AATGCAATCCTATGGGCTACATGAAGGAAGGTTGCAGGGGCATTGACAAAAGGTACTGGA

ACTCGCAATGCCGAACTACCCAGTCTTATGTGCGTGCGCTCACCATGGATAGCAAAAAAA

AAGTTGGCTGGCGCTTTATAAGGATAGACACTTCTTGTGTATGTACACTGACCATTAAAA

>M. mukhlesuri MN534411

--ATGGTTATTTCATACTTAAGTTGCATGAAAGCTGCCCCAATGAAAGAAGCCAATGTCA

GAGGACAAAGTGGCCTGGCTTATCCGGGTCTTCGGACCCACGGTACTCTGGAGAACATAG

GTGGGCCGATAGGTGG---TTCACGAGGAGGTGGACTTCCATCGCTGACAGACACCTTTG

AGCAGGTAATAGAGGAACTCCTAGAAGAGGAACAGACCATGAGGCAGAGTGAAGAGAACA

AGGAC---GACATGTATACATCTAGAGTCATGCTGAGCACTCAAGTGCCTTTGGAGCCAC

CTTTGCTTTTTCTTCTTGAGGAGTACAAAAACTACTTGGATGCTGCAAACATGTCCATGA

GGGTGAGGCGCCACTCTGACCCGGCTCGGCGCGGGGAACTGAGCGTCTGTGACAGTATTA

GCGAATGGGTGACTGCGGCCGACAAGAAAACTGCAGTGGACATGGCGGGGCAAACGGTTA

CCGTTCTGGAAAAAGTCCCAGTACCCAAAGGTCAACTGAAACAATATTTCTATGAGACCA

AATGCAATCCTATGGGCTACATGAAGGATGGTTGCAGGGGCATTGACAAAAGGTACTGGA

ACTCGCAATGCCGAACTACCCAGTCTTATGTGCGTGCGCTCACCATGGATAGCAAAAAAA

AAGTGGGCTGGCGCTTTATAAGGATAGACACTTCTTGTGTATGTACACTGACCATTAAA-

>M. mukhlesuri MN534412

--ATGGTTATTTCATACTTAAGTTGCATGAAAGCTGCCCCAATGAAAGAAGCCAATGTCA

GAGGACAAAGTGGCCTGGCTTATCCGGGTCTTCGGACCCACGGTACTCTGGAGAACATAG

GTGGGCCGATAGGTGG---TTCACGAGGAGGTGGACTTCCATCGCTGACAGACACCTTTG

AGCAGGTAATAGAGGAACTCCTAGAAGAGGAACAGACCATGAGGCAGAGTGAAGAGAACA

AGGAC---GACATGTATACATCTAGAGTCATGCTGAGCACTCAAGTGCCTTTGGAGCCAC

CTTTGCTTTTTCTTCTTGAGGAGTACAAAAACTACTTGGATGCTGCAAACATGTCCATGA

GGGTGAGGCGCCACTCTGACCCGGCTCGGCGCGGGGAACTGAGCGTCTGTGACAGTATTA

GCGAATGGGTGACTGCGGCCGACAAGAAAACTGCAGTGGACATGGCGGGGCAAACGGTTA

CCGTTCTGGAAAAAGTCCCAGTACCCAAAGGTCAACTGAAACAATATTTCTATGAGACCA

AATGCAATCCTATGGGCTACATGAAGGATGGTTGCAGGGGCATTGACAAAAGGTACTGGA

ACTCGCAATGCCGAACTACCCAGTCTTATGTGCGTGCGCTCACCATGGATAGCAAAAAAA

AAGTGGGCTGGCGCTTTATAAGGATAGACACTTCTTGTGTATGTACACTGACCATTAAA-

>M. mukhlesuri MN534413

--ATGGTTATTTCATACTTAAGTTGCATGAAAGCTGCCCCAATGAAAGAAGCCAATGTCA

GAGGACAAAGTGGCCTGGCTTATCCGGGTCTTCGGACCCACGGTACTCTGGAGAACATAG

GTGGGCCGATAGGTGG---TTCACGAGGAGGTGGACTTCCATCACTGACAGACACCTTTG

AGCAGGTAATAGAGGAACTCCTAGAAGAGGAACAGACCATGAGGCAGAGTGAAGAGAACA

AGGAC---GACATGTATACATCTAGAGTCATGCTGAGCACTCAAGTGCCTTTGGAGCCAC

CTTTGCTTTTTCTTCTTGAGGAGTACAAAAACTACTTGGATGCTGCAAACATGTCCATGA

GGGTGAGGCGCCACTCTGACCCGGCTCGGCGCGGGGAACTGAGCGTCTGTGACAGTATTA

GCGAATGGGTGACTGCGGCCGACAAGAAAACTGCAGTGGACATGGCGGGGCAAACGGTTA

CCGTTCTGGAAAAAGTCCCAGTACCCAAAGGTCAACTGAAACAATATTTCTATGAGACCA

AATGCAATCCTATGGGCTACATGAAGGATGGTTGCAGGGGCATTGACAAAAGGTACTGGA

ACTCGCAATGCCGAACTACCCAGTCTTATGTGCGTGCGCTCACCATGGATAGCAAAAAAA

AAGTGGGCTGGCGCTTTATAAGGATAGACACTTCTTGTGTATGTACACTGACCATTAAA-

>M. mukhlesuri MN534414

--ATGGTTATTTCATACTTAAGTTGCATGAAAGCTGCCCCAATGAAAGAAGCCAATGTCA

GAGGACAAAGTGGCCTGGCTTATCCGGGTCTTCGGACCCACGGTACTCTGGAGAACATAG

GTGGGCCGATAGGTGG---TTCACGAGGAGGTGGACTTCCATCGCTGACAGACACCTTTG

AGCAGGTAATAGAGGAACTCCTAGAAGAGGAACAGACCATGAGGCAGAGTGAAGAGAACA

AGGAC---GACATGTATACATCTAGAGTCATGCTGAGCACTCAAGTGCCTTTGGAGCCAC

CTTTGCTTTTTCTTCTTGAGGAGTACAAAAACTACTTGGATGCTGCAAACATGTCCATGA

GGGTGAGGCGCCACTCTGACCCGGCTCGGCGCGGGGAACTGAGCGTCTGTGACAGTATTA

GCGAATGGGTGACTGCGGCCGACAAGAAAACTGCAGTGGACATGGCGGGGCAAACGGTTA

CCGTTCTGGAAAAAGTCCCAGTACCCAAAGGTCAACTGAAACAATATTTCTATGAGACCA

AATGCAATCCTATGGGCTACATGAAGGATGGTTGCAGGGGCATTGACAAAAGGTACTGGA

ACTCGCAATGCCGAACTACCCAGTCTTATGTGCGTGCGCTCACCATGGATAGCAAAAAAA

AAGTGGGCTGGCGCTTTATAAGGATAGACACTTCTTGTGTATGTACACTGACCATTAAA-

>M. mukhlesuri MN534415

--ATGGTTATTTCATACTTAAGTTGCATGAAAGCTGCCCCAATGAAAGAAGCCAATGTCA

GAGGACAAAGTGGCCTGGCTTATCCGGGTCTTCGGACCCACGGTACTCTGGAGAACATAG

GTGGGCCGATAGGTGG---TTCACGAGGAGGTGGACTTCCATCGCTGACAGATACCTTTG

AGCAGGTAATAGAGGAACTCCTAGAAGAGGAACAGACCATGAGGCAGAGTGAAGAGAACA

AGGAC---GACATGTATACATCTAGAGTCATGCTGAGCACTCAAGTGCCTTTGGAGCCAC

CTTTGCTTTTTCTTCTTGAGGAGTACAAAAACTACTTGGATGCTGCAAACATGTCCATGA

GGGTGAGGCGCCACTCTGACCCGGCTCGGCGCGGGGAACTGAGCGTCTGTGACAGTATTA

GCGAATGGGTGACTGCGGCCGACAAGAAAACTGCAGTGGACATGGCGGGGCAAACGGTTA

CCGTTCTGGAAAAAGTCCCAGTACCCAAAGGTCAACTGAAACAATATTTCTATGAGACCA

AATGCAATCCTATGGGCTACATGAAGGATGGTTGCAGGGGCATTGACAAAAGGTACTGGA

ACTCGCAATGCCGAACTACCCAGTCTTATGTGCGTGCGCTCACCATGGATAGCAAAAAAA

AAGTGGGCTGGCGCTTTATAAGGATAGACACTTCTTGTGTATGTACACTGACCATTAAA-

>M. mukhlesuri MN534416

--ATGGTTATTTCATACTTAAGTTGCATGAAAGCTGCCCCAATGAAAGAAGCCAATGTCA

GAGGACAAAGTGGCCTGGCTTATCCGGGTCTTCGGACCCACGGTACTCTGGAGAACATAG

GTGGGCCGATAGGTGG---TTCACGAGGAGGTGGACTTCCATCGCTGACAGATACCTTTG

AGCAGGTAATAGAGGAACTCCTAGAAGAGGAACAGACCATGAGGCAGAGTGAAGAGAACA

AGGAC---GACATGTATACATCTAGAGTCATGCTGAGCACTCAAGTGCCTTTGGAGCCAC

CTTTGCTTTTTCTTCTTGAGGAGTACAAAAACTACTTGGATGCTGCAAACATGTCCATGA

GGGTGAGGCGCCACTCTGACCCGGCTCGGCGCGGGGAACTGAGCGTCTGTGACAGTATTA

GCGAATGGGTGACTGCGGCCGACAAGAAAACTGCAGTGGACATGGCGGGGCAAACGGTTA

CCGTTCTGGAAAAAGTCCCAGTACCCAAAGGTCAACTGAAACAATATTTCTATGAGACCA

AATGCAATCCTATGGGCTACATGAAGGATGGTTGCAGGGGCATTGACAAAAGGTACTGGA

ACTCGCAATGCCGAACTACCCAGTCTTATGTGCGTGCGCTCACCATGGATAGCAAAAAAA

AAGTGGGCTGGCGCTTTATAAGGATAGACACTTCTTGTGTATGTACACTGACCATTAAA-

>M. mukhlesuri MN534417

--ATGGTTATTTCATACTTAAGTTGCATGAAAGCTGCCCCAATGAAAGAAGCCAATGTCA

GAGGACAAAGTGGCCTGGCTTATCCGGGTCTTCGGACCCACGGTACTCTGGAGAACATAG

GTGGGCCGATAGGTGG---TTCACGAGGAGGTGGACTTCCATCGCTGACAGATACCTTTG

AGCAGGTAATAGAGGAACTCCTAGAAGAGGAACAGACCATGAGGCAGAGTGAAGAGAACA

AGGAC---GACATGTATACATCTAGAGTCATGCTGAGCACTCAAGTGCCTTTGGAGCCAC

CTTTGCTTTTTCTTCTTGAGGAGTACAAAAACTACTTGGATGCTGCAAACATGTCCATGA

GGGTGAGGCGCCACTCTGACCCGGCTCGGCGCGGGGAACTGAGCGTCTGTGACAGTATTA

GCGAATGGGTGACTGCGGCCGACAAGAAAACTGCAGTGGACATGGCGGGGCAAACGGTTA

CCGTTCTGGAAAAAGTCCCAGTACCCAAAGGTCAACTGAAACAATATTTCTATGAGACCA

AATGCAATCCTATGGGCTACATGAAGGATGGTTGCAGGGGCATTGACAAAAGGTACTGGA

ACTCGCAATGCCGAACTACCCAGTCTTATGTGCGTGCGCTCACCATGGATAGCAAAAAAA

AAGTGGGCTGGCGCTTTATAAGGATAGACACTTCTTGTGTATGTACACTGACCATTAAA-

>M. mukhlesuri MN534418

--ATGGTTATTTCATACTTAAGTTGCATGAAAGCTGCCCCAATGAAAGAAGCCAATGTCA

GAGGACAAAGTGGCCTGGCTTATCCGGGTCTTCGGACCCACGGTACTCTGGAGAACATAG

GTGGGCCGATAGGTGG---TTCACGAGGAGGTGGACTTCCATCGCTGACAGATACCTTTG

AGCAGGTAATAGAGGAACTCCTAGAAGAGGAACAGACCATGAGGCAGAGTGAAGAGAACA

AGGAC---GACCTGTATACATCTAGAGTCATGCTGAGCACTCAAGTGCCTTTGGAGCCAC

CTTTGCTTTTTCTTCTTGAGGAGTACAAAAACTACTTGGATGCTGCAAACATGTCCATGA

GGGTGAGGCGCCACTCTGACCCGGCTCGGCGCGGGGAACTGAGCGTCTGTGACAGTATTA

GCGAATGGGTGACTGCGGCCGACAAGAAAACTGCAGTGGACATGGCGGGGCAAACGGTTA

CCGTTCTGGAAAAAGTCCCAGTACCCAAAGGTCAACTGAAACAATATTTCTATGAGACCA

AATGCAATCCTATGGGCTACATGAAGGATGGTTGCAGGGGCATTGACAAAAGGTACTGGA

ACTCGCAATGCCGAACTACCCAGTCTTATGTGCGTGCGCTCACCATGGATAGCAAAAAAA

AAGTGGGCTGGCGCTTTATAAGGATAGACACTTCTTGTGTATGTACACTGACCATTAAA-

>M. fissipes MN534419

--ATGGTTATTTCATACTTAAGTTGCATGAAAGCTGCCCCAATGAAAGAAGCCAATGTCA

GAGGACAAAGTGGCCTGGCTTATCCGGGTCTTCGGACCCACGGTACTTTGGAGAACATAG

GTGGGCCGATAGGTGG---TTCACGAGGAGGTGGACTTCCATCGCTGACAGACACCTTTG

AGCAGGTAATAGAGGAACTCCTAGAAGAGGAACAGACCATGAGGCAGAGTGAAGAGAACA

AGGAC---GACATGTATACATCTAGAGTCATGCTGAGCACTCAAGTGCCTTTGGAGCCAC

CTTTGCTTTTTCTTCTTGAGGAGTACAAAAACTACTTGGATGCTGCAAACATGTCCATGA

GGGTGAGGCGCCACTCTGACCCGGCTCGGCGCGGGGAACTGAGCGTCTGTGACAGTATTA

GCGAATGGGTGACTGCGGCCGACAAGAAAACTGCAGTGGACATGGCGGGGCAAACAGTTA

CCGTTCTGGAAAAAGTCCCAGTACCCAAAGGTCAACTGAAACAATATTTCTATGAGACCA

AATGCAATCCTATGGGCTACATGAAGGATGGTTGCAGGGGCATTGACAAAAGGTACTGGA

ACTCGCAATGCCGAACTACCCAGTCTTATGTGCGTGCGCTCACCATGGATAGCAAAAAAA

AAGTGGGCTGGCGCTTTATAAGGATAGACACTTCTTGTGTATGTACACTGACCATTAAA-

>M. fissipes MN534420

--ATGGTTATTTCATACTTAAGTTGCATGAAAGCTGCCCCAATGAAAGAAGCCAATGTCA

GAGGACAAAGTGGCCTGGCTTATCCGGGTCTTCGGACCCACGGTACTCTGGAGAACATAG

GTGGGCCGATAGGTGG---TTCACGAGGAGGTGGACTTCCATCGCTGACAGACACCTTTG

AGCAGGTAATAGAGGAACTCCTAGAAGAGGAACAGACCATGAGGCAGAGTGAAGAGAACA

AGGAC---GACATGTATACATCTAGAGTCATGCTGAGCACTCAAGTGCCTTTGGAGCCAC

CTTTGCTTTTTCTTCTTGAGGAGTACAAAAACTACTTGGATGCTGCAAACATGTCCATGA

GGGTGAGGCGCCACTCTGACCCGGCTCGGCGCGGGGAACTGAGCGTCTGTGACAGTATTA

GCGAATGGGTGACTGCGGCCGACAAGAAAACTGCAGTGGACATGGCGGGGCAAACAGTTA

CCGTTCTGGAAAAAGTCCCAGTACCCAAAGGTCAACTGAAACAATATTTCTATGAGACCA

AATGCAATCCTATGGGCTACATGAAGGATGGTTGCAGGGGCATTGACAAAAGGTACTGGA

ACTCGCAATGCCGAACTACCCAGTCTTATGTGCGTGCGCTCACCATGGATAGCAAAAAAA

AAGTGGGCTGGCGCTTTATAAGGATAGACACTTCTTGTGTATGTACACTGACCATTAAA-

>M. chakrapanii MN534421

--ATGGTTATTTCATACTTAAGTTGCATGAAAGCTGCCCCAATGAAAGAAGCCAATGTCA

GAGGACAAAGTGGCCTGGCTTATCCGGGTCTTCGGACCCACGGTACTCTGGAGAACATAG

GTGGGCCGATAGGTGG---TTCACGAGGAGGTGGACTTCCATCGCTGACAGACACCTTTG

AGCAGGTAATAGAGGAACTTCTAGAAGAGGAACAGACCATGAGGCAGAGTGAAGAGAACA

AGGAC---GACATGTATACATCTAGAGTCATGCTGAGCACTCAAGTGCCTTTGGAGCCAC

CTTTGCTTTTTCTTCTTGAGGAGTACAAAAACTACTTGGATGCTGCAAACATGTCCATGA

GGGTGAGGCGCCACTCCGACCCGGCTCGGCGCGGGGAACTGAGCGTCTGTGACAGTATTA

GCGAATGGGTGACTGCGGCCGACAAGAAAACTGCAGTGGACATGGCGGGGCAAACGGTTA

CCGTTCTGGAAAAAGTCCCAGTACCCAAAGGTCAACTGAAACAATATTTCTATGAGACCA

AATGCAATCCTATGGGCTACATGAAGGATGGTTGCAGGGGCATTGACAAAAGGTACTGGA

ACTCGCAATGCCGAACTACCCAGTCTTATGTGCGTGCGCTCACCATGGATAGCAAAAAAA

AAGTGGGCTGGCGCTTTATAAGGATAGACACTTCTTGTGTATGTACACTGACCATTAAA-

>M. chakrapanii MN534422

--ATGGTTATTTCATACTTAAGTTGCATGAAAGCTGCCCCAATGAAAGAAGCCAATGTCA

GAGGACAAAGTGGCCTGGCTTATCCGGGTCTTCGGACCCACGGTACTCTGGAGAACATAG

GTGGGCCGATAGGTGG---TTCACGAGGAGGTGGACTTCCATCGCTGACAGACACCTTTG

AGCAGGTAATAGAGGAACTTCTAGAAGAGGAACAGACCATGAGGCAGAGTGAAGAGAACA

AGGAC---GACATGTATACATCTAGAGTCATGCTGAGCACTCAAGTGCCTTTGGAGCCAC

CTTTGCTTTTTCTTCTTGAGGAGTACAAAAACTACTTGGATGCTGCAAACATGTCCATGA

GGGTGAGGCGCCACTCCGACCCGGCTCGGCGCGGGGAACTGAGCGTCTGTGACAGTATTA

GCGAATGGGTGACTGCGGCCGACAAGAAAACTGCAGTGGACATGGCGGGGCAAACGGTTA

CCGTTCTGGAAAAAGTCCCAGTACCCAAAGGTCAACTGAAACAATATTTCTATGAGACCA

AATGCAATCCTATGGGCTACATGAAGGATGGTTGCAGGGGCATTGACAAAAGGTACTGGA

ACTCGCAATGCCGAACTACCCAGTCTTATGTGCGTGCGCTCACCATGGATAGCAAAAAAA

AAGTGGGCTGGCGCTTTATAAGGATAGACACTTCTTGTGTATGTACACTGACCATTAAA-

>M. mymensinghensis MN534423

--ATGGTTATTTCATACTTAAGTTGCATGAAAGCTGCCCCAATGAAAGAAGCCAATGTCA

GAGGACAAAGTGGCCTGGCTTATCCGGGTCTTCGGACCCACGGTACTCTGGAGAACATAG

GTGGGCCAATAGGTGG---TTCACGAGGCGGTGGACTTCCATCGCTGACAGACACCTTTG

AGCAGGTAATAGAGGAACTTCTAGAAGAGGAACAGACCATGAGGCAGAGTGAAGAGAACA

AGGAC---GACATGTATACATCTAGAGTCATGCTGAGCACTCAAGTGCCTTTGGAGCCAC

CTTTGCTTTTTCTTCTTGAGGAGTACAAAAACTACTTGGATGCTGCAAACATGTCCATGA

GGGTGAGGCGCCACTCCGACCCGGCTCGGCGCGGGGAACTGAGCGTCTGTGACAGTATTA

GCGAATGGGTGACTGCGGCCGACAAGAAAACTGCAGTGGACATGGCGGGGCAAACGGTTA

CCGTTCTGGAAAAAGTCCCAGTACCCAAAGGTCAACTGAAACAATATTTCTATGAGACCA

AATGCAATCCTATGGGCTACATGAAGGATGGTTGCAGGGGCATTGACAAAAGGTACTGGA

ACTCGCAATGCCGAACTACCCAGTCTTATGTGCGTGCGCTCACCATGGATAGCAAAAAAA

AAGTGGGCTGGCGCTTTATAAGGATAGACACTTCTTGTGTATGTACACTGACCATTAAA-

>Microhyla sp. 3 MN534424

--------------------------------GCTGCCCCAATGAAAGAAGCCAATGTCA

GAGGACAAAGTGGCCTGGCTTATCCGGGTCTTCGGACCCACGGTACTCTGGAGAACATAG

GTGGGCCGATAGGTGG---TTCAAGAGGAGGTGGACTTCCATCGCTGACAGATACCTTTG

AGCAGGTTATAGAGGAACTCCTAGAAGAGGAACAGACCATGAGGCAGAGTGAAGAGAACA

AGGAC---GACATGTATTCATCTAGAGTAATGCTGAGCACTCAAGTGCCTTTGGAGCCAC

CATTGCTTTTTCTCCTTGAGGAGTACAAAAACTACTTGGATGCTGCAAACATGTCCATGA

GGGTGAGGCGCCACTCCGACCCCGCTCGGCGCGGGGAACTGAGCGTCTGTGACAGTATTA

GCGAATGGGTGACTGCGGCCGACAAGAAAACCGCAGTGGACATGGCGGGGCAAACGGTTA

CTGTTCTGGAAAAAGTCCCAGTACCCAAAGGTCAACTGAAACAATATTTCTATGAGACCA

AATGCAATCCTATGGGCTACATGAAGGAAGGTTGCAGGGGCATTGACAAAAGGTACTGGA

ACTCGCAATGCCGAACTACCCAGTCTTATGTGCGTGCGCTCACCATGGATAGCAAAAAAA

AAGTTGGCTGGCGCTTTATAAGGATAGACACTTCTTGTGTATGTACACTGACCATTAAAA

>Microhyla sp. 3 MN534425

--------------------------------GCTGCCCCAATGAAAGAAGCCAATGTCA

GAGGACAAAGTGGCCTGGCTTATCCGGGTCTTCGGACCCACGGTACTCTGGAGAACATAG

GTGGGCCGATAGGTGG---TTCAAGAGGAGGTGGACTTCCATCGCTGACAGATACCTTTG

AGCAGGTTATAGAGGAACTCCTAGAAGAGGAACAGACCATGAGGCAGAGTGAAGAGAACA

AGGAC---GACATGTATTCATCTAGAGTAATGCTGAGCACTCAAGTGCCTTTGGAGCCAC

CATTGCTTTTTCTCCTTGAGGAGTACAAAAACTACTTGGATGCTGCAAACATGTCCATGA

GGGTGAGGCGCCACTCCGACCCCGCTCGGCGCGGGGAACTGAGCGTCTGTGACAGTATTA

GCGAATGGGTGACTGCGGCCGACAAGAAAACCGCAGTGGACATGGCGGGGCAAACGGTTA

CTGTTCTGGAAAAAGTCCCAGTACCCAAAGGTCAACTGAAACAATATTTCTATGAGACCA

AATGCAATCCTATGGGCTACATGAAGGAAGGTTGCAGGGGCATTGACAAAAGGTACTGGA

ACTCGCAATGCCGAACTACCCAGTCTTATGTGCGTGCGCTCACCATGGATAGCAAAAAAA

AAGTTGGCTGGCGCTTTATAAGGATAGACACTTCTTGTGTATGTACACTGACCATTAAAA

>M. okinavensis MN534426

--ATGGTTATTTCATACTTAAGTTGCATGAAAGCTGCCCCAATGAAAGAAGCCAATGTCA

GAGGACAAAGTGGCCTGGCTTATCCGGGTCTTCGGACCCACGGTACTCTGGAGAACATAG

GTGGGCCGATAGGTGG---TTCAAGAGGAGGTGGACTTCCATCGCTGACAGATACCTTTG

AGCAGGTTATAGAGGAACTCCTAGAAGAGGAACAGACCATGAGGCAGAGTGAAGAGAACA

AGGAC---GACATGTATTCATCTAGAGTAATGCTGAGCACTCAAGTGCCTTTGGAGCCAC

CATTGCTTTTTCTCCTTGAGGAGTACAAAAACTACTTGGATGCTGCAAACATGTCCATGA

GGGTGAGGCGCCACTCCGACCCCGCTCGGCGCGGGGAACTGAGCGTCTGTGACAGTATTA

GCGAATGGGTGACTGCGGCCGACAAGAAAACCGCAGTGGACATGGCGGGGCAAACGGTTA

CTGTTCTGGAAAAAGTCCCAGTACCCAAAGGTCAACTGAAACAATATTTCTATGAGACCA

AATGCAATCCTATGGGCTACATGAAGGAAGGTTGCAGGGGCATTGACAAAAGGTACTGGA

ACTCGCAATGCCGAACTACCCAGTCTTATGTGCGTGCGCTCACCATGGATAGCAAAAAAA

AAGTTGGCTGGCGCTTTATAAGGATAGACACTTCTTGTGTATGTACACTGACCATTAAA-

>M. picta MN534427

CTATGGTTATTTCATACTTAAGTTGCATGAAAGCTGCCCCAATGAAAGAAGCCAATGTCA

GAGGACAAAGTGGCCTGGCTTATCCGGGTCTTCGGACCCACGGTACTCTGGAGAACATAG

GTGGGCCGATAGGTGG---TTCAAGAGGAGGTGGACTTCCATCGCTGACAGACACCTTTG

AGCAGGTTATAGAGGAGCTCCTAGAAGAGGAACAGACCATGAGGCAGAGTGAAGAGAACA

AGGAC---GACATGTATTCTTCTAGAGTAATGCTGAGCACTCAAGTGCCTTTGGAGCCAC

CTTTGCTTTTTCTCCTTGAGGAGTACAAAAACTACTTGGATGCTGCAAACATGTCCATGA

GGGTGAGGCGCCACTCTGATCCCGCTCGGCGCGGGGAACTGAGCGTCTGTGACAGTATTA

GCGAATGGGTGACTGCGGCCGACAAGAAAACTGCAGTGGACATGGCGGGGCAAACGGTTA

CAGTTCTGGAAAAAGTCCCAGTACCCAAAGGTCAACTGAAACAATATTTCTATGAGACCA

AATGCAATCCCATGGGCTACATGAAGGAAGGTTGCAGGGGCATTGACAAAAGGTACTGGA

ACTCGCAATGCCGAACTACCCAGTCTTATGTGCGTGCGCTCACCATGGATAGCAAAAAAA

AAGTTGGCTGGCGCTTTATAAGGATAGACACTTCTTGTGTATGTACACTGACCATTAAA-

>M. ornata MN534428

--ATGGTTATTTCATACTTAAGTTGCATGAAAGCTGCCCCAATGAAAGAAGCCAATGTCA

GAGGACAAAGTGGCCTGGCTTATCCGGGTCTTCGGACCCATGGTACTCTGGAGAACATAG

GTGGGCCGATAGGTGG---TTCAAGAGGAGGTGGACTTCCATCGCTGACAGATACATTTG

AGCAGGTCATAGAGGAGCTCCTAGAAGAGGAACAGACCATGAGGCAGAGTGAAGAGAACA

AGGAC---GACATGTATTCATCTAGAGTAATGCTGAGCACTCAAGTGCCTTTGGAGCCAC

CTTTGCTTTTTCTCCTTGAGGAGTACAAAAACTACTTGGATGCTGCAAACATGTCCATGA

GGGTGAGGCGCCACTCTGACCCCGCTCGACGTGGGGAACTAAGCGTCTGTGACAGTATTA

GTGAATGGGTGACTGCGGCCGACAAGAAAACTGCAGTGGACATGGCGGGGCAAACAGTTA

CTGTTCTCGAAAAAGTCCCAGTACCCAAAGGTCAACTGAAACAATATTTCTATGAGACCA

AATGCAATCCTATGGGCTACATGAAGGAAGGTTGCAGGGGCATTGACAAAAGGTACTGGA

ACTCGCAATGCCGAACTACCCAGTCTTATGTGCGTGCGCTCACCATGGATAGCAAAAAAA

AAGTTGGCTGGCGCTTTATAAGGATAGACACTTCTTGTGTATGTACA-------------

>M. rubra MN534429

--ATGGTTATTTCATACTTAAGTTGCATGAAAGCTGCCCCAATGAAAGAAGCCAATGTCA

GAGGACAAAGTGGCCTGGCTTATCCGGGTCTTCGGACCCATGGTACTCTGGAGAACATAG

GTGGGCCGATAGGTGG---TTCAAGAGGAGGTGGACTTCCATCGCTGACAGATACCTTTG

AGCAGGTCATAGAGGAGCTCCTAGAAGAGGAACAGACCATGAGGCAGAGTGAAGAGAACA

AGGAC---GACATGTATTCATCTAGAGTAATGCTGAGCACTCAAGTGCCTTTGGAGCCAC

CTTTGCTTTTTCTCCTTGAGGAGTACAAAAACTACTTGGATGCTGCAAACATGTCCATGA

GGGTGAGGCGCCACTCTGACCCAGCTCGACGTGGGGAGCTAAGCGTCTGTGACAGTATTA

GTGAATGGGTGACTGCGGCCGACAAGAAAACTGCAGTGGACATGGCGGGGCAAACAGTTA

CTGTTCTCGAAAAAGTCCCAGTACCCAAAGGTCAACTGAAACAATATTTCTATGAGACCA

AATGCAATCCTATGGGCTACATGAAGGAAGGTTGCAGGGGCATTGACAAAAGGTACTGGA

ACTCGCAATGCCGAACTACCCAGTCTTATGTGCGTGCGCTCACCATGGATAGCAAAAAAA

AAGTTGGCTGGCGCTTTATAAGGATAGACACTTCTTGTGTATGTACACTGACCATTAAA-

>M. mihintalei MN534430

CTATGGTTATTTCATACTTAAGTTGCATGAAAGCTGCCCCAATGAAAGAAGCCAATGTCA

GAGGACAAAGTGGCCTGGCTTATCCGGGTCTTCGGACCCATGGTACTCTGGAGAACATAG

GTGGGCCGATAGGTGG---TTCAAGAGGAGGTGGACTTCCATCGCTGACAGATACCTTTG

AGCAGGTCATAGAGGAGCTCCTAGAAGAGGAACAGACCATGAGGCAGAGTGAAGAGAACA

AGGAC---GACATGTATTCATCTAGAGTAATGCTGAGCACTCAAGTGCCTTTGGAGCCAC

CTTTGCTTTTTCTCCTTGAGGAGTACAAAAACTACTTGGATGCTGCAAACATGTCCATGA

GGGTGAGGCGCCACTCTGACCCCGCTCGACGTGGGGAGCTAAGCGTCTGTGACAGTATTA

GTGAATGGGTGACTGCGGCCGACAAGAAAACTGCAGTGGACATGGCGGGGCAAACAGTTA

CTGTTCTCGAAAAAGTCCCAGTACCCAAAGGTCAACTGAAACAATATTTCTATGAGACCA

AATGCAATCCTATGGGCTACATGAAGGAAGGTTGCAGGGGCATTGACAAAAGGTACTGGA

ACTCGCAATGCCGAACTACCCAGTCTTATGTGCGTGCGCTCACCATGGATAGCAAAAAAA

AAGTTGGCTGGCGCTTTATAAGGATAGACACTTCTTGTGTATGTACACTGACCATTAAAA

>M. aurantiventris MN534431

CTATGGTTATTTCATACTTAAGTTGCATGAAAGCTGCCCCAATGAAAGAAACCAATGTCA

GAGGACAAAGTGGCCTGGCTTATCCGGGTCTTCGGACCCATGGTACTCTGGAGAACATAG

GCGGGCCGATAGGTGG---TTCAAGAGGAGGTGGACTTCCATCGCTGACCGATACCTTTG

AGCAGGTTATAGAGGAACTCCTGGAAGAGGAACAGACCATAAGGCAGAGTGAAGAGAACA

AGGAC---GACATGTATTCATCTAGAGTAATGCTGAGCACTCAAGTGCCTTTGGAGCCAC

CTTTGCTTTTTCTCCTTGAGGAGTACAAAAACTACTTGGATGCTGCAAACATGTCCATGA
GGGTGAGGCGCCACTCCGACCCGGCTCGGCGCGGGGAGCTGAGCGTCTGTGACAGTATTA
GTGAATGGGTGACTGCGGCCGACAAAAAAACTGCAGTGGACATGGCGGGGCAAACGGTTT
CTGTTCTCGAAAAAGTCCCAGTACCCAAAGGTCAACTGAAACAATATTTCTATGAGACCA
AATGCAATCCTATGGGCTACATGAAGGAAGGTTGCAGGGGCATTGACAAAAGGTACTGGA
ACTCGCAATGCCGAACTACCCAGTCTTATGTGCGTGCGCTCACCATGGATAGCAAAAAAA
AAGTTGGCTGGCGCTTTATAAGGATAGACACTTCTTGTGTATGTACACTGACCATTAAA-

>M. butleri MN534432
CTATGGTTATTTCATACTTAAGTTGCATGAAAGCTGCCCCAATGAAAGAAACCAATGTCA
GAGGACAAAGTGGCCTGGCTTATCCGGGTCTTCGGACCCATGGTACTCTGGAGAACATAG
GCGGGCCGATAGGTGG---TTCAAGAGGAGGTGGACTTCCATCGCTGACCGATACCTTTG
AGCAGGTTATAGAGGAACTCCTGGAAGAGGAACAGACCATAAGGCAGAGTGAAGAAAACA
AGGAC---GACATGTATTCATCTAGAGTAATGCTGAGCACTCAAGTGCCTTTGGAGCCAC
CTTTGCTTTTTCTCCTTGAGGAGTACAAAAACTACTTGGATGCTGCAAACATGTCCATGA
GGGTGAGGCGCCACTCCGACCCCGCTCGGCGCGGGGAACTGAGCGTCTGTGACAGTATTA
GTGAATGGGTGACTGCAGCCGACAAAAAAACTGCAGTGGACATGGCGGGGCAAACGGTCA
CTGTTCTCGAAAAAGTCCCAGTACCCAAAGGTCAACTGAAACAATATTTCTATGAGACCA
AATGCAATCCTATGGGCTACATGAAGGAAGGTTGCAGGGGCATTGACAAAAGGTACTGGA
ACTCGCAATGCCGAACTACCCAGTCTTATGTGCGTGCGCTCACCATGGATAGCAAAAAAA
AAGTTGGCTGGCGCTTTATAAGGATAGACACTTCTTGTGTATGTACACTGACCATTAAA-

>M. butleri MN534433
CTATGGTTATTTCATACTTAAGTTGCATGAAAGCTGCCCCAATGAAAGAAACCAATGTCA
GAGGACAAAGTGGCCTGGCTTATCCGGGTCTTCGGACCCATGGTACTCTGGAGAACATAG
GCGGGCCGATAGGTGG---TTCAAGAGGAGGTGGACTTCCATCGCTGACCGATACCTTTG
AGCAGGTTATAGAGGAACTCCTGGAAGAGGAACAGACCATAAGGCAGAGTGAAGAGAACA
AGGAC---GACATGTATTCATCTAGAGTAATGCTGAGCACTCAAGTGCCTTTGGAGCCAC
CTTTGCTTTTTCTCCTTGAGGAGTACAAAAACTACTTGGATGCTGCAAACATGTCCATGA
GGGTGAGGCGCCACTCCGACCCCGCTCGGCGCGGGGAACTGAGCGTCTGTGACAGTATTA

GTGAATGGGTGACTGCGGCCGACAAAAAAACTGCAGTGGACATGGCGGGGCAAACGGTTA

CTGTTCTCGAAAAAGTCCCAGTACCCAAAGGTCAACTGAAACAATATTTCTATGAGACCA

AATGCAATCCTATGGGCTACATGAAGGAAGGTTGCAGGGGCATTGACAAAAGGTACTGGA

ACTCGCAATGCCGAACTACCCAGTCTTATGTGCGTGCGCTCACCATGGATAGCAAAAAAA

AAGTTGGCTGGCGCTTTATAAGGATAGACACTTCTTGTGTATGTACACTGACCATTAAA-

>M. butleri MN534434

CTATGGTTATTTCATACTTAAGTTGCATGAAAGCTGCCCCAATGAAAGAAACCAATGTCA

GAGGACAAAGTGGCCTGGCTTATCCGGGTCTTCGGACCCATGGTACTCTGGAGAACATAG

GCGGGCCGATAGGTGG---TTCAAGAGGAGGTGGACTTCCATCGCTGACCGATACCTTTG

AGCAGGTTATAGAGGAACTACTGGAAGAGGAACAGACCATAAGGCAGAGTGAAGAGAACA

AGGAC---GACATGTATTCATCTAGAGTAATGCTGAGCACTCAAGTGCCTTTGGAGCCAC

CTTTGCTTTTTCTCCTTGAGGAGTACAAAAACTACTTGGATGCTGCAAACATGTCCATGA

GGGTGAGGCGCCACTCCGACCCTGCAAGGCGCGGGGAACTGAGCGTCTGTGACAGTATTA

GTGAATGGGTGACTGCGGCCGACAAAAAAACTGCAGTGGACATGGCGGGGCAAACGGTCA

CTGTTCTCGAAAAAGTCCCAGTACCCAAAGGTCAACTGAAACAATATTTCTATGAGACCA

AATGCAATCCTATGGGCTACATGAAGGAAGGTTGCAGGGGCATTGACAAAAGGTACTGGA

ACTCGCAATGCCGAACTACCCAGTCTTATGTGCGTGCGCTCACCATGGATAGCAAAAAAA

AAGTTGGCTGGCGCTTTATAAGGATAGACACTTCTTGTGTATGTACACTGACCATTAAA-

>M. butleri MN534435

CTATGGTTATTTCATACTTAAGTTGCATGAAAGCTGCCCCAATGAAAGAAACCAATGTCA

GAGGACAAAGTGGCCTGGCTTATCCGGGTCTTCGGACCCATGGTACTCTGGAGAACATAG

GCGGGCCGATAGGTGG---TTCAAGAGGAGGTGGACTTCCATCGCTGACCGATACCTTTG

AGCAGGTTATAGAGGAACTACTGGAAGAGGAACAGACCATAAGGCAGAGTGAAGAGAACA

AGGAC---GACATGTATTCATCTAGAGTAATGCTGAGCACTCAAGTGCCTTTGGAGCCAC

CTTTGCTTTTTCTCCTTGAGGAGTACAAAAACTACTTGGATGCTGCAAACATGTCCATGA

GGGTGAGGCGCCACTCCGACCCCGCTCGGCGCGGGGAACTGAGCGTCTGTGACAGTATTA

GTGAATGGGTGACTGCGGCCGACAAAAAAACTGCAGTGGACATGGCGGGGCAAACGGTCA

CTGTTCTCGAAAAAGTCCCAGTACCCAAAGGTCAACTGAAACAATATTTCTATGAGACCA

AATGCAATCCTATGGGCTACATGAAGGAAGGTTGCAGGGGCATTGACAAAAGGTACTGGA

ACTCGCAATGCCGAACTACCCAGTCTTATGTGCGTGCGCTCACCATGGATAGCAAAAAAA

AAGTTGGCTGGCGCTTTATAAGGATAGACACTTCTTGTGTATGTACACTGACCATTAAA-

>M. butleri MN534436

CTATGGTTATTTCATACTTAAGTTGCATGAAAGCTGCCCCAATGAAAGAAACCAATGTCA

GAGGACAAAGTGGCCTGGCTTATCCGGGTCTTCGGACCCATGGTACTCTGGAGAACATAG

GCGGGCCGATAGGTGG---TTCAAGAGGAGGTGGACTTCCATCGCTGACCGATACCTTTG

AGCAGGTTATAGAGGAACTCCTGGAAGAGGAACAGACCATAAGGCAGAGTGAAGAGAACA

AGGAC---GACATGTATTCATCTAGAGTAATGCTGAGCACTCAAGTGCCTTTGGAGCCAC

CTTTGCTTTTTCTCCTTGAGGAGTACAAAAACTACTTGGATGCTGCAAACATGTCCATGA

GGGTGAGGCGCCACTCCGACCCCGCTCGGCGCGGGGAACTGAGCGTCTGTGACAGTATTA

GTGAATGGGTGACTGCGGCCGACAAAAAAACTGCAGTGGACATGGCGGGGCAAACGGTCA

CTGTTCTCGAAAAAGTCCCAGTACCCAAAGGTCAACTGAAACAATATTTCTATGAGACCA

AATGCAATCCTATGGGCTACATGAAGGAAGGTTGCAGGGGCATTGACAAAAGGTACTGGA

ACTCGCAATGCCGAACTACCCAGTCTTATGTGCGTGCGCTCACCATGGATAGCAAAAAAA

AAGTTGGCTGGCGCTTTATAAGGATAGACACTTC--------------------------

>M. zeylanica MN534437

--ATGGTTATTTCATACTTAAGTTGCATGAAAGCTGCCCCAATGAAAGAAGCCAATGTCA

GAGGACAAAGTGGCCTGGCTTATCCGGGTCTTCGGACCCATGGTACTCTGGAGAACATAG

GCGGGCCGATAGGTGG---TTCAAGAGGAGGTGGACTTCCATCGCTGACAGACACCTTTG

AGCAGGTTATAGAGGAACTCCTGGAAGAGGAACAGACCATAAGGCAGAGTGAAGAGAACA

AGGAC---GACATGTATTCTTCTAGAGTAATGCTGAGCACTCAAGTGCCTTTGGAGCCAC

CTTTGCTTTTTCTTCTTGAGGAGTACAAAAACTACTTGGATGCGGCAAACATGTCCATGC

GGGTGAGGCGCCACTCCGACCCCGCTCGGCGCGGGGAACTGAGCGTCTGTGACAGTATTA

GTGAATGGGTGACTGCGGCCGACAAGAAAACTGCAGTGGACATGGCGGGGCAAACAGTTA

CTGTTCTCGAAAAAGTCCCAGTACCCAAAGGTCAACTGAAACAATATTTCTATGAGACCA

AATGCAATCCTATGGGCTACATGAAGGAAGGTTGCAGGGGCATAGACAAAAGGCACTGGA

AA--

>M. karunaratnei MN534438

--ATGGTTATTTCATACTTAAGTTGCATGAAAGCTGCCCCAATGAAAGAAGCCAATGTCA

GAGGACAAAGTGGCCTGGCTTATCCGGGTCTTCGGACCCATGGTACTCTGGAGAACATAG

GCGGGCCGATAGGTGG---TTCAAGAGGAGGTGGACTTCCATCGCTGACAGACACCTTTG

AGCAGGTTATAGAGGAACTCCTGGAAGAGGAACAGACCATAAGGCAGAGTGAAGAGAACA

AGGAC---GACATGTATTCATCTAGAGTAATGCTGAGCACTCAAGTGCCTTTGGAGCCAC

CTTTGCTTTTTCTTCTTGAGGAGTACAAAAACTACTTGGATGCGGCAAACATGTCCATGC

GGGTGAGGCGCCACTCCGACCCCGCTCGGCGCGGGGAACTGAGCGTCTGTGACAGTATTA

GTGAATGGGTGACTGCGGCCGACAAGAAAACTGCAGTGGACATGGCGGGGCAAACAGTTA

CTGTTCTCGAAAAAGTCCCAGTACCCAAAGGTCAACTGAAACAATATTTCTATGAGACCA

AATGCAATCCTATGGGCTACATGAAGGAAGGTTGCAGGGGCATTGACAAAAGGTACTGGA

ACTCGCAATGCCGAACTACCCAGTCTTACGTGCGTGCGCTCACCATGGATAGCAAAAAAA

AAGTTGGCTGGCGCTTTATAAGGATAGACACTTCTTGCGTATGTACACTGACCATTAAA-

>Microhyla sp. 2 MN534439

--ATGGTTATTTCATACTTAAGTTGCATGAAAGCTGCCCCAATGAAAGAAGCCAATGTCA

GAGGACAAAGTGGCCTGGCTTATCCGGGTCTTCGGACCCATGGTACTCTGGAGAACATAG

GCGGGCCGATAGGTGG---TTCAAGAGGAGGTGGACTTCCATCGCTGACCGATACCTTTG

AGCAGGTTATAGAGGAACTCCTGGAAGAGGAACAGACCATAAGGCAGAGTGAAGAGAACA

AGGAC---GACATGTATTCATCTAGAGTAATGCTGAGCACTCAAGTGCCTTTGGAGCCAC

CTTTGCTTTTTCTTCTTGAGGAGTACAAAAACTACTTGGATGCTGCAAACATGTCCATGA

GGGTGAGGCGCCACTCCGACCCCGCTCGGCGCGGGGAACTGAGCGTCTGTGACAGTATTA

GCGAATGGGTGACTGCGGCCGACAAGAAAACTGCAGTGGACATGGCGGGGCAAACGGTTA

CTGTTCTCGAAAAAGTCCCAGTACCCAAAGGTCAACTGAAACAATATTTCTATGAGACCA

AATGCAATCCTATGGGCTACATGAAGGAAGGTTGCAGGGGCATTGACAAAAGGTACTGGA

ACTCGCAATGCCGAACTACCCAGTCTTATGTGCGTGCGCTCACCATGGATAGCAAAAAAA

AAGTTGGCTGGCGCTTTATAAGGATAGACACTTCTTGTGTATGTACACTGACCATTAAA-

>Microhyla sp. 2 MN534440

--ATGGTTATTTCATACTTAAGTTGCATGAAAGCTGCCCCAATGAAAGAAGCCAATGTCA

GAGGACAAAGTGGCCTGGCTTATCCGGGTCTTCGGACCCATGGTACTCTGGAGAACATAG

GCGGGCCGATAGGTGG---TTCAAGAGGAGGTGGACTTCCATCGCTGACCGATACCTTTG

AGCAGGTTATAGAGGAACTCCTGGAAGAGGAACAGACCATAAGGCAGAGTGAAGAGAACA

AGGAC---GACATGTATTCATCTAGAGTAATGCTGAGCACTCAAGTGCCTTTGGAGCCAC

CTTTGCTTTTTCTTCTTGAGGAGTACAAAAACTACTTGGATGCTGCAAACATGTCCATGA

GGGTGAGGCGCCACTCCGACCCCGCTCGGCGCGGGGAACTGAGCGTCTGTGACAGTATTA

GCGAATGGGTGACTGCGGCCGACAAGAAAACTGCAGTGGACATGGCGGGGCAAACGGTTA

CTGTTCTCGAAAAAGTCCCAGTACCCAAAGGTCAACTGAAACAATATTTCTATGAGACCA

AATGCAATCCTATGGGCTACATGAAGGAAGGTTGCAGGGGCATTGACAAAAGGTACTGGA

ACTCGCAATGCCGAACTACCCAGTCTTATGTGCGTGCGCTCACCATGGATAGCAAAAAAA

AAGTTGGCTGGCGCTTTATAAGGATAGACACTTCTTGTGTATGTACACTGACCATTAAA-

>M. superciliaris MN534441

--------------------------------GCTGCCCCAATGAAAGAAGCCAATGTCA

GAGGACAAAGTGGCCTGGCTTATCCGGGTCTTCGGACCCATGGTACTCTGGAGAACATAG

GCGGGCCGATAGGTGG---TTCAAGAGGAGGTGGACTTCCATCGCTGACAGATACCTTTG

AGCAGGTTATAGAGGAACTCCTGGAAGAGGAACAGACCATAAGACAGAGTGAGGAGAACA

AGGAC---GACATGTATTCATCTAGAGTAATGCTGAGCACTCAAGTGCCTTTGGAGCCAC

CTTTGCTTTTTCTTCTTGAGGAGTACAAAAACTACTTGGATGCTGCAAACATGTCCATGA

GGGTGAGGCGCCACTCCGACCCCGCTCGGCGCGGGGAACTGAGCGTCTGTGACAGTATTA

GTGAGTGGGTGACTGCGGCCGACAAGAAAACTGCAGTGGACATGGCGGGGCAAACAGTTA

CTGTTCTCGAAAAAGTCCCAGTACCCAAAGGTCAACTGAAACAATATTTCTATGAGACCA

AATGCAATCCTATGGGCTACATGAAGGAAGGTTGCAGGGGCATTGACAAAAGGTACTGGA

ACTCGCAATGCCGAACTACCCAGTCTTA--------------------------------

>M. annectens MN534442

CTATGGTTATTTCATACTTAAGTTGCATGAAAGCTGCCCCAATGAAGGAAGCCAATGTCA

GAGGACAAAGTGGCCTGGCTTATCCGGGTCTTCGGACCCACGGTACTCTGGAGAACATAG

GTGGGCCGGTAGGTGG---TTCAAGAGGAGGTGGACTTCCATCCCTGACAGATACCTTTG

AGCAGGTTATAGAGGAACTCCTAGAAGAGGAACAGACCATAAGGCAGAGTGAGGAGAACA

AGGAC---GACATGTATTCATCTAGAGTTATGCTGAGCACTCAAGTGCCTTTGGAGCCAC

CTTTGCTTTTTCTCCTTGAGGAGTACAAAAACTACTTGGATGCTGCAAACATGTCCATGA

GGGTGAGGCGCCACTCTGACCCCGCTCGACGTGGGGAACTGAGCGTCTGTGACAGTATTA

GCGAATGGGTGACTGCGGCCGACAAGAAAACTGCAGTGGACATGGCGGGGCAAACAGTTA

CTGTTCTCGAAAAAGTCCCAGTACCCAAAGGTCAACTGAAACAATATTTCTATGAGACCA

AATGCAATCCTATGGGCTACATGAAGGAAGGTTGCAGGGGCATTGATAAAAGGTACTGGA

ACTCGCAATGCCGAACTACCCAATCTTATGTGCGTGCGCTCACCATGGATAGCAAAAAAA

AAGTTGGCTGGCGCTTTATAAGGATAGACACTTCTTGTGTATGTACACTGACCATTAAA-

>M. annamensis MN534443

CGATGGTTATTTCATACTTAAGTTGCATGAAAGCTGCCCCAATGAAGGAAGCCAATGTCA

GAGGACAAAGTGGCCTGGCTTATCCGGGTCTTCGGACCCACGGTACTCTGGAGAACATAG

GTGGGCCGATAGGTGG---TTCAAGAGGAGGTGGACTTTCATCACTGACAGATACCTTTG

AGCAGGTTATAGAGGAACTCCTAGAAGAGGAACAGACCATAAGGCAGAGTGAAGAGAACA

AGGAC---GACATGTATTCATCTAGAGTTATGCTGAGCACTCAAGTGCCTTTGGAGCCAC

CTTTGCTTTTTCTCCTTGAGGAGTACAAAAACTACTTGGATTCTGCAAACATGTCCATGA

GGGTGAGGCGCCACTCTGACCCTGCTCGACGCGGGGAACTGAGCGTCTGTGACAGTATTA

GCGAATGGGTGACTGCGGCCGACAAGAAAACTGCAGTGGACATGGCGGGGCAAACAGTTA

CTGTTCTCGAAAAAGTCCCAGTACCCAAAGGTCAACTGAAACAATATTTCTATGAGACCA

AATGCAATCCTATGGGCTACATGAAGGAAGGTTGCAGGGGCATTGACAAAAGGTACTGGA

ACTCGCAATGCCGAACTACCCAGTCTTATGTGCGTGCGCTCACCATGGATAGCAAAAAAA

AAGTTGGCTGGCGCTTTATAAGGATAGACACTTCTTGTGTATGTACACTGACCATTAAA-

>M. nanapollexa MN534444

CGATGGTTATTTCATACTTAAGTTGCATGAAAGCTGCCCCAATGAAGGAAGCCAATGTCA

GAGGACAAAGTGGCCTGGCTTATCCGGGTCTTCGGACCCATGGTACTCTGGAGAACATAG

GTGGGCCGATAGGTGG---TTCAAGAGGAGGTGGACTTCCATCCCTGACAGATGCCTTTG

AGCAGGTTATAGAGGAACTCCTAGAAGAGGAACAGACCATAAGGCAGAGTGAAGAGAACA

AGGAC---GACATGTATTCATCTAGAGTTATGCTGAGCACTCAAGTGCCTTTGGAGCCAC

CTTTGCTTTTTCTCCTTGAGGAGTACAAAAACTACTTGGATGCTGCAAACATGTCCATGA

GGGTGAGGCGCCACTCTGACCCCGCTCGACGTGGGGAACTGAGCGTCTGTGACAGTATTA

GCGAATGGGTGACTGCGGCCGACAAGAAAACTGCAGTGGACATGGCGGGGCAAACGGTTA

CTGTTCTCGAAAAAGTCCCTGTACCCAAGGGTCAACTGAAACAATATTTCTATGAGACCA

AATGCAATCCTATGGGCTACATGAAGGAAGGTTGCAGGGGCATTGACAAAAGGTACTGGA

ACTCGCAATGCCGAACTACCCAGTCTTATGTGCGCGCGCTCACCATGGATAGCAAAAAAA

AAGTTGGCTGGCGCTTTATAAGGATAGACACTTCTTGTGTATGTACACTGACCATTAAA-

>M. marmorata MN534445

CGATGGTTATTTCATACTTAAGTTGCATGAAAGCTGCCCCAATGAAGGAAGCCAATGTCA

GAGGACAAAGTGGCCTGGCTTATCCGGGTCTTCGGACCCACGGTACTCTGGAGAACATAG

GTGGGCCGATAGGTGG---TTCAAGAGGAGGTGGACTTCCATCACTGACAGATACCTTTG

AGCAGGTTATAGAGGAACTCCTAGAAGAGGAACAGACCATAAGGCAGAGTGAAGAGAACA

AGGAC---GACATGTATTCATCTAGAGTTATGCTGAGCACTCAAGTGCCTTTGGAGCCAC

CTTTGCTTTTTCTCCTTGAGGAGTACAAAAACTACTTGGATGCTGCAAACATGTCCATGA

GGGTGAGGCGCCACTCTGACCCCGCTCGACGTGGGGAACTGAGCGTCTGTGACAGTATTA

GCGAATGGGTGACTGCGGCCGACAAGAAAACTGCAGTGGACATGGCGGGGCAAACAGTTA

CTGTTCTCGAAAAAGTCCCAGTACCCAAAGGTCAACTGAAACAATATTTCTATGAGACCA

AATGCAATCCTATGGGCTACATGAAGGAAGGTTGCAGGGGCATTGACAAAAGGTACTGGA

ACTCGCAATGCCGAACTACCCAGTCTTATGTGCGTGCGCTCACCATGGATAGCAAAAAAA

AAGTTGGCTGGCGCTTTATAAGGATAGACACTTCTTGTGTATGTACACTGACCATTAAA-

>M. arboricola MN534446

CGATGGTTATTTCATACTTAAGTTGCATGAAAGCTGCCCCAATGAAGGAAGCCAATGTCA

GAGGACAAAGTGGCCTGGCTTATCCGAGTCTTCGGACCCACGGTACTCTGGAGAACATAG

GTGGGCCGATAGGTGG---TTCAAGAGGAGGTGGACTTCCATCCCTGACCGATACCTTTG

AGCAGGTTATAGAGGAACTCCTAGAAGAGGAACAGTCAATAAGGCAGAGTGAAGAGAACA

AGGAC---GACATGTATTCATCTAGAGTTATGCTGAGCACTCAAGTGCCTTTGGAGCCAC

CTTTGCTTTTTCTCCTTGAGGAGTACAAAAACTATTTGGATGCTGCAAACATGTCCATGA

GGGTGAGGCGCCACTCTGACCCTGCTCGACGTGGGGAACTGAGCGTCTGTGACAGTATAA

GCGAATGGGTGACTGCGGCCGACAAGAAAACTGCAGTGGACATGGCGGGGCAAACAGTTA

CTGTTCTTGAAAAAGTCCCAGTACCCAAAGGTCAACTGAAACAATATTTCTATGAGACCA

AATGCAATCCTATGGGCTACATGAAGGAAGGTTGCAGGGGCATTGACAAAAGGTACTGGA

ACTCGCAATGCCGAACTACCCAGTCTTATGTGCGTGCGCTCACCATGGATAGCAAAAAAA

AAGTTGGCTGGCGCTTTATAAGGATAGACACTTCTTGTGTATGTACACTGACCATTAAA-

>M. arboricola MN534447

CGATGGTTATTTCATACTTAAGTTGCATGAAAGCTGCCCCAATGAAGGAAGCCAATGTCA

GAGGACAAAGTGGCCTGGCTTATCCGAGTCTTCGGACCCACGGTACTCTGGAGAACATAG

GTGGGCCGATAGGTGG---TTCAAGAGGAGGTGGACTTCCATCCCTGACAGATACCTTTG

AGCAGGTTATAGAGGAACTCCTAGAAGAGGAACAGTCAATAAGGCAGAGTGAAGAGAACA

AGGAC---GACATGTATTCATCTAGAGTTATGCTGAGCACTCAAGTGCCTTTGGAGCCAC

CTTTGCTTTTTCTCCTTGAGGAGTACAAAAACTATTTGGATGCTGCAAACATGTCCATGA

GGGTGAGGCGCCACTCTGACCCCGCTCGACGTGGGGAACTGAGCGTCTGTGACAGTATAA

GCGAATGGGTGACTGCGGCCGACAAGAAAACTGCAGTGGACATGGCGGGGCAAACAGTTA

CTGTTCTCGAAAAAGTCCCAGTACCCAAAGGTCAACTGAAACAATATTTCTATGAGACCA

AATGCAATCCTATGGGCTACATGAAGGAAGGTTGCAGGGGCATTGACAAAAGGTACTGGA

ACTCGCAATGCCGAACTACCCAGTCTTATGTGCGTGCGCTCACCATGGATAGCAAAAAAA

AAGTTGGCTGGCGCTTTATAAGGATAGACACTTCTTGTGTATGTACACTGACCATTAAA-

>M. pulchella MN534448

CGATGGTTATTTCATACTTAAGTTGCATGAAAGCTGCCCCAATGAAGGAAGCCAATGTCA

GAGGACAAAGTGGCCTGGCTTATCCGGGTCTTCGGACCCACGGTACTCTGGAGAACATAG

GTGGGCCGATAGGTGG---TTCAAGAGGAGGTGGACTTCCATCCCTGACAGATACCTTTG

AGCAGGTTATAGAGGAACTCCTAGAAGAGGAACAGTCAATAAGGCAGAGTGAAGAGAACA

AGGAC---GACATGTATTCATCTAGAGTTATGCTGAGCACTCAAGTGCCTTTGGAGCCAC

CTTTGCTTTTTCTCCTTGAGGAGTACAAAAACTACTTGGATGCTGCAAACATGTCCATGA

GGGTGAGGCGCCACTCTGACCCCGCTCGACGTGGGGAACTGAGCGTCTGTGACAGTATTA

GCGAATGGGTGACTGCGGCCGACAAGAAAACTGCAGTGGACATGGCGGGGCAAACAGTTA

CTGTTCTCGAAAAAGTCCCAGTACCCAAAGGTCAACTGAAACAATATTTCTATGAGACCA

AATGCAATCCTATGGGCTACATGAAGGAAGGTTGCAGGGGCATTGACAAAAGGTACTGGA

ACTCGCAATGCCGAACTACCCAGTCTTATGTGCGTGCGCTCACCATGGATAGCAAAAAAA

AAGTTGGCTGGCGCTTTATAAGGATAGACACTTCTTGTGTATGTACACTGACCATTAAA-

>Micryletta nigromaculata MN534449

CTAGGGTTATTTCATACTTAAGTTGCATGAAAGCTGCCCCAATGAAAGAAGCCAATGTCA

GAGGACAAAGTGGCCTGGCTTATCCAGGTCTTCGGACCCACGGTACTTTGGAGAACATAG

GTGGGCCGATAGGTGG---TTCAAGAGGAGGTGGCCTTCCATCCCTGACAGATACCTTTG

AGCATGTTATAGAGGAACTCCTAGAAGAGGAACAGAACATAAGGCAGAGTGAAGAGAACA

AAGACTCAGACATGTATTCATCTAGAGTTATGCTGAGCACTCAAGTGCCTTTGGAGCCGC

CTTTGCTTTTTCTCCTTGAGGAGTACAAAAACTACCTGGATGCTGCAAACATGTCCATGA

GGGTGAGGCGCCACTCTGACCCCGCTCGACGTGGGGAACTGAGCGTCTGTGACAGTATTA

GTGAATGGGTGACAGCAGCAGACAAGAAAACTGCAGTGGACATGGCAGGGCAAACTGTTA

CTGTTCTTGAAAAAGTCCCAGTACCCAAAGGTCAACTGAAACAATATTTCTATGAGACCA

AATGCAATCCTATGGGCTACATGAAGGAAGGTTGCAGGGGCATTGACAAAAGGTACTGGA

ACTCGCAATGCCGAACTACCCAGTCTTATGTGCGTGCGCTCACAATGGATAGCAAAAAAA

AAGTTGGCTGGCGCTTTATAAGGATAGACACTTCTTGTGTATGTACACTGACCATTAAA-
